# ROS-autocatalytic nanoplatform interfering astrocyte-cancer crosstalk and enhancing glioblastoma therapy

**DOI:** 10.1038/s41467-026-77395-2

**Published:** 2026-09-10

**Authors:** Yun Chen, Zonghua Tian, Hongrui Fan, Jingyi Zhou, Shilin Zhang, Xuwen Li, Boyu Su, Chen Jiang, Tao Sun

**Affiliations:** Department of Pharmaceutics, School of Pharmaceutical Sciences, Key Laboratory of Smart Drug Delivery, Ministry of Education, State Key Laboratory of Brain Function and Disorders, MOE Frontiers Center for Brain Science, Minhang Hospital, Fudan University, Shanghai, China

**Keywords:** CNS cancer, Drug delivery, Nanotechnology in cancer

## Abstract

Glioblastoma (GBM) remains incurable and exhibits exceptionally high metabolic demands, driving the evolution of hyperactive mitochondrial systems within tumors. In parallel, mitochondrial transfer from astrocytes to cancer cells fuels metabolic reprogramming and enhances GBM tumorigenicity. These malignant traits markedly constrain therapeutic efficacy and patient survival. Herein, we report a dehydroascorbic acid (DHA)-functionalized, ROS-responsive, ferrocene-integrated polymer nanoplatform (GS@DFP) co-loaded with S-Gboxin and Galunisertib, simultaneously targeting GBM mitochondria and mitochondrial transfer in astrocyte-cancer cell crosstalk. After crossing the blood-brain barrier (BBB) through Glut1-mediated transport enabled by DHA, GS@DFP responds to elevated intracellular reactive oxygen species (ROS) levels in GBM to trigger payload release and initiate Fenton reactions. S-Gboxin inhibits mitochondrial complex V and depletes ATP, whereas Galunisertib downregulates TGF-β/SMAD-driven thrombospondin-1 (TSP-1) expression, suppressing tumor microtube (MT) formation and thereby reducing astrocyte-to-GBM mitochondrial transfer. Notably, TGF-β inhibition also reprograms the immunosuppressive tumor microenvironment (TME). This work establishes a multifunctional nanoplatform that targets mitochondrial vulnerabilities and intercellular crosstalk, offering a translatable strategy for enhancing GBM therapy.

## Abstract (web summary)

Mitochondrial transfer from astrocytes to cancer cells fuels metabolic reprogramming and enhances glioblastoma (GBM) tumorigenicity. Here this group reports a polymeric nanoplatform co-loading S-Gboxin and Galunisertib targeting GBM mitochondria and mitochondrial transfer in astrocyte-cancer cell crosstalk, thereby enhancing GBM therapeutic efficacy.

## Introduction

Glioblastoma (GBM) is the most aggressive primary brain tumor, with a median overall survival (OS) of <15 months despite surgical resection, radiotherapy, and temozolomide (TMZ) chemotherapy^1,2^. A key unmet challenge lies in its unique metabolic adaptability and tumor microenvironment (TME) crosstalk: GBM exhibits higher mitochondrial respiration than normal brain cells, and it hijacks support from native niche cells, particularly astrocytes, to sustain this hypermetabolic state^3–6^. Recent studies have identified astrocyte-to-GBM mitochondrial transfer as a critical driver of GBM progression^7^. Through tumor microtubes (MTs)—cytoplasmic extensions regulated by the TGF-β/SMAD/thrombospondin-1 (TSP-1) pathway—GBM actively acquires mitochondria from astrocytes, expanding its mitochondrial pool, rewiring metabolism, and enhancing resistance to mitochondrial inhibitors^8–10^. TCGA data confirm that GBM patients with high TSP-1 expression exhibit poorer OS, while astrocyte-rich tumor regions exhibit higher mitochondrial content than peritumoral tissue. This intercellular mitochondrial transfer represents a distinct metabolic vulnerability that is not addressed by conventional therapies, highlighting the necessity for strategies that simultaneously disrupt both intrinsic mitochondrial activity and extrinsic mitochondrial acquisition.

Two classes of agents show promise for targeting GBM mitochondria and crosstalk, but their clinical application is hindered by critical flaws. Mitochondrial inhibitors can bind mitochondrial ATP synthase, reducing membrane potential and inducing apoptosis^11,12^. S-Gboxin is a potent inhibitor of oxidative phosphorylation in tumor cells. However, tumor-microenvironment interactions may influence its therapeutic efficacy, suggesting the need for combinational strategies. Another strategy involves TGF-β pathway inhibitors, such as galunisertib, which suppress SMAD2/3 phosphorylation and downregulate TSP-1, reducing MT formation and mitochondrial transfer. Compared with directly targeting axonal proteins like GAP43 or using cytokinins to disrupt the cytoskeleton, modulating the TGF-β-SMAD-TSP-1 pathway offers a safer and more feasible intervention point among these various options^8,13^. However, its hydrophobicity (log *P* = 3.8) and poor blood-brain barrier (BBB) penetration (<0.1% brain accumulation after intravenous injection) limit efficacy^14^.

**Fig. 1:**
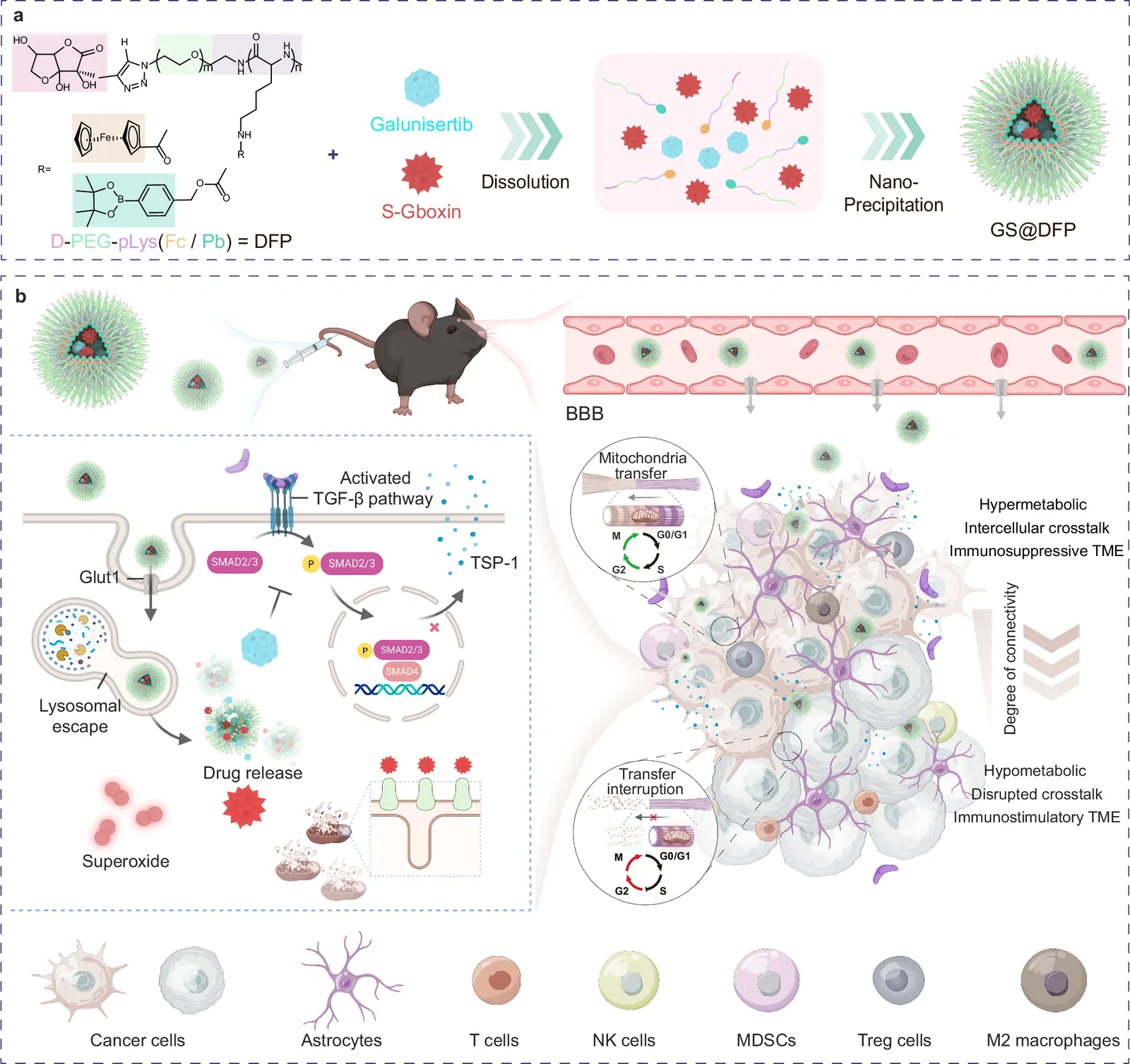
GS@DFP inhibits mitochondria and regulates mitochondrial transfer in GBM. **a** Structure and construction of GS@DFP. **b** After crossing the BBB via DHA-mediated targeting, GS@DFP generates superoxide species and responds to ROS to trigger drug release. S-Gboxin binds to ATP synthase to disrupt mitochondrial function, while galunisertib inhibits SMAD2/3 phosphorylation, thereby downregulating TSP-1 expression, suppressing tumor MT formation, reducing mitochondrial transfer, and concurrently activating antitumor immunity. Illustrations were generated using 3ds Max, Adobe Illustrator, and BioRender.com. Created in BioRender. Jiang, C. (2025). https://BioRender.com/8x6yk6f.

In this work, we develop a multifunctional polymer nanoplatform (GS@DFP) with four key design features, each tailored to solve a specific GBM therapeutic bottleneck. Dehydroascorbic acid (DHA), which binds Glut1—a glucose transporter overexpressed in GBM, is employed as the targeting group enabling receptor-mediated BBB transcytosis and tumor accumulation. Phenylboronic acid ester (Pb) moieties on the polymer backbone undergo hydrolysis in high reactive oxygen species (ROS) environments, which can trigger synchronized release of S-Gboxin and galunisertib. Ferrocene (Fc) acts as a Fenton catalyst, converting GBM’s endogenous H_2_O_2_ into hydroxyl radicals (•OH)—amplifying oxidative stress and overcoming redox homeostasis. S-Gboxin subsequently disrupts intrinsic mitochondria, while galunisertib blocks extrinsic mitochondrial transfer, together resulting in a substantial disruption of the GBM mitochondrial network (Fig. 1). Additionally, TGF-β inhibition reshapes the TME, addressing GBM’s immunosuppressive core. We demonstrate that GS@DFP not only achieves efficient BBB penetration and GBM targeting but also exhibits enhanced antitumor efficacy—prolonging survival in 40% of mice beyond 50 days. This work highlights mitochondrial transfer as a therapeutically actionable vulnerability and provides a strategy for targeting intercellular crosstalk in GBM.

## Results

### Mitochondrial transfer from astrocytes to cancer cells in GBM

**Fig. 2:**
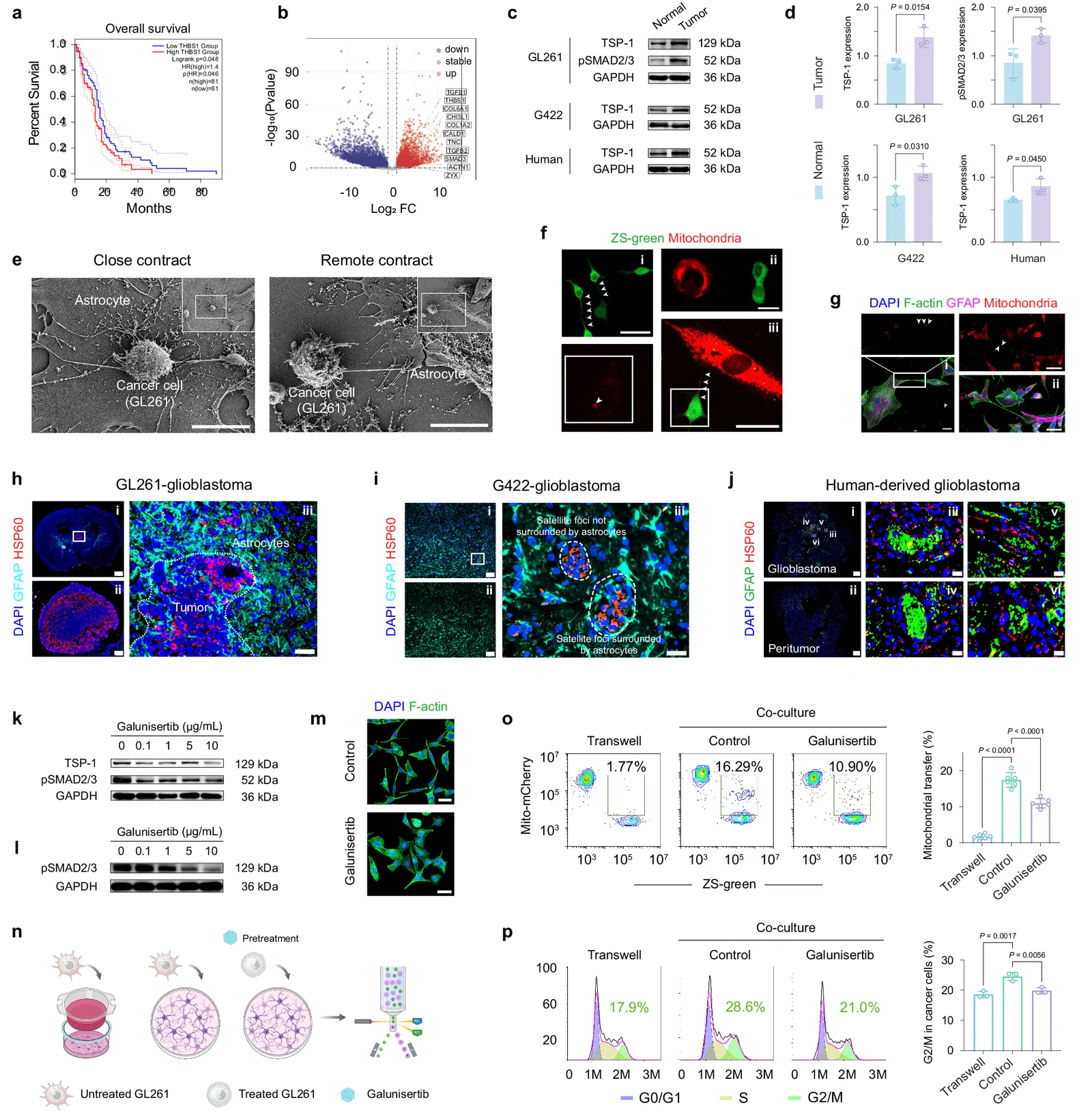
Mitochondrial transfer and regulation in GBM. **a** GIPEA analysis shows correlation between TSP-1 and overall survival. **b** Volcano plot analysis reveals genes associated with tumor MTs. Western blot analysis of pSMAD2/3 and TSP-1 levels in GL261/G422 tumor-bearing mouse models, clinical GBM patients (**c**) and the quantitative results (**d**), (*n* = 3 independent samples). **e** FESEM shows tumor cells and astrocytes connected through tumor MTs.  Scale bar = 50 μm. **f** GL261 cells form intercellular connections (i); mitochondrial transfer does not occur without tumor MTs (ii), while hijacking mitochondria from astrocytes in the presence of MTs (iii).  Scale bar = 20 μm. **g** Punctate mitochondrial signals were observed within tumor MTs. Scale bar = 20 μm. **h** In GL261 tumor-bearing mouse model sections (i), distribution of mitochondria (ii), and tumor tissues adjacent to astrocytes contain more mitochondria (iii). Scale bar = 500 μm (i), 50 μm (ii), 20 μm (iii). **i** In G422 tumor-bearing mouse model sections (i), reactive astrocytes accumulate (ii), and tumor satellite foci surrounded by reactive astrocytes contain more mitochondria (iii). Scale bar = 100 μm (i, ii), 20 μm (iii). **j** In clinical samples of GBM and adjacent normal tissue sections (i, ii), and astrocytes are engulfed (iii, iv), more mitochondria signals are present in the surrounding tumor tissues (v, vi). Scale bar = 1 mm (i, ii), 20 μm (iii–vi). **k**, **l** Western blot showing TSP-1 and pSMAD2/3 expression in GL261 and G422 cells treated with galunisertib. **m** Morphological observation of tumor MTs in G422 cells treated with galunisertib.  Scale bar = 20 μm. **n** Schematic illustration of co-culture conditions. Illustrations were generated using Adobe Illustrator and BioRender.com. **o** The proportion of cancer cells acquiring mitochondria from astrocytes and corresponding quantification (*n* = 6 independent samples). **p** The proportion of cancer cells in different cell-cycle phases and corresponding quantification (*n* = 3 independent samples). Panel **n** was created using BioRender. Created in BioRender. Jiang, C. (2025). https://BioRender.com/8x6yk6f. Data are presented as mean ± SD, and error bars represent SD. One-way ANOVA with Tukey’s multiple comparisons test was used to compare multiple groups, and a two-tailed unpaired Student’s *t* test was used for comparisons between two groups.

**Fig. 3:**
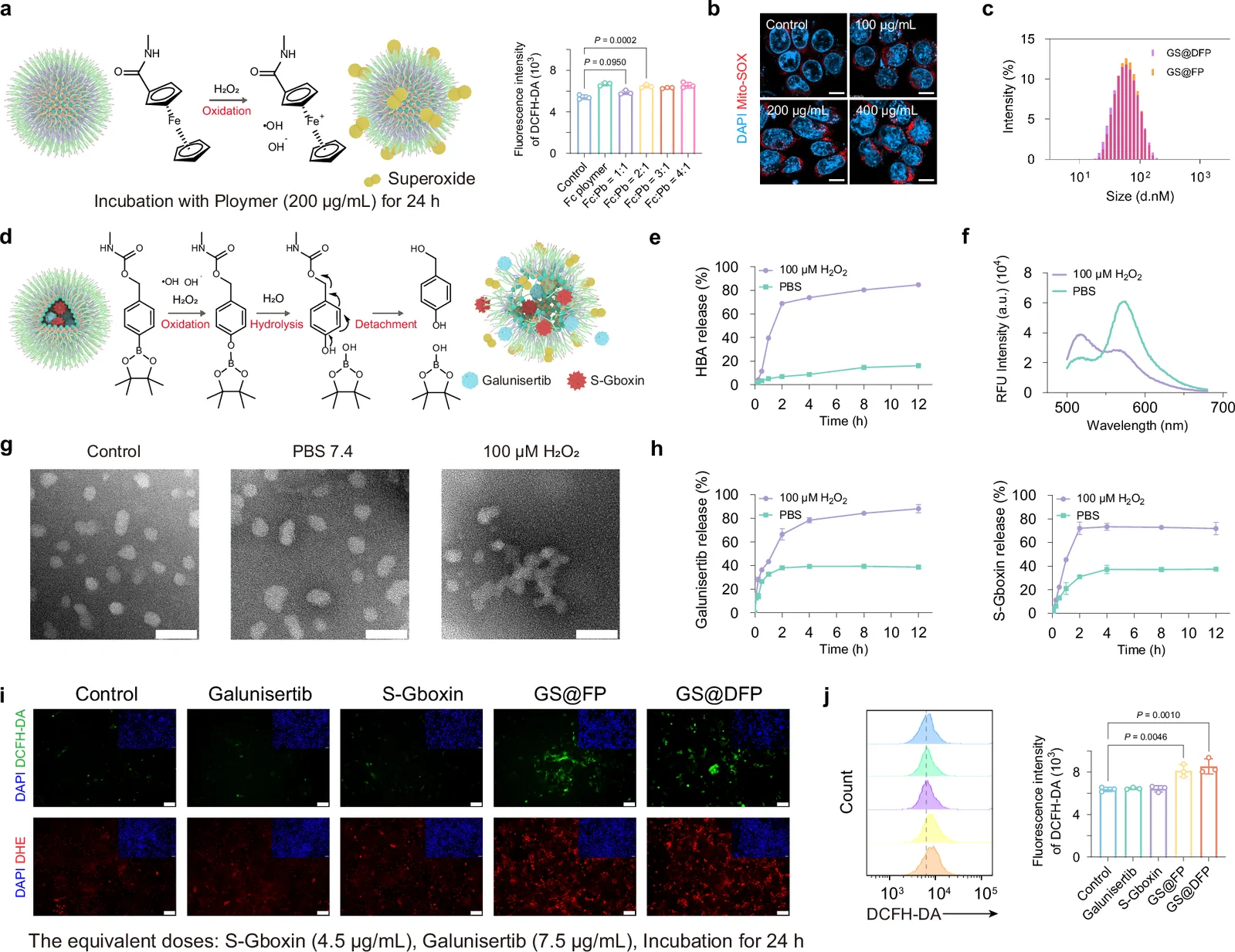
Construction and characterization of GS@DFP. **a** Left: schematic illustration showing the mechanism of superoxide generation by ferrocene within blank nanoparticles. Illustrations were generated using 3ds Max. Right: quantitative analysis of mean fluorescence intensity (MFI) indicating intracellular oxidative stress levels in GL261 cells after incubation with different blank nanoparticles (*n* = 3 independent samples). **b** Blank nanoparticles with a 2:1 ratio induce mitochondrial superoxide accumulation. Scale bar =  20 μm. **c** Hydrodynamic diameters of GS@FP and GS@DFP. **d** Schematic illustration of the ROS-responsive cleavage mechanism of GS@DFP. Illustrations were generated using 3ds Max. **e** Cumulative release profiles of p-hydroxybenzyl alcohol (HBA) from GS@DFP under different conditions (*n* = 3 independent samples). **f** H_2_O_2_ triggers the hydrophilic-hydrophobic transition that alters the emission intensity (535–580 nm) of the fluorescent donor Pb-FITC polymer and the fluorescent acceptor Fc-TAMRA polymer at an excitation wavelength of 460 nm. **g** TEM images of GS@DFP before drug release and after 24 h of release under different conditions. Scale bar = 100 nm. **h** Cumulative release profiles of galunisertib and S-Gboxin from GS@DFP under different conditions (*n* = 3 independent samples). **i** Representative CLSM images showing intracellular oxidative stress indicated by DCFH-DA and DHE probes under different treatment conditions. Scale bar = 100 μm. **j** Representative FCM and quantitative results of MFI for DCFH-DA-stained GL261 cells (*n* = 3 independent samples). Data are presented as mean ± SD, and error bars represent SD. One-way ANOVA with Tukey’s multiple comparisons test was used to compare multiple groups.

The TGF-β/SMAD/TSP-1 pathway is a key regulator of tumor microtube (MT) formation. Analysis of The Cancer Genome Atlas (TCGA) revealed that genes within this pathway are highly upregulated in GBM (Supplementary Fig. 1). Notably, TSP-1 overexpression correlates with a 1.4-fold higher risk of death in GBM patients (Fig. 2a). Enrichment and functional association analyses further indicated that TGF-β-responsive genes are strongly associated with tumor MT formation and are significantly upregulated in GBM compared with normal brain tissue (Fig. 2b and Supplementary Fig. 2). Western blot analysis confirmed that TSP-1 levels were elevated in tumors relative to adjacent normal tissue in various GBM mouse models and human samples (Fig. 3c, d), highlighting the potential therapeutic value of targeting the TGF-β/SMAD/TSP-1 pathway.

To demonstrate MT connections between tumor cells and astrocytes, GL261 cells were co-cultured with mouse astrocytes and examined by field-emission scanning electron microscopy^15,16^. Tubular structures approximately 50–80 μm in length were observed connecting cancer cells to astrocytes (Fig. 2e). Subsequently, mitochondria in astrocytes were labeled with Mito-Tracker Red^17–20^. After co-culturing with ZS-green–transfected GL261 cells, punctate red fluorescence observed within cancer cells confirmed mitochondrial transfer (Fig. 2f). Similarly, fluorescence microscopy revealed mitochondria within tumor MTs connecting G422 cells to astrocytes (Fig. 2g). In orthotopic GBM mouse models and patient-derived tumor sections, mitochondrial and GAP43 fluorescence intensities  were significantly higher in tumor regions compared with peritumoral tissue (Fig. 2g–i and Supplementary Fig. 3). Notably, tumor areas surrounded by reactive astrocytes exhibited enriched mitochondrial content (Fig. 2g–i), further confirming the occurrence of astrocyte-to-tumor mitochondrial transfer in vivo.

To evaluate the effect of galunisertib on MTs, cancer cells were treated with the compound. Following treatment, pSMAD2/3 and TSP-1 expression were markedly downregulated in GL261 cells (Fig. 2k), while pSMAD2/3 levels also decreased in G422 cells (Fig. 2l), accompanied by a significant reduction in tumor MT formation (Fig. 2m). In addition to directly increasing mitochondrial content in tumor cells, mitochondrial transfer promoted proliferation by arresting more cells in the G2/M phase. To further investigate the inhibitory effects of galunisertib on mitochondrial transfer and malignant proliferation^7^, GL261 cells pretreated with galunisertib were co-cultured with astrocytes transfected with Mito-mCherry under different co-culture configurations (Fig. 2n). The results showed that transwell separation almost completely blocked mitochondrial transfer^21,22^, as indicated by double-positive signals close to background levels, demonstrating that direct physical contact between donor and recipient cells is essential for transfer. Compared with the untreated group, galunisertib pretreatment markedly reduced double-positive signals by 34% (Fig. 2o) and the increased proportion of G2/M-phase cells by 72% (Fig. 2p). Collectively, these results indicate that inhibition of TSP-1 suppresses astrocyte-to-tumor mitochondrial transfer and associated malignant proliferation in GBM.

### Preparation and characterization of GS@DFP

To prepare a dual-drug delivery system, PEG-poly(L-lysine) was synthesized as the polymeric backbone (Supplementary Fig. 4), followed by modification with either CDI-linked phenylboronic acid ester^23–25^ or ferrocene carboxylic acid^26–28^ to obtain two types of functionalized polymers (Supplementary Fig. 5). Through a click addition reaction, DHA was conjugated to the Fc polymer^29–31^ as a targeting ligand to achieve GBM-specific delivery (Supplementary Fig. 6). Given that both polymers consume intracellular H_2_O_2_, blank nanoparticles with varying mixing ratios were prepared and incubated with GL261 cells (Supplementary Fig. 7a, b). Starting from a 2:1 ratio, the empty nanoparticles significantly induced oxidative stress in cancer cells (Fig. 3a) while promoting mitochondrial superoxide accumulation in a concentration-dependent manner (Fig. 3b). To determine the optimal DHA-modified Fc polymer ratio, cou-6@DFP (loaded with coumarin 6 probe) was incubated with GL261 cells at varying modification ratios. Quantitative intracellular fluorescence analysis identified 20% modification as optimal (Supplementary Fig. 7c). Using this ratio, drug-loaded nanoparticles were fabricated by nanoprecipitation. The resulting GS@FP and GS@DFP displayed comparable hydrodynamic diameters of approximately 50 nm (Fig. 3c) and zeta potentials of around +4.12 mV (Supplementary Fig. 8a, b). The encapsulation efficiency and drug-loading capacity of GS@DFP were 4.68% for S-Gboxin and 7.27% for galunisertib, respectively, demonstrating stability and biocompatibility (Supplementary Fig. 8c, d).

The ROS-responsiveness of GS@DFP was further evaluated. The phenylboronic acid ester moiety of the Pb polymer underwent consecutive oxidation and hydrolysis under H_2_O_2_ stimulation (Fig. 3d). Accordingly, dual-drug release from GS@DFP was markedly accelerated in the presence of H_2_O_2_ (Fig. 3h), accompanied by rapid generation of p-hydroxybenzyl alcohol (HBA) (Fig. 3e), a cleavage product of the phenylboronic acid ester^25,29^. Transmission electron microscopy (TEM) and dynamic light scattering (DLS) confirmed evident morphological and hydrodynamic changes (Fig. 3g and Supplementary Fig. 7d), verifying the ROS-responsive behavior of GS@DFP. Moreover, fluorescence resonance energy transfer (FRET) analysis demonstrated that H_2_O_2_-triggered phenylboronic acid ester cleavage altered the hydrophilic-hydrophobic balance of the Pb polymer^32,33^, thereby changing the spatial distance between the two polymers and abolishing the FRET effect (Fig. 3f). Similarly, intracellular oxidative stress assessment using DCFH-DA and DHE probes revealed significantly higher ROS generation in GL261 cells treated with GS@DFP compared with untreated or single-drug groups (Fig. 3i, j), confirming the dual functionality of GS@DFP in drug release and oxidative stress induction.

### Intracellular properties and brain targeting capability of nanoparticles

**Fig. 4:**
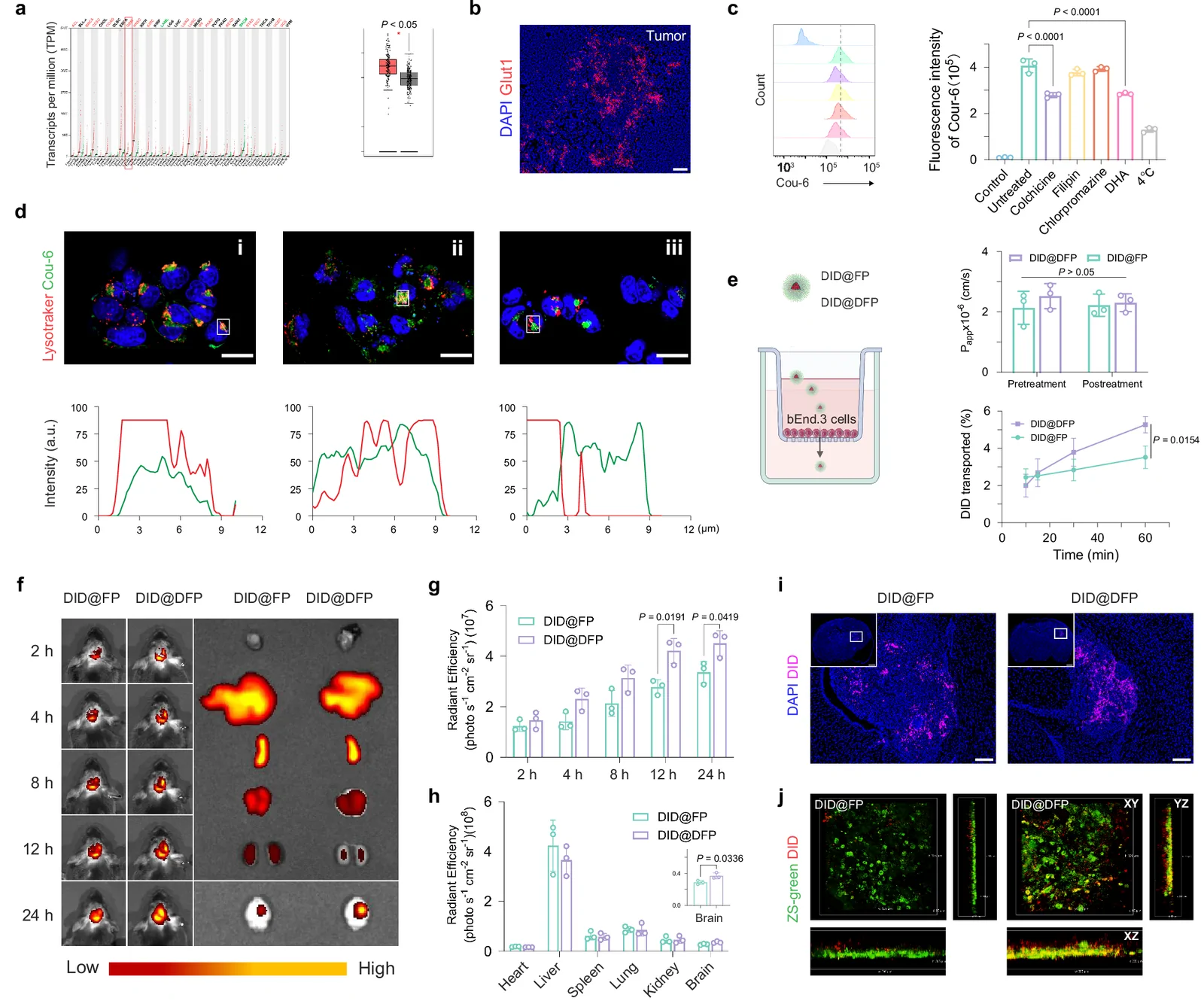
Intracellular properties and brain targeting capability of nanoparticles. **a** Comparison of the expression of Glut1 between tumor and normal tissues using GEPIA, Box plot of Glut1 expression (log_2_(TPM + 1)) in GBM (red, *n* = 163 patient samples) vs. normal tissues (gray, *n* = 207 patient samples). Boxes represent 25th–75th percentiles, centre lines show medians, and whiskers extend to Tukey’s 1.5 × IQR limits (minima/maxima of non-outlier data). **b** Expression of Glut1 in GBM tissues. Scale bar = 100 μm. **c** Left: representative FCM images showing cellular uptake of Cou-6@DFP after different treatments. Right: quantitative analysis of MFI (*n* = 3 independent samples). **d** Lysosomal escape of Cou-6@DFP in GL261 cells after incubation for 0.5 h (i), 2 h (ii), and 4 h (iii), along with quantitative fluorescence tracking analysis. Intensity is measured in arbitrary units (a.u.).  Scale bar = 20 μm. **e** Left: schematic illustration of the in vitro BBB model. Illustrations were generated using Adobe Illustrator and BioRender.com. Right: permeability coefficient of the BBB model (*n* = 3 independent samples), and DID accumulation in the lower chamber of the BBB model (*n* = 3 independent samples). **f** Left: fluorescence images of DID accumulation in the brain at different time points after intravenous injection of DID@FP and DID@DFP. Right: in vitro organ fluorescence imaging at 24 h. **g** Quantification of fluorescence intensity at the tumor site within 24 h (*n* = 3 mice in each group). **h** Quantification of fluorescence intensity in different ex vivo organs at 24 h (*n* = 3 mice in each group). **i** Frozen-section images showing tumor accumulation of DID.  Scale bar = 200  μm. **j** Two-photon in vivo microscopy showing the accumulation of DID in tumor regions. Panel **e**  was created using BioRender. Created in BioRender. Jiang, C. (2025). https://BioRender.com/8x6yk6f. Data are presented as mean ± SD, and error bars represent SD. One-way ANOVA with Tukey’s multiple comparisons test was used to compare multiple groups, and a two-tailed unpaired Student’s *t* test was used for comparisons between two groups.

Pan-cancer analysis using the GIPEA database revealed that GBM exhibits intrinsic glucose avidity^30^, characterized by elevated expression of glucose transporters, such as Glut1 (Fig. 4a). Fluorescence staining of GL261 tumor sections further demonstrated high Glut1 expression within tumor regions (Fig. 4b), which provides a theoretical basis for our use of DHA-modified nanoparticles. To investigate the internalization mechanism of DHA-modified nanoparticles, GL261 cells were pretreated with various endocytosis inhibitors, DHA, or under cold conditions before incubation with Cou-6@DFP (loaded with Coumarin-6 probe). The results indicated that Cou-6@DFP was actively internalized via macropinocytosis with DHA assistance (Fig. 4c). Lysosomal escape was verified by colocalization analysis using Lyso-Tracker Red and confocal laser scanning microscopy (CLSM), showing efficient lysosomal escape of Cou-6@DFP (Fig. 4d). Subsequently, we established an in vitro BBB Transwell model by bEnd.3 cells, and DID@DFP (loaded with 1,1′-dioctadecyl-3,3,3′,3′-tetramethylindodicarbocyanine probe) was prepared. After confirming the formation of a compact monolayer by bEnd.3 cells, nanoparticle translocation across the BBB was quantified (Fig. 4e). Compared with DID@FP, DID@DFP exhibited enhanced BBB penetration efficiency (Fig. 4e), confirming the DHA-mediated BBB crossing capability.

Then, we tested the brain tumor targeting ability of DHA-modified nanoparticles. Following intravenous injection of DID@FP or DID@DFP into GL261-bearing mice, in vivo fluorescence imaging demonstrated progressively increased tumor accumulation of DID over time (Fig. 4f, h), with stronger fluorescence observed in the DID@DFP group. This was consistent with in vitro organ fluorescence quantification and frozen-section imaging results (Fig. 4g, i). Furthermore, ZS-green GL261 orthotopic mouse models were established to evaluate tumor targeting in vivo. After administration of DID@FP or DID@DFP, fluorescence signals at the tumor site were visualized using two-photon fluorescence microscopy. At a depth of 100 μm below the cortex, within an 85  μm-thick tumor cell layer, the DID@DFP group exhibited significantly greater DID signal accumulation (Fig. 4j and Supplementary Fig. 9), further confirming a contributory role of DHA in tumor targeting, potentially involving Glut1-mediated transport among other pathways.

### GS@DFP disrupts mitochondria and inhibits cell proliferation

**Fig. 5:**
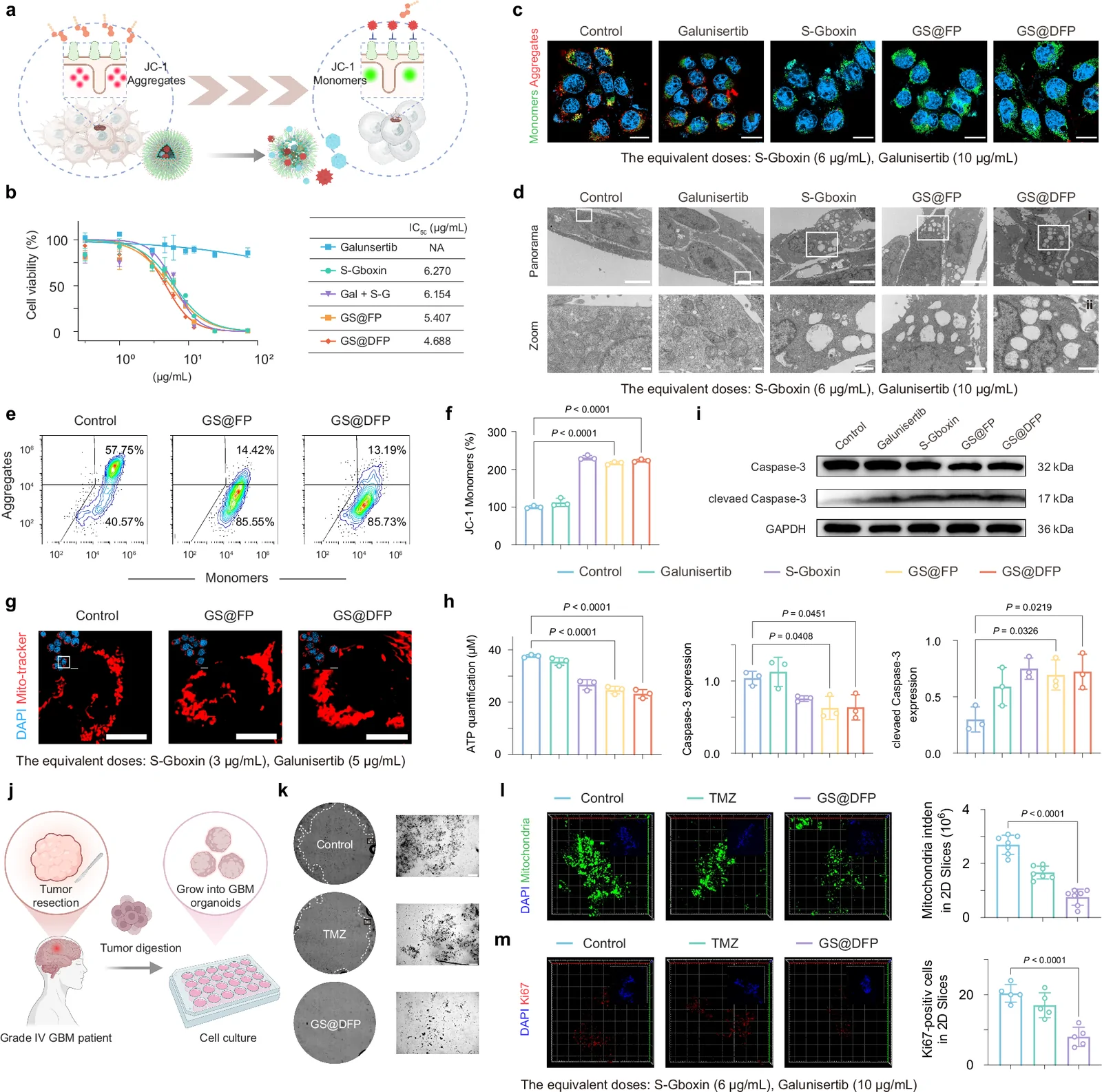
GS@DFP disrupts mitochondria and inhibits cell proliferation. **a** Schematic illustration of mitochondrial disruption and JC-1-indicated membrane potential changes. **b** Fitted cell viability curves and half-maximal inhibitory concentrations (IC₅₀) of GL261 cells after treatment with galunisertib, S-Gboxin, galunisertib + S-Gboxin, GS@FP, or GS@DFP for 24 h (*n* = 4 independent samples). **c** Representative CLSM images of JC-1-stained GL261 cells after different treatments for 6 h. Scale bar = 20 μm. **d** TEM images showing mitochondrial morphological changes in cancer cells after different treatments for 12 h.  Scale bar = 5 μm in (i). Scar bar = 1 μm in (ii). Representative flow cytometry plots of JC-1 staining (**e**) and quantitative analysis of JC-1 monomers (**f**) in GL261 cells (*n* = 3 independent samples). **g** CLSM images of mitochondrial morphology in GL261 cells after treatments. Scale bar = 10 μm. **h** Intracellular ATP levels of GL261 cells measured after different treatments for 12 h (*n* = 3 independent samples). **i** Western blot analysis and quantitative results of apoptosis-related proteins Caspase-3 and cleaved Caspase-3 following mitochondrial disruption (*n* = 3 independent samples). **j** Establishment of patient-derived tumor organoid models from primary WHO grade IV GBM tissues. **k** Panoramic and representative local images showing the effects of TMZ or GS@DFP treatment for 48 h on GBM organoid formation. Scale bar = 100 μm. **l** Representative 3D mitochondrial staining images and fluorescence quantification of GBM organoids after different treatments (*n* = 7 independent slices). **m** Representative 3D Ki67 fluorescence staining images and quantification of Ki67⁺ cells in GBM organoids after different treatments (*n* = 5 independent slices). Panels **a**, **j**  were created using BioRender. Created in BioRender. Jiang, C. (2025). https://BioRender.com/8x6yk6f. Data are presented as mean ± SD, and error bars represent SD. One-way ANOVA with Tukey’s multiple comparisons test was used to compare multiple groups.

It has been reported that S-Gboxin can bind to ATP synthase on the mitochondrial inner membrane, reducing membrane potential and damaging mitochondria, and thereby inhibiting oxidative phosphorylation in cancer cells and promoting cell death (Fig. 5a). Cell viability of cancer cells after treatment with galunisertib, S-Gboxin, galunisertib/S-Gboxin, GS@FP, or GS@DFP was assessed using the Cell Counting Kit-8 (CCK-8) assay. Galunisertib exhibited no significant cytotoxicity, while GS@DFP showed the lowest half-maximal inhibitory concentration (IC₅₀)^12^, likely due to DHA-mediated cellular uptake combined with ferrocene-induced oxidative stress (Fig. 5b). As a mitochondrial membrane potential probe, JC-1 shifts from red fluorescence in the aggregated state to green fluorescence in the monomer state upon membrane depolarization (Fig. 5a). Strong green fluorescence was observed in cells treated with GS@FP or GS@DFP, indicating that S-Gboxin’s ability to induce membrane depolarization was retained (Fig. 5c). Subsequent flow cytometry analysis confirmed the clear transition from red to green fluorescence (Fig. 3e, f, and Supplementary Fig. 10a). To observe direct mitochondrial damage within cells, mitochondria were stained with the Mito-Tracker Red^34^. Compared with the normal tubular mitochondrial structures in untreated cells, dye leakage was observed in cells treated with low-dose S-Gboxin, GS@FP, or GS@DFP, indicating disruption of mitochondrial integrity (Fig. 5g and Supplementary Fig. 10b). Further evaluation of intracellular ATP levels using an ATP assay kit revealed a significant decrease in ATP content under all three treatments (Fig. 5h).

Mitochondrial depolarization can trigger pro-death autophagy, subsequently activating apoptosis-related proteins and inducing cell apoptosis. Bio-transmission electron microscopy (bio-TEM) revealed that high-dose S-Gboxin caused notable mitochondrial cristae disruption and subsequent dissolution, with similar phenomena observed in the GS@FP- or GS@DFP-treated groups (Fig. 5d). Representative CLSM images of autophagy-related proteins confirmed pro-death autophagy resulting from mitochondrial damage (Supplementary Fig. 11). To verify the subsequent activation of apoptosis, Western blot analysis was performed to detect Caspase-3 and cleaved Caspase-3 (Fig. 5i). The results showed that Caspase-3 was partially cleaved into cleaved Caspase-3 in cells treated with S-Gboxin, GS@FP, or GS@DFP, thereby inducing apoptosis (Fig. 5j). Collectively, these data indicate that GS@FP and GS@DFP efficiently damage mitochondria and effectively promote cancer cell apoptosis.

Organoids are widely recognized as advanced in vitro models for evaluating drug efficacy^35–37^. To assess the therapeutic potential of GS@DFP against GBM, GBM organoid models were constructed using tumor tissues derived from primary grade IV GBM patients (Fig. 5k). The organoids were treated with either TMZ or GS@DFP, and their effects on organoid growth were evaluated. Compared with the control group, TMZ slightly reduced organoid formation, yet a considerable number of organoid clusters remained. In contrast, GS@DFP markedly induced organoid necrosis and substantially decreased organoid formation (Fig. 5l). Formed organoids were then treated with TMZ or GS@DFP, and mitochondrial content was assessed using the Mito-Tracker Green. The results demonstrated that GS@DFP effectively disrupted mitochondria within the organoids (Fig. 5m). Similarly, the proliferative capacity of organoids after different treatments was evaluated using the Ki67 probe. GS@DFP exhibited the strongest proliferation-inhibitory effect, as indicated by the lowest red fluorescence signal (Fig. 5n). Collectively, these results confirm that GS@DFP Defficiently damages mitochondria in GBM organoids and inhibits their proliferation, demonstrating the broad cancer cell-killing capability of GS@DFP.

### GS@DFP downregulates mitochondrial transfer and malignant proliferation

**Fig. 6:**
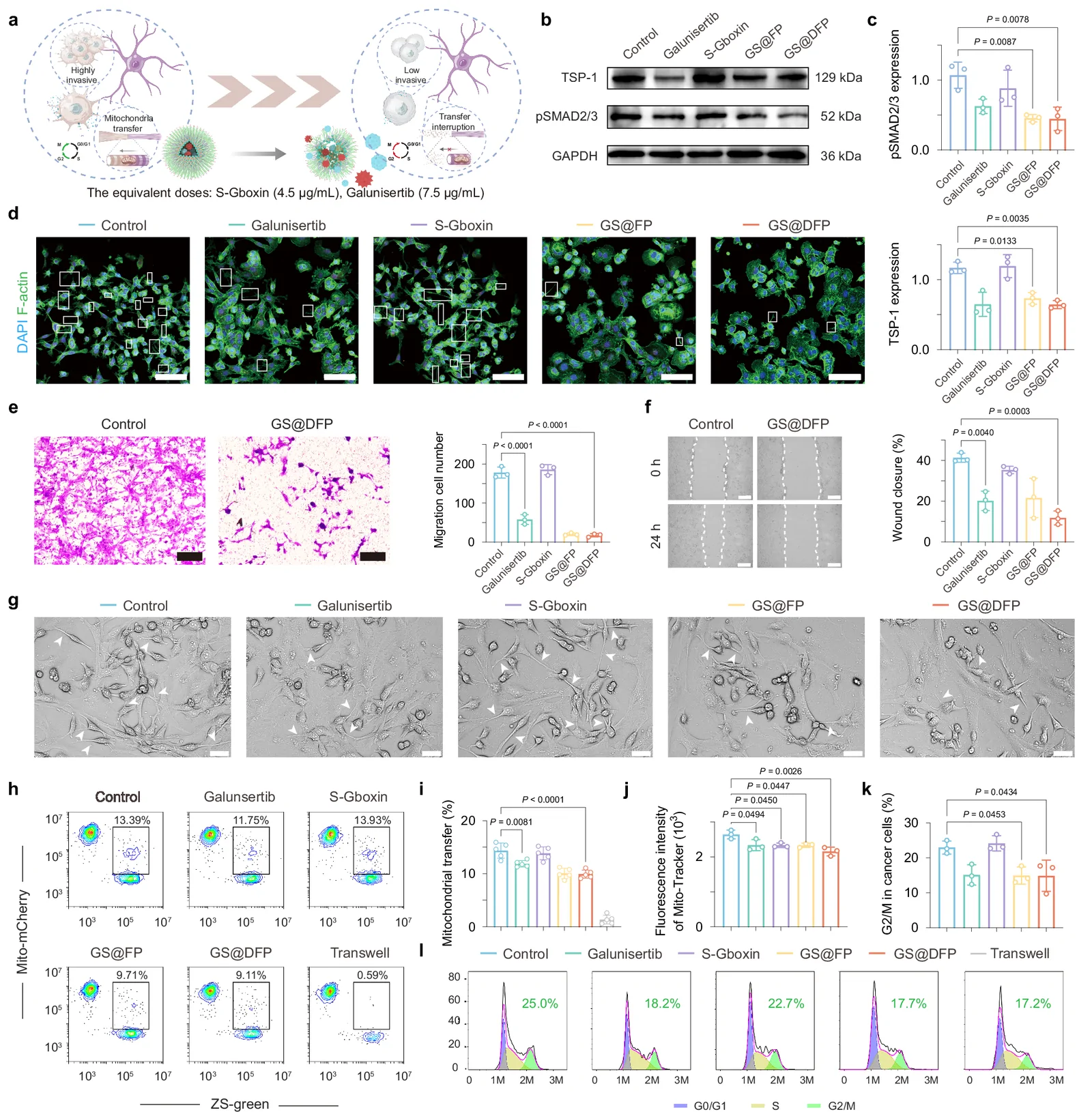
GS@DFP inhibits tumor MTs, thereby downregulating mitochondrial transfer and malignant proliferation. **a** Schematic illustration showing that GS@DFP inhibits tumor MTs, thereby weakening cancer cell invasion, reducing mitochondrial transfer, and decreasing tumorigenicity. Illustrations were generated using 3ds Max, Adobe Illustrator and BioRender.com. Western blot analysis of GL261 cells treated with galunisertib, S-Gboxin, GS@FP, or GS@DFP for 24 h, showing the expression of pSMAD2/3 and TSP-1 (**b**) and the quantitative results (**c**) (*n* = 3 independent samples). **d** Tumor MT expression in GL261 cells after different treatments for 24 h. Scale bar = 100 μm. **e** Representative images and quantification of invasive GL261 cells stained with crystal violet 24 h after incubation with different treatments, determined by Transwell assay (*n* = 3 independent samples). Scale bar = 100 μm. **f** Representative images and quantification of migratory GL261 cells after incubation with different treatments for 24 h, determined by wound-healing assay (*n* = 3 independent samples). Scale bar = 20 μm. **g** Representative images showing tumor MT-mediated connections between cancer cells and astrocytes after 24 h of pretreatment. Scale bar  = 20 μm. Representative flow cytometry plots (**h**) and quantitative analysis (**i**) showing the proportion of ZS-green GL261 cells receiving astrocyte-derived mitochondria after co-culture with Mito-mCherry-transfected astrocytes (*n* = 5 independent samples). **j** Flow cytometric quantification of mitochondrial content in ZS-green GL261 cells after co-culture with astrocytes (*n* = 3 independent samples). Flow cytometric plots (**l**) and quantitative results (**k**) of cell cycle phases in ZS-green GL261 cells after co-culture with astrocytes (*n* = 3 independent samples). Panel **a**  was created using BioRender. Created in BioRender. Jiang, C. (2025). https://BioRender.com/8x6yk6f. Data are presented as mean ± SD, and error bars represent SD. One-way ANOVA with Tukey’s multiple comparisons test was used to compare multiple groups.

As crucial cytoplasmic extensions of cancer cells, tumor MTs play a vital role in cancer cell invasion and migration^8^, and also serve as conduits for direct mitochondrial acquisition from astrocytes (Fig. 6a). Western blot analysis confirmed that, similar to galunisertib (Fig. 6b), both GS@FP and GS@DFP effectively downregulated the expression of MT-associated proteins pSMAD2/3 and TSP-1 in GL261 cells (Fig. 6c). Consistently, CLSM revealed a significant inhibition of MT formation (Fig. 6d). Inhibition of these protrusive structures markedly weakened the invasive and migratory capacities of tumors^8^, as evidenced by decreased transmembrane invasion (Fig. 6e and Supplementary Fig. 12a, b) and reduced wound-healing ability of GL261 cells (Fig. 6f and Supplementary Fig. 12c, d). Subsequently, co-culturing cancer cells with astrocytes under different treatments revealed markedly reduced tumor MT connections (Fig. 6g). These results demonstrate that GS@FP and GS@DFP effectively reduce tumor MT formation, thereby inhibiting cancer cell proliferation and migration while directly diminishing connections between cancer cells and astrocytes.

To demonstrate that GS@DFP effectively mediates the downregulation of mitochondrial transfer following MT inhibition, Mito-mCherry-transfected astrocytes were co-cultured with pretreated ZS-Green GL261 cells, and the proportion of GL261 cells receiving astrocyte-derived mitochondria was quantified based on double-positive fluorescence signals (Fig. 6h and Supplementary Fig. 13). Consistent with previous findings, groups treated with galunisertib-containing formulations exhibited a reduced proportion of double-positive cells (Fig. 6i and Supplementary Fig. 16a). This downregulation was further validated in co-culture systems using Mito-Tracker Red-labeled astrocytes and carboxyfluorescein diacetate succinimidyl ester (CFSE)-stained cancer cells (Supplementary Fig. 14) and different transfection reagents (Supplementary Fig. 15). We next quantified intracellular mitochondrial content in cancer cells after co-culture with astrocytes. Results showed that S-Gboxin induced intrinsic mitochondrial damage in cancer cells, while galunisertib reduced mitochondrial transfer by inhibiting MT formation. Notably, GS@DFP, which co-delivers both drugs, exhibited the lowest mitochondrial content (Fig. 6j and Supplementary Fig. 16b), highlighting the superiority of the combined therapeutic strategy. Furthermore, analysis of cell-cycle distribution revealed that inhibition of mitochondrial transfer by GS@FP and GS@DFP significantly reduced the proportion of tumor cells in the G2/M phase (Fig. 6k, l, and Supplementary Fig. 16c), suggesting a reversal of metabolic reprogramming and attenuation of tumorigenicity. Overall, these results confirm that GS@FP and GS@DFP effectively inhibit mitochondrial transfer from astrocytes and counteract the associated malignant proliferative effects in cancer cells.

### GS@DFP disrupts mitochondria in GBM and downregulates mitochondrial transfer

**Fig. 7:**
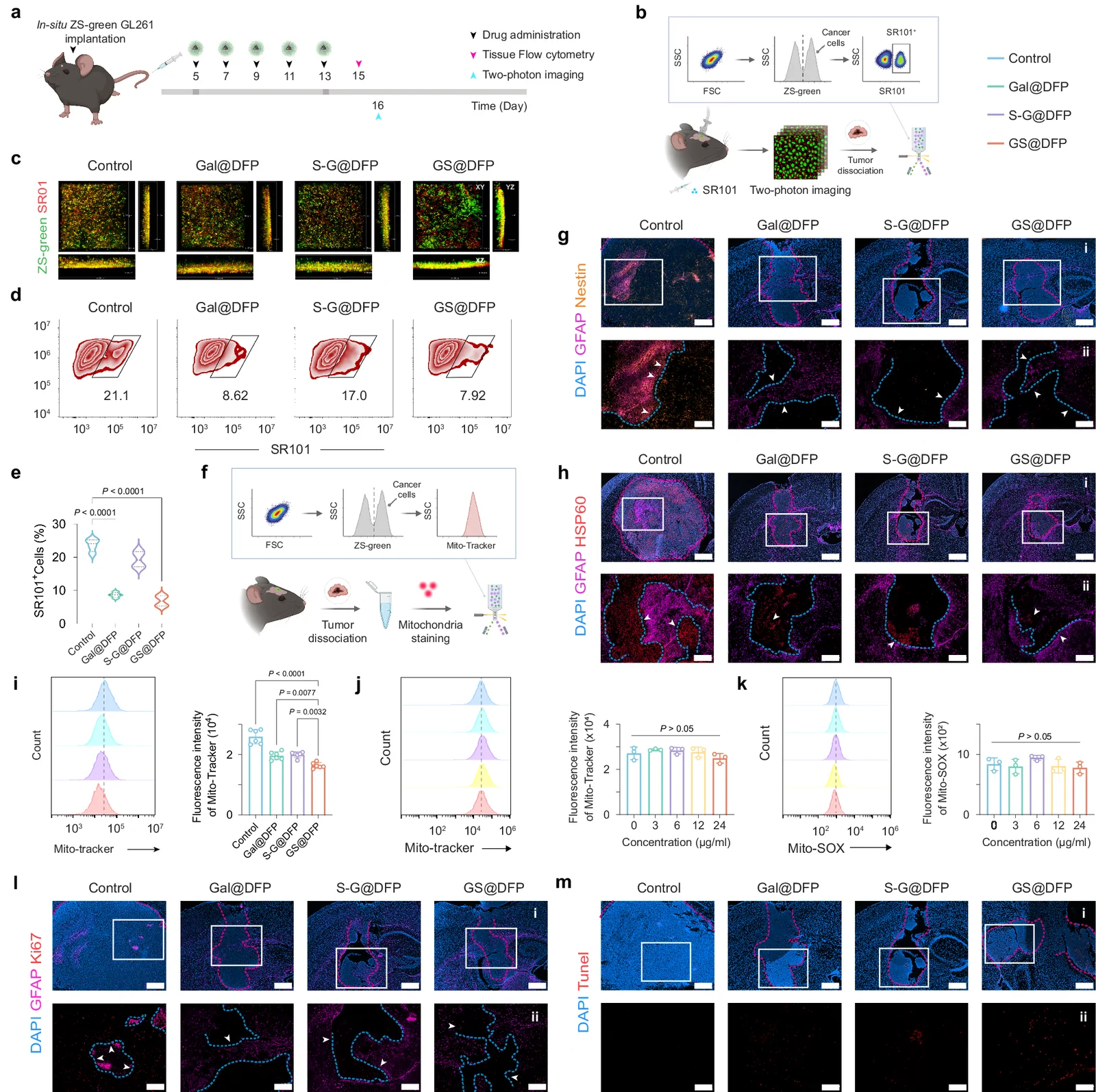
GS@DFP disrupts mitochondria in tumor tissues and downregulates mitochondrial transfer. **a** Schematic timeline of ZS-Green GL261 orthotopic tumor-bearing mouse model establishment and treatments. **b** Schematic diagram showing tumor observation by two-photon confocal microscopy after intraperitoneal injection of SR101 and the flow cytometry gating strategy. **c** In vivo observation of uptake of SR101 by cancer cells from astrocytes under two-photon microscopy. Representative flow cytometry plots (**d**) and quantitative analysis (**e**) of SR101⁺ cancer cells (*n* = 4 mice in each group). **f** Schematic diagram of mitochondrial content measurement in orthotopic tumor-bearing mice and flow cytometry gating strategy. Fluorescence staining analysis of the astrocyte and MT-associated protein Nestin (**g**), fluorescence staining analysis of the astrocyte and mitochondria-associated protein Heat Shock Protein 60 (HSP60) (**h**). Tumor regions are indicated by red dashed lines in (i) (scale bar = 200  μm) and by blue dashed lines in (ii) (scale bar = 100 μm). **i** Representative FCM plots and quantitative results of mitochondrial content in tumors from different treatment groups (*n* = 6 mice in each group). Representative FCM plots and quantitative results showing mitochondrial quantity (**j**) and quality (**k**) in GL261 cells treated with different concentrations of galunisertib (*n* = 3 independent samples). Fluorescence staining analysis of astrocyte and Ki67 protein expression (**l**), fluorescence staining analysis of apoptosis-related signal expression (**m**). Tumor regions are indicated by red dashed lines in (i) (scale bar = 200 μm) and by blue dashed lines in (ii) (scale bar = 100 μm). Panels **a**, **b**, **f**  were created using BioRender. Created in BioRender. Jiang, C. (2025). https://BioRender.com/8x6yk6f. Data are presented as mean ± SD, and error bars represent SD. One-way ANOVA with Tukey’s multiple comparisons test was used to compare multiple groups.

Given that  GS@DFP effectively inhibits mitochondrial activity in cancer cells and downregulates mitochondrial transfer from astrocytes to cancer cells in vitro, we evaluated the MT inhibition capacity of  GS@DFP in ZS-green GL261 tumor-bearing mouse models (Fig. 7a). Sulforhodamine 101 (SR101) is a red fluorescent dye that efficiently and specifically labels astrocytes, making it widely used in neuroimaging studies. Interestingly, cancer cells can specifically take up SR101 from astrocytes via tumor MTs^38,39^, so the uptake level reflects the connectivity between astrocytes and cancer cells (Fig. 7b). After completion of the treatment regimen, tumor-bearing mice with pre-implanted cranial windows were administered SR101, and in vivo two-photon microscopy showed that both Gal@DFP (loaded with galunisertib alone) and GS@DFP (loaded with dual drugs) exhibited suppression of tumor MTs, indicated by a marked reduction in colocalization of red SR101 and green cancer cell signals (Fig. 7c and Supplementary Fig. 18), as well as lower proportions of SR101^+^ cells (Fig. 7d, e, and Supplementary Fig. 17a). Similarly, Gal@DFP and GS@DFP significantly downregulated the expression of the MT-associated protein Nestin in tumor tissues (Fig. 7g and Supplementary Fig. 19a), confirming that galunisertib can effectively suppress tumor MT formation and downregulate connections between cancer cells and astrocytes in vivo with the assistance of the delivery system.

S-Gboxin can disrupt mitochondria within tumors, and tumor MT inhibition also downregulates mitochondrial transfer, thereby reducing mitochondrial content in tumor tissues. Therefore, we assessed mitochondrial content in tumors of orthotopic ZS-green GL261 tumor-bearing mice after various treatments. Using Mito-Tracker Red to stain homogenized brain tissue, the cancer cells were distinguished by their green fluorescence through flow cytometry (Fig. 7f and Supplementary Fig. 17b), and all three treatment groups showed reduced mitochondrial content. Gal@DFP reduced externally derived mitochondria through galunisertib-mediated suppression of mitochondrial transfer, whereas S-G@DFP directly eliminated mitochondria originating from the cancer cells themselves via S-Gboxin. Consistent with our previous in vitro cell experimental results, tumors treated with GS@DFP, combining the dual-drug effect, exhibited the lowest mitochondrial content (Fig. 5i). Interestingly, compared with the treated groups, untreated mice exhibited more pronounced mitochondrial signals in tissues adjacent to astrocytes. After Gal@DFP treatment, mitochondrial signals were primarily enriched in the tumor core, with almost no mitochondrial signal in tumor regions near astrocytes, further indicating downregulation of mitochondrial transfer. Conversely, in mice treated with S-G@DFP, mitochondrial signals largely appeared in regions adjacent to astrocytes. In mice treated with GS@DFP, mitochondrial signals were weak both in the tumor core and in regions close to astrocytes (Fig. 7h and Supplementary Fig. 19b). To exclude a direct effect of galunisertib on mitochondria, cancer cells were treated in vitro with different concentrations of galunisertib, which did not directly affect mitochondrial quantity (Fig. 7j) or quality (Fig. 7k). These results further demonstrate that GS@DFP downregulates mitochondrial transfer in vivo primarily via inhibition of tumor MTs.

We have demonstrated in vitro that downregulating mitochondrial transfer suppresses malignant proliferation driven by metabolic reprogramming in cancer cells. We further investigated whether inhibition of mitochondrial transfer could also reduce astrocyte-mediated malignant tumor support. In tumor-bearing mice treated with Gal@DFP, Kiel 67 (Ki67) proliferation signals in tumor tissues were markedly downregulated. S-G@DFP, through mitochondrial disruption, also suppressed tumor proliferation, while dual-drug GS@DFP treatment exhibited the lowest Ki67 signals (Fig. 7l and Supplementary Fig. 19c). We further examined tumor apoptosis under different treatments, and all three treatment groups displayed enhanced pro-apoptotic activity (Fig. 7m and Supplementary Fig. 19d). The downstream events of proliferation inhibition and apoptosis induction collectively demonstrate the enhanced antitumor efficacy of targeting tumor mitochondria and mitochondrial transfer strategies.

### GS@DFP inhibits tumor growth and prolongs survival in GBM models

**Fig. 8:**
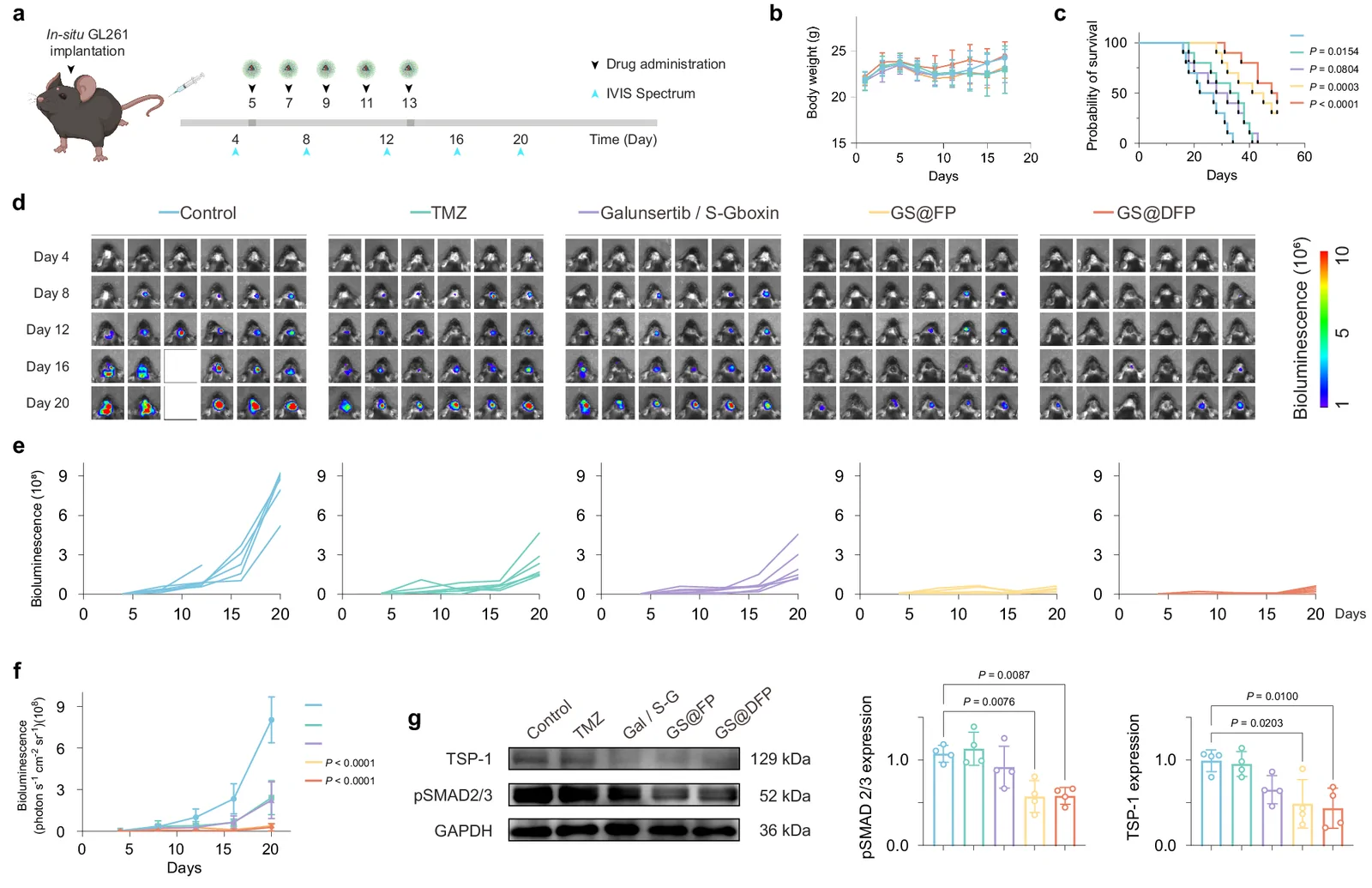
GS@DFP effectively inhibits tumor growth in vivo. **a** Schematic illustration of the establishment and treatment schedule of the GL261-Luci tumor-bearing mouse model. **b** Body weight changes of tumor-bearing mice treated with PBS, TMZ, equivalent doses of galunisertib and S-Gboxin via intravenous injection, GS@FP, or GS@DFP over 20 days (*n* = 6 mice in each group). **c** Survival curves of tumor-bearing mice with different treatment (*n* = 10 mice in each group). **d** In vivo bioluminescence imaging of GL261 tumor-bearing mice, with black boxes representing deceased mice. **e** Quantitative analysis of normalized individual brain bioluminescence intensity of tumor-bearing mice after different treatments (*n* = 6 mice in each group). **f** Quantitative results of brain bioluminescence imaging of tumor-bearing mice (*n* = 6 mice in each group). **g** Western blot analysis and quantitative results of pSMAD2/3 and TSP-1 expression in tumor tissues from each treatment group (*n* = 4 mice in each group). Panel **a**  was created using BioRender. Created in BioRender. Jiang, C. (2025). https://BioRender.com/8x6yk6f. Data are presented as mean ± SD, and error bars represent SD. One-way ANOVA with Tukey’s multiple comparisons test was used to compare multiple groups.

Given that GS@DFP effectively downregulated mitochondrial transfer both in vitro and in vivo, thereby preventing cancer cells from obtaining support from astrocytes, we evaluated the anticancer activity of GS@DFP in GL261-Luci tumor-bearing mouse models (Fig. 8a). Concurrently, the chemotherapy drug TMZ was selected as the clinical treatment group for comparison. Treatments were administered after tumor inoculation, during which tumor bioluminescence signals were repeatedly monitored (Fig. 8d). The results showed that both GS@FP and GS@DFP markedly inhibited tumor growth, achieving enhanced antitumor effects compared with the free-drug combination and TMZ (Fig. 8b, e). Intravenous injection of the free dual drugs combination at equivalent doses did not significantly improve the survival of tumor-bearing mice, while TMZ only marginally extended survival. In contrast, approximately 30% of mice in the GS@FP and GS@DFP groups remained alive beyond 50 days, with the GS@DFP group achieving the longest median survival time (Fig. 8c). During tumor progression, minor fluctuations in body weight were observed across all treatment groups (Fig. 8b). Biochemical index determination (Supplementary Fig. 20) and hematoxylin-eosin (H&E) staining (Supplementary Figs. 21 and 22) confirmed the absence of noticeable tissue toxicity following the treatments. Collectively, these results demonstrate that GS@DFP exhibits potent antitumor efficacy while maintaining biosafety.

### GS@DFP activates the tumor immune microenvironment

**Fig. 9:**
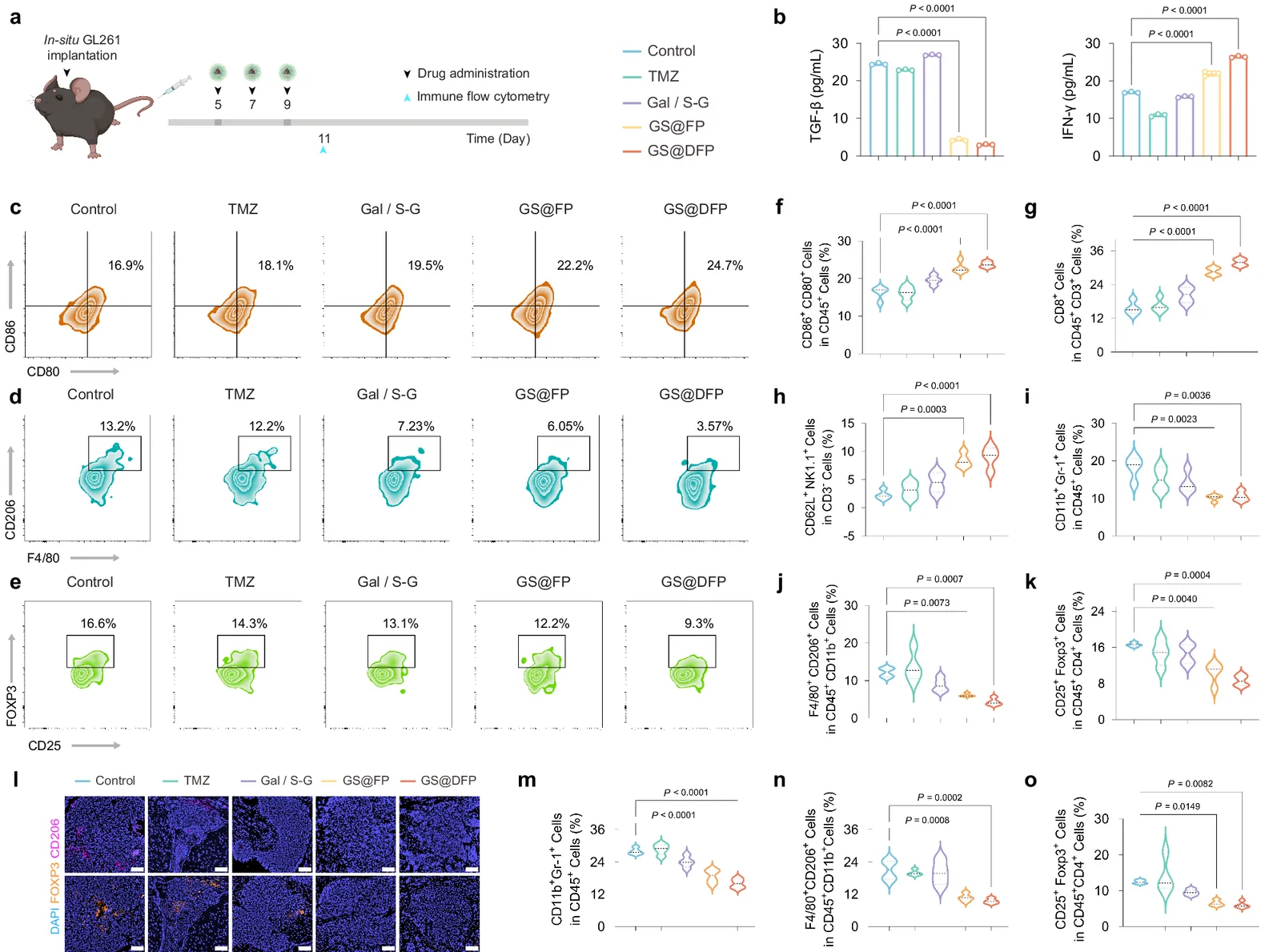
GS@DFP activates the tumor immune microenvironment. **a** Schematic illustration of the GL261-Luci tumor-bearing mouse model establishment, treatment schedule and subsequent immunological analyses. **b** ELISA measurements of TGF-β and IFN-γ secretion in GBM after treatment completion (*n* = 3 mice in each group). Representative flow cytometry plots showing DC maturation (**c**) and the intratumoral infiltration of M2 macrophages (**d**) and Treg cells (**e**) in GBM. **f** Flow cytometry analysis of the proportion of mature DCs in cervical lymph nodes (*n* = 4 mice in each group). **g**–**k** Flow cytometry analysis of the infiltration of immune effector cells (T cells, NK cells) and immunosuppressive cells (MDSCs, M2 macrophages, and Treg cells) in GBM (*n* = 4 mice in each group). **l** Fluorescence staining of FOXP3 and CD206 expression in orthotopic GL261 tumor-bearing mice after treatment. Scale bar = 100 μm. **m**–**o** Flow cytometry analysis of the infiltration of MDSCs, M2 macrophages, and Treg cells in GBM (*n* = 4 mice in each group). Panel **a** was created using BioRender. Created in BioRender. Jiang, C. (2025). https://BioRender.com/8x6yk6f. Data are presented as mean ± SD, and error bars represent SD. One-way ANOVA with Tukey’s multiple comparisons test was used to compare multiple groups.

As a key immunosuppressive cytokine maintaining immune homeostasis, inhibition of the TGF-β signaling pathway can relieve immune suppression and remodel an immune-activated tumor microenvironment (TME) (Supplementary Fig. 23)^40,41^. To verify the immune-activating effect of GS@DFP, the immunomodulatory capacity was assessed in the GL261-Luci tumor-bearing mouse model (Fig. 9a and Supplementary Fig. 24a). After treatment, both GS@FP and GS@DFP markedly suppressed the expression of pSMAD2/3 and TSP-1 in GBM, indicating significant inhibition of downstream TGF-β signaling (Fig. 8g). Correspondingly, the secretion of immunosuppressive cytokine TGF-β was markedly reduced, while the immune-activating cytokine IFN-γ was significantly upregulated, suggesting a reprogramming of the tumor immune microenvironment (Fig. 9b). Interestingly, TMZ treatment resulted in decreased IFN-γ expression, revealing the immunosuppressive effect of chemotherapy.

In GBM, TGF-β inhibits the maturation and antigen-presenting function of dendritic cells (DCs); thus, reversing this inhibition can restore DC maturation^42^. After GS@FP or GS@DFP treatment, the proportion of mature DCs in cervical lymph nodes was significantly increased (Fig. 9c, f, and Supplementary Fig. 24b). In both tumor and spleen tissues, the activity of cytotoxic CD8+ T lymphocytes (Fig. 9g and Supplementary Fig. 25) and Natural Killer (NK) cells (Fig. 9h and Supplementary Fig. 26)—previously suppressed by TGF-β—was notably enhanced^43,44^. Meanwhile, the recruitment and expansion of myeloid-derived suppressor cells (MDSCs)^45^, driven by TGF-β, were markedly attenuated after treatment with GS@FP and GS@DFP (Fig. 9i, m, and Supplementary Fig. 27). The TGF-β-driven polarization of macrophages toward the M2 phenotype (Fig. 9d, j, n, and Supplementary Fig. 28) and differentiation of naive T cells into Regulatory T (Treg) cells (Fig. 9e, k, o, and Supplementary Fig. 29) were both reversed^46^. The significant reduction in FOXP3 and CD206 expression further confirmed the decrease of these two immunosuppressive cell populations (Fig. 9l). Collectively, these results demonstrate that GS@DFP effectively activates the tumor immune microenvironment, reverses immunosuppression, and enhances immune cytotoxicity.

## Discussion

This study highlights the significant therapeutic potential of GS@DFP in enhancing GBM treatment by disrupting the supportive role of healthy niche cells within the brain microenvironment. In contrast to conventional therapies, GS@DFP specifically targets and interrupts the malignant interactions between normal astrocytes and tumor cells, thereby suppressing tumor proliferation and reducing network-like tumor characteristics. The glucose-analogue-based delivery system, combined with dual ROS-responsive functional modifications, enables efficient penetration of the BBB and precise intratumoral delivery.

Targeting the mitochondrial transfer from astrocytes to tumor cells represents a major innovation of this study and offers advantages over traditional GBM treatment strategies. Although chemotherapy and surgical resection remain standard options, they are often limited by suboptimal therapeutic outcomes, persistent immunosuppression, indistinct tumor boundaries, and rapid recurrence. The involvement of diverse cell populations within the TME contributes to these challenges, underscoring the notion that cancer treatment is never a single-target event. A comprehensive understanding of the complex and dynamic tumor microenvironment is essential for improving clinical outcomes.

The ability of GS@DFP to simultaneously target mitochondria transferred from astrocytes and the hyperactive tumor mitochondria driven by GBM’s high metabolic demands is central to its therapeutic efficacy. GBM, as one of the most aggressive brain tumors, exhibits strong network-forming behavior and can co-opt surrounding neuronal and astrocytic ecosystems, including the highly malignant process of mitochondrial transfer. Galunisertib attenuates astrocyte-derived support by inhibiting tumor microtubules and disrupts the TGF-β-driven immunosuppressive core of the TME. In addition to TGF-β pathway inhibition, mitochondrial disruption and ROS generation also contribute to immune remodeling, acting in parallel to reinforce the antitumor immune response. Meanwhile, although intense metabolic activity promotes tumor evolution, it also exposes metabolic vulnerabilities. As a selective ATP synthase inhibitor, S-Gboxin readily disrupts tumor mitochondria and demonstrates substantially greater cytotoxicity than TMZ. Although mitochondrial function is essential for immune cells, the tumor-targeted and ROS-responsive nature of GS@DFP restricts this effect predominantly to tumor regions, thereby preserving overall immune competence. The ROS-responsive polymer nanoparticles modified with a glucose analogue enable efficient co-delivery of both therapeutics. After crossing the BBB, GS@DFP releases its payload in response to elevated ROS levels, achieving precise targeting of both tumor mitochondria and mitochondria acquired via intercellular transfer.

Beyond efficient delivery, GS@DFP exhibits potent oxidative stress-inducing capacity. Through ferrocene-mediated Fenton-like catalysis, it converts endogenous H_2_O_2_ into more reactive species, thereby disrupting the intracellular redox balance in a concentration-dependent manner. Meanwhile, GS@DFP demonstrates stability and high ROS responsiveness, offering advantages in complex biological environments. Both in vitro and in vivo studies confirm that this nanoformulation downregulates mitochondrial transfer, inhibits malignant proliferation, and damages tumor-intrinsic mitochondria, collectively contributing to robust antitumor efficacy. Safety evaluations further reveal that GS@DFP induces no systemic toxicity or adverse effects, supporting its favorable safety profile and broad therapeutic potential.

Despite these encouraging results, certain limitations remain. Mouse GBM models do not fully recapitulate human primary grade IV GBM, necessitating further investigations, including clinical studies, to validate its therapeutic efficacy in patients. Additionally, although GS@DFP demonstrates strong mitochondrial-disrupting and mitochondrial-transfer-inhibiting capabilities, the molecular mechanisms governing its BBB penetration and in vivo biodistribution require deeper exploration. Finally, as another brain cell type tamed by tumors, neurons also exhibit strong pro-tumorigenic effects during glioma initiation and progression. Therefore, deciphering and even disrupting this malignant crosstalk likewise holds substantial exploratory and therapeutic potential.

In conclusion, GS@DFP offers a promising therapeutic strategy for GBM, with clear advantages in tumor suppression, targeting metabolic vulnerabilities, and ensuring safety. Its ability to effectively target GBM and disrupt malignant intercellular interactions provides an integrated approach to addressing the key pathological drivers of GBM progression.

## Methods

### Ethical statement

All animal experiments were performed in accordance with the institutional guidelines and approved by the Institutional Animal Care and Use Committee of Fudan University (2022-07-YJ-JC-86). Body weight loss during the study was not allowed to exceed 20%. Primary glioblastoma and peritumoral tissues from patients were obtained from Shanghai General Hospital (Shanghai, China). The study was approved by the Ethics Committee of Shanghai General Hospital (Approval No. 2019S211). Written informed consent was obtained from all patients for the use of their samples for research and publication purposes.

### Materials

CH₃O–PEG₅₀₀₀–NH₂ and N₃–PEG₅₀₀₀–NH₂ were purchased from Seebio. N⁶-benzyloxycarbonyl-L-lysine, hydrobromic acid, trifluoroacetic acid (TFA), disodium ethylenediaminetetraacetate (EDTA), N,N,N′,N′,N′′-pentamethyldiethylenetriamine (PMDETA), cuprous iodide (CuI), and L(+)-ascorbic acid were obtained from Adamas. 4-hydroxybenzyl alcohol (HBA), 4-(hydroxymethyl)phenylboronic acid, S-Gboxin, and N,N′-carbonyldiimidazole (CDI) were purchased from YuanYe Bio-Tech. Triphosgene was purchased from TCI, while ferrocenecarboxylic acid and galunisertib were obtained from MACKLIN. 1-(3-dimethylaminopropyl)−3-ethylcarbodiimide hydrochloride (EDCI), 4-dimethylaminopyridine (DMAP), and 1-hydroxybenzotriazole (HOBT) were purchased from Energy Chemical. All other solvents were purchased from Adamas. DMEM, DMEM/F12, 0.25% trypsin-EDTA, poly-D-lysine (PDL), and fetal bovine serum (FBS) were obtained from Gibco, and penicillin–streptomycin solution and D-luciferin was purchased from Meilun. N₂ supplement was obtained from Beyotime. FGF-2, EGF, and FITC-dextran were purchased from Yeasen Biotechnology. FITC, TAMRA, coumarin-6, and DiD perchlorate were purchased from Meilun. DHE, JC-1, PI, Mito-Tracker Red, MitoSOX, Lyso-Tracker Red, Lipo6000/Lipo8000 Transfection Reagent were obtained from Beyotime, and SR101, Mito-Tracker Green was purchased from Thermo Fisher.

### Instruments

Nuclear magnetic resonance (NMR) spectrometer (Mercury Plus 400 MHz, Varian, USA); nanoparticle size and zeta potential analyzer (Zetasizer Nano ZS, Malvern, UK); laser confocal live-cell imaging system (LSM 980, Zeiss, Germany); inverted biological microscope (37XB, Shanghai Optical Instrument Factory No. 6, China); whole-slide scanner (VS120, Olympus, Japan); inverted fluorescence microscope (DMI4000D, Leica, Germany); flow cytometer (CytoFLEX S, Beckman Coulter, USA); small-animal in vivo multimodal imaging system (IVIS Spectrum CT, PerkinElmer, USA); and small-animal in vivo two-photon confocal microscope (IVM-CMS Series III, Revvity, China) were used in this study.

### Cell culture and animals

G422 cells  were purchased from TongPai Biotechnology (Shanghai, China), and GL261 cells  were obtained from GaiNing Biological (Shanghai, China). GL261 cells stably expressing Zs-green protein were established by Genomeditech (Shanghai, China). bEnd.3 cells  were purchased from Procell Life Science & Technology (Wuhan, China). Cell identity was verified by the suppliers prior to distribution. All cell lines were routinely tested for mycoplasma contamination using PCR-based assays and were confirmed to be mycoplasma-free before use. G422 and bEnd.3 cells were maintained in DMEM supplemented with 10% FBS and 1% penicillin–streptomycin. GL261 cells were cultured under the same conditions. Astrocytes were maintained in complete DMEM/F12 medium containing 10 ng mL^−1^ fibroblast growth factor-2 (FGF-2), 10 ng mL^−^^1^ epidermal growth factor (EGF), N_2_ supplement, 10% FBS, and 1% penicillin–streptomycin. All cells were incubated at 37 °C in a humidified atmosphere containing 5% CO_2_.

C57BL/6 mice (male, 18–20 g, 6–8 weeks old) and neonatal C57BL/6 pups (within 24 h of birth) were obtained from SIPPR/BK Experimental Animal Co., Ltd. (Shanghai, China). Mice were housed under constant environmental conditions (temperature, 21 ± 1 °C; relative humidity, 40–70%; 12 h light/dark cycle) with free access to food and water. Orthotopic GBM models were established by injecting GL261 or Zs-green GL261 cells (2 × 10^5^ cells per mouse) into the brains of male C57BL/6J mice using a stereotaxic apparatus. For mice bearing orthotopic GBM, euthanasia was performed when animals exhibited neurological symptoms, such as marked ataxia, hunchback posture, or mild hemiplegia in addition to body weight loss.

### Isolation of primary astrocytes

Primary astrocytes were isolated from the cortical tissue of C57 BL/6 neonatal mice. In brief, neonatal mice were euthanized by cervical dislocation, and brains were aseptically removed and placed in pre-chilled HBSS. Meninges, hippocampus, and striatum were carefully removed using ophthalmic forceps, and the remaining cortical tissue was cut into small pieces and digested with 0.125% trypsin for 15 min. Digestion was terminated by adding complete culture medium, and the tissue was washed thoroughly. Cells were then resuspended using a pipette, filtered through a cell strainer, and transferred to T25 culture flasks pre-coated with poly-D-lysine (PDL). When astrocytes reached approximately 90% confluence, flasks were shaken overnight to remove microglia. The adherent cells were considered primary astrocytes and used for subsequent experiments.

### Synthesis of PEG-poly(amino acid) backbone

Lys(Z)-NCA was synthesized following a previously reported method. In brief, 1 equivalent of N6-benzoxycarbonyl-L-lysine was mixed with 0.43 equivalent of triphosgene and reacted in anhydrous THF at 50 °C for 3 h under nitrogen atmosphere. The reaction mixture was concentrated, recrystallized from n-hexane, and filtered under vacuum to obtain Lys(Z)-NCA. The PEG–poly(amino acid) polymer was prepared via ring-opening polymerization (ROP). CH_3_O-PEG_5000_-NH_2_ or N_3_-PEG_5000_-NH_2_ was used as an initiator and mixed with Lys(Z)-NCA at a molar ratio of 1:30 or 1:20 in a two-neck flask. The reaction was carried out in anhydrous DMF at 50 °C for 48 h under nitrogen. After completion, DMF was removed by rotary evaporation, and the protecting groups were removed by reaction with 33% w/v HBr/acetic acid at room temperature for 4 h. The resulting polymer was dialyzed in water using a 3500 Da molecular weight cutoff dialysis bag for 24 h, then lyophilized and stored at −20 °C.

The synthesis of Lys(Z)-NCA and the PEG-poly(amino acid) backbone was further confirmed by ^1^H NMR analysis.

### Synthesis of Pb polymer

The boronic ester active precursor was synthesized following a previously reported method. Briefly, 4-(hydroxymethyl)phenylboronic acid and CDI were mixed at a 1:2 molar ratio and reacted in anhydrous DCM at room temperature for 30 min under nitrogen. DCM was removed by rotary evaporation, and the residue was redissolved in ethyl acetate, washed with water, dried, and concentrated to obtain the boronic ester active precursor. The precursor was then conjugated to the polymer backbone via a substitution reaction. In a two-neck flask, 300 mg of CH_3_O-PEG-pLy_30_, 313 mg of boronic ester active precursor, 117 mg of DMAP, and 133 μL of triethylamine were mixed and reacted in anhydrous DMF at room temperature for 48 h under nitrogen. The reaction mixture was dialyzed in DMF for 12 h and then in water for 24 h using a 3500 Da molecular weight cutoff dialysis bag. The resulting product was lyophilized and stored at −20 °C.

The synthesis of the boronic ester active precursor and Pb polymer was further confirmed by ^1^H NMR analysis.

### Synthesis of Fc polymer/DHA-modified Fc polymer

Fc-COOH was conjugated to the polymer backbone via a classical amide coupling reaction. Briefly, 158 mg of Fc-COOH, 132 mg of EDCI, and 93 mg of HOBt were reacted in DMF at room temperature for 1 h under nitrogen. Subsequently, 248 mg of CH_3_O-PEG-pLy_20_ or N_3_-PEG-pLy_20_ was mixed with 240 μL DIPEA in 20 mL DMF and added to the above solution. After 24 h reaction, the mixture was dialyzed in methanol for 48 h and then in water for 24 h using a 3500 Da molecular weight cutoff dialysis bag. The product was lyophilized and stored at −20 °C. DHAA was synthesized following a previously reported method. Briefly, 0.24 g KOH was dissolved in 15 mL H₂O under nitrogen, stirred, and cooled. Then 0.75 g L-(+)-ascorbic acid was added and stirred at room temperature for 1 h. Next, 1 g allyl bromide dissolved in 30 mL acetone was added. The reaction was carried out at 40 °C for 48 h. After completion, acetone was removed by rotary evaporation, and the residue was extracted with ethyl acetate, washed, dried, and concentrated by rotary evaporation to yield 0.56 g DHAA. DHA modification of Fc polymer was performed via copper-catalyzed azide-alkyne cycloaddition. Briefly, 50 mg of N3-terminated Fc polymer, 2 mg DHAA, 1.8 mg CuI, 8  mg L-(+)-ascorbic acid, and 1 mg PMDETA were dissolved in 30 mL anhydrous DMF and reacted at room temperature in the dark under nitrogen for 12 h. The reaction mixture was dialyzed in 10 mmol/L EDTA aqueous solution for 12 h, followed by deionized water for 24 h, and finally lyophilized to obtain DHA-modified Fc polymer.

The synthesis of Fc polymer and DHA-modified Fc polymer were further confirmed by ^1^H NMR analysis.

### Formulation characterization

For transmission electron microscopy (TEM) sample preparation, 10 µL of freshly prepared GS@DFP or GS@DFP after drug release under different conditions was dropped onto a carbon-supported copper grid and left at room temperature for 1 min. Excess sample was removed from the edges of the grid using filter paper. Then, 10 µL of 3% uranyl acetate staining solution was added to the dish lid, and the grid was floated on the staining solution for 1 min at room temperature. After removing excess stain with filter paper, the grid was rinsed twice with deionized water. After air-drying at room temperature, the samples were imaged using a transmission electron microscope (TEM). For DLS measurement, the hydrodynamic size of different formulations was measured in water at 37 °C.

### FRET effect

Water-soluble fluorescent probes TAMRA and FITC, with a spectral overlap greater than 30%, were selected as model probes. The probes were covalently conjugated to polymer backbones via their NHS groups, reacting with amino groups on the polymers, with TAMRA linked to the Fc polymer and FITC linked to the Pb polymer. The reaction was carried out at room temperature for 24 h. After completion, the reaction mixture was dialyzed in deionized water using a dialysis bag with a molecular weight cutoff of 3500 Da for 72 h to remove unreacted probes. The dialyzed products were then lyophilized and stored at −20 °C. Pb-FITC/Fc-TAMRA nanoparticles were prepared using the nanoprecipitation method. The emission spectra of the nanoparticles, before and after treatment with 100 μM H_2_O_2_, were recorded using a microplate reader, with excitation at 488 nm and emission scanned from 520 to 650 nm.

### Culture and treatment of GBM organoids

The culture medium for GBM organoids was refreshed every 3–4 days. After approximately 2 weeks of culture, the GBM organoids were randomly divided into three groups for treatments.

### Western blot analysis

For cell samples, after respective treatments, cells were lysed on ice for 10 min using RIPA lysis buffer containing PMSF. For tissue samples, 800 µL of RIPA buffer containing PMSF was added per 100 µg of tissue. When detecting phosphorylated proteins, phosphatase inhibitors were added. The lysates were centrifuged at 13,000 × *g* and 4 °C for 10 min, and the supernatants were collected. Protein concentrations were determined using a BCA Protein Assay Kit. Samples were then mixed with 5× loading buffer and heated at 95 °C for 5 min. Equal amounts of protein (20 µg per well) were separated by SDS-PAGE for 2 h and subsequently transferred onto polyvinylidene fluoride (PVDF) membranes. After blocking with rapid blocking solution at room temperature for 20 min, membranes were incubated with primary antibodies overnight at 4 °C. Membranes were washed three times with Tris-buffered saline containing 0.1% Tween-20 (TBST), followed by incubation with HRP-conjugated secondary antibodies at room temperature for 1.5 h. After three additional washes with TBST, protein bands were visualized using enhanced chemiluminescence (ECL) detection on a gel imaging system. Antibodies used: pSMAD2/3 (Beyotime, Cat. AF5920, 1:1000 for WB); TSP-1 (ABclonal, Cat.  A2125, 1:500 for WB); Caspase-3 (ABclonal, Cat. A17900, Clone: AMC0214, 1:1000 for WB); Cleaved Caspase-3 (Cell Signaling Technology, Cat. 9664, Clone: 5A1E, 1:50 for WB); HRP-conjugated GAPDH (ABclonal, Cat. AC035, Clone: AMC0062R, 1:20,000 for WB); HRP-labeled Goat Anti-Rabbit IgG (Beyotime, Cat. A0208, 1:1000 for WB).

### Immunofluorescence analysis

For cell samples, cells in confocal dishes were fixed with 4% paraformaldehyde for 15 min, washed three times with PBS, and permeabilized with 0.1% Triton® X-100 for 15 min. After another PBS wash, cells were blocked with immunofluorescence blocking solution for 30 min. Primary antibodies were added and incubated overnight, followed by washing and incubation with secondary antibodies at room temperature for 1.5 h. Cells were then stained with DAPI for 10 min, washed, and imaged using a fluorescence microscope.

For tissue sections, mice were euthanized, and the brain was perfused with 4% paraformaldehyde and further fixed in 4% paraformaldehyde for 48 h. The tissue was dehydrated, embedded in paraffin, deparaffinized with xylene, and rehydrated through a graded ethanol series. Antigen retrieval was performed in antigen retrieval solution for 30 min, followed by washing with deionized water and blocking with immunofluorescence blocking solution for 30 min. Primary antibodies were incubated overnight, followed by washing and incubation with secondary antibodies at room temperature for 1.5 h. Sections were counterstained with DAPI and mounted with anti-fade mounting medium for fluorescence imaging.

Primary antibodies: HSP60 (ABclonal, Cat. A0564, Clone: ARC0260, 1:500 for IF); GFAP (Beyotime, Cat. AF0156, 1:100 for IF); Nestin (Beyotime, Cat. AF2215, 1:100 for IF); GAP43 (ABclonal, Cat. A19055, Clone: ARC0246, 1:500 for IF); Ki67 (Beyotime, Cat. C2301S, 1:1 for IF)

Secondary antibodies: Donkey Anti-Mouse IgG-Alexa Fluor 488 (Abcam, Cat. ab150109, 1:500 for IF); Donkey Anti-Rabbit IgG-Alexa Fluor 555 (Abcam, Cat. ab150062, 1:500 for IF); Goat Anti-Rabbit IgG-Alexa Fluor 647 (Abcam, Cat. ab150079, 1:500 for IF);

### Cytotoxicity assay

Cell viability under different treatments was assessed using the Cell Counting Kit-8 (CCK-8) assay. GL261 cells (8 × 10^3^ cells/well) were seeded in 96-well plates. When the cells reached approximately 70% confluency, 200 µL of drugs or nanoparticles at different concentrations were added and incubated with the cells for 24 h. After treatment, cells were washed with PBS, and 100 µL of CCK-8 working solution was added for an additional 2 h incubation. Absorbance at 450 nm was measured using a microplate reader, and cell viability and IC_50_ values were calculated for each group.

### Preparation of GS@FP/GS@DFP

Pb polymer, Fc polymer, and DHA-modified Fc polymer were each dissolved in DMF at 50 mg/mL. For GS@FP, Pb and Fc polymers were mixed at a 1:2 mass ratio. A 300 μL aliquot of this polymer mixture was combined with 3 mg S-Gboxin and 5 mg galunisertib, then added dropwise into 2.7 mL PBS at 25 μL/min under vigorous stirring to form drug-loaded nanoparticles via nanoprecipitation. The resulting nanoparticles were dialyzed against PBS (MWCO 3500 Da) for 12 h to remove free drugs and subsequently filtered through a 0.22 μm membrane. GS@DFP was prepared in a similar manner, using a Pb:Fc:DHA-Fc ratio of 5:7:3 for the polymer mixture, with all other steps identical to those for GS@FP.

### Preparation of Cou-6@FP/Cou-6@DFP and DID@FP/DID@DFP

The polymer mixture was prepared as described above. Then, 200 μL of the polymer solution was mixed with 150 μg of Cou-6 or DID and subjected to nanoprecipitation in PBS. Probe-loaded nanoparticles were dialyzed (MWCO 3500 Da, 12 h) and filtered through a 0.22 μm membrane before use.

### Functional characterization of nanoparticles

Intracellular oxidative stress assay: GL261 cells were treated with DFP, GS@FP, or GS@DFP prepared at different ratios at a final material concentration of 200 μg/mL for 24 h. Cells were then incubated with the oxidative stress probes DCFH-DA or DHE for 30 min, washed three times with PBS, and intracellular fluorescence was quantified by flow cytometry.

Cellular uptake study: GL261 cells were pretreated with various endocytosis inhibitors or incubated at low temperature, followed by incubation with Cou-6@DFP for 30 min. After three washes with PBS, intracellular fluorescence was quantified by flow cytometry.

Lysosomal escape assay: GL261 cells were cultured in four-chamber confocal dishes and incubated with Cou-6@DFP for 0.5 h, 2 h, and 4 h. Lyso-Tracker Red probe was then co-incubated with cells for 15 min, followed by two washes with PBS. Cells were further stained with Hoechst for 10 min, washed twice with PBS, and representative fluorescence images were obtained using a confocal microscope.

In vitro BBB translocation assay: Transwell inserts (3 μm pore size, 0.33 cm^2^ surface area) were pre-coated with Matrigel. bEnd.3 cells (1 × 10^5^ cells/insert) were seeded on the inserts. The medium was replaced every 2 days. FITC-dextran was added to the upper chamber, and the concentration in the lower chamber was measured after 1 h. The apparent permeability coefficient (Papp, cm s^−1^) was calculated using the formula: Papp = (dQ/dt)/(C_0_ × S), where dQ/dt represents the permeation rate (µg s^−1^), C_0_ is the initial nanoparticle concentration (µg mL^−1^), and S is the effective surface area of the insert (0.33 cm^2^). For nanoparticle translocation studies, DID@FP or DID@DFP was added to the upper chamber, and DID content in the lower chamber was measured at 10, 15, 30, and 60 min to construct cumulative translocation profiles.

### Assessment of mitochondrial damage

GL261 cells (2 × 10^5^ cells/well) were seeded in six-well plates. At ~80% confluence, cells were treated with equivalent doses of S-Gboxin (6 μg/mL) and galunisertib (10 μg/mL) for 6 h. Mitochondrial membrane potential (ΔΨm) was analyzed by incubating cells with JC-1 (10 μg/mL, 20 min), followed by three PBS washes and flow cytometry. Cellular ATP levels were measured from lysates using an ATP assay kit after centrifugation at 13,000 × *g*. For Bio-TEM, cells were fixed with 4% paraformaldehyde, detached with a cell scraper, centrifuged at 300 × *g*, and processed for imaging. Apoptosis-related proteins were analyzed by Western blot after 24 h treatment (Caspase-3 and cleaved Caspase-3, 1:50; HRP-conjugated secondary antibody, 1:2000).

For confocal microscopy, GL261 cells (1 × 10^4^ cells/well) were seeded in four-chamber dishes at ~50% confluence. To observe mitochondrial morphology, cells were treated with equivalent lower doses of S-Gboxin (3 μg/mL) and galunisertib (5 μg/mL) for 6 h and stained with Mito-Tracker (20 nM, 20 min).

### In vitro tumor microtube (MT) inhibition

To evaluate MT inhibition, GL261 cells (2 × 10^5^ cells/well) were seeded in six-well plates. At ~50% confluence, cells were treated with equivalent doses of S-Gboxin (4.5 μg/mL) and galunisertib (7.5 μg/mL) for 24 h. MT-regulating proteins were analyzed by Western blot using primary antibodies against pSMAD2/3 (1:1000, Beyotime) and TSP-1 (1:500, Abclonal), followed by HRP-conjugated secondary antibody (1:2000, Beyotime).

For direct MT visualization, GL261 cells (1 × 10^4^ cells/well) were seeded in four-chamber confocal dishes and treated under the same conditions for 24 h. Cells were stained with 100 nM phalloidin for immunofluorescence imaging to observe MT structures.

To assess MT inhibition effects on cell migration and invasion, wound healing and transwell assays were performed. For wound healing, GL261 cells (2 × 10^5^ cells/well) were seeded in six-well plates, treated with equivalent doses of S-Gboxin (4.5 μg/mL) and galunisertib (7.5 μg/mL) for 24 h, and a scratch was created. Wound closure was imaged using an inverted microscope and quantified using ImageJ. For invasion assays, treated GL261 cells were collected after 24 h and seeded into transwell inserts. DMEM containing 20% FBS was added to the lower chamber. After 12 h, migrated cells were fixed with 4% PFA, stained with 0.5% crystal violet, imaged under an inverted microscope, and quantified using ImageJ.

### In vitro downregulation of mitochondrial transfer and tumorigenicity

For mitochondrial transfer experiments, astrocytes (3 × 10^5^ cells/well) were seeded in six-well plates and cultured for 2 days. ZS-green GL261 cells (2 × 10^5^ cells/well) were seeded separately in six-well plates. At ~50% confluence, tumor cells were treated with equivalent doses of S-Gboxin (4.5 μg/mL) and galunisertib (7.5 μg/mL) for 24 h, then collected for subsequent experiments.

For MT observation in co-culture systems, treated cancer cells (1.5 × 10^5^ cells/well) were added to astrocytes and co-cultured for 24 h before imaging.

For mitochondrial transfer assays, astrocytes (3 × 10^5^ cells/well) were pre-seeded in six-well plates and transfected with Mito-mCherry (BioScience) using Lipo8000 or Lipo6000 to label mitochondria with red fluorescence. Treated cancer cells (same density) were then co-cultured with astrocytes for 24 h, including a Transwell co-culture group. After 24 h, cells were collected for flow cytometric detection of double-positive signals, representing the fraction of tumor cells receiving mitochondria. Astrocytes were also stained with 20 nM Mito-Tracker Red, washed three times to remove free dye. Cancer cells were labeled with CFSE before co-culture; subsequent procedures were identical.

For quantification of mitochondria in tumor cells, treated cancer cells were co-cultured with astrocytes for 24 h. The entire co-culture system was stained with 20 nM Mito-Tracker Red, and flow cytometry was used to distinguish tumor cells and quantify mitochondrial content.

For cell cycle analysis, treated cancer cells were co-cultured with astrocytes for 24 h. Cells were stained with PI for 20 min, washed three times, and analyzed by flow cytometry. Tumor cell populations were gated, and cell cycle distribution was fitted and quantified using FlowJo.

### In vivo downregulation of mitochondrial transfer

Five days after orthotopic transplantation of ZS-green GL261 cells, mice were administered PBS, Gal@DFP (galunisertib-loaded nanoparticles), S-G@DFP (S-Gboxin-loaded nanoparticles), or GS@DFP (dual-drug-loaded nanoparticles) via tail vein injection (equivalent doses: S-Gboxin, 3 mg/kg; galunisertib, 5 mg/kg) every 2 days for a total of 5 doses.

For assessment of astrocyte-to-tumor mitochondrial transfer and tumor mitochondrial content, in vivo imaging and in vitro analyses were performed. For in vivo imaging, SR101 was intraperitoneally injected at 0.12 mg/g 10 h prior to two-photon microscopy in mice with cranial windows, to observe colocalization of green tumor signals and red SR101 fluorescence. For flow cytometric analysis, mice were perfused with saline, tumor regions were dissected, dissociated, and filtered to obtain single-cell suspensions. For SR101 uptake conditions, the fluorescence intensity of SR101 in gated cancer cell populations was measured by flow cytometry. For tumor mitochondrial content, cells were stained with 20 nM Mito-Tracker for 20 min, centrifuged, washed, and resuspended. Mitochondrial fluorescence in gated cancer cell populations was quantified by flow cytometry.

For histological evaluation, tumor sections were immunostained with mouse anti-GFAP (1:100, Beyotime), rabbit anti-Nestin (1:100, Beyotime), and rabbit anti-HSP60 (1:100, Beyotime). Proliferative cells were detected with a Ki67 Cell Proliferation Detection Kit (Beyotime), and apoptotic cells were assessed with a TUNEL Apoptosis Detection Kit (Beyotime).

### In vivo antitumor and immune-modulatory effects

Five days after orthotopic transplantation of GL261 cells, mice were administered PBS, free galunisertib and S-Gboxin, GS@FP, or GS@DFP via tail vein injection (equivalent doses: S-Gboxin, 3 mg/kg; galunisertib, 5 mg/kg), and simultaneously received oral TMZ (5 mg/kg).

For evaluation of antitumor effects, treatments were given every 2 days for a total of 5 doses. Tumor bioluminescence in mice was monitored and quantified using IVIS following intraperitoneal injection of D-Luciferin (15 mg kg^−1^). Mouse survival and body weight were recorded. At the end of the treatment, blood was collected from the heart, centrifuged at 500 × *g* for 10 min at 4 °C to obtain plasma. Plasma levels of alanine aminotransferase (ALT), aspartate aminotransferase (AST), blood urea nitrogen (BUN), and creatinine (CREA) were measured to assess biosafety. Major organs were fixed in 4% PFA, sectioned, and stained with H&E for histological evaluation of safety.

For immune microenvironment analysis, treatments were given every 2 days for a total of 3 doses. After the final treatment, mice were perfused with saline, and tumor-bearing brains were collected for Western blot or ELISA analysis of cytokine levels. For flow cytometry, tumor regions were dissected, gently dissociated on a 70 μm cell strainer, and brain immune cells were isolated using Percoll. Cervical lymph nodes were similarly processed to obtain dendritic cells (DCs), and spleens were dissociated to obtain peripheral immune cells.

For bone marrow cell analysis, cells were stained with PerCP-Cy5.5-anti-mouse CD11c, APC-anti-mouse CD80, PE-anti-mouse CD86, APC-anti-mouse CD45, AF488-anti-mouse CD11b, AF450-anti-mouse F4/80, PE-Cy7-anti-mouse CD206, AF780-anti-mouse CD11b, APC-anti-mouse CD45, and FITC-anti-mouse Gr-1. For lymphocyte analysis, cells were stained with APC-anti-mouse CD45, FITC-anti-mouse CD3, PerCP-Cy5.5-anti-mouse CD4, PE-anti-mouse CD8a, FITC-anti-mouse CD25, AF450-anti-mouse Foxp3, FITC-anti-mouse CD3, APC-anti-mouse NK1.1, and PE-anti-mouse CD62L. Stained cells were analyzed using a CytoFLEX S flow cytometer.

### Gene expression level and correlation analysis

Gene expression and correlation analyses were performed using GEPIA and Sangerbox 3.0 (http://sangerbox.com/home.html), based on data from the TCGA database and the NCBI dataset (GSE263588). This analysis aimed to determine differences in gene expression levels between tumor and normal tissues, correlations among genes, and associations between gene expression and patients’ OS.

### Schematics

Schematics were created with 3ds Max, Adobe Illustrator and BioRender.com (Created in BioRender. Jiang, C. (2025) https://BioRender.com/8x6yk6f).

### Statistical analysis and reproducibility

Data are presented as mean ± SD. GraphPad Prism 10, FlowJo (v10.8.1), and ImageJ were used for data processing and statistical analyses. One-way ANOVA with Tukey’s multiple comparisons test was used to compare multiple groups, and a two-tailed unpaired Student’s *t* test was used for comparisons between two groups. Kaplan–Meier survival curves were analyzed using the log-rank test to assess differences in survival between groups. *p* > 0.05 represents non-significance, while *p* < 0.05 represents statistical significance. No statistical method was used to predetermine sample size. No data were excluded from the analyses.

### Reporting summary

Further information on research design is available in the Nature Portfolio Reporting Summary linked to this article.

## Supplementary information

Supplementary Information

Reporting Summary

Transparent Peer Review File

## Source data

Source Data

## Data Availability

All data generated or analyzed during this study are available within the Article, its Supplementary Information file and the Source data file. Source data are provided with this paper, including the raw numerical data underlying all graphs and the raw NMR data supporting structural characterization. The NMR characterization data are provided in the “NMR raw data” worksheet of the Source data file. Source data are provided with this paper.
