# Supplementary material for: ROS-autocatalytic nanoplatform interfering astrocyte-cancer crosstalk and enhancing glioblastoma therapy
Source: Nat Commun. 2026 Sep 10;17:10672. doi: 10.1038/s41467-026-77395-2 (PMC13655175; doi:10.1038/s41467-026-77395-2)
Supplement: Supplementary file 1 [file 41467_2026_77395_MOESM1_ESM.pdf]

Supplementary Information file of "ROS-Autocatalytic Nanoplatform Interfering Astrocyte-Cancer Crosstalk and Enhancing Glioblastoma Therapy"

Yun Chen[1], Zonghua Tian[1], Hongrui Fan[1], Jingyi Zhou[1], Shilin Zhang[1], Xuwen Li[1], Boyu Su[1], Chen Jiang[1], Tao Sun[1*]*

[1]Department of Pharmaceutics, School of Pharmaceutical Sciences, Fudan University, Key Laboratory of Smart Drug Delivery, Ministry of Education, State Key Laboratory of Brain Function and Disorders, MOE Frontiers Center for Brain Science, Minhang Hospital, Fudan University, Shanghai, 201203 China

[*]: corresponding authors

E-mail: jiangchen@shmu.edu.cn; sunt@fudan.edu.cn

Supplementary Figures

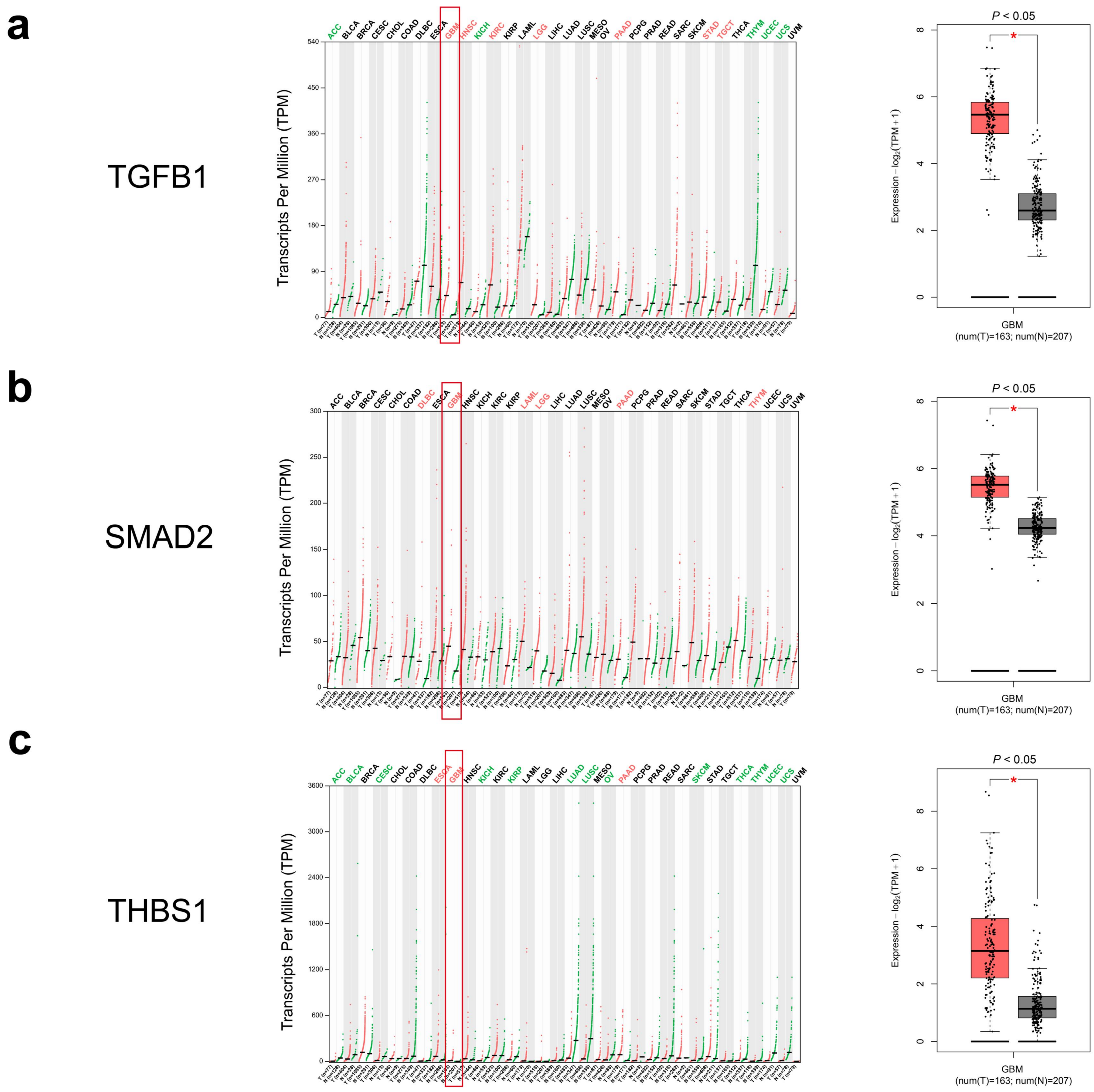

Supplementary Figure 1. Comparison of the expression levels of TGFB1, SMAD2, and THBS1 (TSP-1) between tumor and adjacent normal tissues in GBM patient samples analyzed using GEPIA. Box plot of TGFB1, SMAD2, and THBS1 expression (log_2(TPM+1)) in GBM (red, n=163 patient samples) vs. normal tissues (gray, n=207 patient samples). Boxes represent 25th-75th percentiles, centre lines show medians, and whiskers extend to Tukey's 1.5×IQR limits (minima/maxima of non-outlier data).

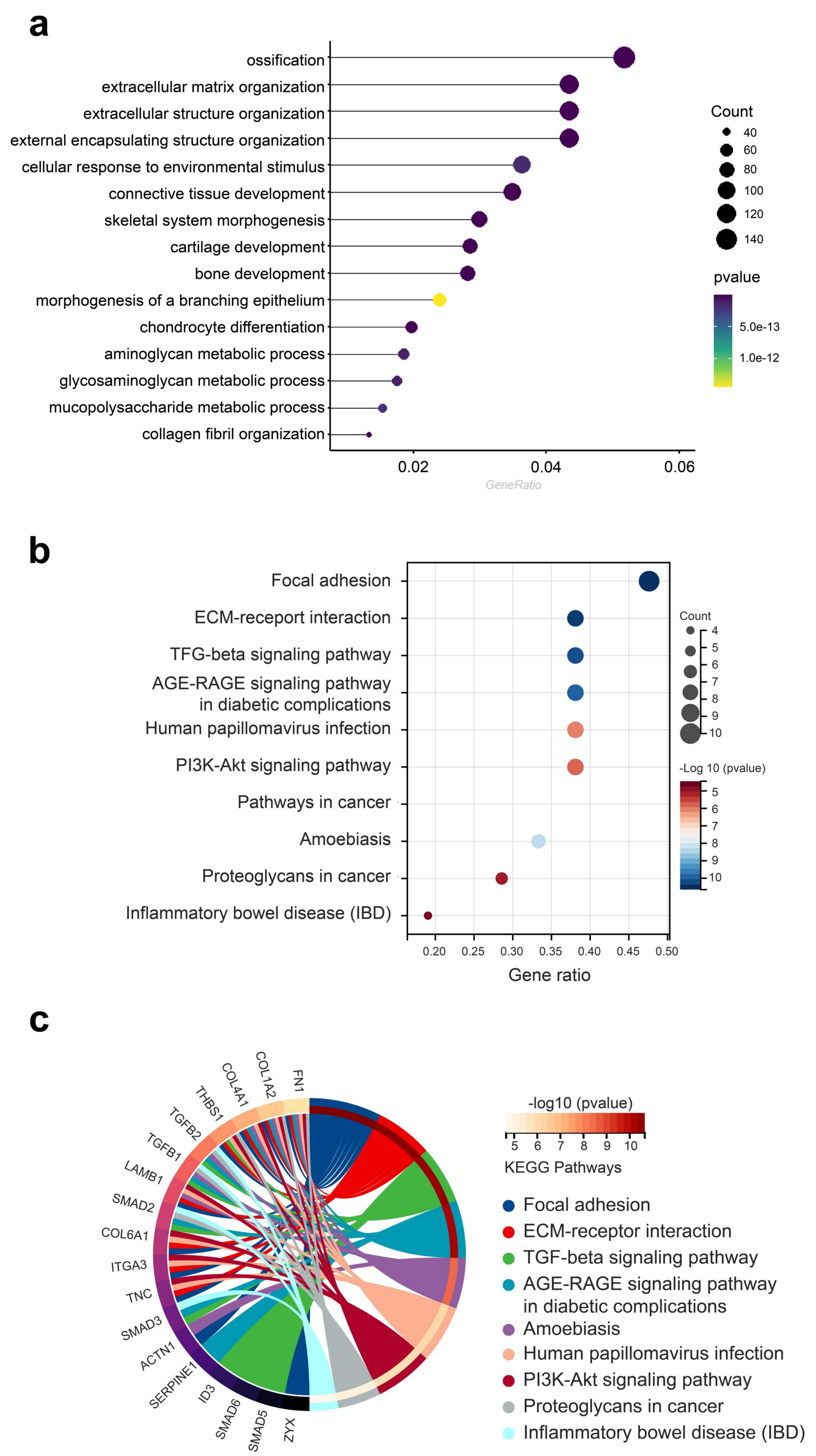

Supplementary Figure 2. (a) Lollipop plot illustrating the signaling pathways associated with all upregulated genes in GBM patient samples. (b) Bubble plot summarizing the enriched KEGG pathways. The x-axis represents the gene ratio, and bubble size indicates the number of enriched genes; (c) Chord diagram showing the relationships between TGF-β high-response-related genes and significantly enriched KEGG pathways.

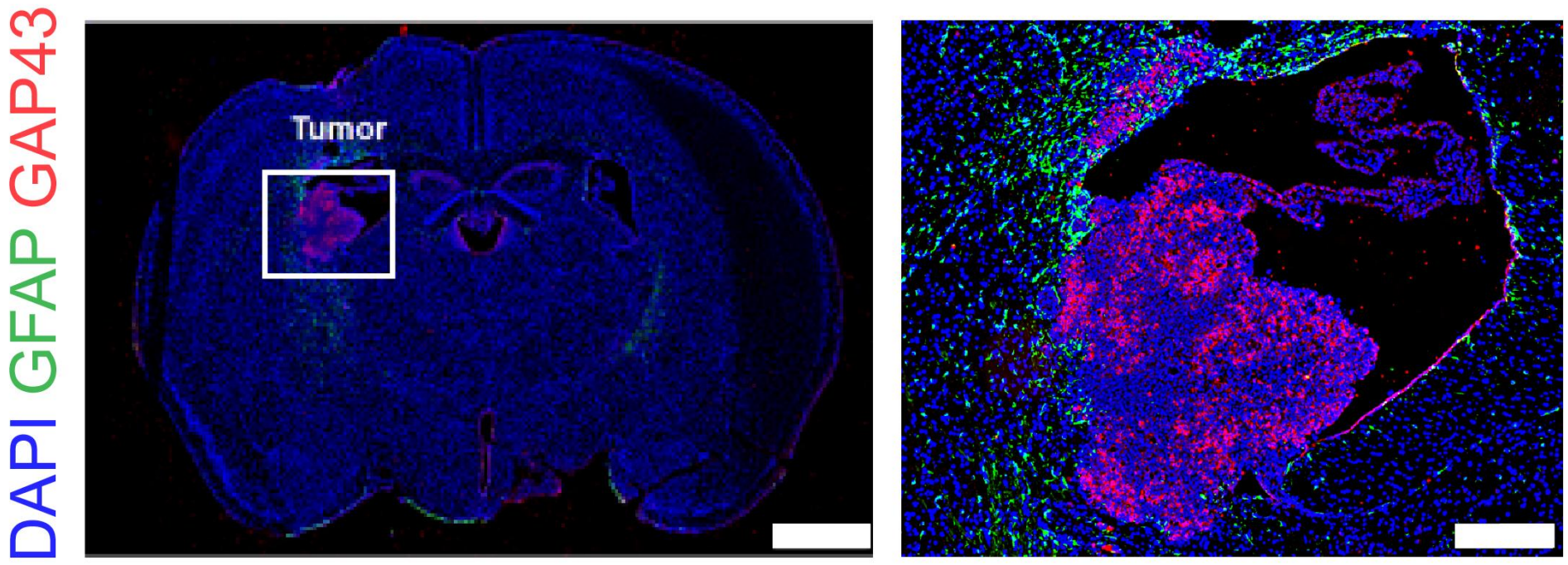

Supplementary Figure 3. Expression of the tumor MT-associated axonal protein GAP43 in tumor tissues of the GL261-bearing mouse model. Scale bar = 500 μm and 100 μm).

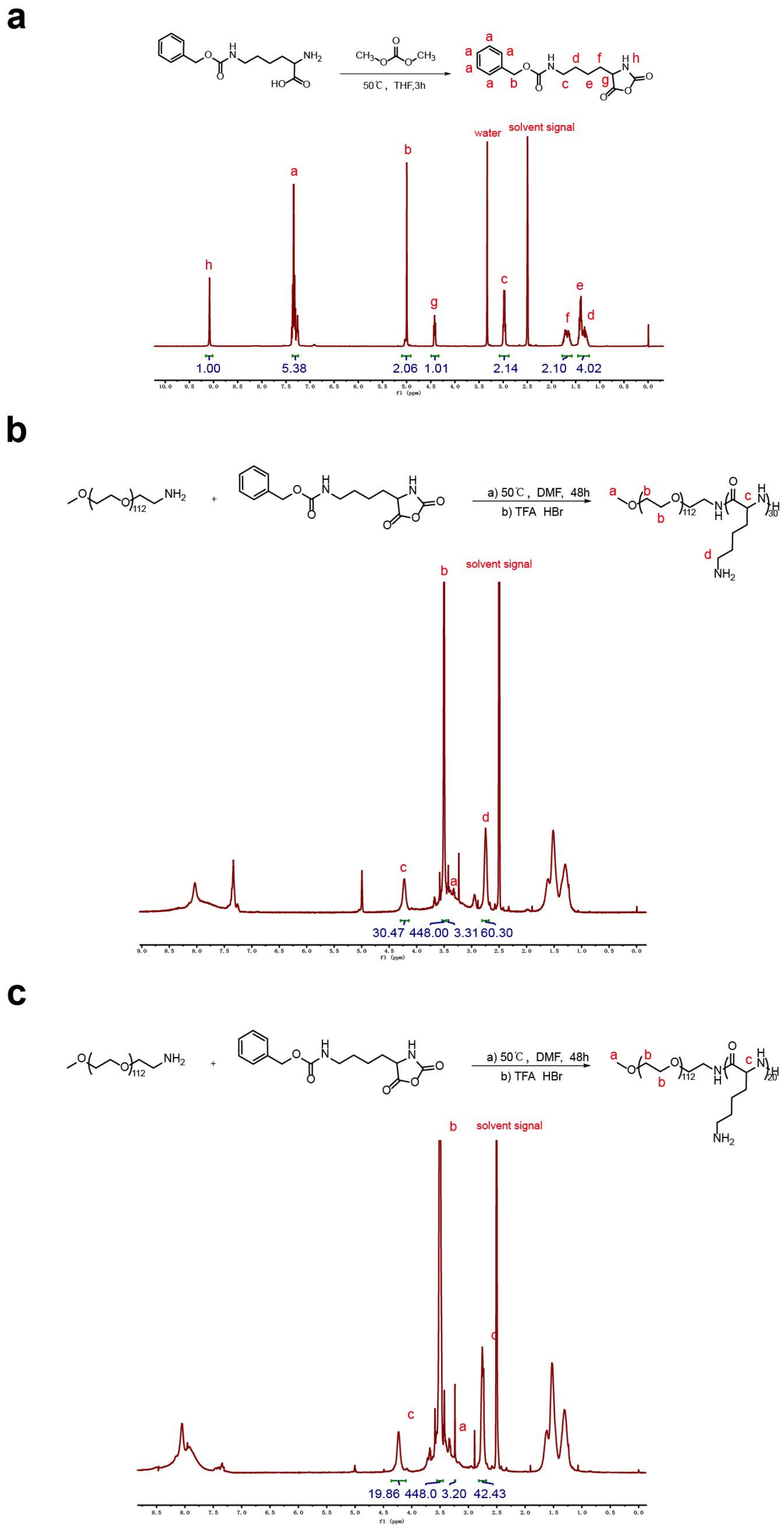

Supplementary Figure 4. (a) ^{1}H NMR spectra of Lys-NCA in DMSO-*d*6. (b) ^{1}H NMR spectra of PEG-Lys_{30} in DMSO-*d*6. (c) ^{1}H NMR spectra of PEG-Lys_{20} in DMSO-*d*6.

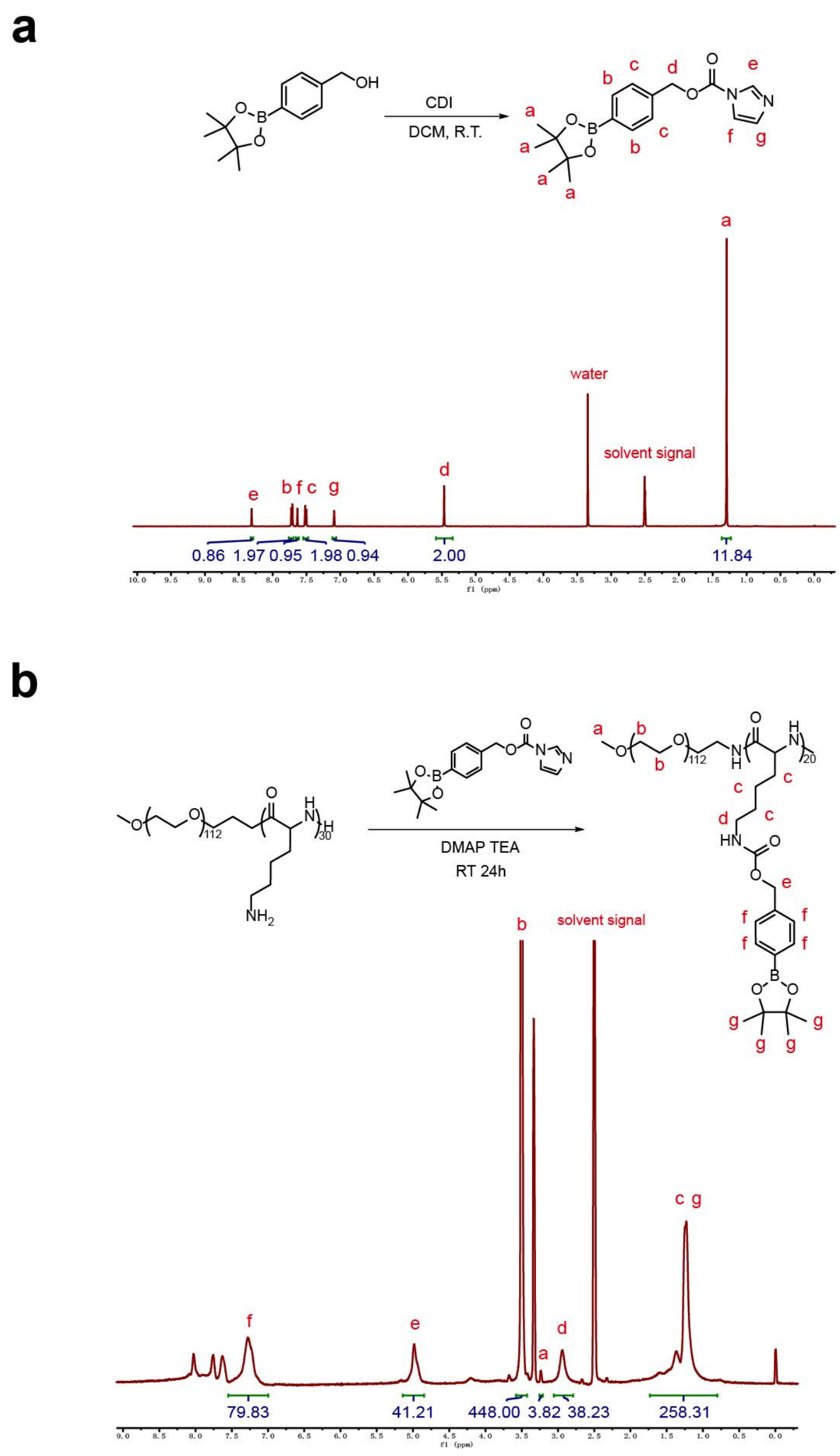

Supplementary Figure 5. (a) ^1H NMR spectra of the phenylboronic ester-CDI precursor in DMSO-*d*6. (b) ^1H NMR spectra of the Pb polymer in DMSO-*d*6.

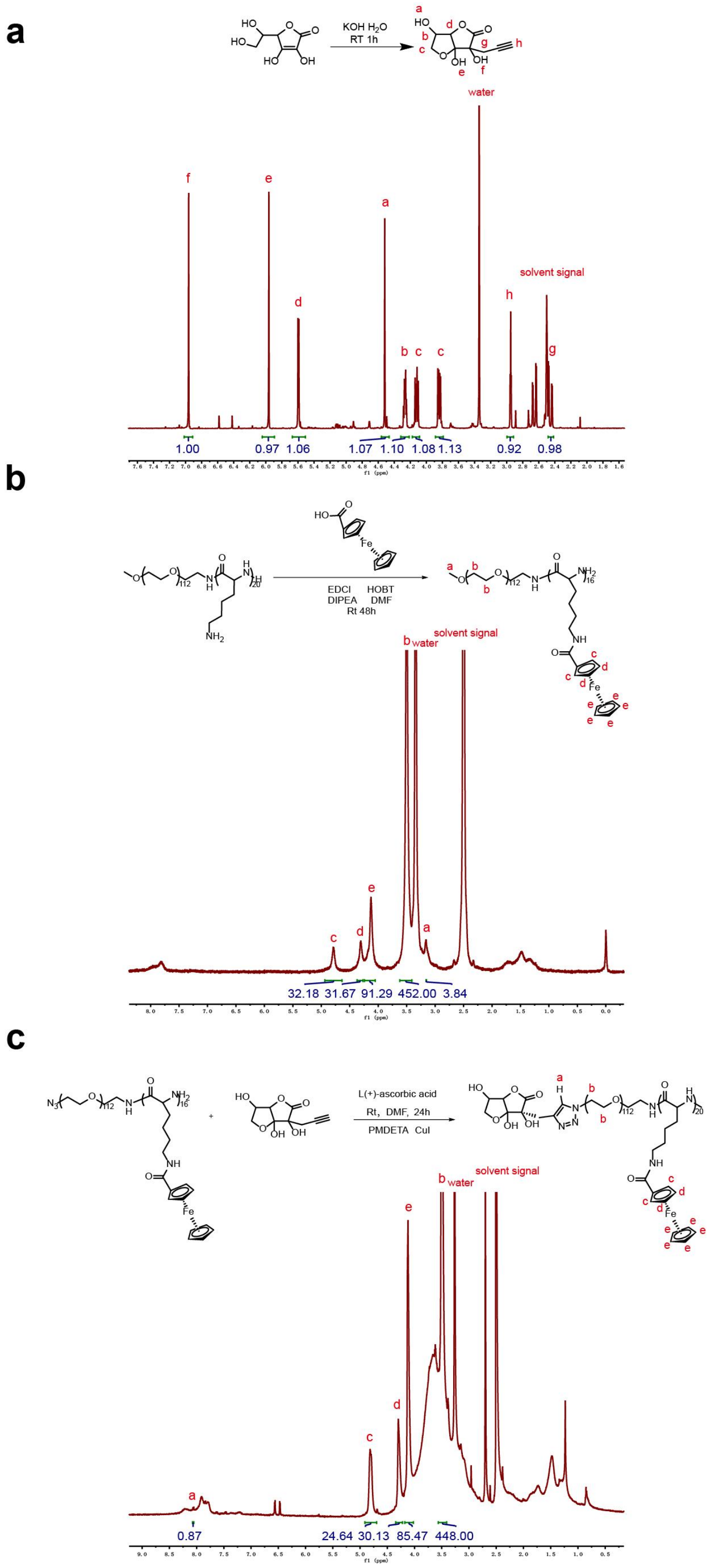

Supplementary Figure 6. (a) ^{1}H NMR spectra of DHAA in DMSO-*d*6. (b) ^{1}H NMR spectra of Fc polymer in DMSO-*d*6. (c) ^{1}H NMR spectra of DHA-modified Fc polymer in DMSO-*d*6.

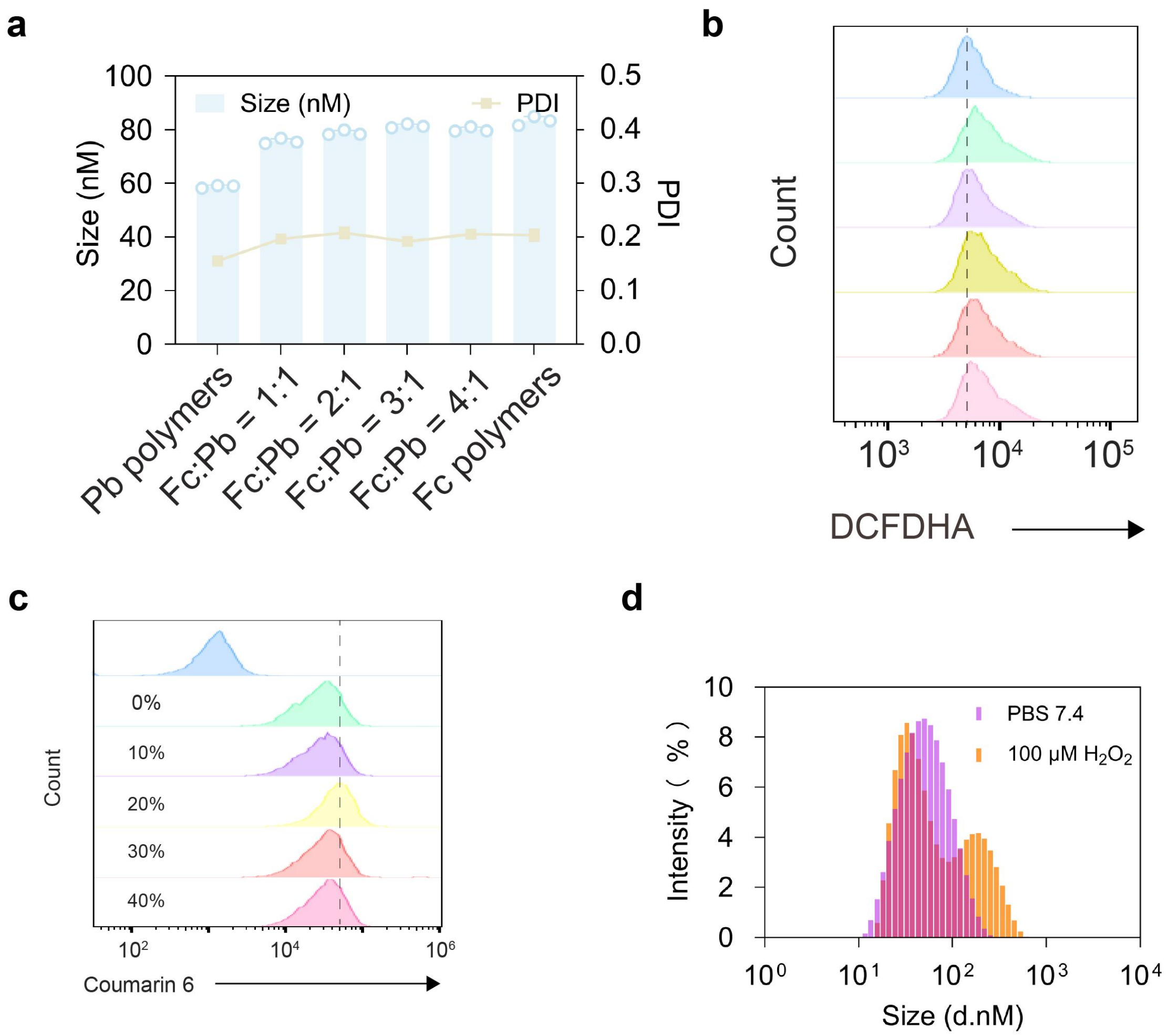

Supplementary Figure 7. (a) Particle size and PDI of blank nanoparticles formed from Pb polymer alone, Fc polymer alone, or from different ratios of the two polymers (n=3 independent samples). (b) Representative FCM images showing changes in intracellular oxidative stress in GL261 cells after uptake of different blank nanoparticles. (c) Representative FCM plots showing intracellular Coumarin 6 fluorescence intensity in GL261 cells after uptake of nanoparticles with different DHA modification ratios. (d) Changes in hydrodynamic size of nanoparticles after responsive drug release. Data are presented as mean ± SD, error bars represent SD.

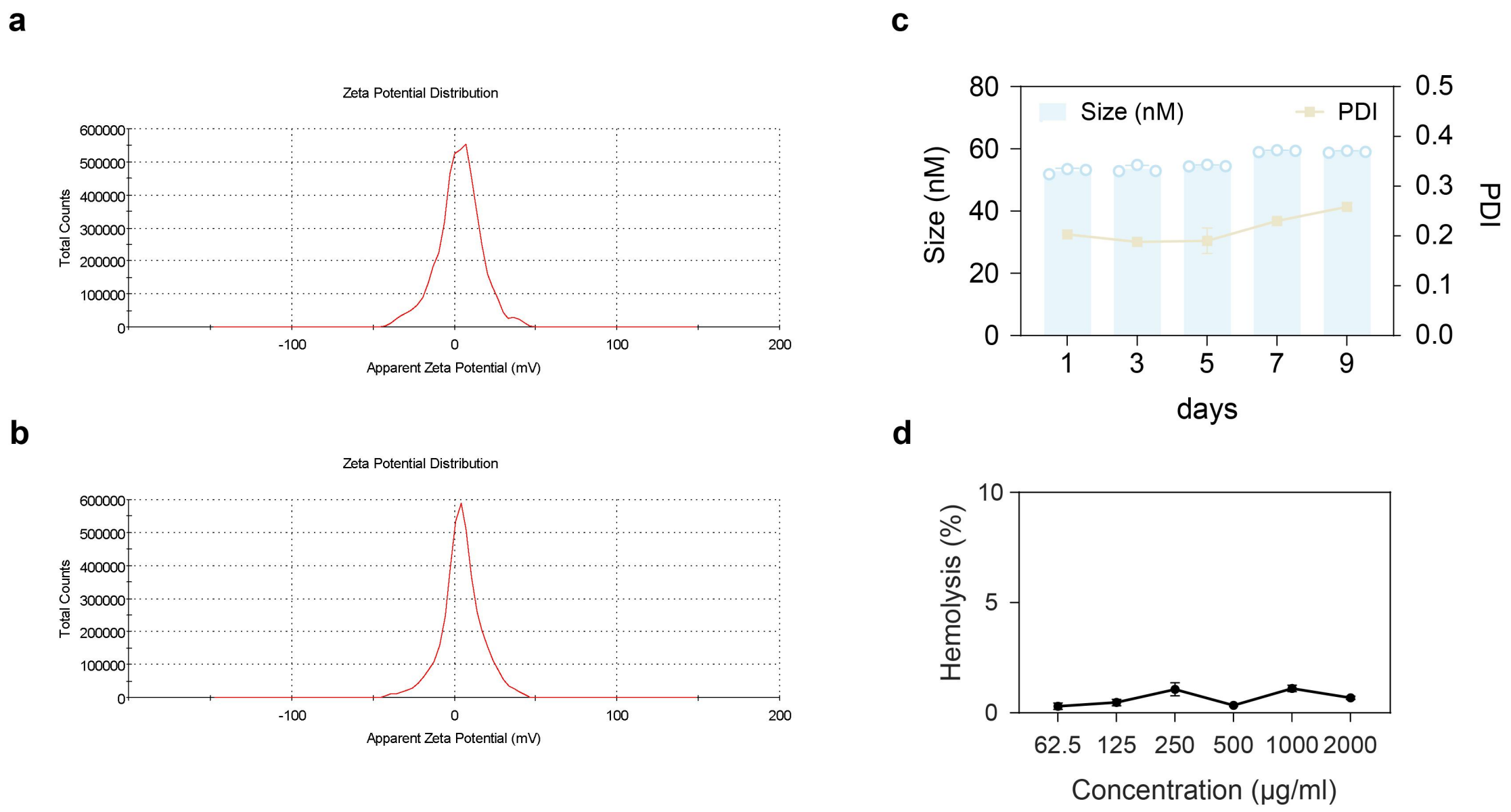

Supplementary Figure 8. (a) (b) Zeta potentials of GS@FP (a) and GS@DFP (b). (c) Changes in particle size and PDI of GS@DFP stored at 4 °C over 9 days (n=3 independent samples). (d) Hemolysis assay demonstrating the safety of the nanoparticles (n=3 independent samples). Data are presented as mean ± SD, error bars represent SD.

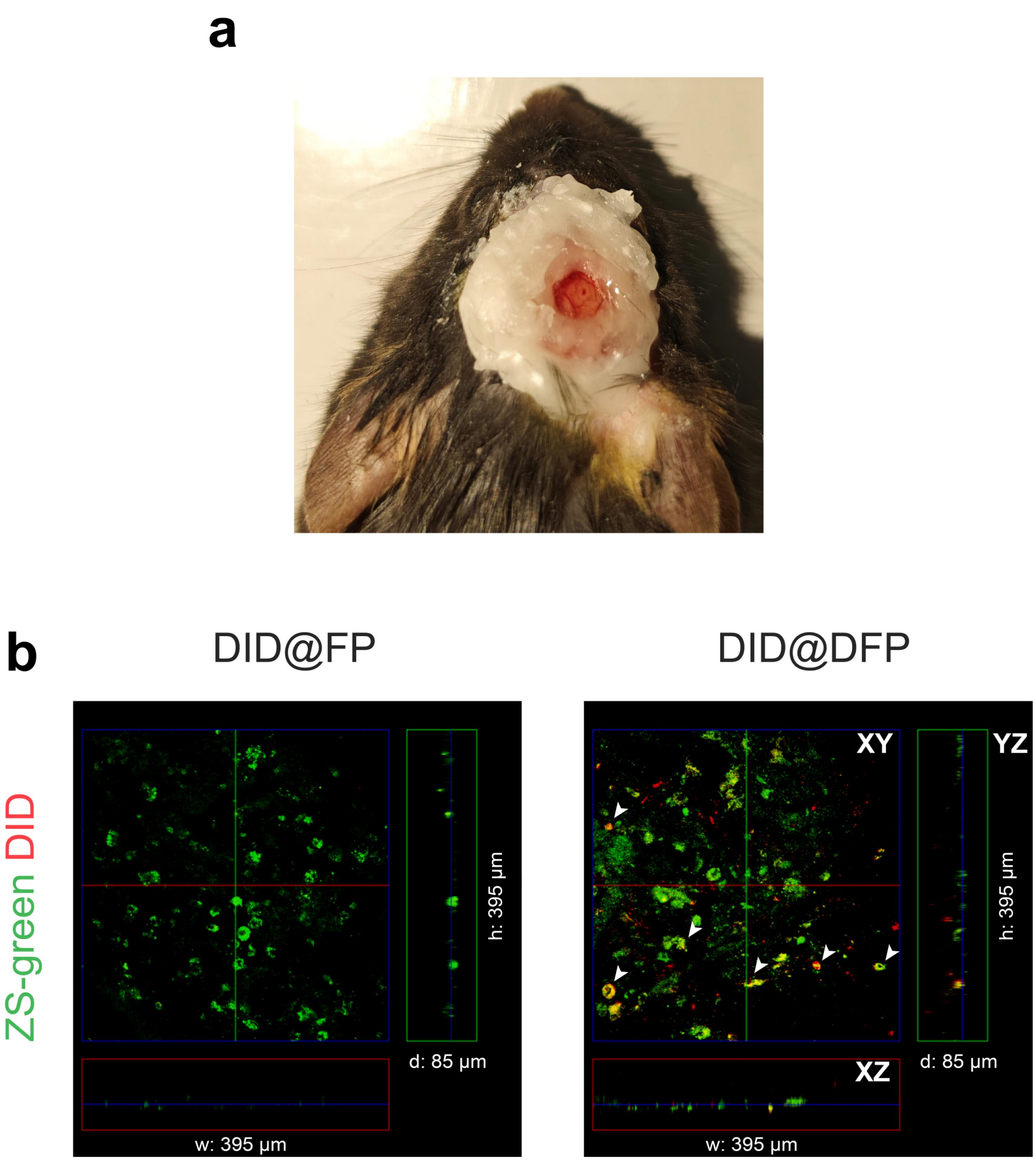

Supplementary Figure 9. (a) Schematic representation of the cranial window ZS-green GL261 tumor-bearing mouse model. (b) Maximum projection orthogonal view under two-photon microscopy 24 h after intravenous administration of different DID-loaded nanoparticles in tumor-bearing mouse models.

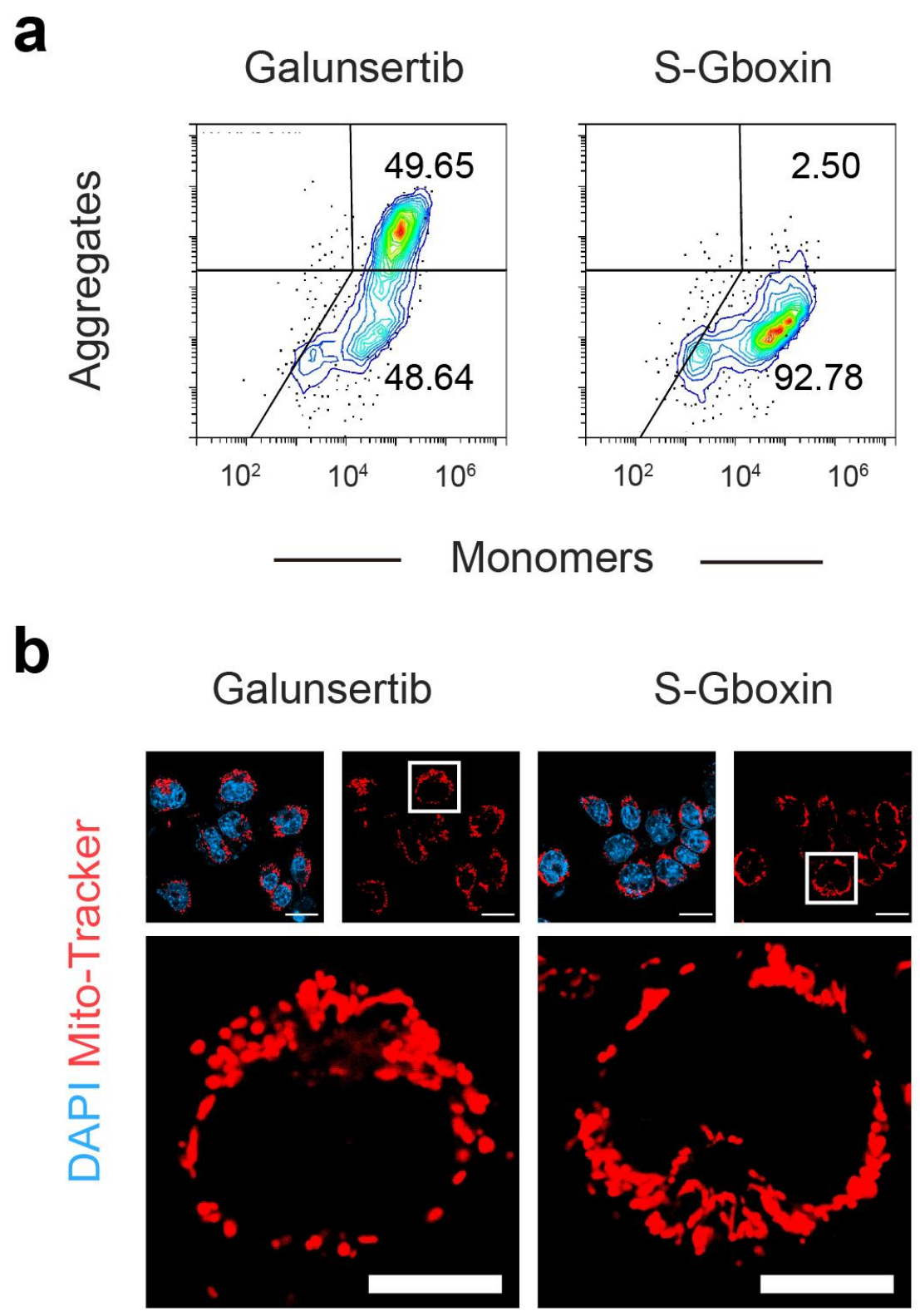

Supplementary Figure 10. (a) Representative flow cytometry plots showing JC-1 probe fluorescence changes after treatment with Galunisertib and S-Gboxin. (b) Mito-Tracker detection of mitochondrial dye leakage in cancer cells after Galunisertib and S-Gboxin treatment. Scale bar = 10 μm in ZOOM.

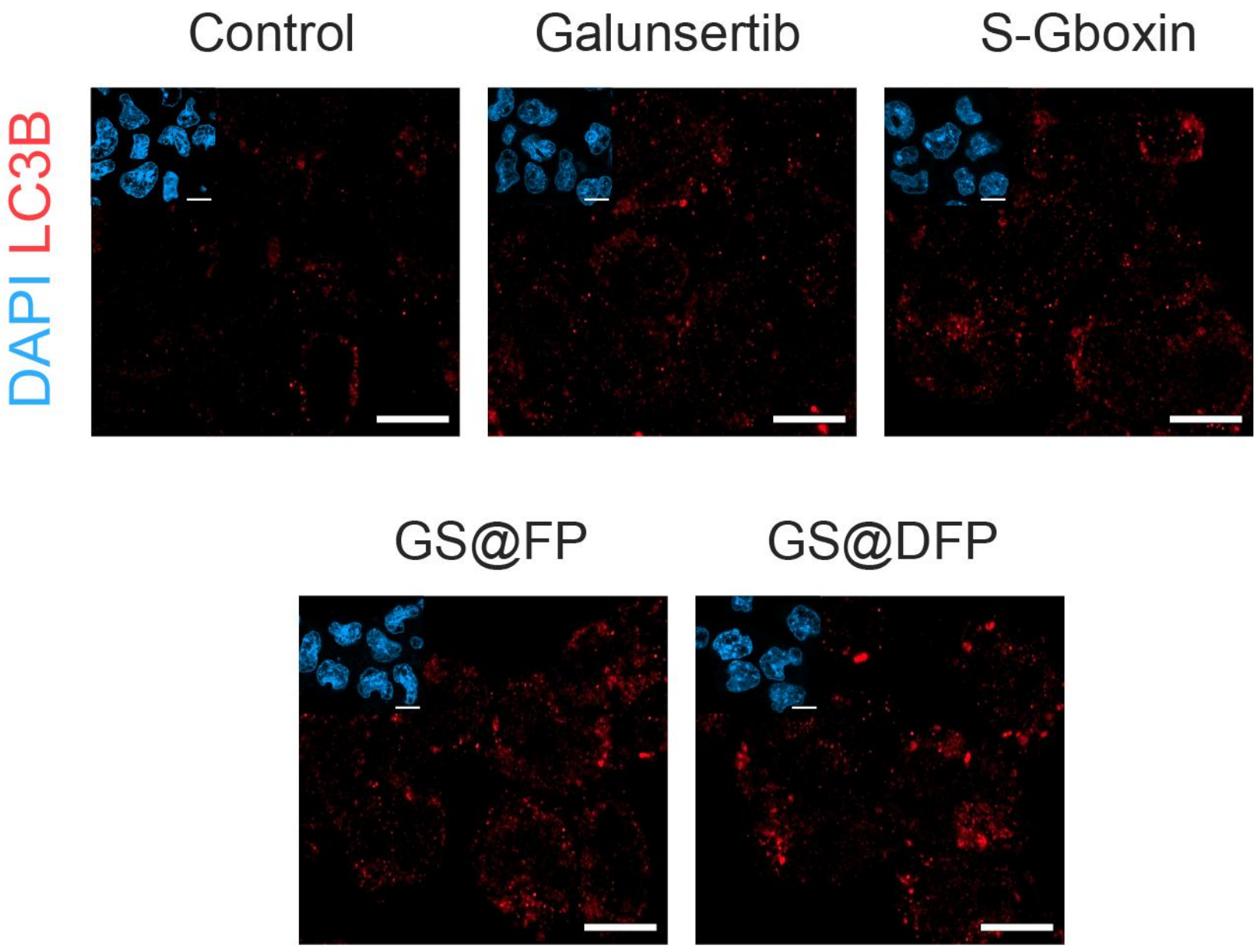

Supplementary Figure 11. Representative fluorescence images showing the level of autophagy in GL261 cells after different treatments. Scale bar = 20 μm.

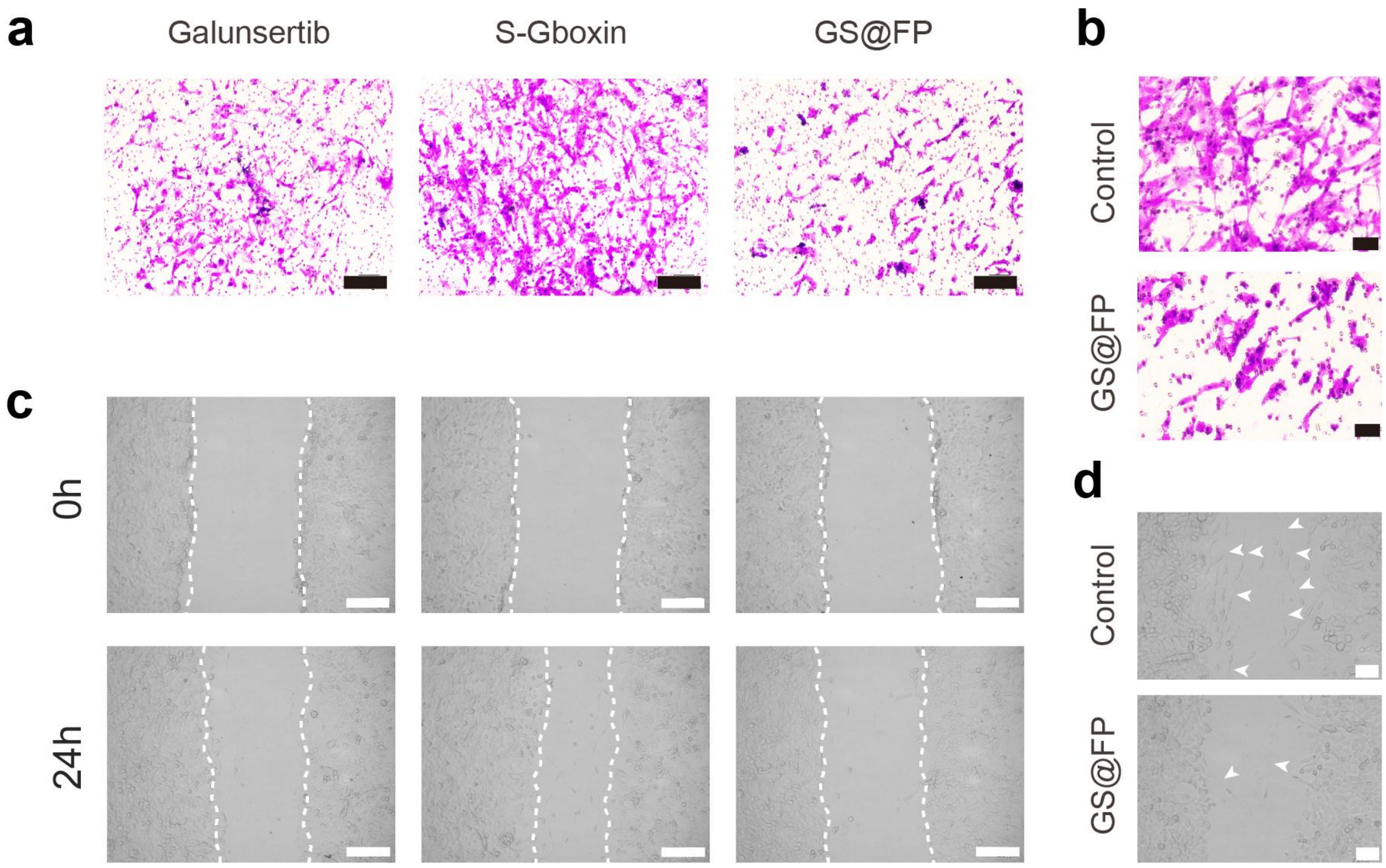

Supplementary Figure 12. (a) Representative images showing inhibition of tumor cell invasion by Galunisertib, S-Gboxin, and GS@FP. Scale bar = 200 μm; (b) Local representative images of tumor cell invasion inhibition by GS@FP. Scale bar = 50 μm; (c) Representative images showing inhibition of tumor cell migration by Galunisertib, S-Gboxin, and GS@FP. Scale bar = 200 μm; (d) Local representative images of tumor cell migration inhibition by GS@FP, white arrows indicate tumor microtubes. Scale bar = 50 μm.

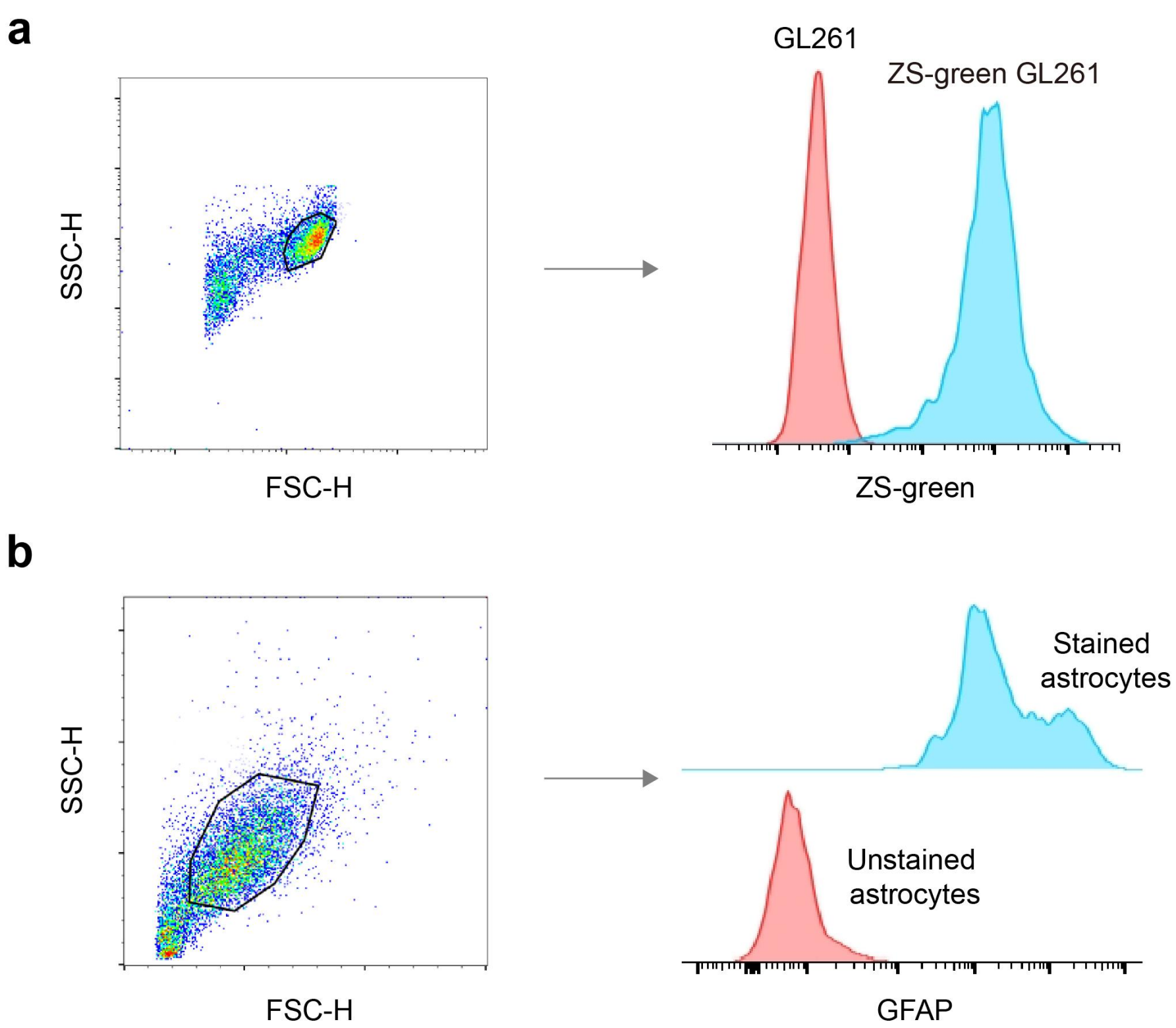

Supplementary Figure 13. (a) Flow cytometric analysis of ZS-green GL261 transfection. (b) Flow cytometric assessment of astrocyte purity after staining astrocytes with FITC-conjugated GFAP antibody.

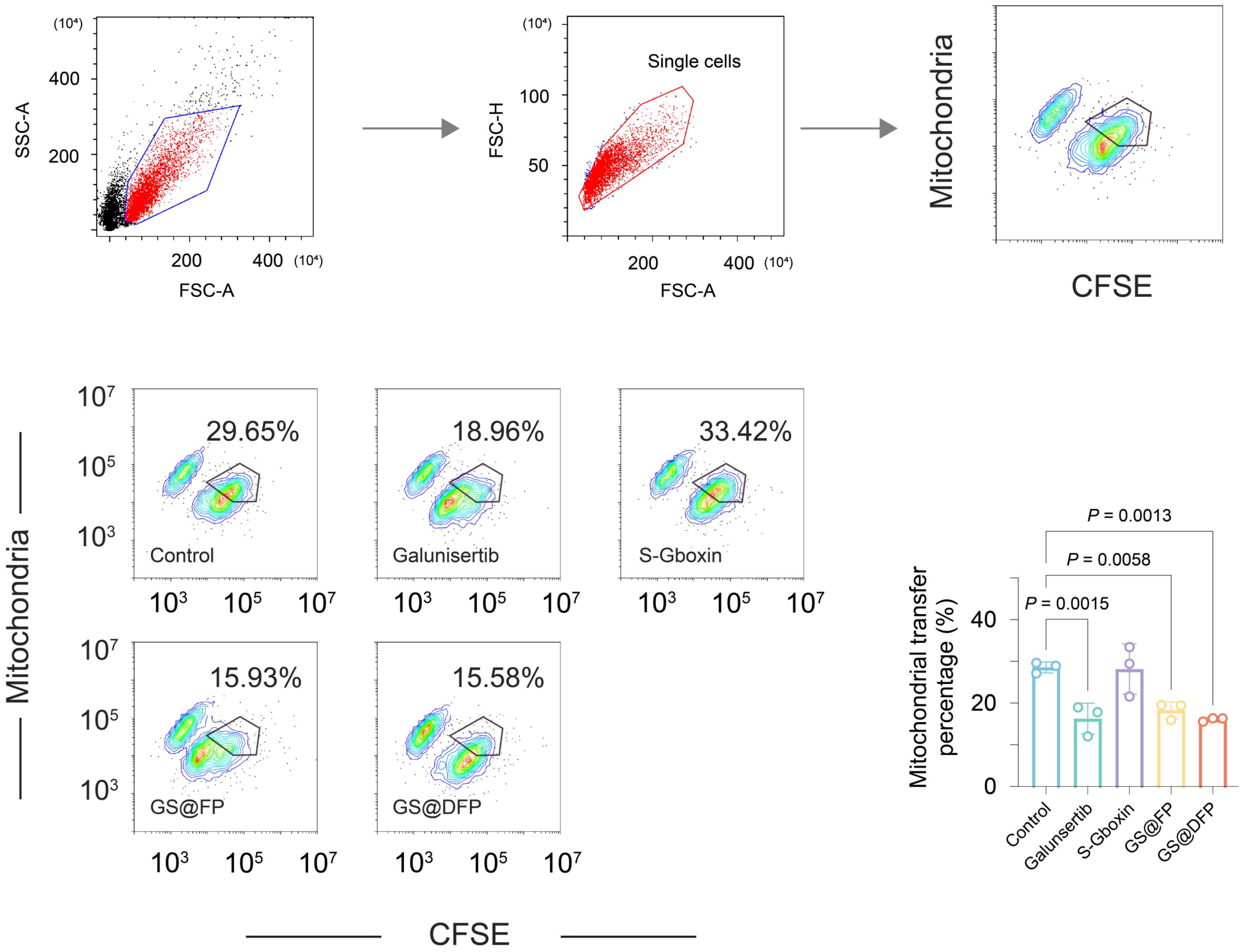

Supplementary Figure 14. GL261 cells from different treatment groups were stained with CFDASE and co-cultured for 24 h with astrocytes pre-stained with Mito-Tracker Red. Flow cytometry gating strategy and quantification of cancer cells receiving transferred mitochondria (n=3 independent samples). Data are presented as mean ± SD, error bars represent SD. One-way ANOVA with Tukey's multiple comparisons test was used to compare multiple groups.

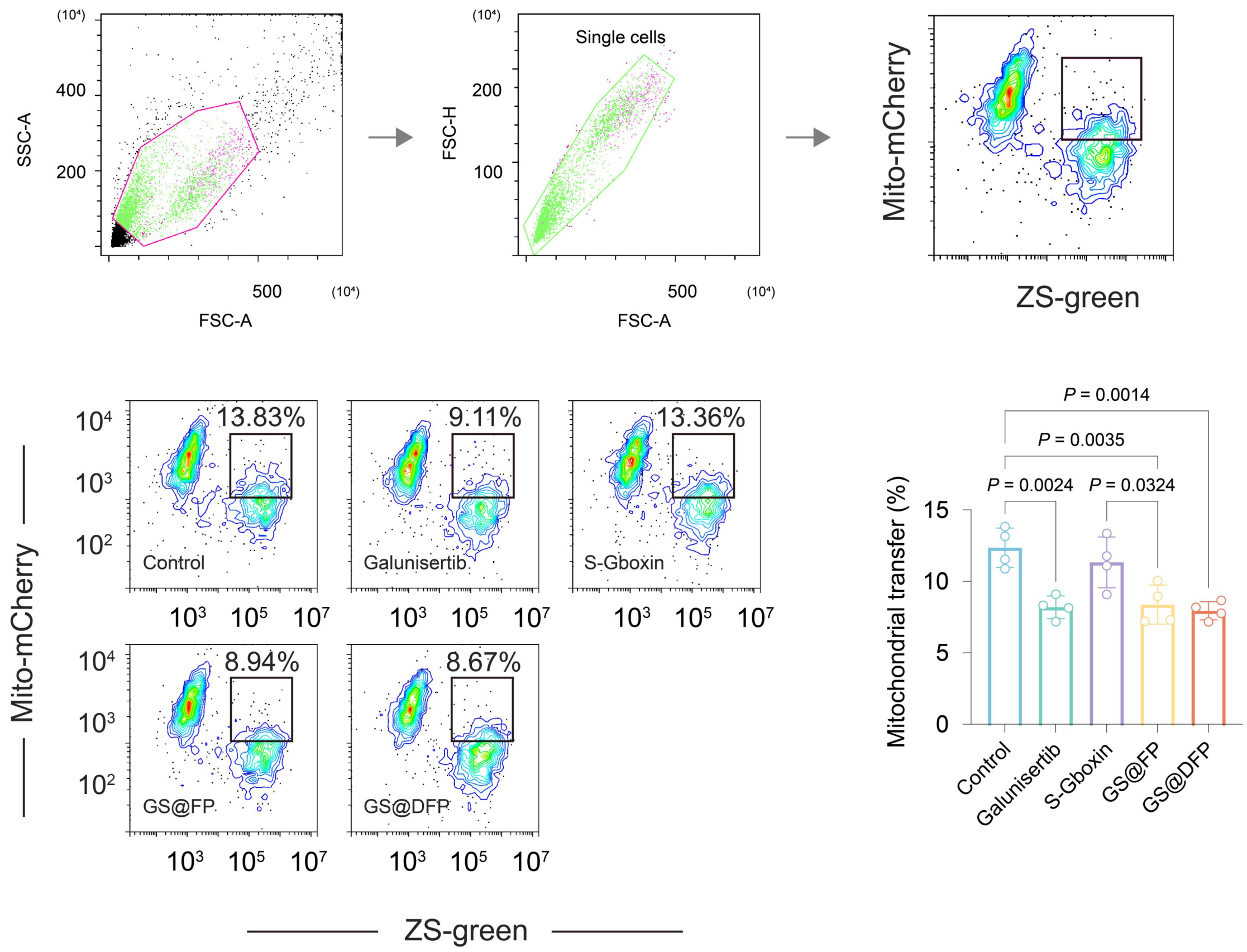

Supplementary Figure 15. ZS-green GL261 cells from different treatment groups were co-cultured for 24 h with astrocytes pre-transfected with Mito-mCherry using Lipo6000. Flow cytometry gating strategy and quantification of cancer cells receiving transferred mitochondria (n = 4 independent samples). Data are presented as mean ± SD, error bars represent SD. One-way ANOVA with Tukey's multiple comparisons test was used to compare multiple groups.

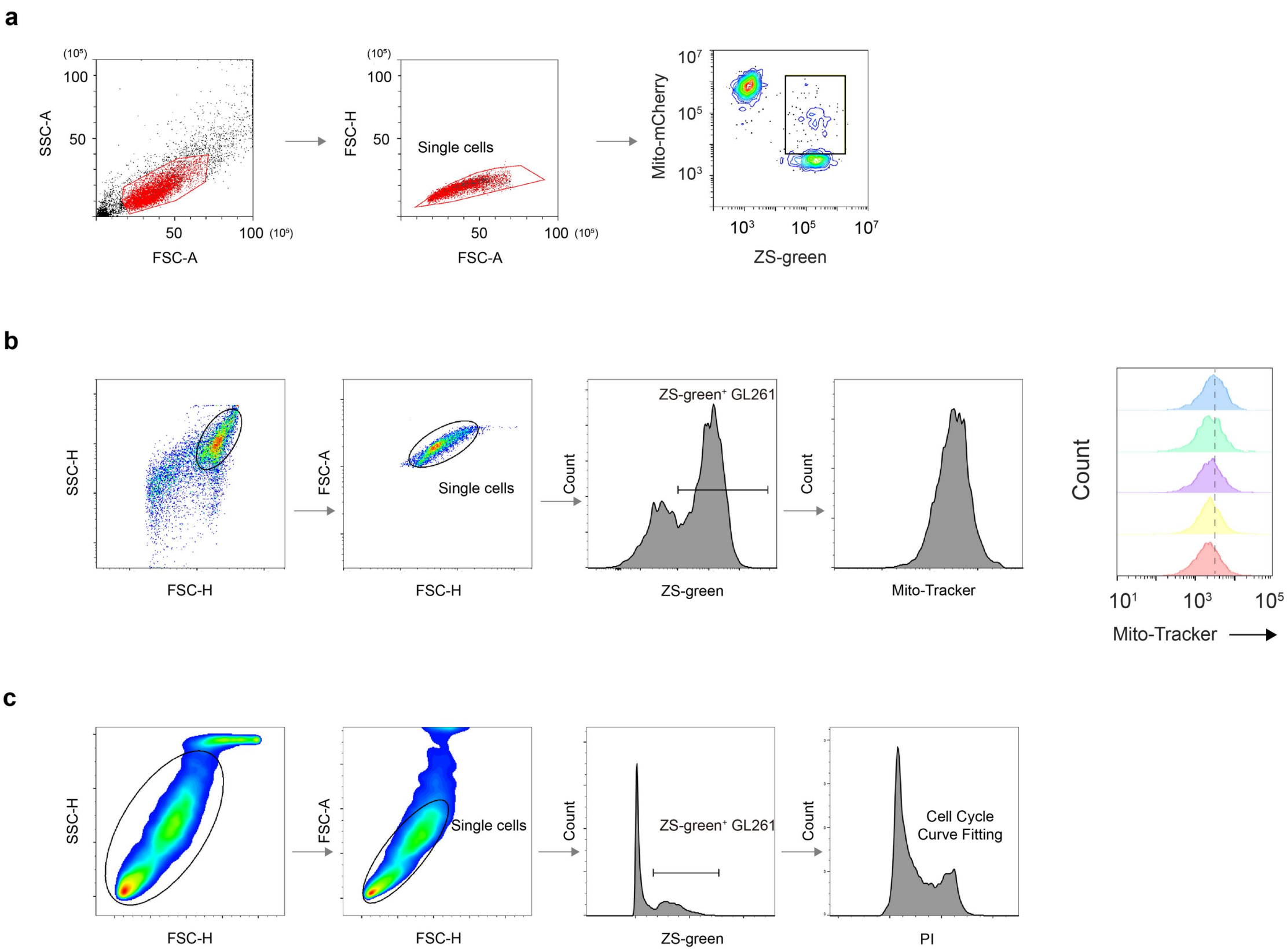

Supplementary Figure 16. (a) ZS-green GL261 cells after 24 h of different treatments were co-cultured for 24 h with astrocytes pre-transfected with Mito-mCherry using Lipo8000, and flow cytometry was used to detect the gating strategy of cancer cells receiving transferred mitochondria. (b) ZS-green GL261 cells after 24 h of different treatments were co-cultured with astrocytes for 24 h, and flow cytometry was used to detect the gating strategy of total mitochondrial content in cancer cells. (c) ZS-green GL261 cells after 24 h of different treatments were co-cultured with astrocytes for 24 h, stained with PI, and flow cytometry was used to detect the gating strategy of the proportion of cells in different cell cycle phases.

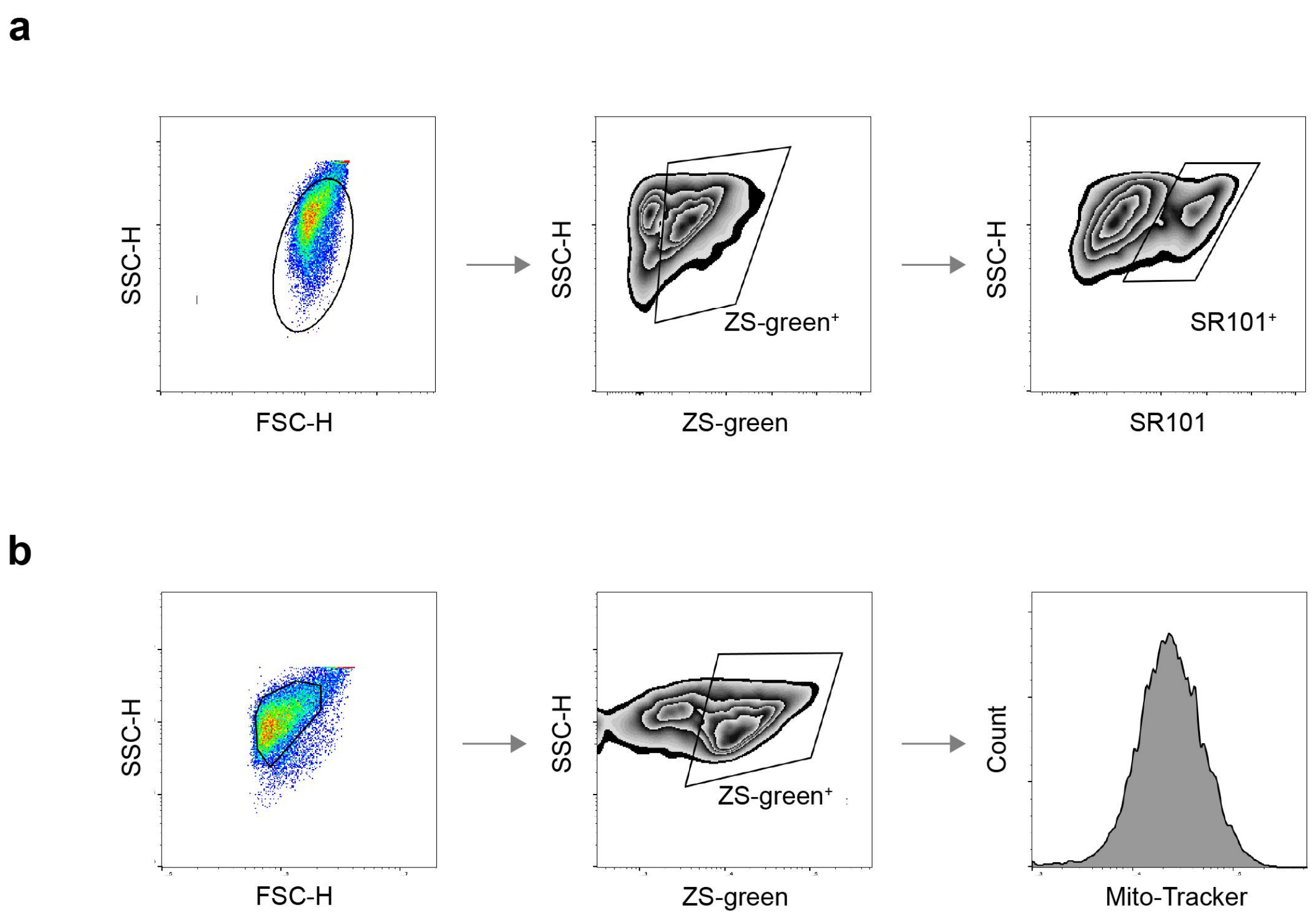

Supplementary Figure 17. (a) Tumor-bearing mice from different treatment groups were intraperitoneally injected with SR101 10 h prior, then brain tissues were enzymatically digested and triturated, and flow cytometry was used to detect the gating strategy for the proportion of cancer cells that acquired SR101 from astrocytes. (b) Brain tissues from tumor-bearing mice of different treatment groups were enzymatically digested and triturated, stained with Mito-Tracker Red for 20 min, and flow cytometry was used to detect the gating strategy for total mitochondrial content in cancer cells.

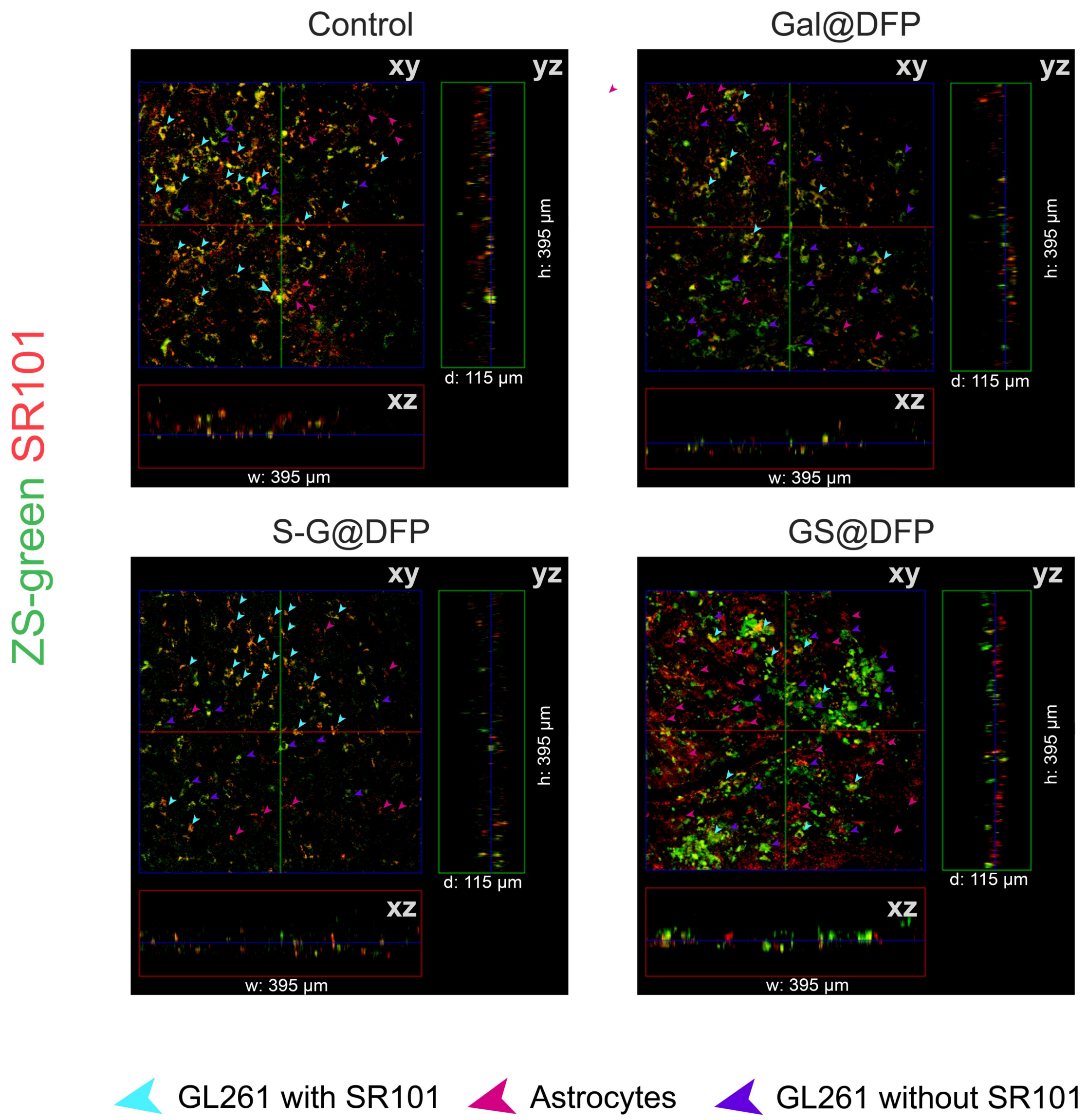

Supplementary Figure 18. Maximum orthogonal imaging plane observed by two-photon microscopy 10 h after intraperitoneal injection of SR101 in tumor-bearing mice at the end of treatment.

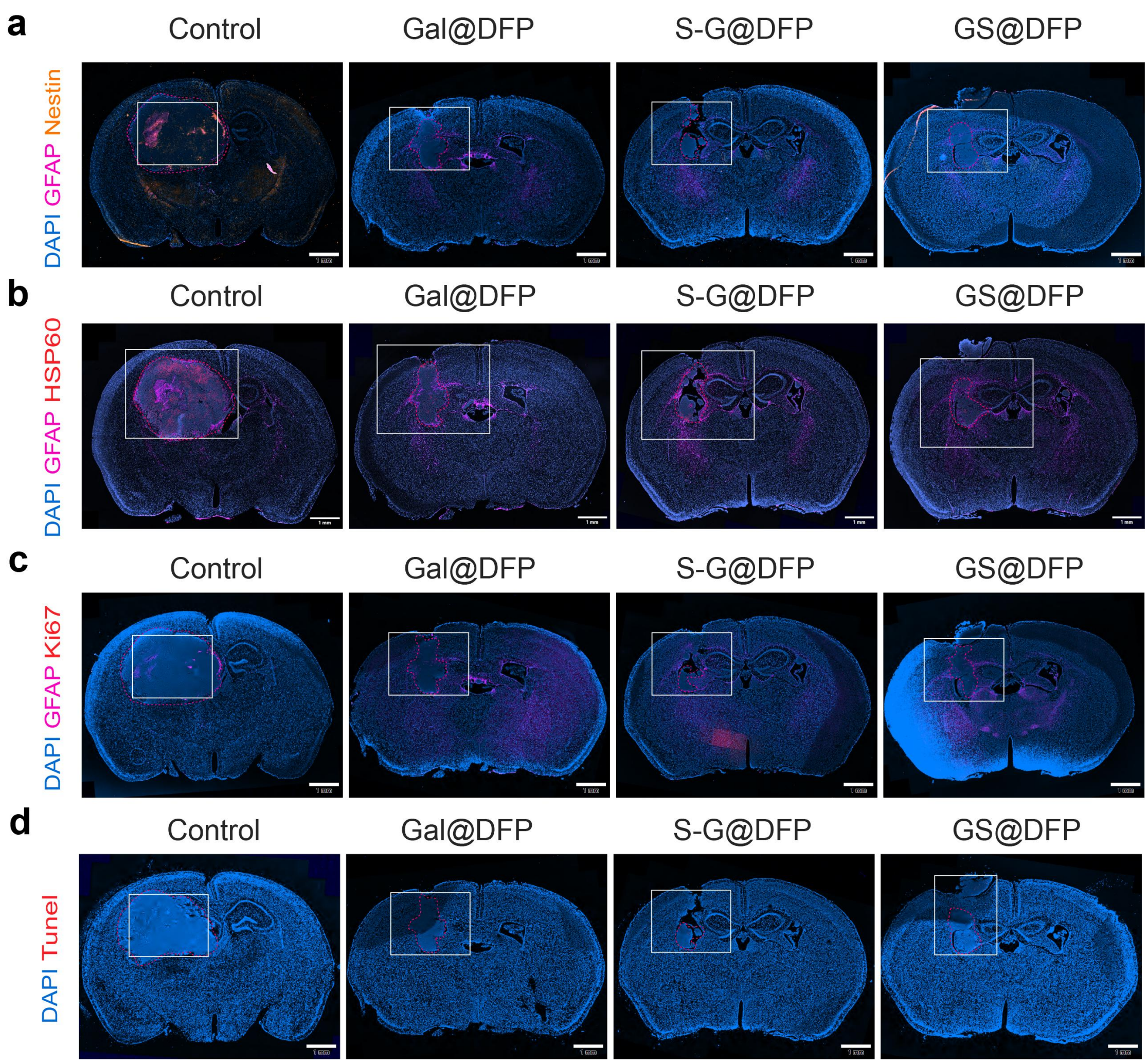

Supplementary Figure 19. (a) (b) (c) (d) Representative panoramic images of brain sections from tumor-bearing mice after different treatments, immunofluorescently stained for the tumor microtubule-associated protein Nestin (a), mitochondrial marker HSP60 (b), proliferation marker Ki67 (c), and apoptosis marker TUNEL (d). Scale bar = 1 mm

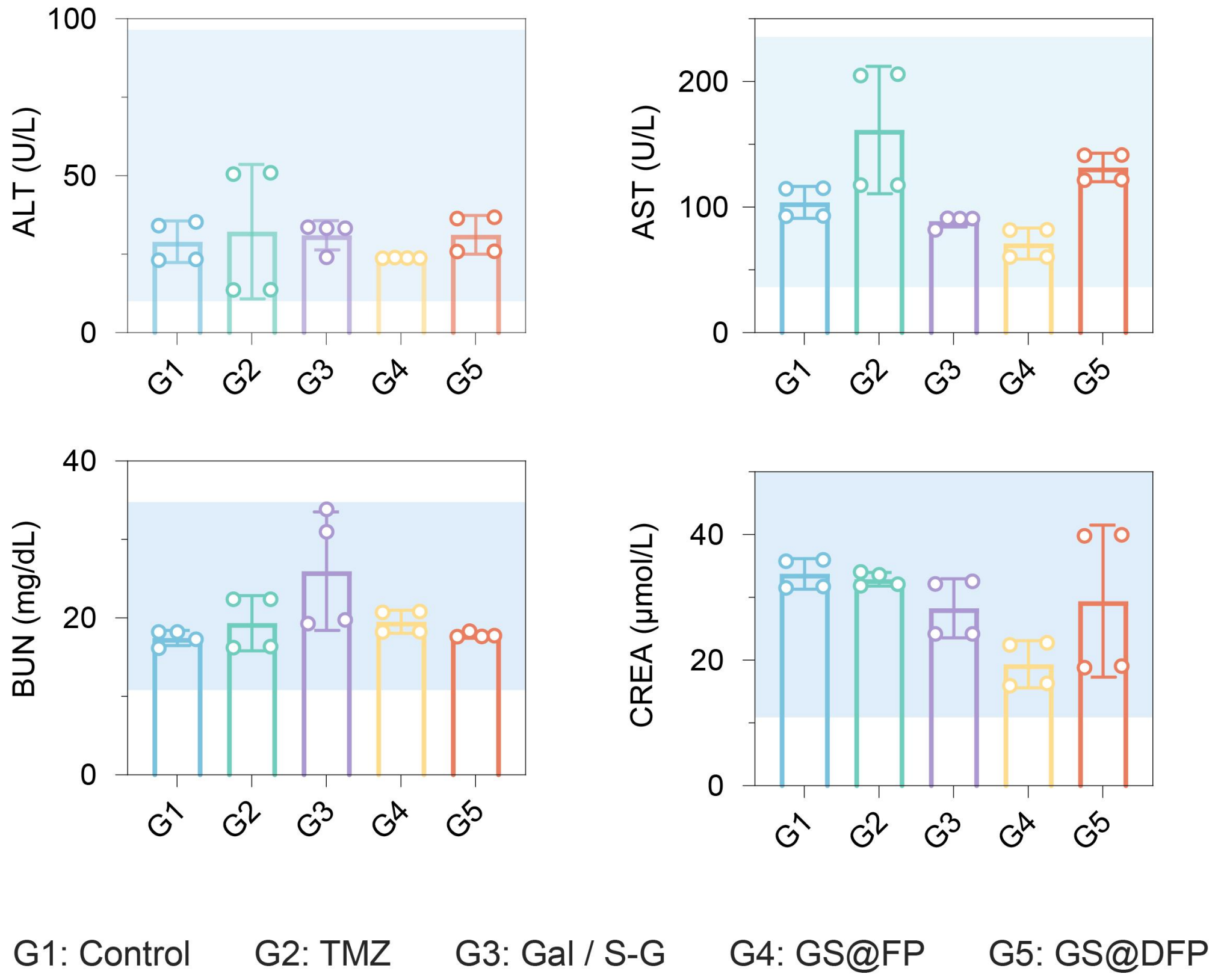

Supplementary Figure 20. Semi-quantitative analysis of ALT, AST, BUN, and CREA levels in the serum of mice from different treatment groups. Blue shading indicates the normal range for each parameter. ALT, alanine aminotransferase; AST, aspartate aminotransferase; CREA, creatinine; BUN, blood urea nitrogen (n=4 independent samples). Data are presented as mean ± SD, error bars represent SD.

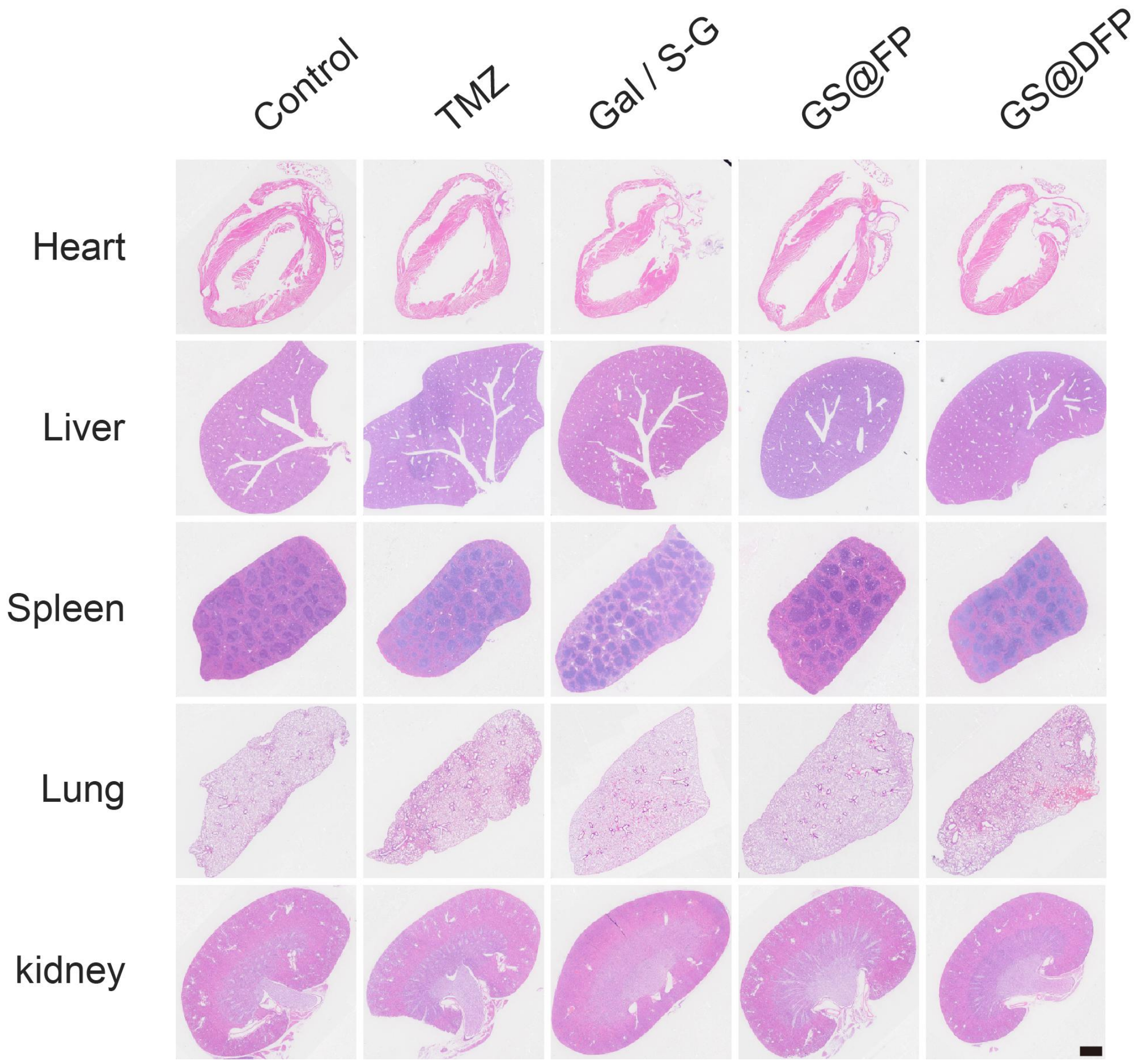

1 **Supplementary Figure 21.** H&E-stained sections of major organs from mice in different

2 treatment groups. Scale bar = 1 mm.

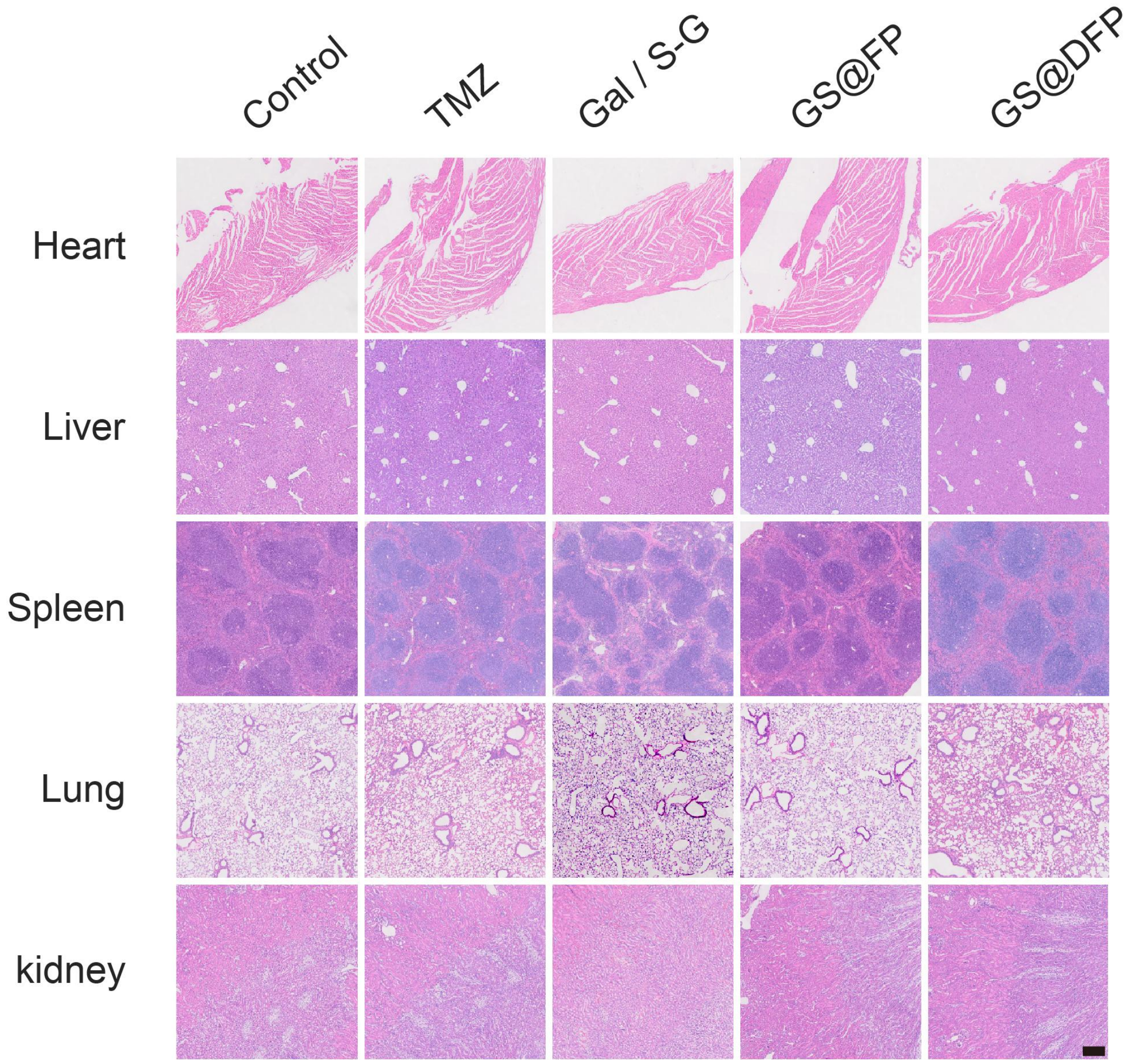

Supplementary Figure 22. Magnified views of H&E-stained sections of major organs from mice in different treatment groups. Scale bar = 200 μm.

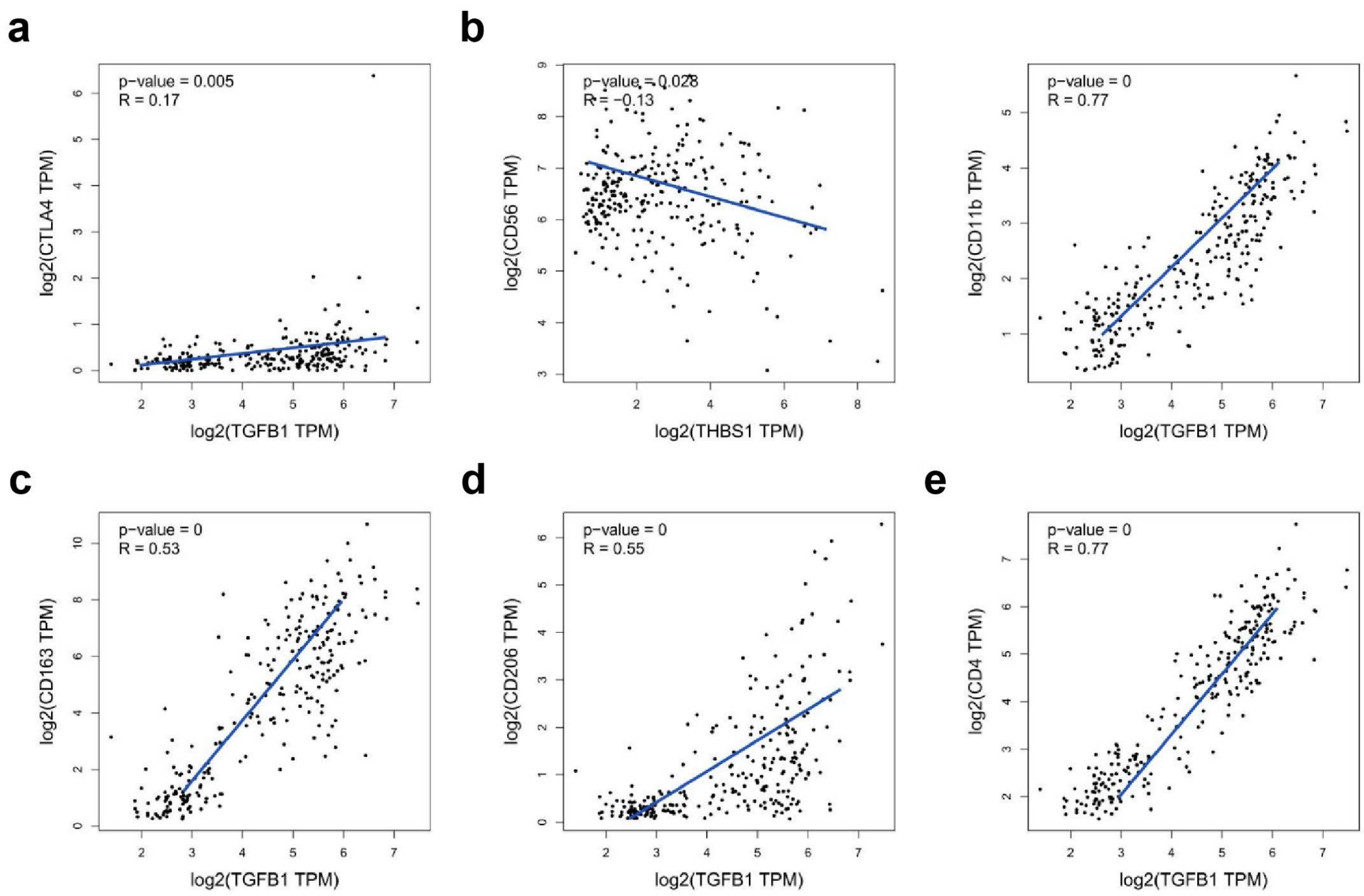

Supplementary Figure 23. Correlation analysis of TGFB1 / THBS1 (TSP-1) with various immune cell markers using GEPIA. (a) Correlation between TGFB1 and CTLA4; (b) Correlation between THBS1 and CD56, CD11b; (c) Correlation between TGFB1 and CD163; (d) Correlation between TGFB1 and CD206; (e) Correlation between TGFB1 and CD4.

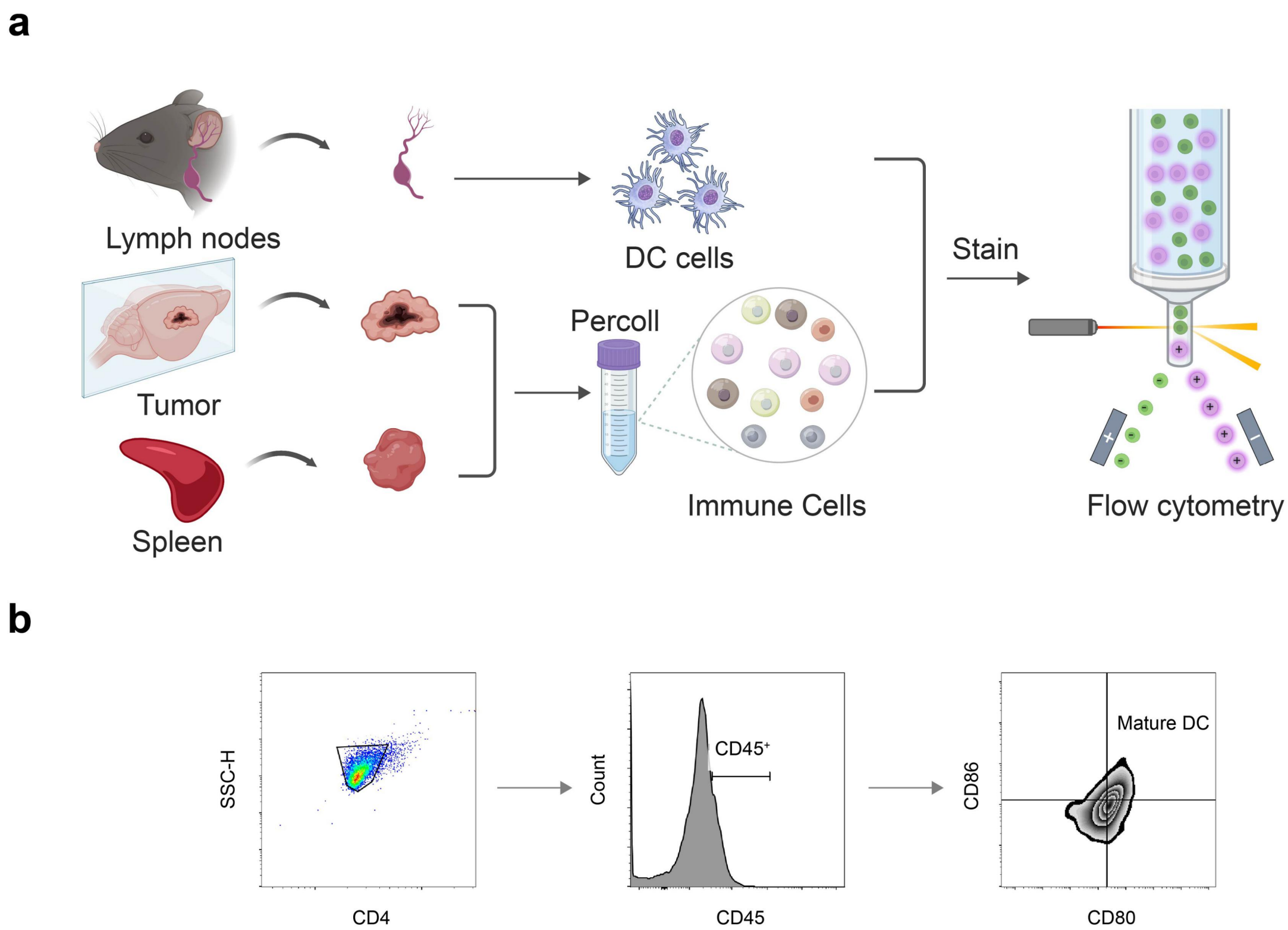

Supplementary Figure 24. (a) Schematic illustration of immune cell activation in the cervical lymph nodes, brain tumors, and spleen of tumor-bearing mice. (b) Gating strategy for flow cytometry analysis of DC cell maturation in the cervical lymph nodes. Created in BioRender. Jiang, C. (2025). https://BioRender.com/8x6yk6f.

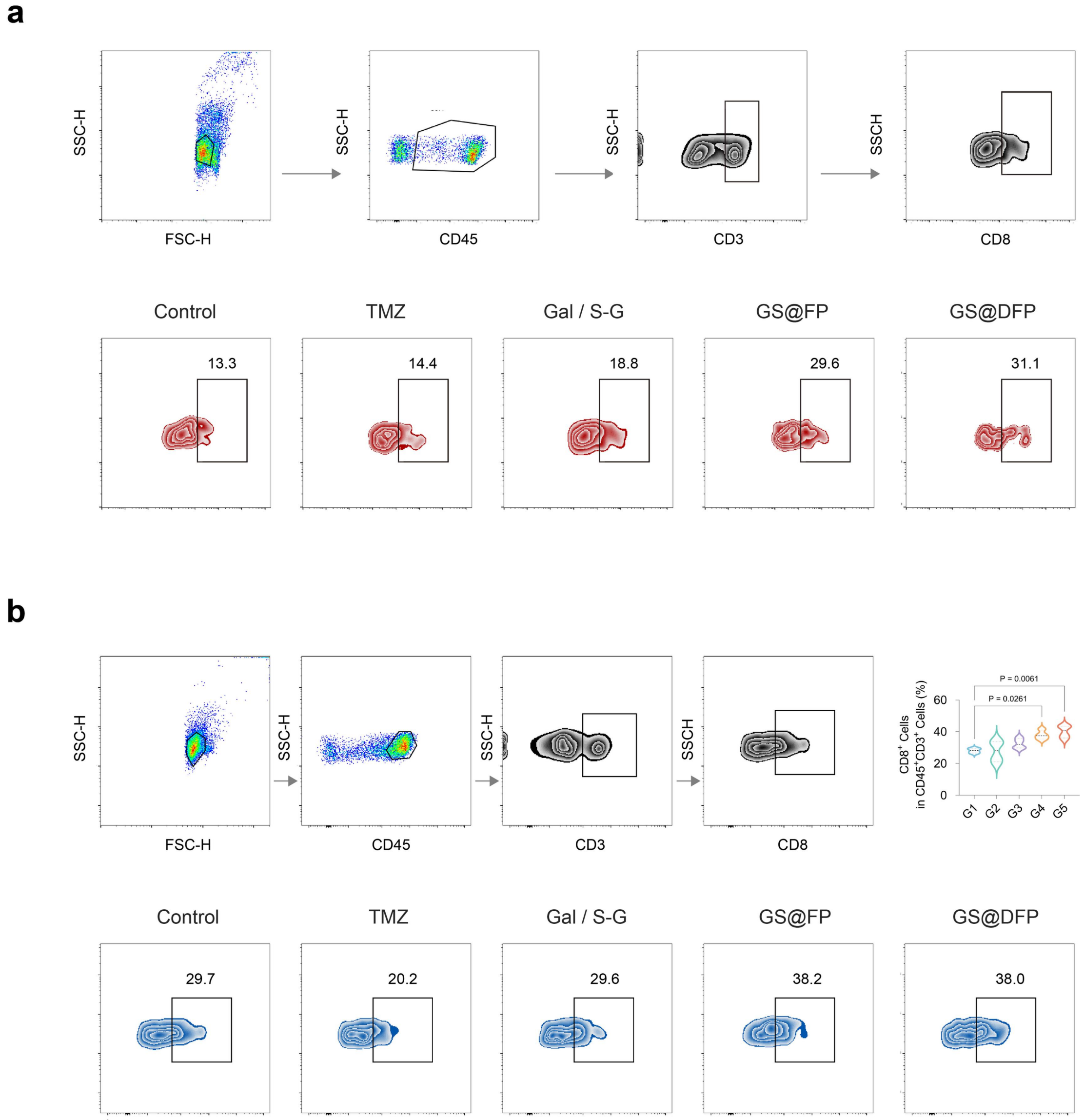

Supplementary Figure 25. (a) Gating strategy and representative flow cytometry plots for flow cytometry analysis of cytotoxic T cell infiltration in brain tumors. (b) Gating strategy and representative flow cytometry plots with quantification of cytotoxic T cell infiltration in the spleen (n=4 independent samples). Data are presented as mean ± SD, error bars represent SD. One-way ANOVA with Tukey's multiple comparisons test was used to compare multiple groups.

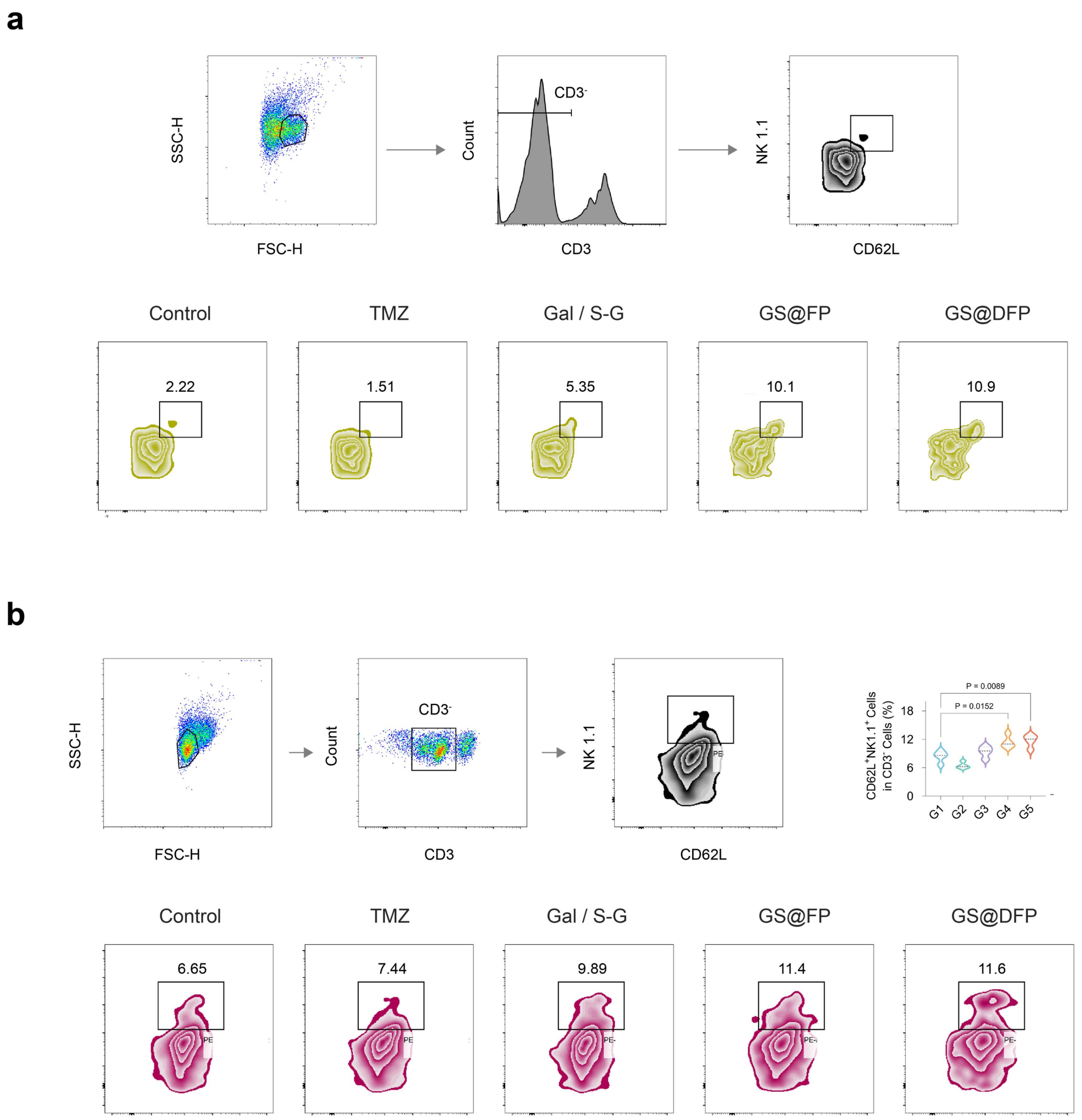

Supplementary Figure 26. (a) Gating strategy and representative flow cytometry plots for flow cytometry analysis of NK cell infiltration in brain tumors. (b) Gating strategy and representative flow cytometry plots with quantification of NK cell infiltration in the spleen (n=4 independent samples). Data are presented as mean ± SD, error bars represent SD. One-way ANOVA with Tukey's multiple comparisons test was used to compare multiple groups.

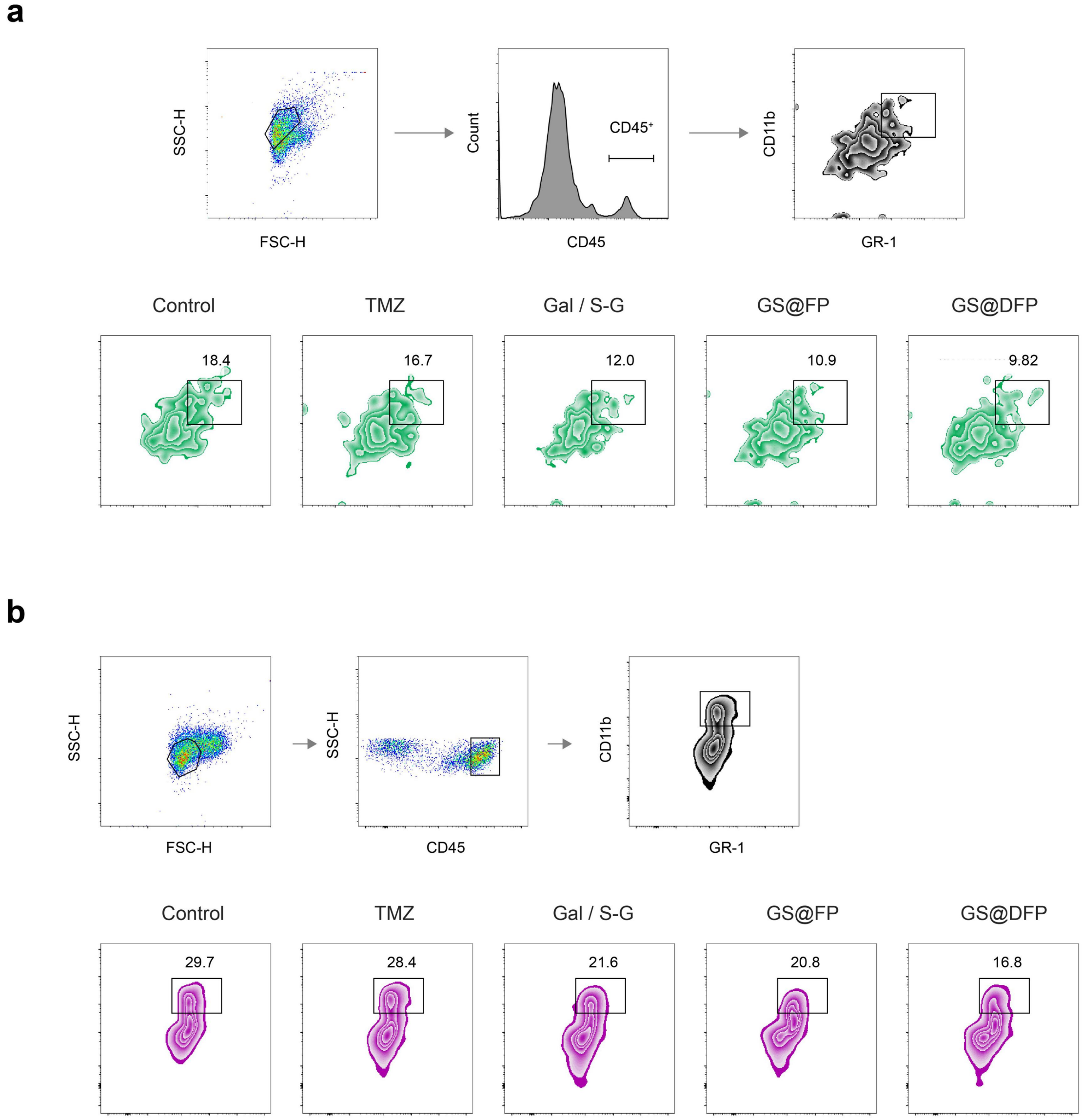

Supplementary Figure 27. (a) Gating strategy and representative flow cytometry plots for flow cytometry analysis of MDSC cell infiltration in brain tumors. (b) Gating strategy and representative flow cytometry plots of MDSC cell infiltration in the spleen.

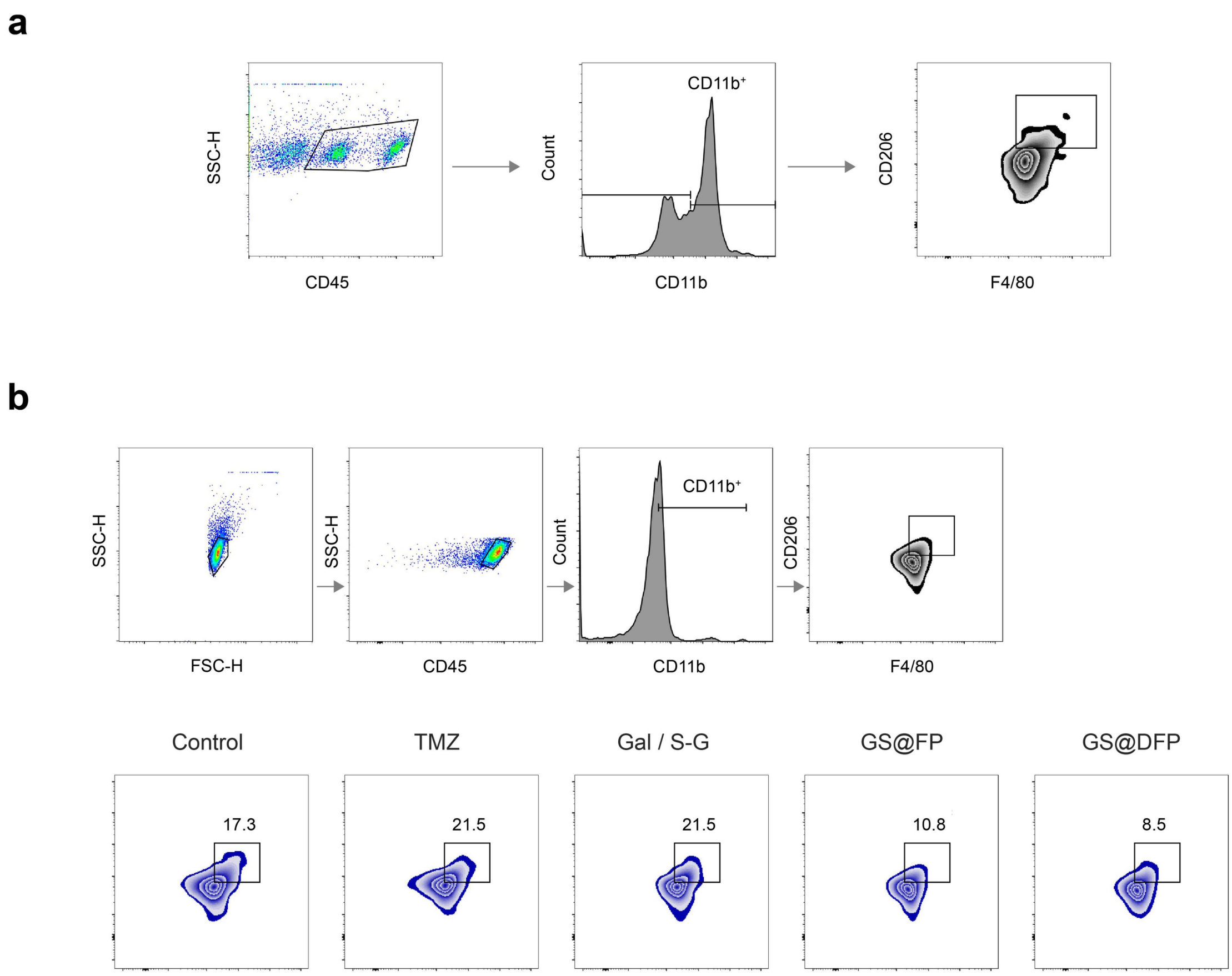

Supplementary Figure 28. (a) Gating strategy for flow cytometry analysis of TAM cell infiltration in brain tumors. (b) Gating strategy and representative flow cytometry plots of TAM cell infiltration in the spleen.

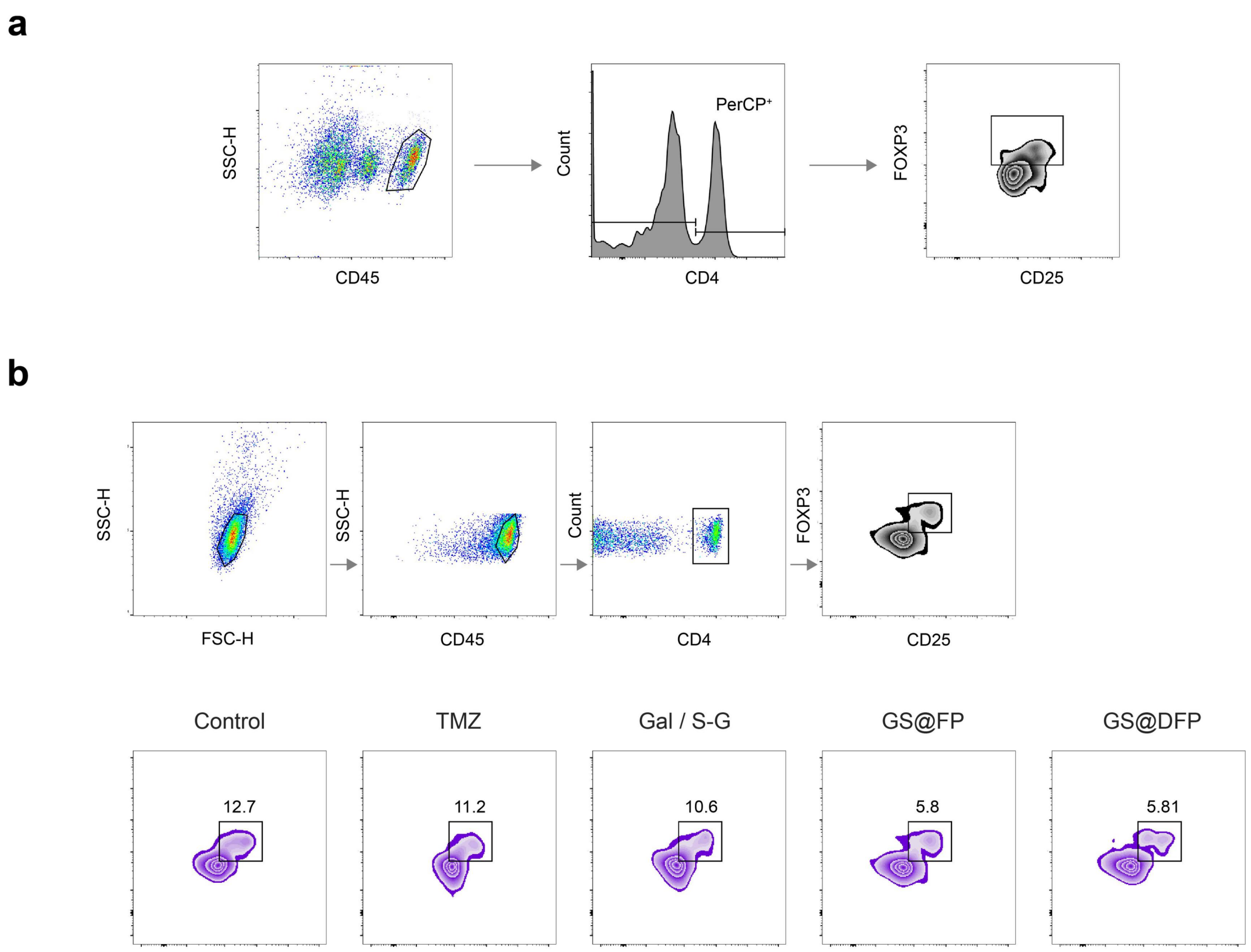

Supplementary Figure 29. (a) Gating strategy for flow cytometry analysis of Treg cell infiltration in brain tumors. (b) Gating strategy and representative flow cytometry plots of Treg cell infiltration in the spleen.

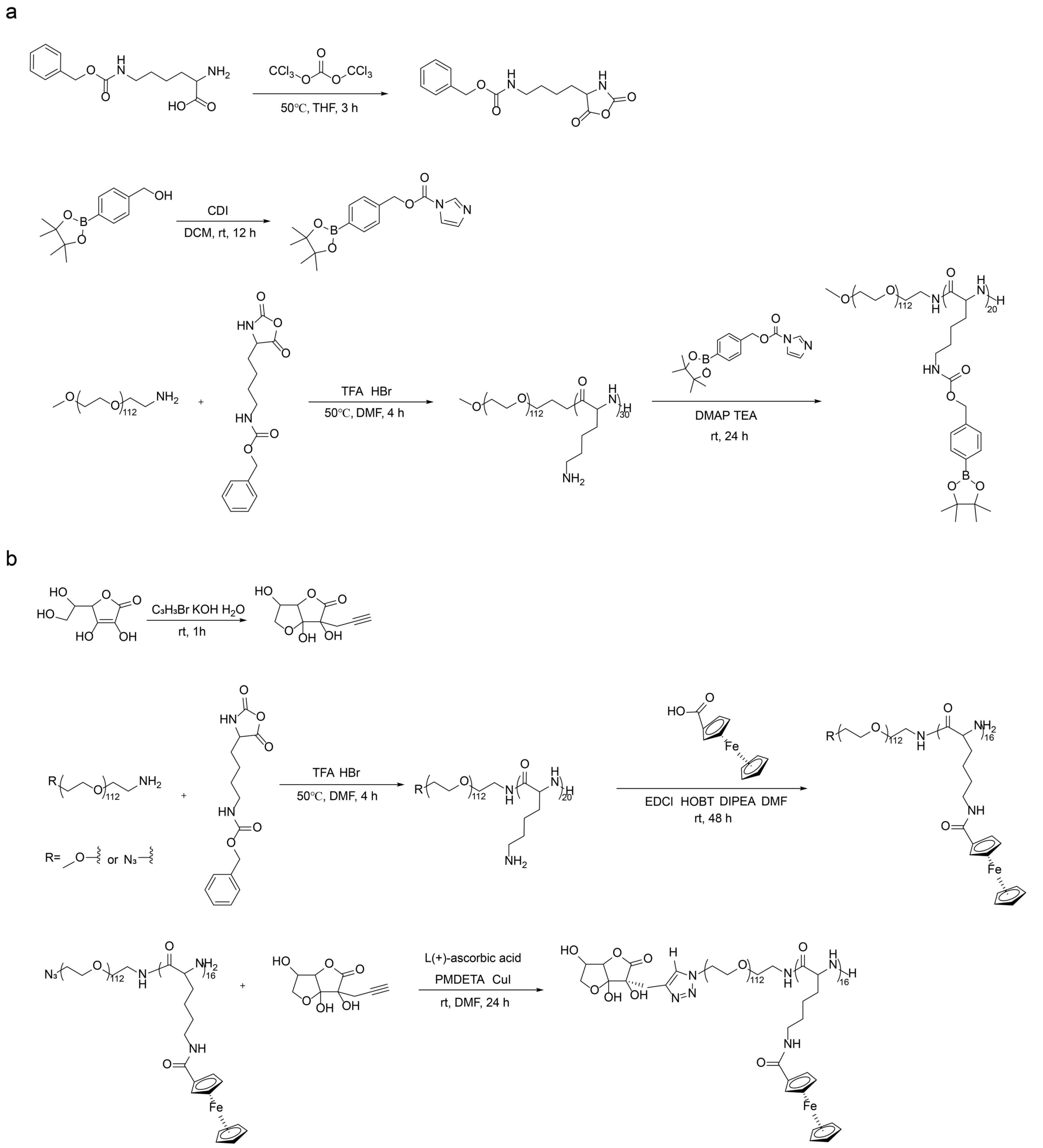

Supplementary Figure 30. Synthetic routes of polymeric backbones. (a) Synthetic procedure for PB-polymer precursor; (b) Synthetic procedure of Fc-polymer and DHA-modified Fc-polymer.

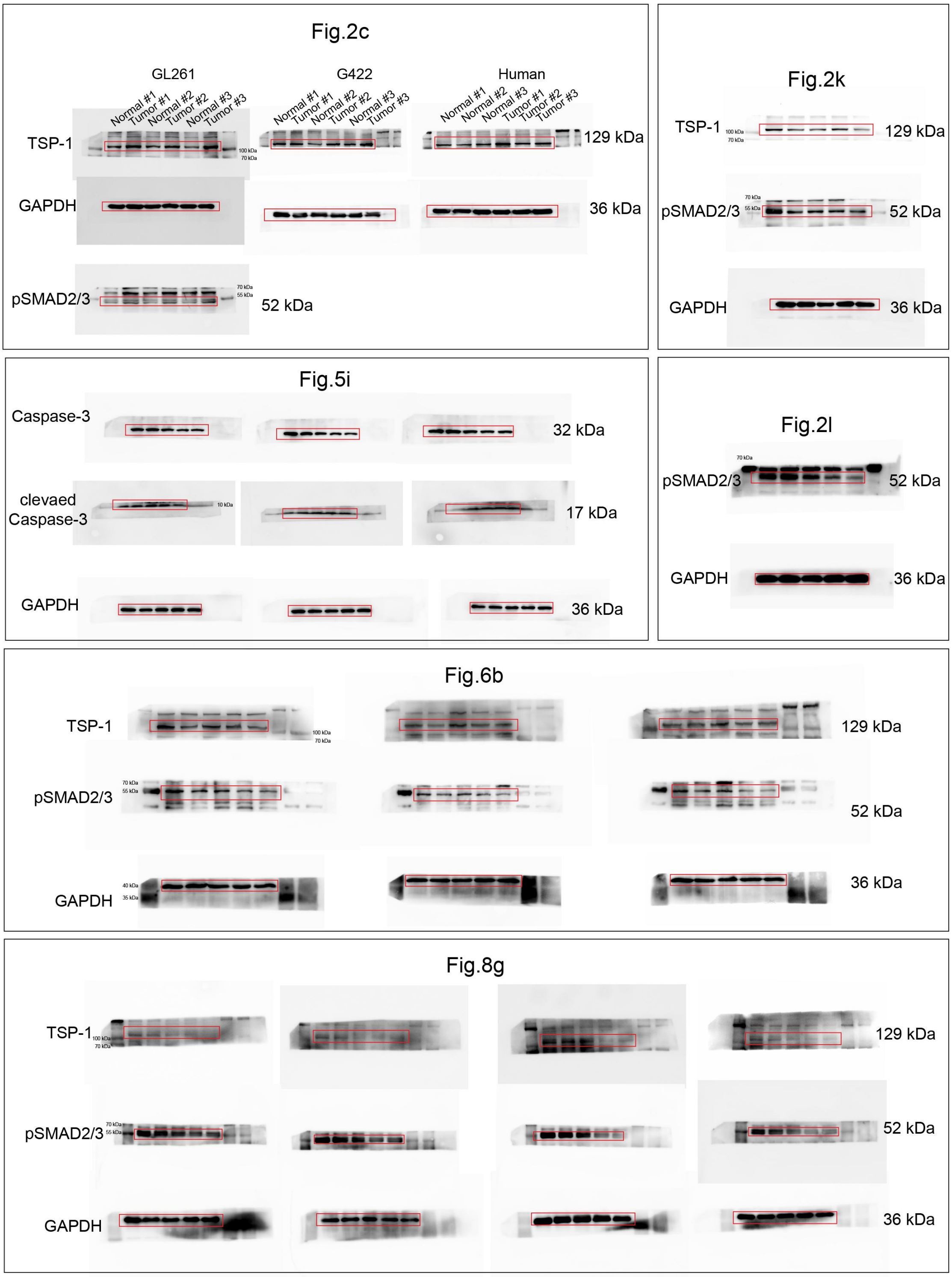

Supplementary Figure 31. Uncropped images of all blots shown in main and Supplementary Figures.
