# Supplementary material for: ROS-autocatalytic nanoplatform interfering astrocyte-cancer crosstalk and enhancing glioblastoma therapy
Source: Nat Commun. 2026 Sep 10;17:10672. doi: 10.1038/s41467-026-77395-2 (PMC13655175; doi:10.1038/s41467-026-77395-2)
Supplement: Supplementary file 3 [file 41467_2026_77395_MOESM3_ESM.pdf]

nature portfolio

Peer Review File

ROS-Autocatalytic Nanoplatform Interfering Astrocyte-Cancer Crosstalk and Enhancing Glioblastoma Therapy

Corresponding Author: Professor Tao Sun

This file contains all reviewer reports in order by version, followed by all author rebuttals in order by version.

Version 0:

Reviewer comments:

Reviewer #1

(Remarks to the Author)
REVIEWER COMMENTS

The manuscript by Yun Chen, et al. aimed to exhibit a novel BBB-penetrating nanoparticle for GBM therapeutic drugs delivery, with comprehensive framework and appreciable work load. In this manuscript, the authors select DHA-Glut1 as a potential bridge, to locate tumor-killing S-Gboxin and TGF-β pathway inhibitor Galunisertib from serum to brain. However, there are still some concerns and questions to answer for publication.

Major Concerns:
1. Delivery efficiency and in vivo toxicity data are of great importance for a nanoparticle delivery system. The authors shall provide: 1) detailed in vivo drug distribution information among major tissues at different time point, and 2) biological half-time data, especially those of drugs in cerebrospinal fluid. Besides, a series of toxicity index like organ injury information and maximum toleration dosage shall be offered and precisely described in the manuscript.
2. High Glut1 protein expression in GBM tumor cells needs further validation as a experimentally direct comparison between tumor and normal tissues or between GBM cell lines and astrocytes is missing. Besides, whether Glut1 is a convincing carrier remains questionable, because, firstly, authors does not provide sufficient evidence to support the Glut1-dependency of BBB penetration observed in this article while BBB-penetrating efficiency accelerated by DHA is limited according to fig. 4 to fig. 7, and secondly, Glut1 is ubiquitously expressed in various tissues to transport glucose, which means these tissues might also consume your nanoparticles. The authors are encouraged to discussion advantages of selecting Glut1 rather than other highly expressed membrane proteins in GBM.
3. According to fig. 5b, Galunisertib itself does not present obvious growth suppression effect and the tumor-killing effect of S-Gboxin through OXPHOS inhibition is not amplified by a further suppression of mitochondria transfer, no matter using the nanoparticle delivery system or not. So what is the necessity of including Galunisertib?
4. Many experimental details are missing, and as a result, a lot of cross-group comparison, like those in fig. 3, fig. 5 and fig. 6, can not result in solid conclusions. The authors need to provide experimental details, including dosage, treatment duration, etc. in both figure legend and methods, and re-modify descriptions in the manuscript.

Minor Comments:
1. Authors shall provide figure files with high resolution. A lot of images in the manuscript, especially those of immunofluorescence assays, are so blurred to tell details. For example, fig.2f can not prove the fluorescence signal is mitochondrial, in terms of smeared localization.
2. Please point out the tubular structures referred to transfer mitochondria in fig. 2e.
3. According to fig. 3i, S-Gboxin sole treatment also generates ROS although being much lower than GS@FP and GS@DFP, but it is not presented in statistic data, fig. 3i.
4. The so-called tubular structures in fig. 6g are much more like junctions between cells and authors are encouraged to give direct evidences to prove they are MT connections.
5. The n number (i.e. the sample size used to derive statistics) should be provided and defined as a precise value (not a range), using the wording “n=X samples/cells/independent experiments” etc. where applicable.

Reviewer #2

(Remarks to the Author)
This manuscript presents a multifunctional ROS-responsive nanoplatform (GS@DFP) designed to simultaneously disrupt intrinsic tumor mitochondrial function and astrocyte-to-glioblastoma mitochondrial transfer. By integrating DHA-mediated BBB penetration, ferrocene-driven ROS amplification, mitochondrial ATP synthase inhibition (S-Gboxin), and TGF-β/SMAD/TSP-1 pathway blockade (Galunisertib), the authors address a critical and emerging mechanism of GBM metabolic adaptation and therapy resistance.
The study is comprehensive, technically rigorous, and conceptually innovative. The combination of advanced nanomaterial engineering with mechanistic insights into tumor–astrocyte crosstalk and immune remodeling is a major strength. However, some mechanistic clarifications are required to fully support the conclusions and enhance translational relevance.
• The authors attribute enhanced BBB penetration and tumor targeting primarily to DHA-mediated Glut1 transport. While supportive in vitro and in vivo evidence is provided, direct mechanistic validation remains incomplete: it is unclear whether Glut1 is strictly required for nanoparticle transport across the BBB; the in vitro BBB model lacks competitive inhibition or transporter-blocking experiments.
• Ferrocene-mediated Fenton-like reactions play a central role in ROS amplification and drug release. However, potential off-target oxidative stress in normal brain tissue is insufficiently addressed: elevated ROS levels may damage neurons or normal astrocytes, especially given the brain’s vulnerability to oxidative stress.
• The manuscript emphasizes suppression of astrocyte-derived mitochondrial support but does not clearly distinguish between functional inhibition and direct astrocyte damage. It remains unclear whether GS@DFP selectively inhibits mitochondrial transfer mechanisms or induces astrocyte dysfunction/cytotoxicity.
• The authors attribute immune activation primarily to TGF-β pathway inhibition. However, mitochondrial disruption and ROS generation may also contribute to immune remodeling.
• The survival extension observed in GS@DFP-treated mice is notable; however, the survival curves show partial, not complete, tumor control: in this regard, claims such as “paralyzing the GBM mitochondrial network” may overstate the therapeutic effect.

Reviewer #3

(Remarks to the Author)
In this study, the authors designed a nanoparticle-based dehydroascorbic acid (DHA)-functionalized, ROS-responsive, ferrocene-integrated polymer nanoplatform (GS@DFP) co-loaded with S-Gboxin and Galunisertib, aiming to simultaneously target GBM mitochondria and astrocyte–cancer cell mitochondrial transfer. This is a translational study and focuses on evaluating the therapeutic efficacy of the proposed nanodrug. Authors provides a new combination strategy, and this is the major novelty of this study. Based on authors’ results, the therapeutic effect seems promising. Collectively, this study is promising and potentially important in developing effective treatment for cancers. However, a major concern for this study is the scientific rational of this work that the authors provided. The rationale is based on very few literatures, which make it looks questionable and is insufficient. Based on current study, it is difficult to comprehend that the promising therapeutic effect of targeting both mechanisms (GBM mitochondria and astrocyte–cancer cell mitochondrial transfer) can be attirbuted to the very reason claimed by authors. It is possible that the underlying mechanisms of two mechanisms are totally irrelevant or other mechanisms may also be involved. To support or strengthen the hypothesis and conclusion claimed by the authors, strong and convincing evidence/data are needed. Additionally, the scientific rigor needs to be improved. The current enthusiasm for this manuscript is moderate.
Major Comments:
1. The major concern from this reviewer is the rationale for this study. Here are the detailed comments.
(1) Reference 8 is an abstract from a meeting without a manuscript in fall length and a peer review, which is inappropriate as key evidence for its scientific premise.
(2) In lines 50–53, the authors claim that S-Gboxin fails to block astrocyte-derived mitochondrial compensation, but they did not provide convincing evidence or appropriate reference. One would presume that the conclusion is based on references 10 or 11. Studies in these two reports are insufficient to support for this conclusion. It is imperative that the authors provide direct evidence or supporting references. Moreover, it is also questionable that an ATP synthase inhibitor would be unable to suppress astrocyte-derived mitochondria and mitochondrial compensation.
(3) Only single publication is used to support the role of TGF-β in regulating tumor microtubes and mitochondrial transfer, which is insufficient to establish a strong rationale. Given the well-known roles of TGF-β in immunology and many other biological processes, regarding mitochondrial transfer as the main effect of TGF-β signaling and using it as a central rationale is not convincing. In addition, in line 55, reference 12 only reports the use of a TGF-β pathway inhibitor but does not examine mitochondrial transfer phenotypes. Overall, the involvement of the TGF-β pathway in mitochondrial transfer is insufficiently supported and the data of mitochondrial transfer inhibition is the primary effect of TGF-β pathway inhibitors needs additional supporting evidence. It remains possible that the major therapeutic effect observed in this study is unrelated to mitochondrial transfer.
(4) The authors combine multiple elements into a single drug design targeting different pathways. The rationale for this combination is weak. It is questionable about whether inhibition of mitochondrial transfer is indeed a key downstream effect of TGF-β inhibition, and whether the biological effects of inhibiting these two pathways (GBM mitochondria and astrocyte-cancer cell mitochondrial transfer) are mechanistically related. The authors should provide clear and supporting evidence demonstrating a true synergistic effect of simultaneously targeting GBM mitochondria and astrocyte-mediated mitochondrial

transfer. In addition, they should show that the effects observed with single agents and their combination are on-target rather than the result of off-target effects.
2. The conclusions are based on only a single model, which is not convincing. GBM is well known for its high degree of heterogeneity, and at least one additional model is required, at least in key data.
3. Typically, figures are presented in numerical order. Starting the Results section with Figure 2 is not very common.
4. Immunoblot images throughout the manuscript should include sufficient surrounding background and should not be cropped too narrowly.
5. In line 51, comparing the IC50 values of S-Gboxin and TMZ in a mouse cell line is confusing and does not provide meaningful information.
6. In Figures 5i and 5j, the labels are incorrect. Several panels in this part are mislabeled and confusing. In Figures 5k–5m and the corresponding Results section, the inclusion of TMZ appears irrelevant to this study. The rationale for introducing TMZ at this point is confusing. In addition, in line 265, “GS@Defficiently” appears to be a typographical error. This section contains too many mistakes and is extremely confusing. Additionally, in Figure 2m, for the statistical analysis of Ki-67, it is unclear whether the y-axis represents percentages or absolute numbers.
7. Throughout the manuscript, the colors used in the figures are very similar, making it difficult to distinguish different groups. The authors should also clearly indicate what each bar represents, rather than relying on a single legend or representative description across whole figure. This issue is particularly severe in Figures 6 and 7.
8. In Figure 7, the effects of the authors’ drug and TMZ are not directly comparable. Based on the data presented, the authors’ conclusion that their drug is better than TMZ is not well supported. The dosing across different groups is not comparable. In addition, sensitivity of cancer cells to TMZ varies dramatically among different cell types Therefore the drug presented in this study is an inappropriate candidate.
9. Mitochondrial function is known critical for the immune microenvironment, including T cells and other immune populations. Thus, mitochondrial inhibition may impair antitumor immune responses or other tumor-suppressive factors. The Discussion section should consider effects of both sides.

Minor comments:
1. Figure 5b: missing a “)”.
2. Line 331: misuse of spacing.

Version 1:

Reviewer comments:

Reviewer #1

(Remarks to the Author)
All comments raised have been addressed. No more questions.

Reviewer #2

(Remarks to the Author)
Comments have been properly addressed, with additional experiments and evidence.

Reviewer #3

(Remarks to the Author)
In this revised manuscript, the authors have addressed all my comments with revised figure panels as well text revisions. The revised manuscript is now sufficient for its further consideration at Nature Communications.

Open Access This Peer Review File is licensed under a Creative Commons Attribution 4.0 International License, which permits use, sharing, adaptation, distribution and reproduction in any medium or format, as long as you give appropriate credit to the original author(s) and the source, provide a link to the Creative Commons license, and indicate if changes were made.
In cases where reviewers are anonymous, credit should be given to 'Anonymous Referee' and the source.
The images or other third party material in this Peer Review File are included in the article's Creative Commons license, unless indicated otherwise in a credit line to the material. If material is not included in the article's Creative Commons license and your intended use is not permitted by statutory regulation or exceeds the permitted use, you will need to obtain permission directly from the copyright holder.
To view a copy of this license, visit https://creativecommons.org/licenses/by/4.0/

Point-by-point Response to the Reviewers' Comments

Manuscript ID: [**NCOMMS-25-100174**]

Dear Editor and Reviewers:

We hope this message finds you well. We sincerely thank you for the careful evaluation of our manuscript entitled "**ROS-Autocatalytic Nanoplatform for Targeting Intervention in Astrocyte-Cancer Crosstalk Enhances Glioblastoma Therapy**" (Manuscript ID: **NCOMMS-25-100174**). We respectfully submit this point by point response for reconsideration, as we believe that the reviewers' concerns mainly relate to mechanistic clarification and experimental completeness, rather than reflecting a fundamental conceptual weakness of the study.

A List of Changes Made

In response to the valuable comments from the three reviewers, we have provided comprehensive and systematic revisions. To facilitate the review process:

1) In the **point-by-point response**, reviewers' comments are highlighted in blue. Newly added experimental results are labeled as Figure RX and further emphasized with yellow highlighting. References to existing experimental data are indicated in **bold red font**, while citations of original manuscript text are marked in red. Revisions made to the manuscript are shown in green.

2) In the **Revised Manuscript**, we have updated the main text, the discussion section and other areas that need to be modified, including clarification of the exact meaning of sample sizes used for statistical analysis. As above, original text references are marked in red, and revised content is indicated in green.

3) In the **Revised Supporting Information**, the same color-coding scheme has been applied: original text references are shown in red, and revised content in green.

Specifically, we have made the following improvements:

Response to Reviewer #1

We would like to thank reviewer #1 for the suggestions. We fully agree that in vivo biodistribution, pharmacokinetics, and safety evaluation are essential for

nanomedicine strategies. Accordingly, we have supplemented the revised manuscript with data on the in vivo half-life and biodistribution of the formulation, along with a systematic safety assessment. Following the reviewer's suggestion, we further analyzed Glut1 expression at the cellular, tissue, and human tumor sample levels to strengthen the theoretical basis for Glut1-mediated targeting. In addition, we conducted Glut1 inhibition and glucose competition experiments to functionally validate its role in the targeting strategy. Based on these findings, we have revised the relevant descriptions to ensure scientific rigor and accuracy. All other comments from the reviewer have also been addressed in detail.

Response to Reviewer #2

We thank Reviewer #2 for the insightful comments regarding transporter involvement, ROS-related side effects, astrocyte selectivity, and immune regulation mechanisms. To address these concerns, we performed Glut1 inhibition and glucose competition experiments to validate its functional role in targeting. We also compared ROS responses between tumor cells and normal brain cells following treatment with blank nanoparticles, demonstrating that ROS amplification predominantly occurs in tumor cells, thereby enabling selective effects. In mitochondrial transfer experiments, only tumor cells were pretreated prior to co-culture, minimizing direct exposure of astrocytes to nanoparticles and excluding potential cytotoxic interference from the experimental design. Finally, we have expanded the Discussion to clarify that the observed immune remodeling is likely driven by the combined effects of mitochondrial dysfunction, ROS generation, and TGF-β pathway modulation, rather than a single dominant mechanism.

Response to Reviewer #3

We thank Reviewer #3 for the critical evaluation and agree that conference abstracts should not be used as key evidence. For this purpose, we have updated the references. Given the limited research on mitochondrial transfer in GBM, we established a systematically validated experimental framework, including pathway analysis,

visualization of tumor microtube structures, molecular regulation of associated proteins, and direct assessment of astrocyte-to-tumor mitochondrial transfer along with its impact on cell cycle progression, forming a coherent and internally validated evidence chain.

We clarify that TGF-β signaling represents a pharmacologically actionable and disease-relevant regulatory axis within this process, rather than the sole determinant. Compared with directly targeting structural components such as GAP43 or actin, intervention at the aberrantly activated TGF-β pathway provides greater disease specificity and reduces potential off-target risks.

To further support this, we supplemented mitochondrial rescue experiments and CCCP pretreatment of astrocytes, and additionally validated the inhibitory effect of Galunisertib on mitochondrial transfer in the G422 model. These results collectively demonstrate the feasibility of modulating mitochondrial transfer through this pathway across multiple models. Regarding synergistic effects and potential off-target concerns, both our original and newly added data consistently support coordinated therapeutic effects and mechanism-driven, target-specific activity. In summary, our central hypothesis is supported by systematic experimental validation rather than inference from limited literature, and remains well justified in the revised manuscript.

Reviewer #1

The manuscript by Yun Chen, et al. aimed to exhibit a novel BBB-penetrating nanoparticle for GBM therapeutic drugs delivery, with comprehensive framework and appreciable work load. In this manuscript, the authors select DHA-Glut1 as a potential bridge, to locate tumor-killing S-Gboxin and TGF-β pathway inhibitor Galunisertib from serum to brain. However, there are still some concerns and questions to answer for publication.

Major Concerns:

1. Delivery efficiency and in vivo toxicity data are of great importance for a nanoparticle delivery system. The authors shall provide: 1) detailed in vivo drug distribution information among major tissues at different time point, and 2) biological half-time data, especially those of drugs in cerebrospinal fluid. Besides, a series of toxicity index like organ injury information and maximum toleration dosage shall be offered and precisely described in the manuscript.

Response: Thank you to the reviewer for this critical and constructive comment. In accordance with the reviewer's suggestion, we have systematically supplemented the revised manuscript with comprehensive in vivo evaluations of delivery efficiency, pharmacokinetic behavior, and biosafety. These newly added experiments are thoroughly presented.

(1) Pharmacokinetics and biological half-life

In response to the reviewer's concern regarding the half-lives of the two drugs, and to comprehensively evaluate delivery efficiency, we divided the treatment groups into three categories: free dual-drug group, non-targeted GS@FP group, and targeted GS@DFP group. The equivalent doses of the two drugs were S-Gboxin (3 mg/kg) and Galunisertib (5 mg/kg). We then conducted systematic pharmacokinetic studies by measuring plasma drug concentrations over time and performing model fitting to obtain relevant pharmacokinetic parameters.

The results showed that, for S-Gboxin, formulation significantly extended its elimination half-life from 1.9 h (free drug) to approximately 6.6 h, representing an increase of about 350%. For Galunisertib, formulation extended its elimination

half-life from 3.8 h (free drug) to approximately 10.2 h, corresponding to an increase of about 315%.

Taken together, these results demonstrate that the delivery system significantly prolongs the half-life of both drugs in systemic circulation and cerebrospinal fluid, enhances their stability, and further supports its advantage in brain-targeted delivery.

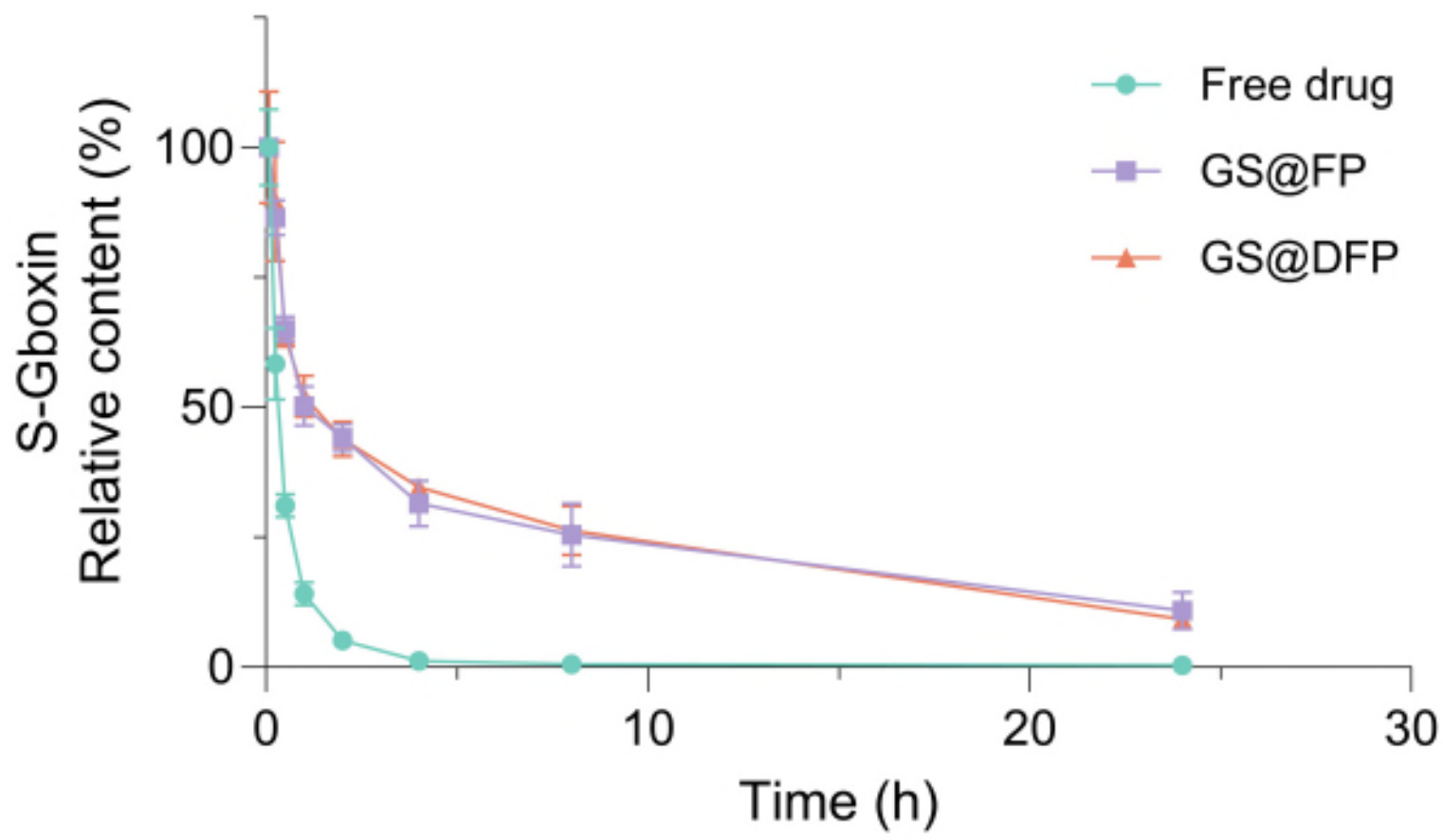

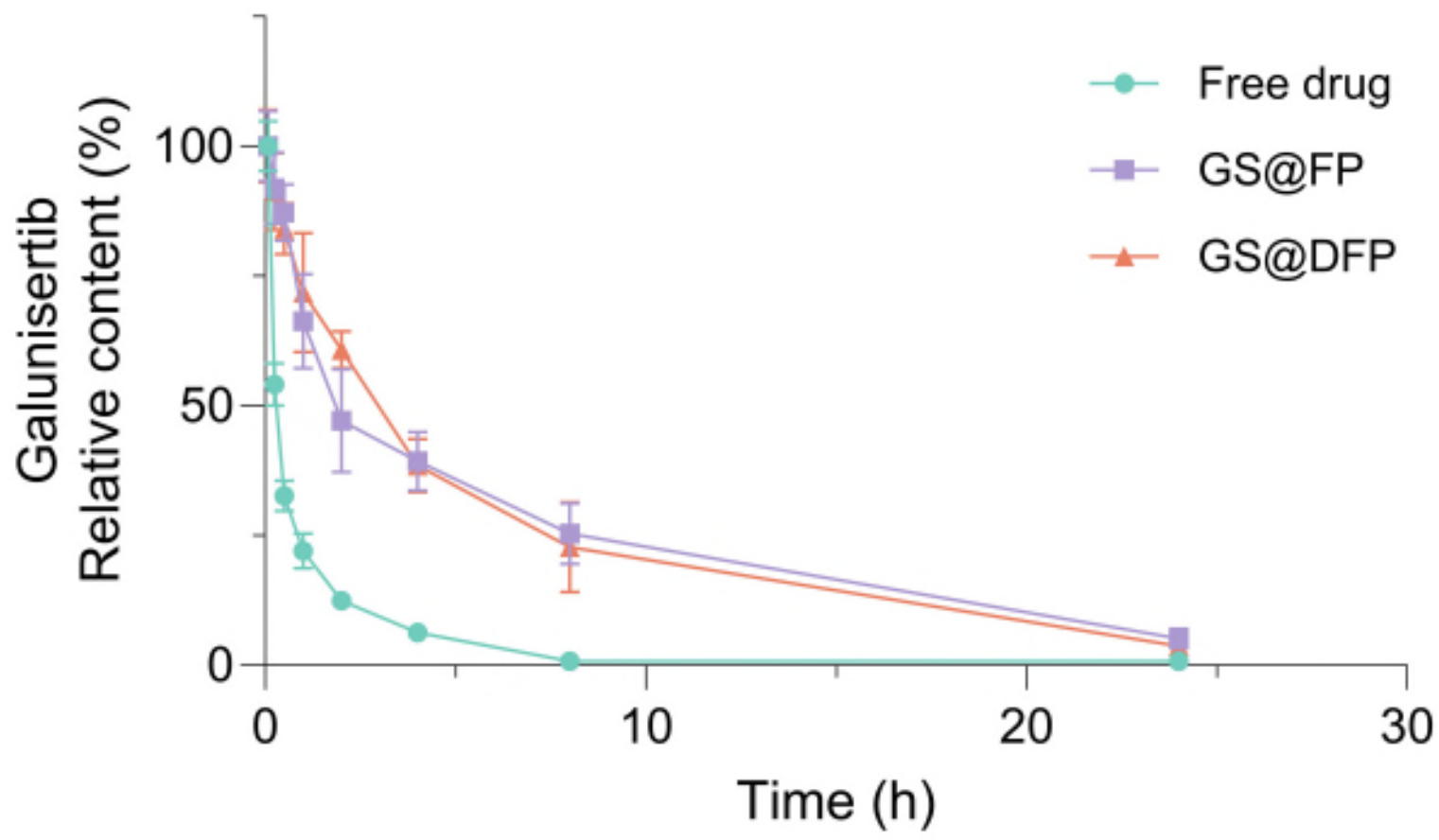

Figure R1. Relative plasma concentration profiles of S-Gboxin and Galunisertib over 24 h under different treatment groups (n = 3 independent samples). The equivalent doses of the two drugs were set as follows: S-Gboxin: 3 mg/kg; Galunisertib: 5 mg/kg.

(2) In vivo biodistribution study

In response to the reviewer’s concerns regarding in vivo distribution, and to comprehensively evaluate distribution differences, we divided the treatment groups

into three categories: a free dual-drug group, a non-targeted group (GS@FP), and a targeted group (GS@DFP). The equivalent doses of the two drugs were set as follows: S-Gboxin (3 mg/kg) and Galunisertib (5 mg/kg). At 6 h after intravenous administration, the drug distributions in major organs—including brain, liver, spleen, lung, kidney, and tumor tissues—were quantitatively analyzed by LC–MS across different groups, thereby providing an overview of biodistribution profiles.

In addition, based on the subsequently determined elimination half-lives of the two drugs, tumor tissues were collected at 2 h and 24 h after administration to further assess the temporal changes in drug concentrations in the brain.

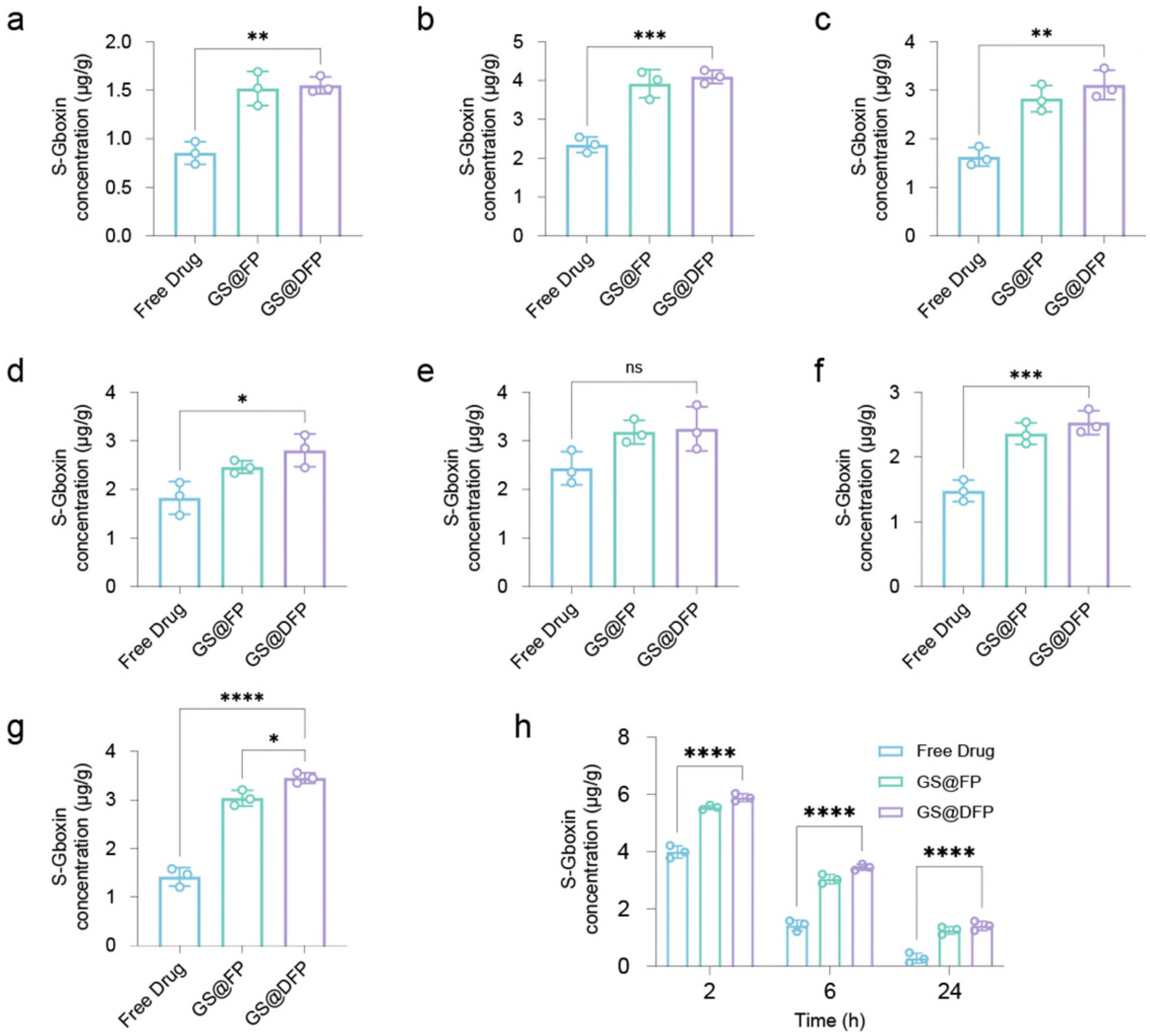

Figure R2. a–g) Distribution of S-Gboxin in major organs—including brain, liver, spleen, lung, kidney, brain, and tumor tissue—under different treatment groups (n = 3 independent samples); h) Time-dependent changes in S-Gboxin concentration in brain tumors following administration of GS@DFP (n = 3 independent samples). *p <

0.05, **p < 0.01, ***p < 0.001, ****p < 0.0001.

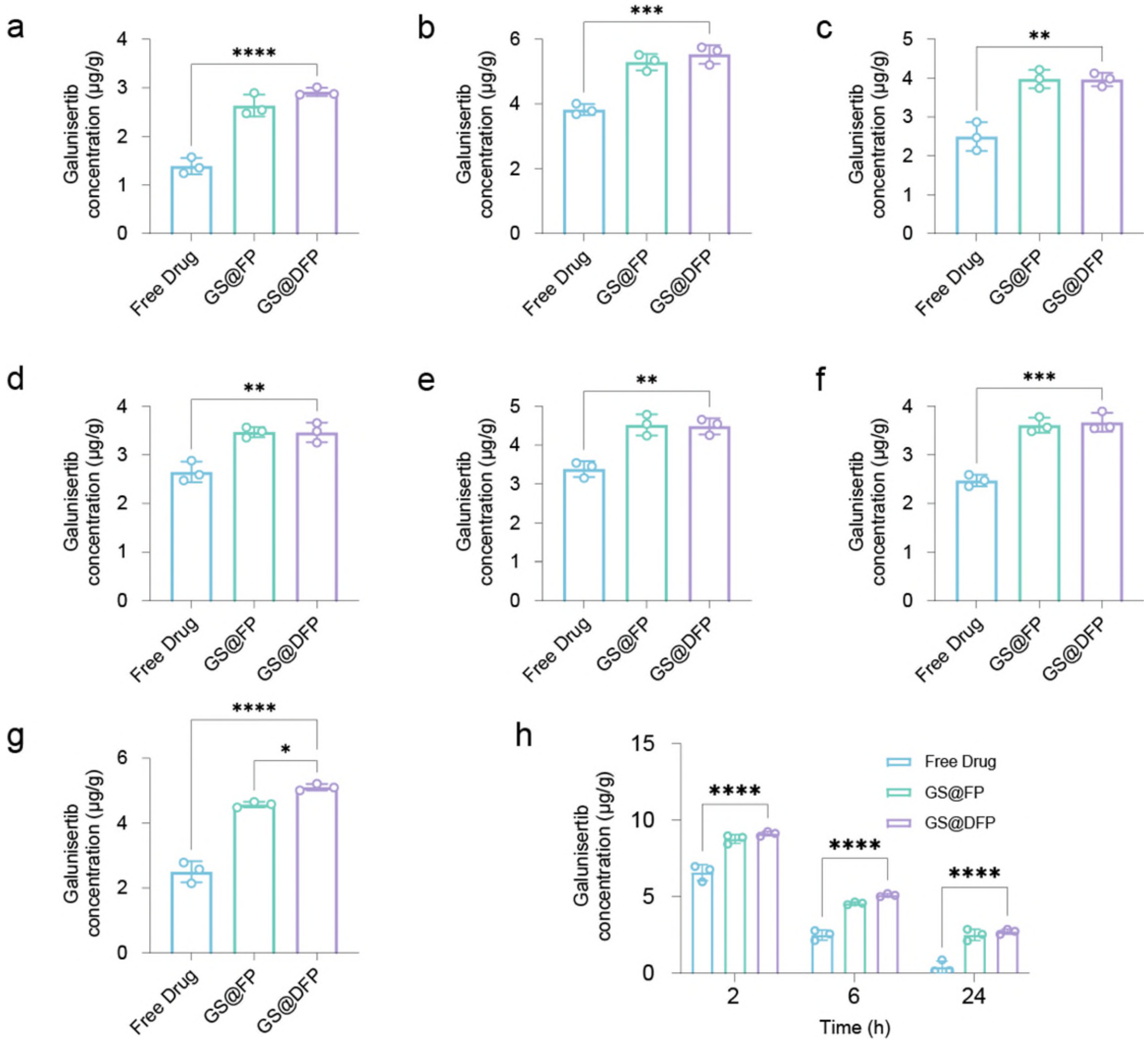

Figure R3. a–g) Distribution of Galunisertib in major organs—including brain, liver, spleen, lung, kidney, and tumor tissues—under different treatment groups (n = 3 independent samples); h) Time-dependent changes in Galunisertib concentration in brain tumors following administration of GS@DFP (n = 3 independent samples). *p < 0.05, **p < 0.01, ***p < 0.001, ****p < 0.0001.

The results showed that, after formulation, both drugs exhibited markedly improved systemic circulation compared with the free drug group. The nanodelivery system demonstrated significantly enhanced accumulation in target tissues, particularly in tumors. At 6 h, the tumor accumulation of the formulated dual-drug system was approximately 2.2-fold higher than that of the free drug group. Meanwhile, nonspecific distribution in non-target organs was reduced.

We also observed that, compared with the non-modified group, the DHA-modified group exhibited relatively increased tumor accumulation of both drugs, directly indicating the important role of DHA modification in enhancing tumor-targeted accumulation. In addition, time-dependent analysis of both formulations showed favorable retention of the drugs at the tumor site after administration, demonstrating the excellent in vivo delivery efficiency and distribution characteristics of this system.

(3) In vivo toxicity and biosafety evaluation (therapeutic dose)

To systematically assess the biosafety of the material at therapeutically relevant doses, we conducted multidimensional toxicity evaluations in the manuscript, including histopathological analysis of major organs via H&E staining, as well as serum biochemical assessments such as ALT, AST, BUN, and CREA. The equivalent doses of the two drugs were set as follows: S-Gboxin (3 mg/kg) and Galunisertib (5 mg/kg).

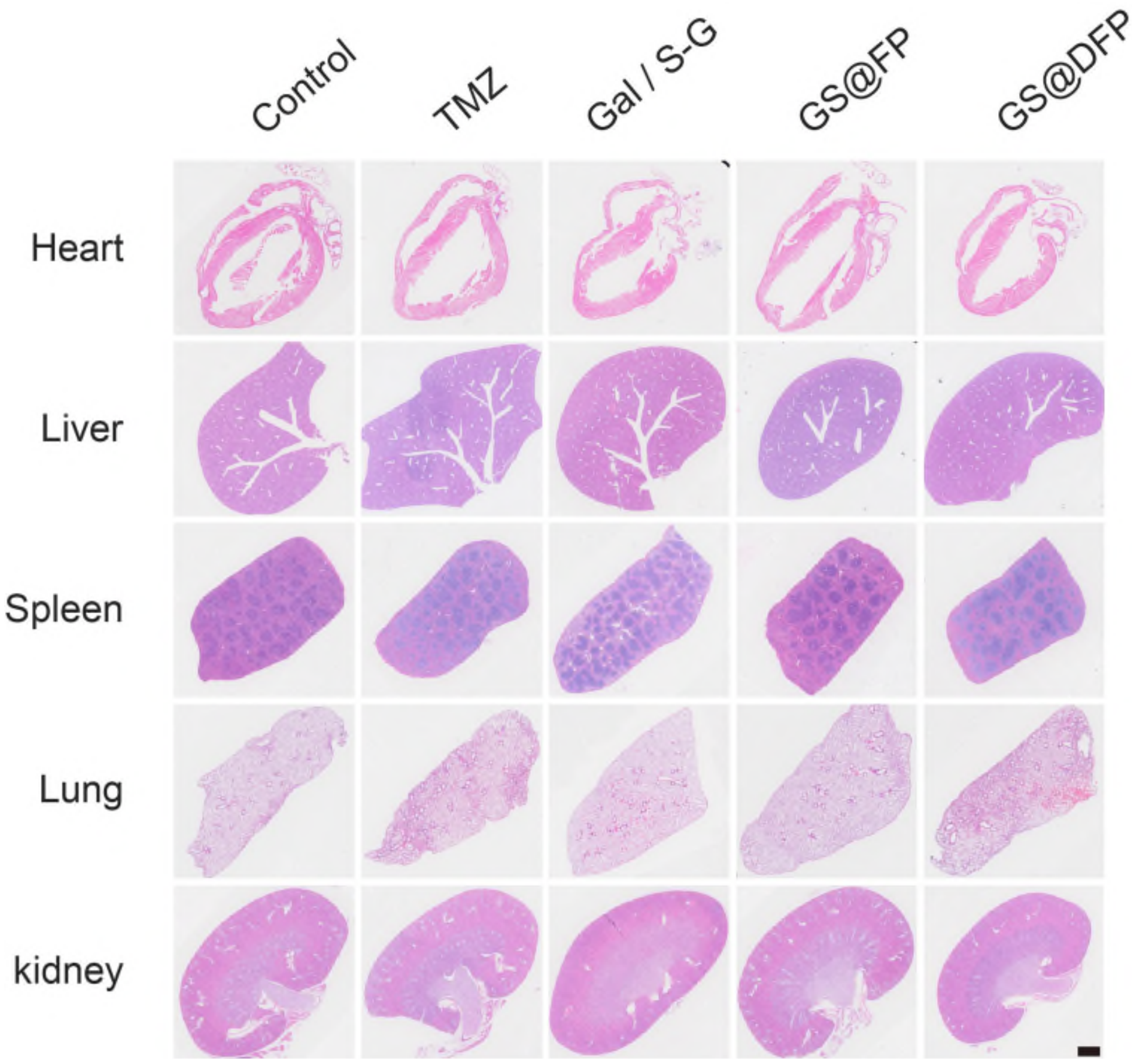

Supplementary Figure 21. H&E-stained sections of major organs from mice in different treatment groups. Scale bar = 1 mm.

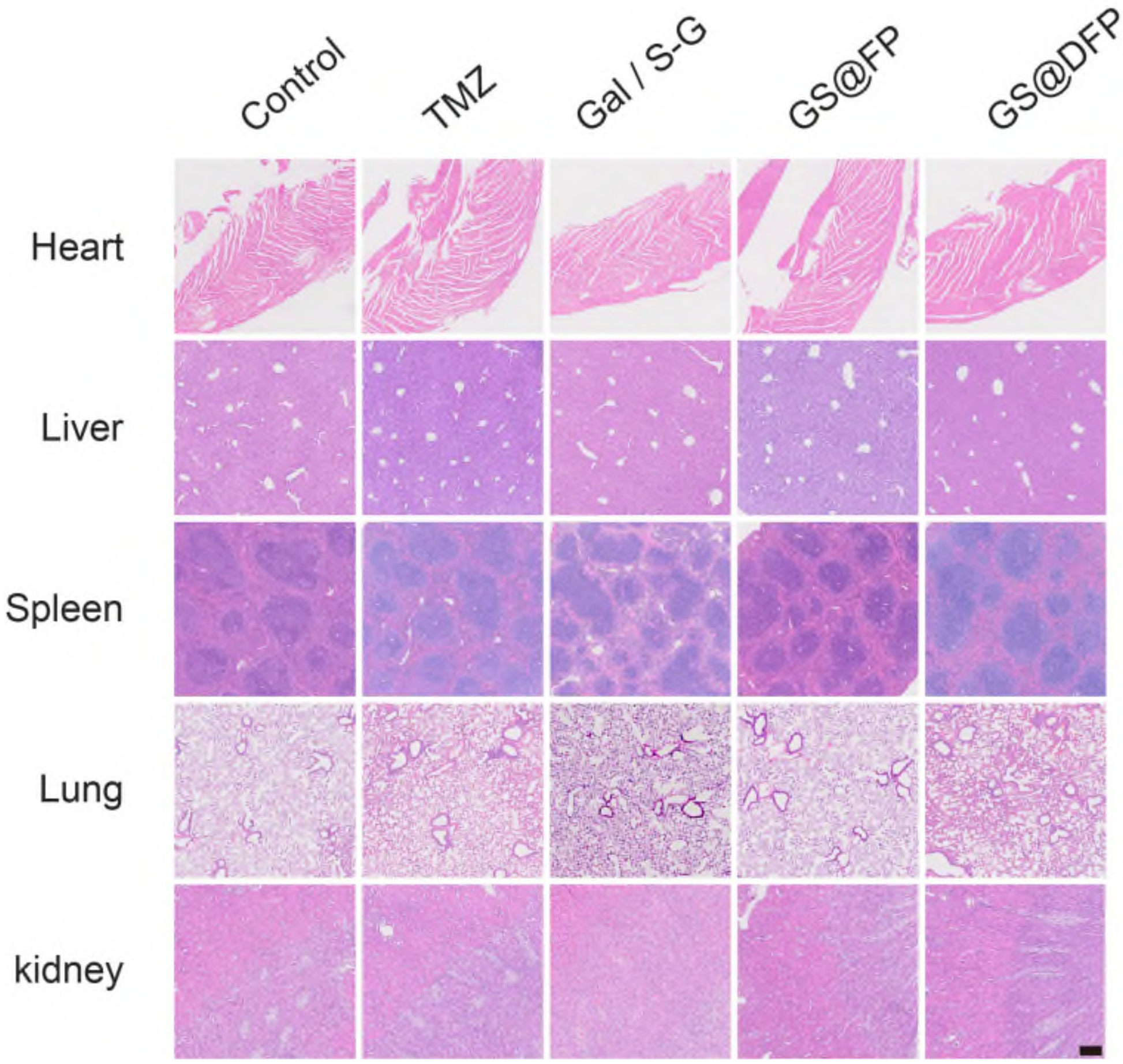

Supplementary Figure 22. Magnified views of H&E-stained sections of major organs from mice in different treatment groups. Scale bar = 200 μm.

The results show that no evident tissue damage, inflammatory infiltration, or abnormal changes in biochemical parameters were observed across all treatment groups (including different therapeutic regimens), indicating that this nanodelivery system exhibits good biocompatibility and safety within the therapeutic dose range.

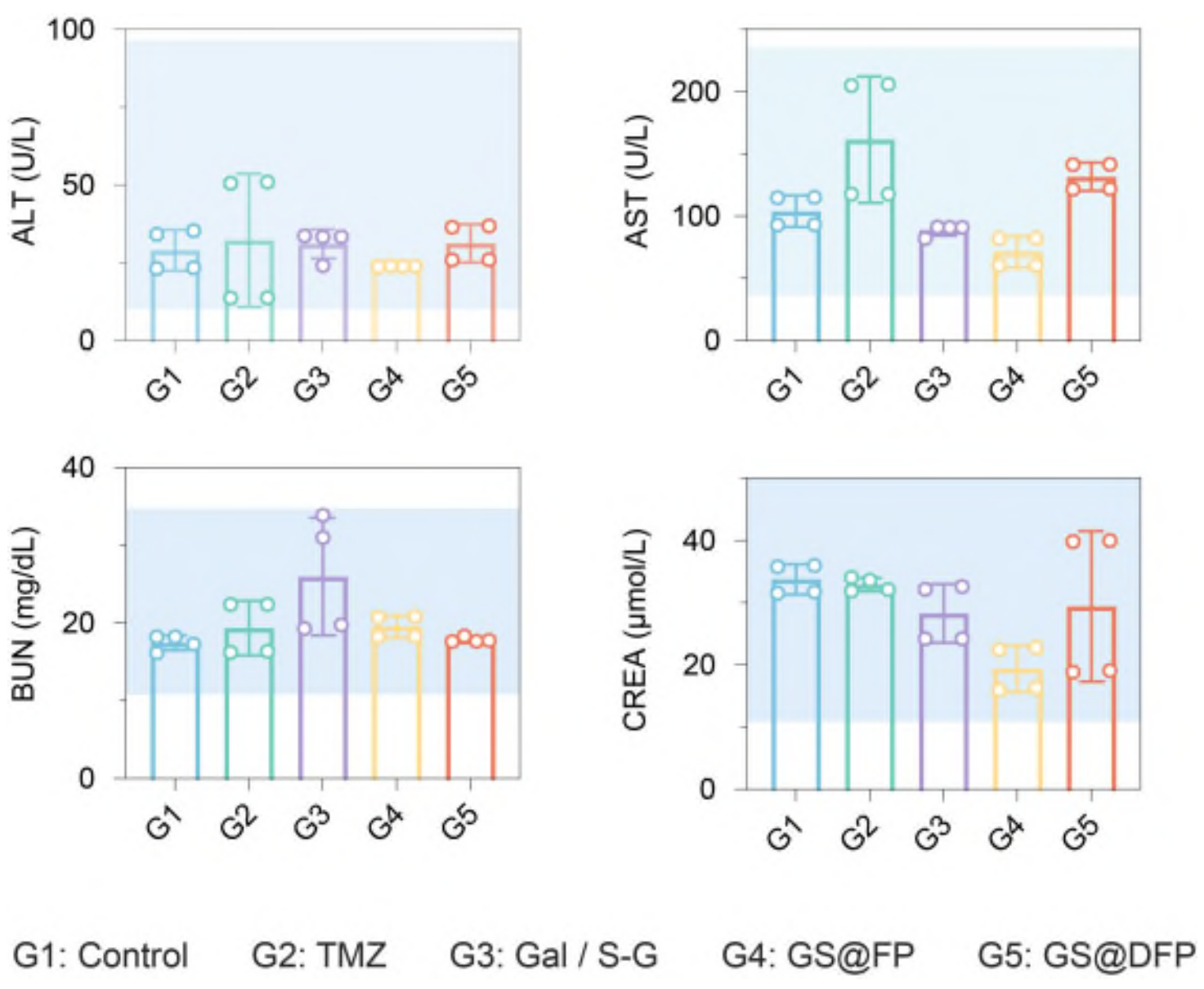

Supplementary Figure 20. Semi-quantitative analysis of ALT, AST, BUN, and CREA levels in the serum of mice from different treatment groups. Blue shading indicates the normal range for each parameter. ALT, alanine aminotransferase; AST, aspartate aminotransferase; CREA, creatinine; BUN, blood urea nitrogen (n=4 independent samples).

(4) Maximum tolerated dose (MTD) evaluation (supratherapeutic doses)

Building upon the aforementioned safety assessments, we further evaluated the maximum tolerated dose (MTD) and the potential toxicity threshold of this system through a dose-escalation study (2 × and 4 × the therapeutic dose), The initial reference equivalent therapeutic doses for the two drugs were set as follows: S-Gboxin (3 mg/kg) and Galunisertib (5 mg/kg).

The results indicate that even at doses exceeding the therapeutic level, the nanoplatform did not induce significant organ damage or severe biochemical abnormalities, and exhibited overall better tolerability than the free-drug counterparts. These findings further demonstrate that the delivery system is not only safe at therapeutic doses but also maintains favorable tolerability over a higher dose range, thereby providing a broader safety window to support its potential clinical application.

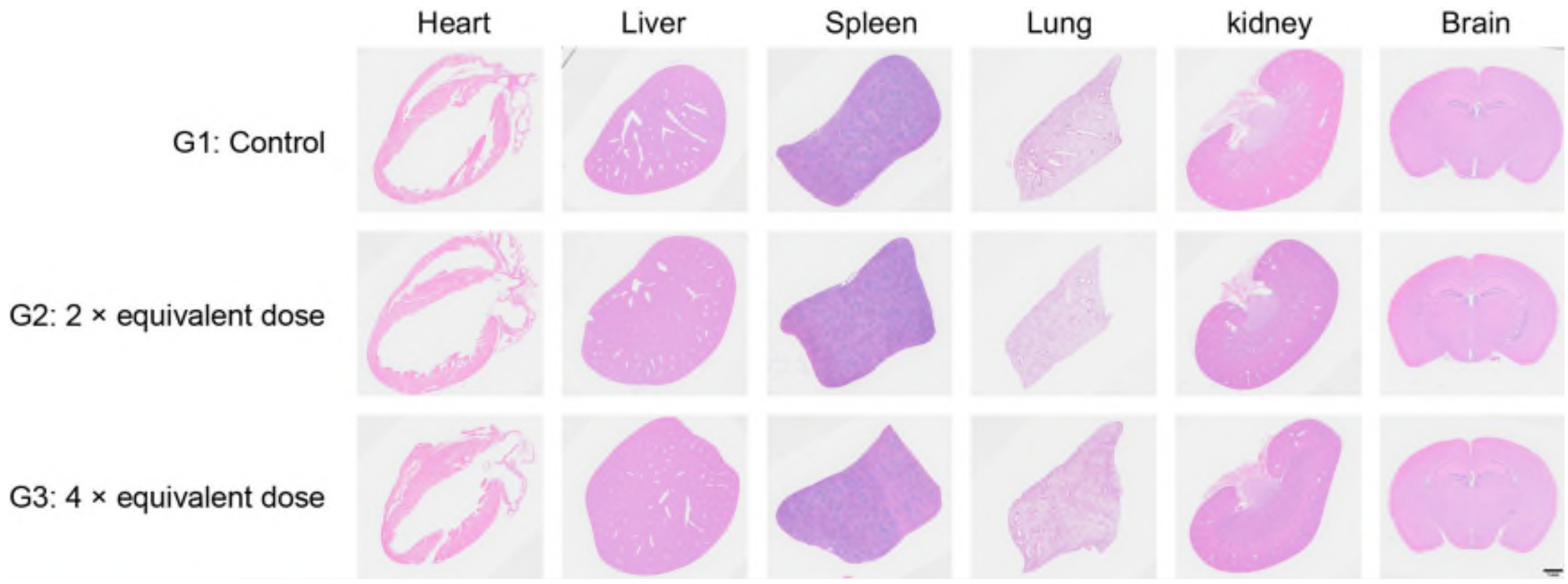

Figure R4. H&E-stained sections of major organs from mice in different treatment groups. Scale bar = 1 mm.

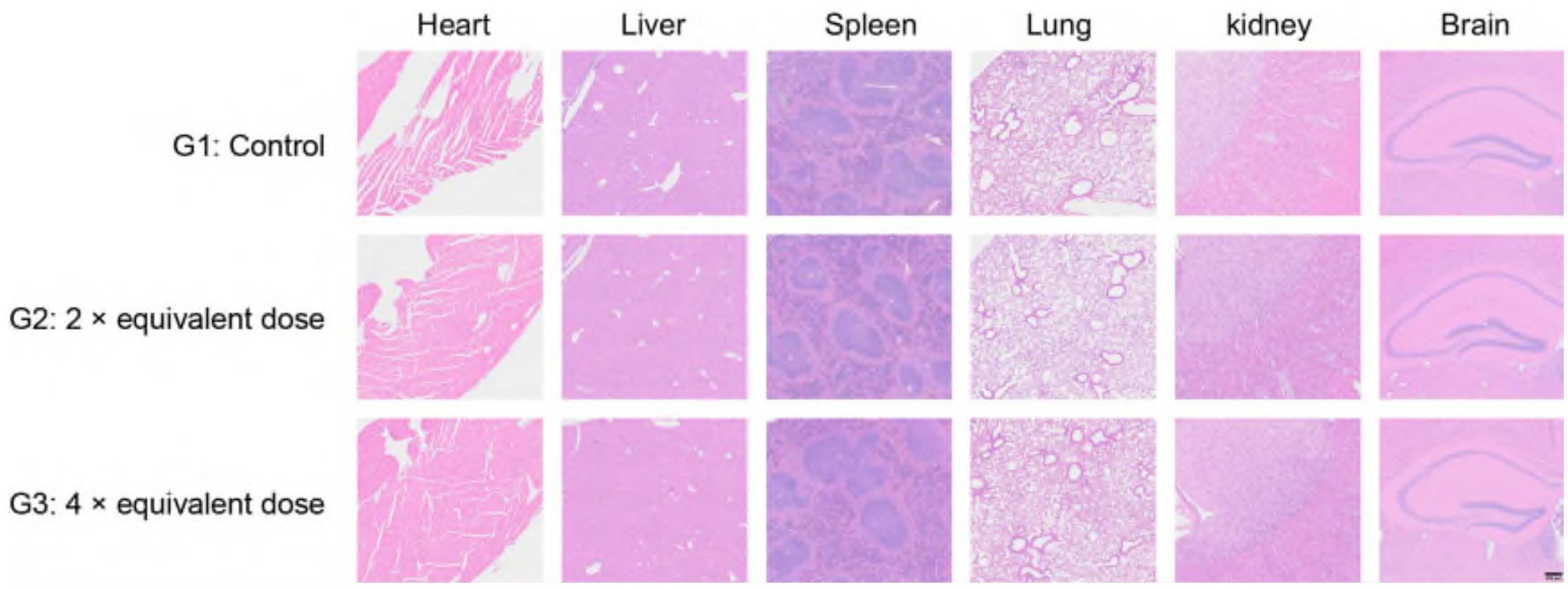

Figure R5. Magnified views of H&E-stained sections of major organs from mice in different treatment groups. Scale bar = 200 µm.

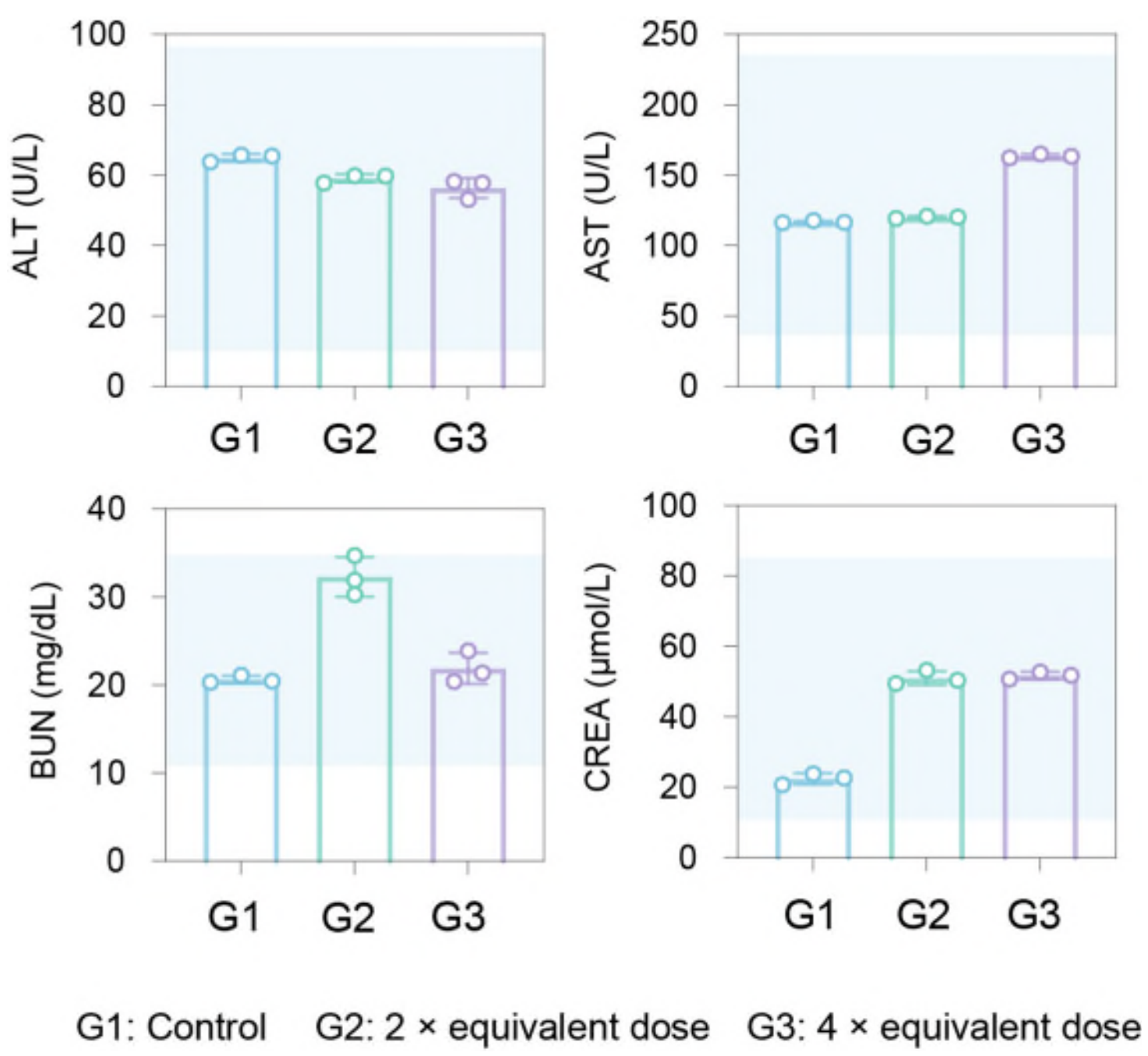

Figure R6. Semi-quantitative analysis of ALT, AST, BUN, and CREA levels in the serum of mice from different treatment groups. Blue shading indicates the normal range for each parameter. ALT, alanine aminotransferase; AST, aspartate aminotransferase; CREA, creatinine; BUN, blood urea nitrogen (n=3 independent samples).

In summary, the supplemented data on in vivo biodistribution, pharmacokinetics, and systemic safety collectively and systematically validate the delivery efficiency and biosafety of this nanodelivery system from multiple perspectives. These additions substantially strengthen the reliability of the study's conclusions and its potential for translational application.

2. High Glut1 protein expression in GBM tumor cells needs further validation as a experimentally direct comparison between tumor and normal tissues or between GBM cell lines and astrocytes is missing. Besides, whether Glut1 is a convincing carrier remains questionable, because, firstly, authors does not provide sufficient evidence to support the Glut1-dependency of BBB penetration observed in this article while BBB-penetrating efficiency accelerated by DHA is limited according to fig. 4 to fig. 7, and secondly, Glut1 is ubiquitously expressed in various tissues to transport glucose, which means these tissues might also consume your nanoparticles. The authors are encouraged to discussion advantages of selecting Glut1 rather than other highly expressed membrane proteins in GBM.

Response: We sincerely thank the reviewer for raising this important question. In response to the concerns regarding insufficient validation of Glut1 expression and the rationale for its use as a delivery target, we have conducted systematic experimental validation and mechanistic analyses in the revised manuscript. The corresponding results are presented in detail in both the main text and the Supplementary Information.

(1) Validation of elevated Glut1 expression in GBM

Following the reviewer's suggestion, we supplemented comparative analyses of Glut1

expression across different biological contexts, including: (i) GL261 cells versus primary astrocytes; (ii) GBM tumor tissues versus normal brain tissues; and (iii) human GBM samples versus corresponding normal brain tissues.

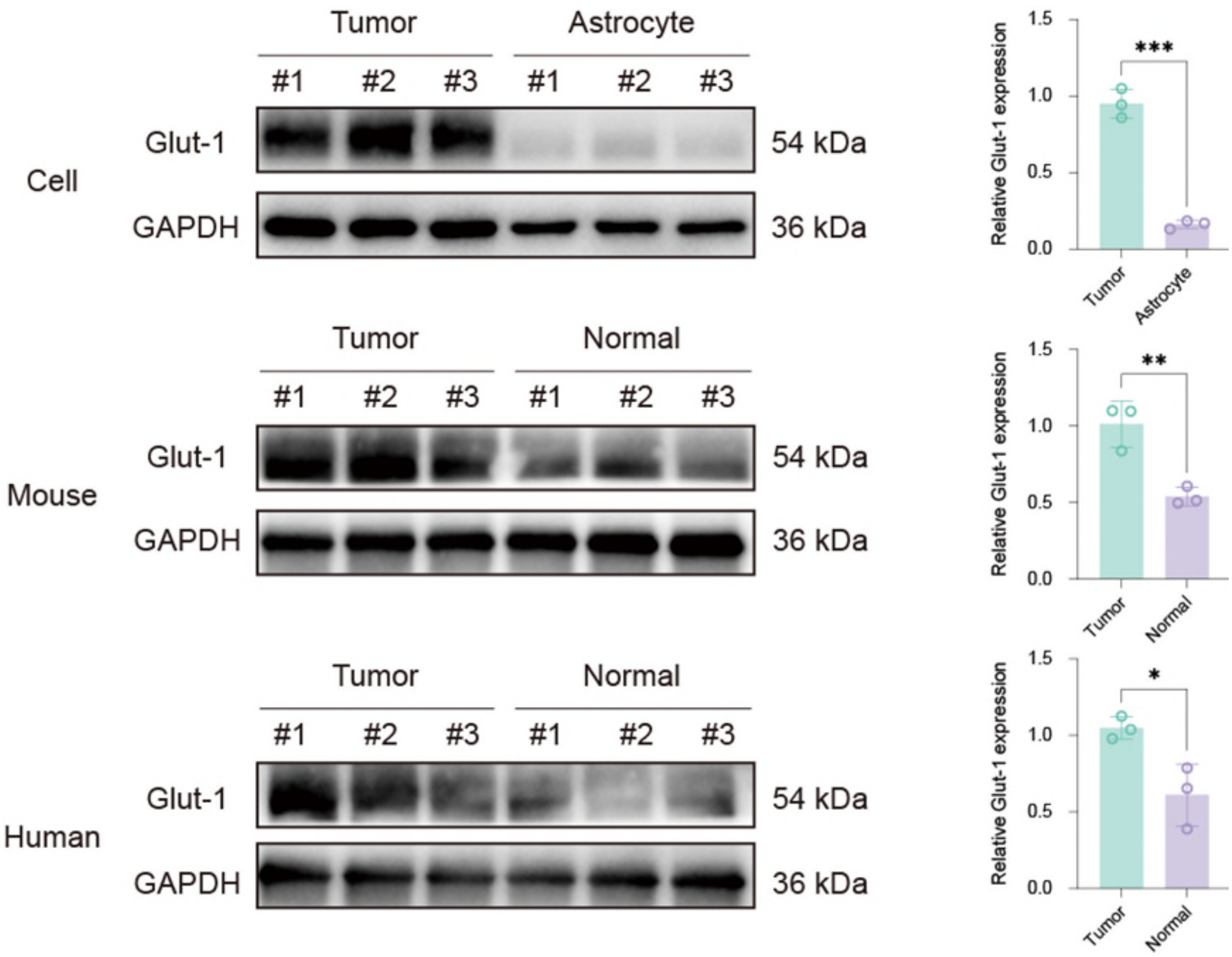

Figure R7. Glut1 expression and corresponding quantitative analysis in GL261 cells versus astrocytes, murine GBM versus normal tissues, and human GBM versus normal tissues (n = 3 independent samples).

The results consistently demonstrate that Glut1 is significantly upregulated in GBM tumor tissues and tumor cells, whereas its expression is relatively low in normal brain tissues and astrocytes. These findings, at both the cellular and tissue levels, support the differential expression pattern of Glut1 in GBM and provide experimental evidence for its suitability as a brain-targeting delivery receptor.

(2) Mechanistic validation of Glut1-mediated BBB penetration

To address the question of whether BBB penetration depends on Glut1, we supplemented mechanistic validation experiments, including Glut1

inhibition/competition studies (using a Glut1 inhibitor as well as excess glucose/DHA for competitive assays). Specifically, bEnd.3 cells were pretreated with the Glut1 inhibitor WZB117 (10 μM) or 50 mM glucose solution for 30 min, followed by assessment of permeability coefficient changes before and after treatment. An in vitro Transwell model was again employed to simulate the BBB, with an extended incubation time in this set of experiments.

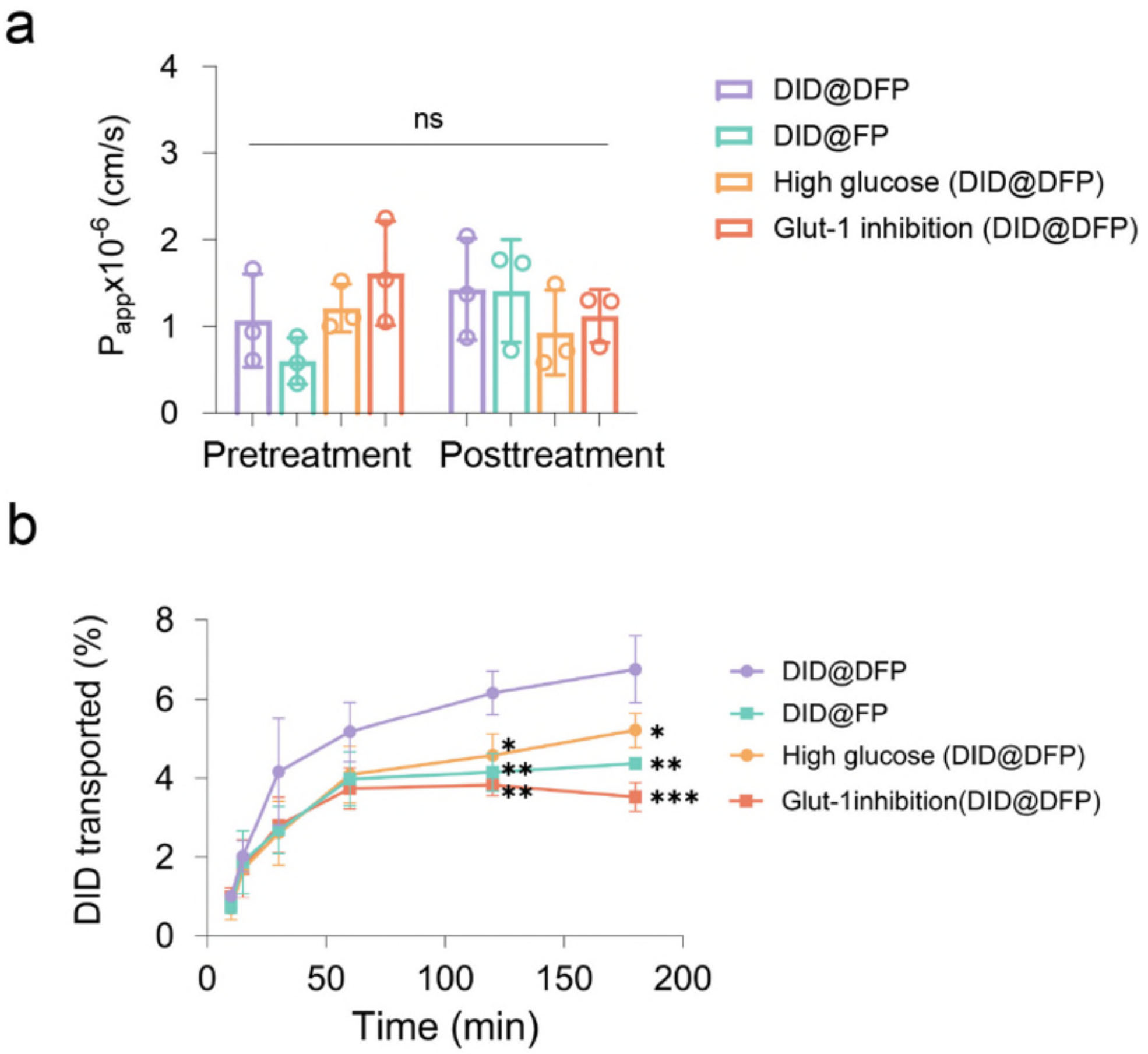

Figure R8. a) Changes in permeability coefficients of bEnd.3 cells before and after various treatments (n = 3 independent samples); b) DiD penetration ratios across different groups (n = 3 independent samples).

The results show that the permeability coefficients of bEnd.3 cells remained essentially unchanged before and after treatment, indicating that neither the formulation nor the pretreatments affected tight junction integrity. Meanwhile, DiD fluorescence detected in the lower chamber demonstrated that, upon Glut1 inhibition,

the trans-BBB transport efficiency of the nanoplatform decreased significantly by approximately 50%, suggesting that BBB crossing is, to a certain extent, dependent on Glut1-mediated transport. In addition, the presence of excess glucose reduced transport efficiency by approximately 25%, indicating that glucose competition for Glut1 partially interferes with DHA-assisted transport. Furthermore, we again observed that, compared with the unmodified group, DHA modification consistently enhanced BBB penetration efficiency, in agreement with the trend shown in the original figures (Fig. 4-7). Accordingly, we have adopted a more cautious interpretation of its biological significance in the revised manuscript.

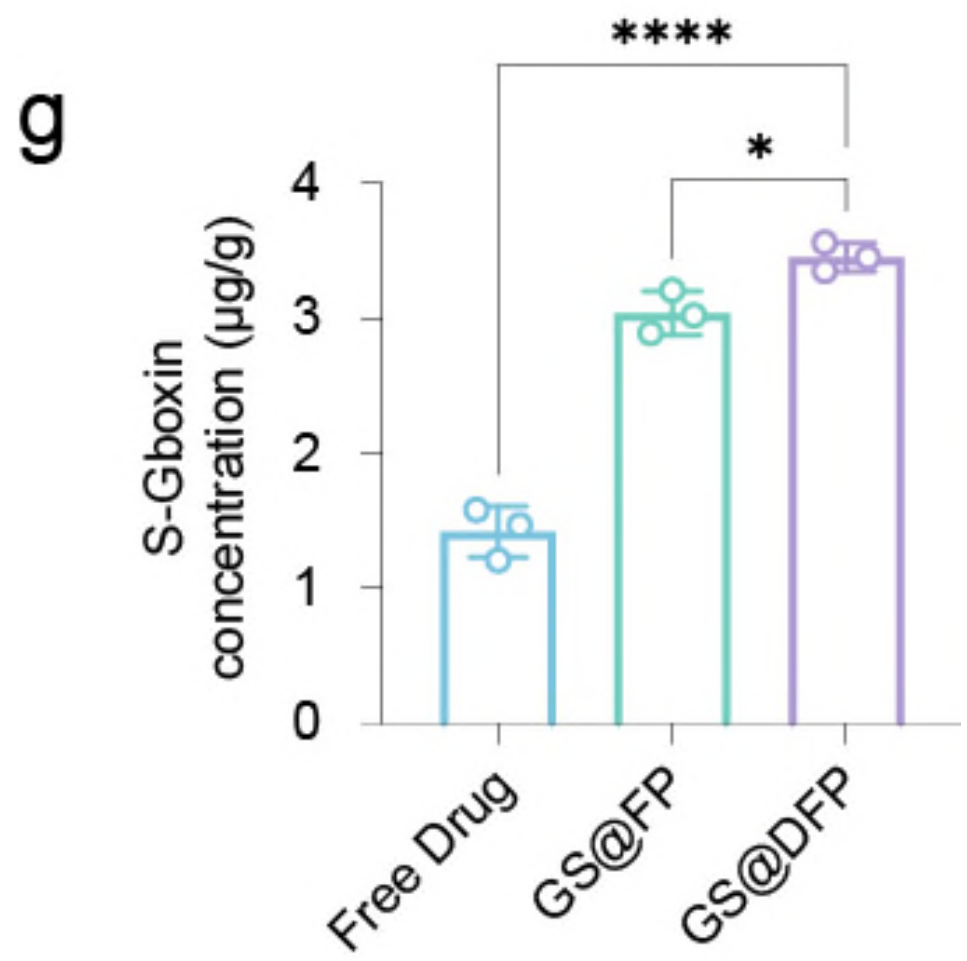

Figure R2. g) Distribution of S-Gboxin in tumor tissue under different treatment groups (n = 3 independent samples)

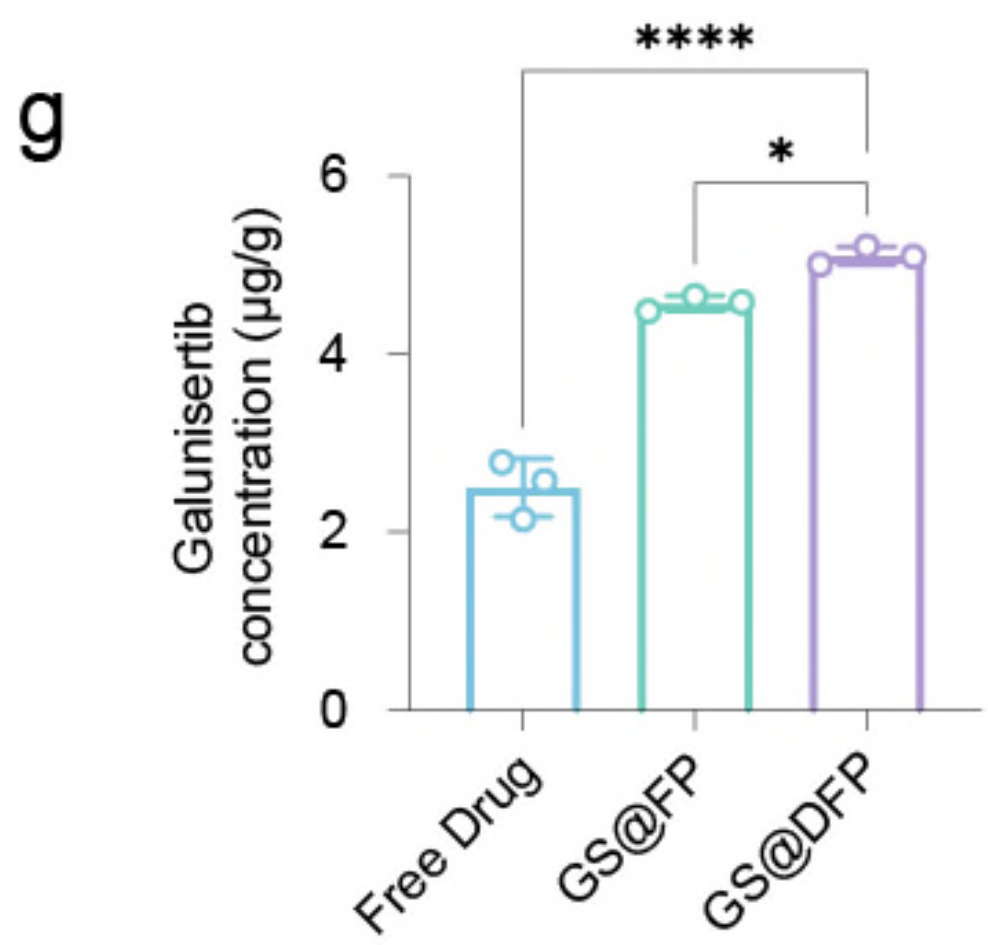

Figure R3. g) Distribution of Galunisertib in tumor tissue under different treatment groups (n = 3 independent samples)

Meanwhile, at the 6 h of the in vivo distribution experiment,, the tumor accumulation of the formulated dual-drug system was approximately 2.2-fold higher than that of the free drug group. Meanwhile, nonspecific distribution in non-target organs was reduced. We also observed that, compared with the non-modified group, the DHA-modified group exhibited relatively increased tumor accumulation of both drugs, directly indicating the important role of DHA modification in enhancing tumor-targeted accumulation.

Taken together, these results support a contributory role of Glut1 in BBB transport from a functional intervention perspective, while also indicating that it is not the sole pathway. Based on this understanding, we have revised the relevant statements in the manuscript to avoid overinterpreting the mechanism as one of complete dependence.

In the revised manuscript (**lines 209-210**), we have changed the explanation:

~~further confirming that DHA effectively mediates tumor targeting.~~

further confirming a contributory role of DHA in tumor targeting, potentially involving Glut1-mediated transport among other pathways.

(3) Regarding the concerns of "insufficient Glut1 dependency and limited BBB enhancement"

We sincerely thank the reviewer for raising this critical issue. Accordingly, in the revised manuscript, we have carefully refined the relevant descriptions by replacing potentially misleading expressions such as "Glut1-dependent transport" with "Glut1-involved or -facilitated transport," in order to avoid overinterpretation.

With respect to the relatively modest enhancement in BBB penetration conferred by DHA, we further analyzed this observation and consider it to be biologically reasonable for the following reasons:

1) The blood-brain barrier is a highly selective barrier, and exogenous delivery

systems typically struggle to achieve a significant increase in transport efficiency. Therefore, in the context of brain delivery research, even a modest improvement is of great significance (**Nat Rev Mater 7, 314－331 (2022). Nat Rev Mater 9, 628－642 (2024).**);

2) The brain-targeting performance of this system is more likely attributable to the synergistic effects of multiple factors, including the physicochemical properties of the nanocarrier as well as the transport-promoting effect introduced by DHA modification (**Adv. Funct. Mater.2023, 33, 2209216**. **Acta Pharm Sin B. 2023 Mar;13(3):1246-1261.**), rather than a single dominant mechanism;

3) From the overall trend, the DHA-modified group consistently exhibits improved BBB penetration across multiple time points. Although the magnitude is limited, the effect demonstrates good reproducibility and statistical significance;

4) In response to the reviewer's concern, we extended the incubation time in the supplementary experiments, which led to a more pronounced difference. This suggests that the incubation duration used in the original experiments may indeed have been insufficient, and further supports the facilitating role of DHA in promoting BBB penetration.

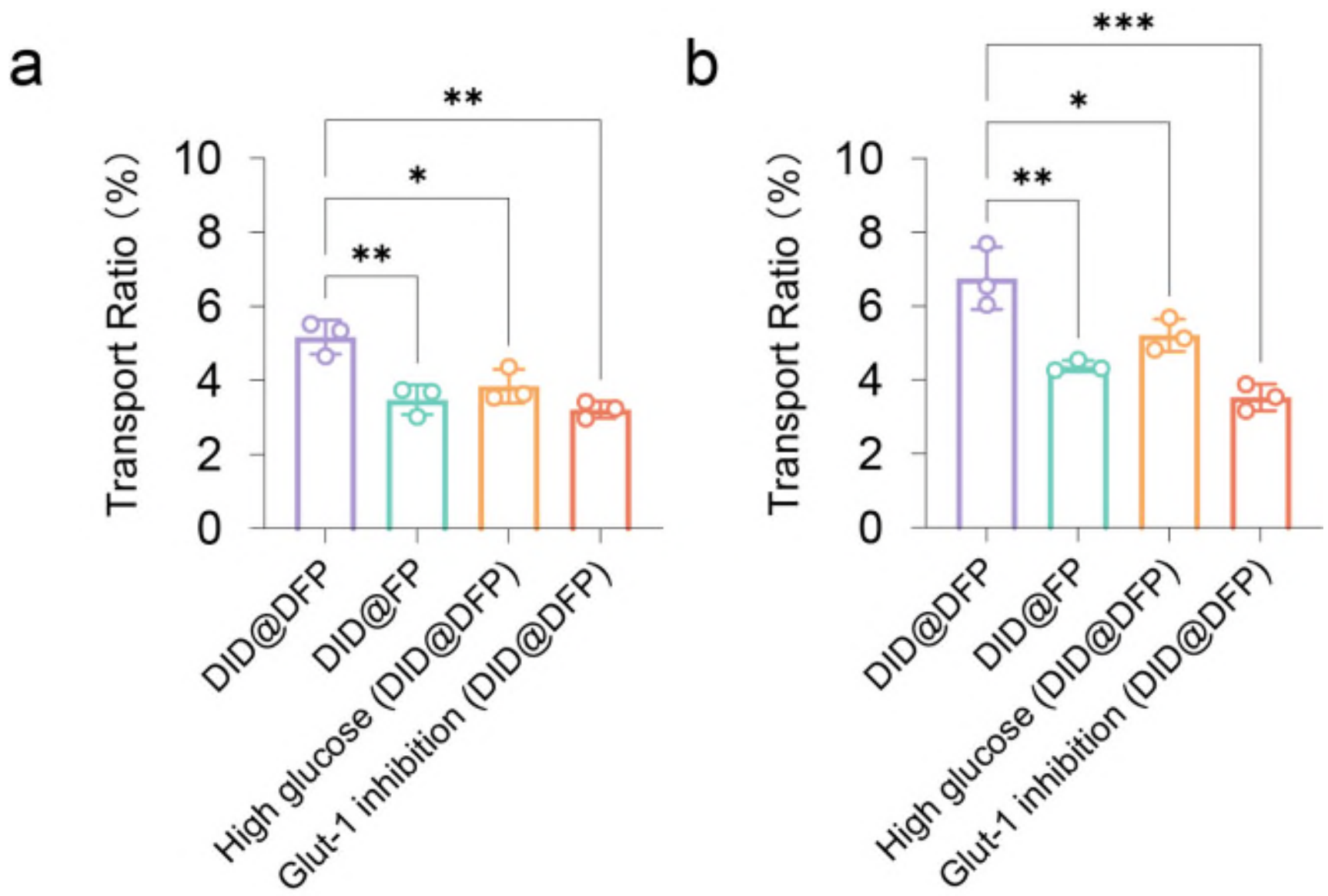

Figure R9. a) Quantitative analysis of DiD penetration ratios across different groups at 2 h (n = 3 independent samples); b) Quantitative analysis of DiD penetration ratios across different groups at 3 h (n = 3 independent samples).

Based on the above analysis, we have further reduced the emphasis on a single mechanism in the revised manuscript and refined our conclusion as follows: DHA modification can enhance brain delivery of the nanoplatform to a certain extent, with its role more appropriately described as an “enhancing effect” on BBB transport rather than a determining factor. Therefore, we consider that the current results support a contributory role of Glut1-related mechanisms in brain delivery, but do not establish it as the sole or decisive pathway. Accordingly, the manuscript has been revised to present a more cautious and objective interpretation.

(4) Regarding the concern of “nonspecific uptake” due to the widespread expression of Glut1

We thank the reviewer for raising this important point. We agree that the widespread expression of Glut1 may contribute to a certain degree of nonspecific distribution. Consistently, a moderate level of accumulation in the liver was observed, which is commonly associated with RES-mediated clearance in nanodelivery systems (**Nat Rev Mater 10, 252－267 (2025)**).

However, our biodistribution results also show that the nanoplatform achieves appreciable accumulation in brain tumor tissues, with an increasing trend compared to control groups. This suggests that Glut1-mediated transport contributes to BBB crossing and tumor delivery, rather than leading to indiscriminate uptake across all Glut1-expressing tissues.

Therefore, in this study, Glut1 is more appropriately interpreted as a facilitating mechanism that enhances delivery efficiency, rather than conferring strict tissue specificity. Taken together, these findings support that the observed targeting performance reflects functional delivery to tumor sites, while a certain degree of nonspecific distribution remains a known and manageable characteristic of such strategies.

(5) Rationale for selecting Glut1 as the targeting moiety

In response to the question of "why Glut1 was selected over other membrane proteins," we have provided a more systematic discussion in the revised manuscript. Compared with other potential targets, Glut1 offers several advantages:

1) It is highly expressed in both GBM and BBB endothelial cells, providing "dual-targeting" potential;

2) It is involved in glucose transport and represents an endogenous transport pathway that can be effectively exploited by analogs such as DHA; The glucose transporter family comprises several subfamilies, among which Glut-1 is extensively utilized by brain cells for glucose transport (**Protein Sci. 2016 Mar;25(3):546-58.**). Compared to Glut-2, Glut-1 transport is unidirectional. Additionally, blood glucose concentrations in the brain are relatively stable, ranging from approximately 3.5 to 5.5 mM, whereas the half-saturation constant for Glut-1 glucose transport is approximately 3–7 mM. This provides a window for the targeted transport of our Glut-1-modified formulations.

3) It has been validated in multiple studies as a feasible target for brain delivery system design, offering a solid foundation and practical feasibility. (**Adv. Funct. Mater.2023, 33, 2209216**. **Acta Pharm Sin B. 2023 Mar;13(3):1246-1261.**)

Summary

In summary, through additional expression validation, mechanistic experiments, and in vivo biodistribution analyses, we systematically demonstrate the elevated expression of Glut1 in GBM and its important role in brain delivery, thereby further strengthening the rationale and overall rigor of the study design.

3. According to fig. 5b, Galunisertib itself does not present obvious growth suppression effect and the tumor-killing effect of S-Gboxin through OXPHOS inhibition is not amplified by a further suppression of mitochondria transfer, no matter using the nanoparticle delivery system or not. So what is the necessity of including Galunisertib?

Response: We thank the reviewer for raising this important question. We understand

that, based on the observation in Fig. 5b that Galunisertib does not exhibit significant direct cytotoxicity and does not markedly enhance the killing effect of S-Gboxin, concerns have been raised regarding its necessity.

We would like to clarify that the primary rationale for incorporating Galunisertib in this study is not as a direct cytotoxic agent, but rather as a modulator of the tumor microenvironment, aimed at disrupting the functional interactions between tumor cells and surrounding stromal cells. It should be noted that evaluating its role solely based on short-term cell viability assays may underestimate its functional significance in regulating the tumor "support system." Therefore, its necessity should be assessed in the context of its overall impact on tumor microenvironment modulation. Specifically, our in vitro and in vivo studies collectively demonstrate that Galunisertib exerts the following effects:

(1) Inhibition of tumor-associated structures and invasive behavior

In vitro results show that Galunisertib significantly disrupts tumor microtube structures and suppresses the migratory and invasive capacities of tumor cells, indicating its role in regulating tumor physical architecture and dissemination behavior.

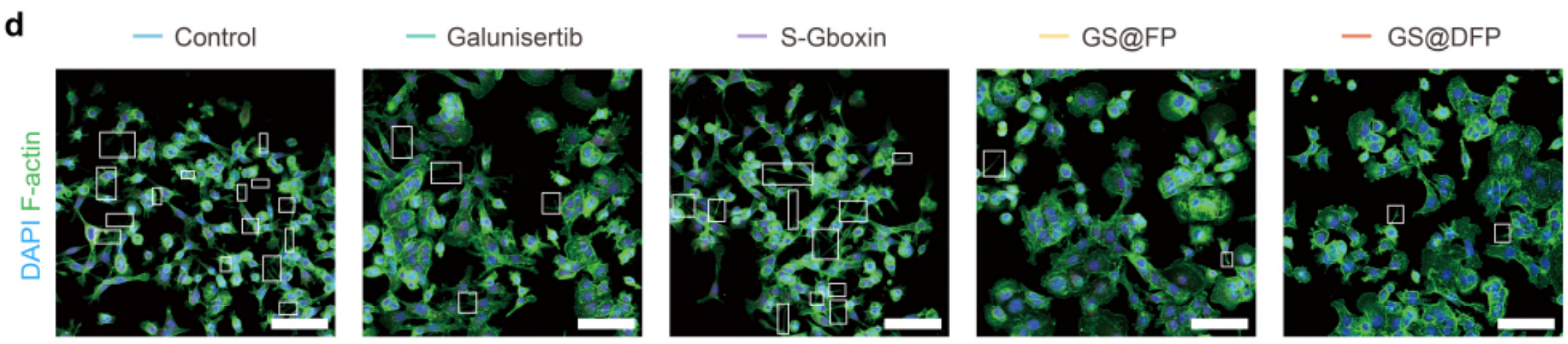

Fig. 6d. Tumor MT expression in GL261 cells after different treatments for 24 h. Scar bar = 100 μm.

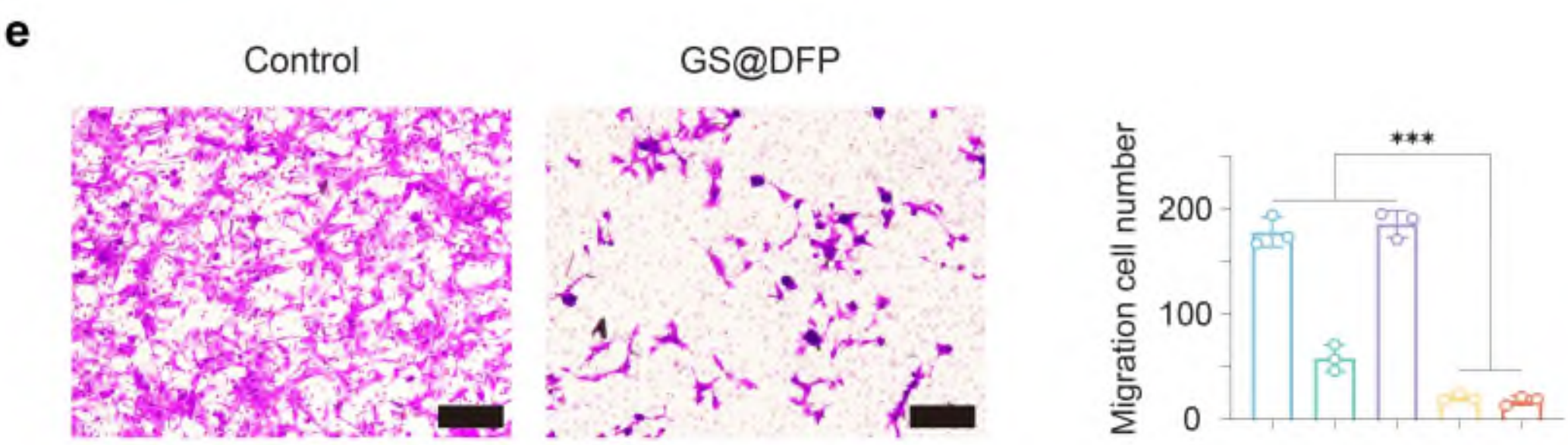

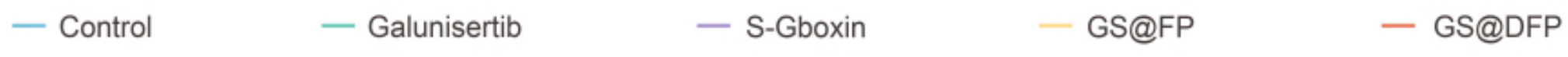

Fig. 6e. Representative images and quantification of invasive GL261 cells stained with crystal violet 24 h after incubation with different treatments, determined by Transwell assay (n = 3 independent samples). Scar bar = 100 μm.

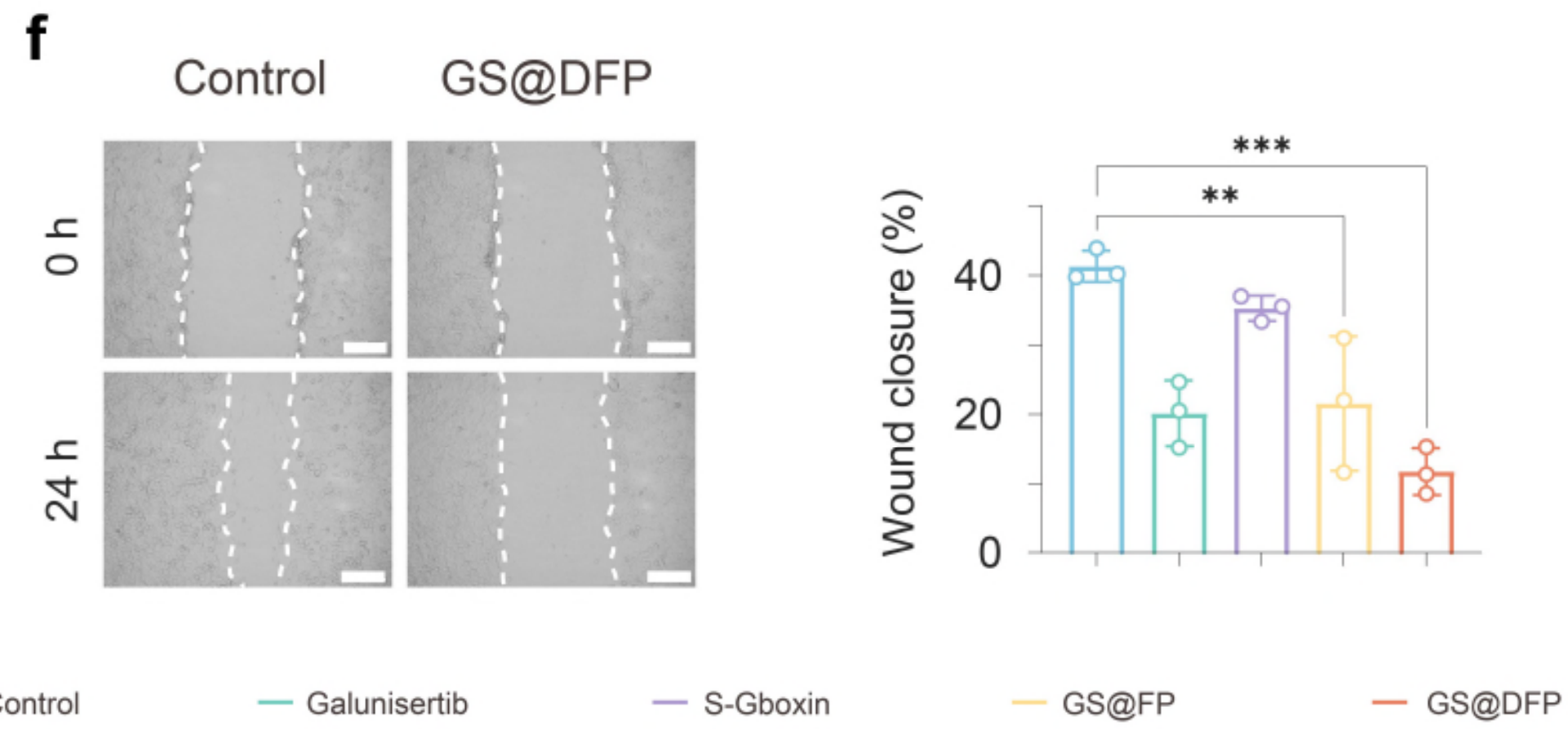

Fig. 6f. Representative images and quantification of migratory GL261 cells after incubation with different treatments for 24 h, determined by wound-healing assay (n=3 independent samples). Scar bar = 20 μm.

(2) Suppression of mitochondrial transfer-related processes

We observed that Galunisertib reduces material transfer from astrocytes to tumor cells and significantly decreases the level of mitochondrial transfer. In vitro, inhibition of mitochondrial transfer further reduces the proportion of cells entering active phases of the cell cycle. This effect contributes to weakening the metabolic support of tumor cells by targeting their "energy supply." Importantly, in the absence of such microenvironmental intervention, even if S-Gboxin suppresses intrinsic mitochondrial function in tumor cells, tumors may still acquire metabolic compensation via exogenous mitochondrial transfer, thereby diminishing therapeutic efficacy. Therefore, targeting this "compensatory pathway" is of clear necessity.

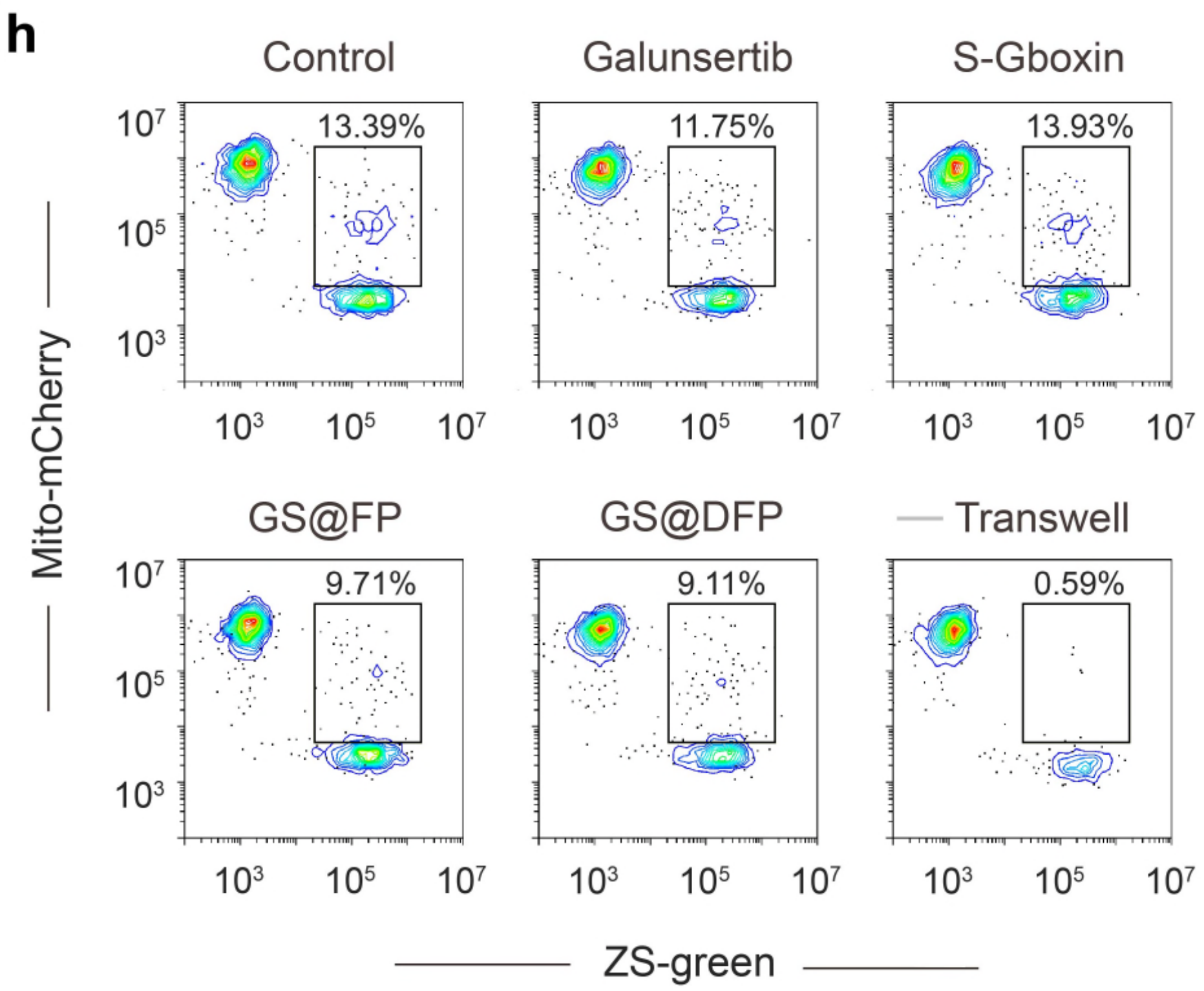

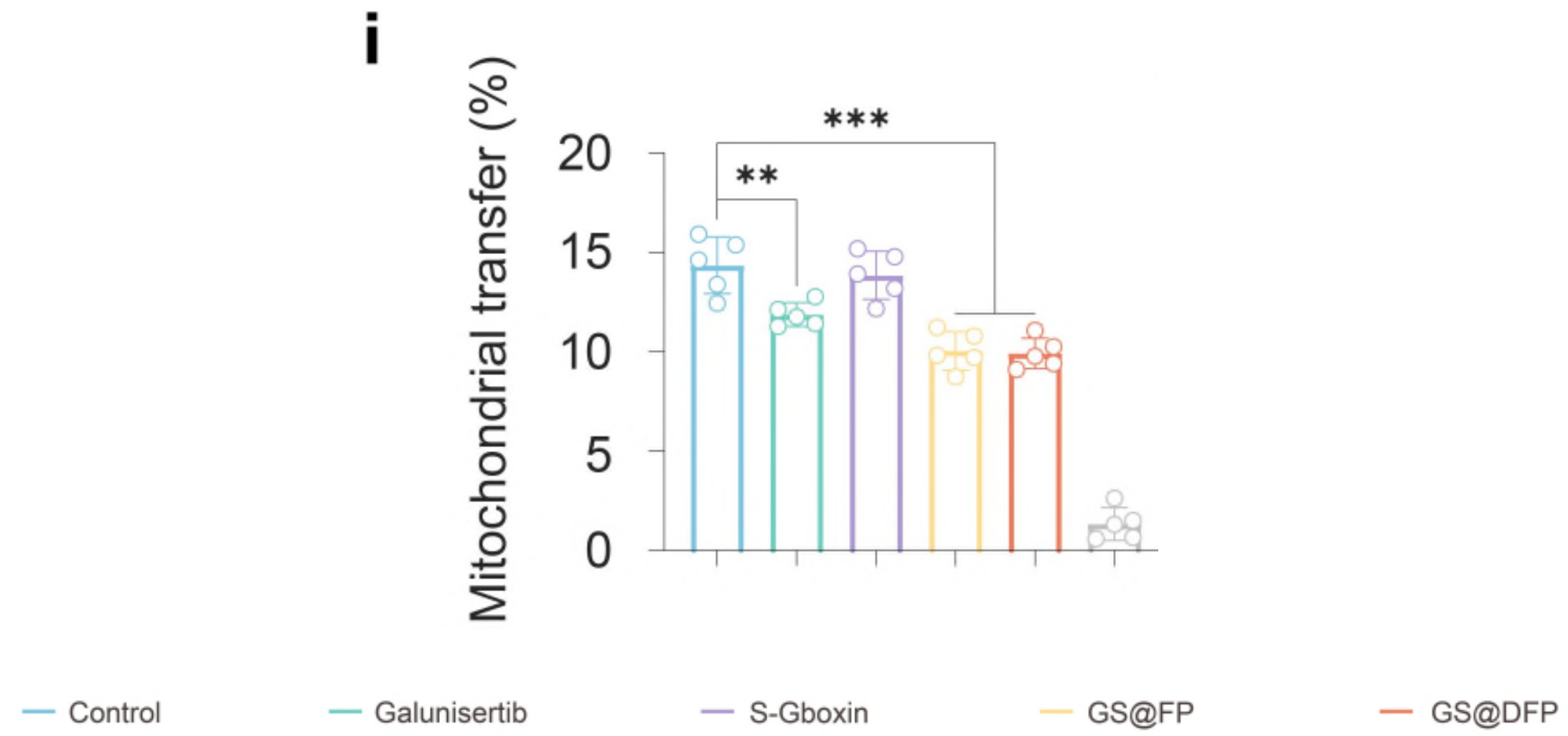

Fig. 6h i. Representative flow cytometry plots (h) and quantitative analysis (i) showing the proportion of ZS-green GL261 cells receiving astrocyte-derived mitochondria after co-culture with Mito-mCherry–transfected astrocytes (n=5 independent samples).

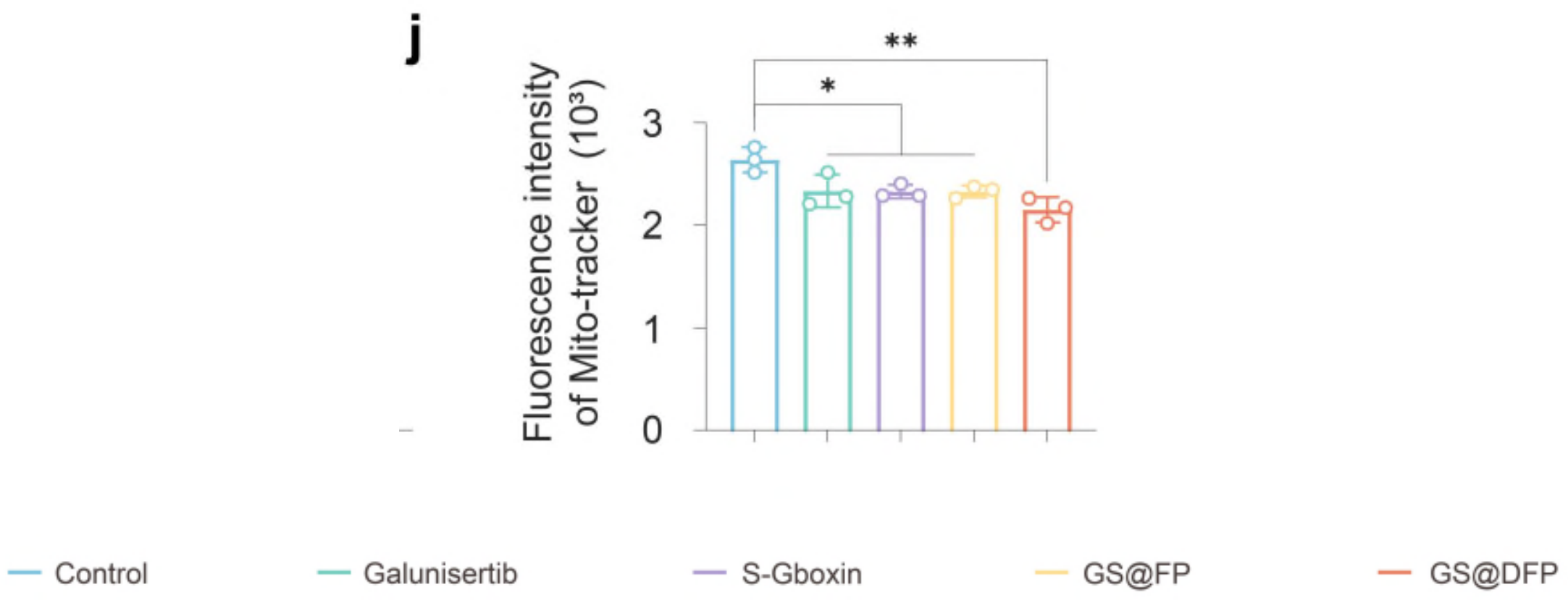

Fig. 6j. Flow cytometric quantification of mitochondrial content in ZS-green GL261 cells after co-culture with astrocytes (n=3 independent samples).

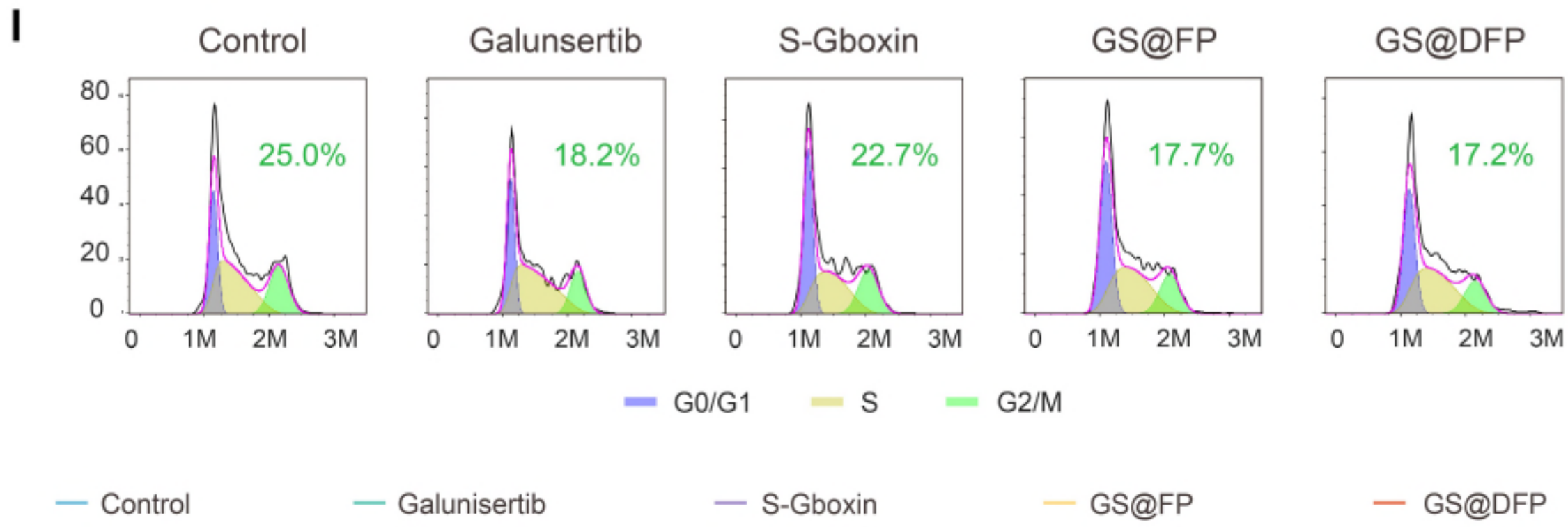

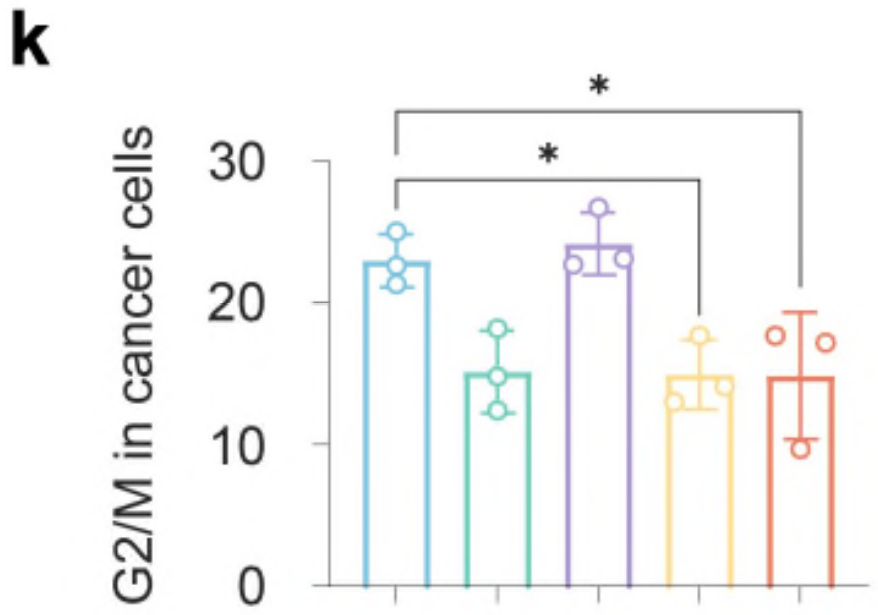

Fig. 6k l. Flow cytometric plots (l) and quantitative results (k) of cell cycle phases in ZS-green GL261 cells after co-culture with astrocytes (n=3 independent samples).

(3) Synergistic contribution to overall antitumor efficacy in vivo

Although Galunisertib alone does not exhibit pronounced cytotoxicity, in vivo it contributes to antitumor efficacy by inhibiting tumor microtube formation, reducing astrocyte-derived support, and suppressing mitochondrial transfer, thereby weakening the tumor “support system” and ultimately promoting tumor cell apoptosis.

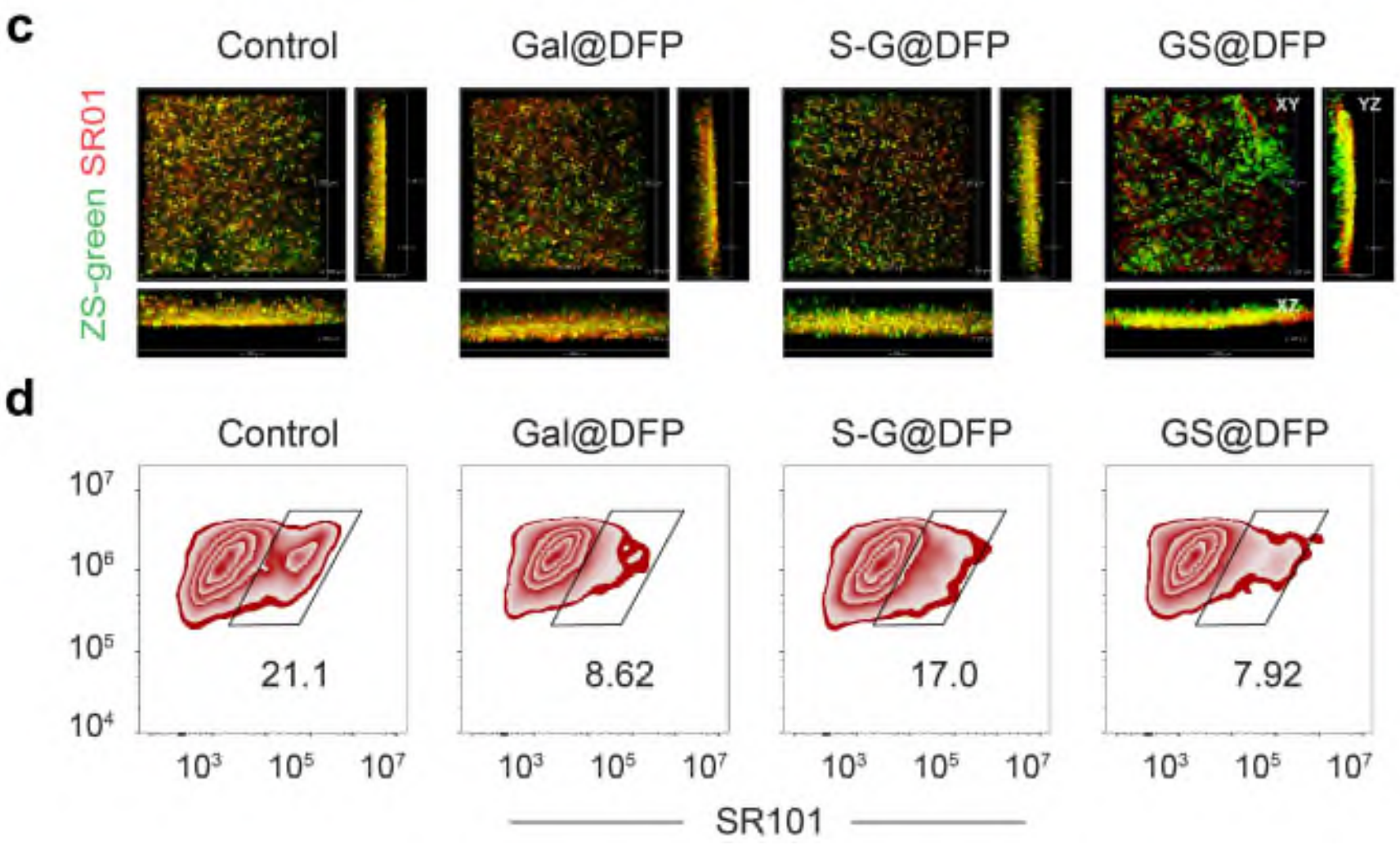

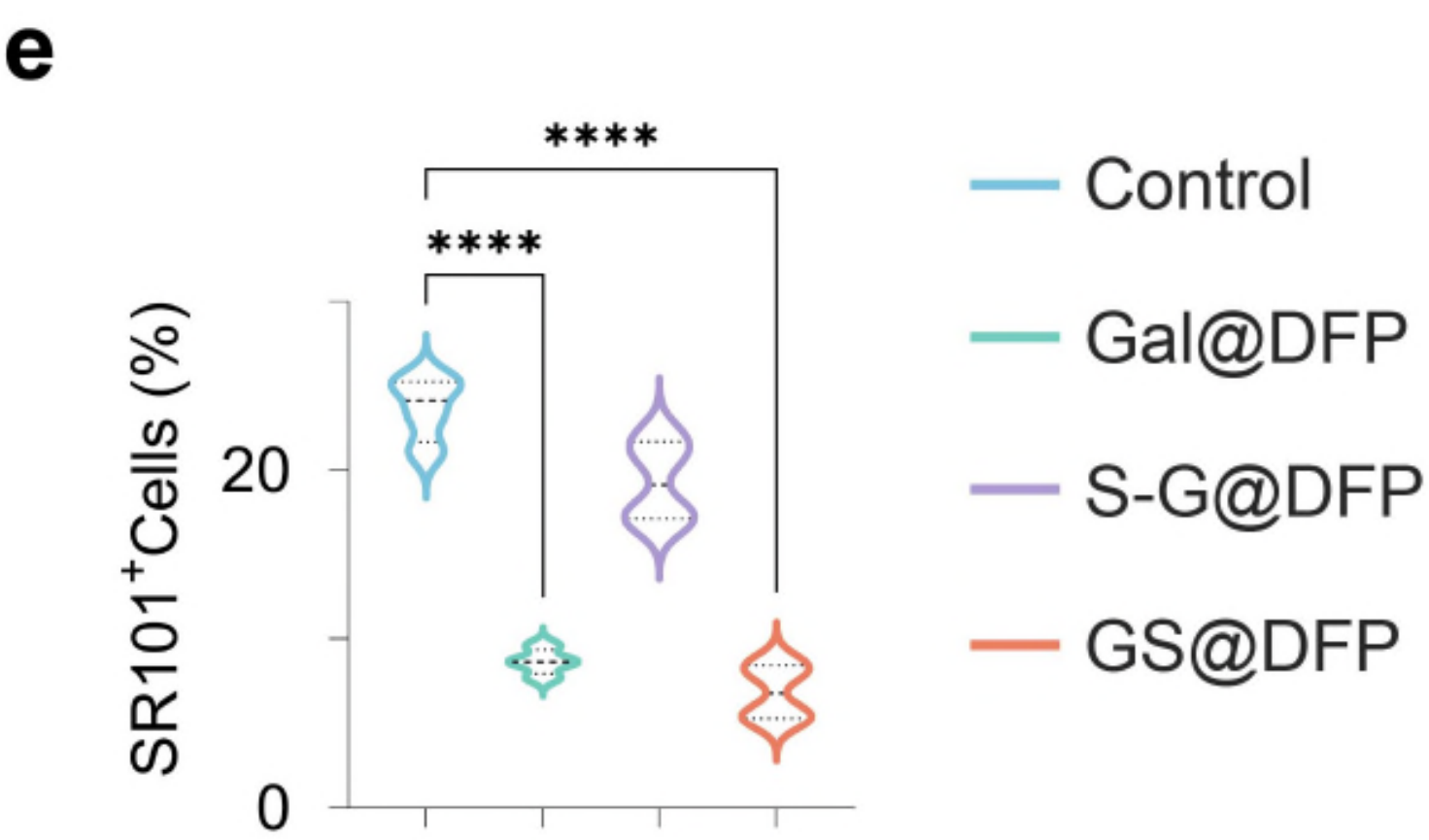

Figure 7c d e. **c)** *In vivo* observation of uptake of SR101 by cancer cells from astrocytes under two-photon microscopy; **d e)** Representative flow cytometry plots (**d**) and quantitative analysis (**e**) of SR101⁺ cancer cells (n=4 independent samples);

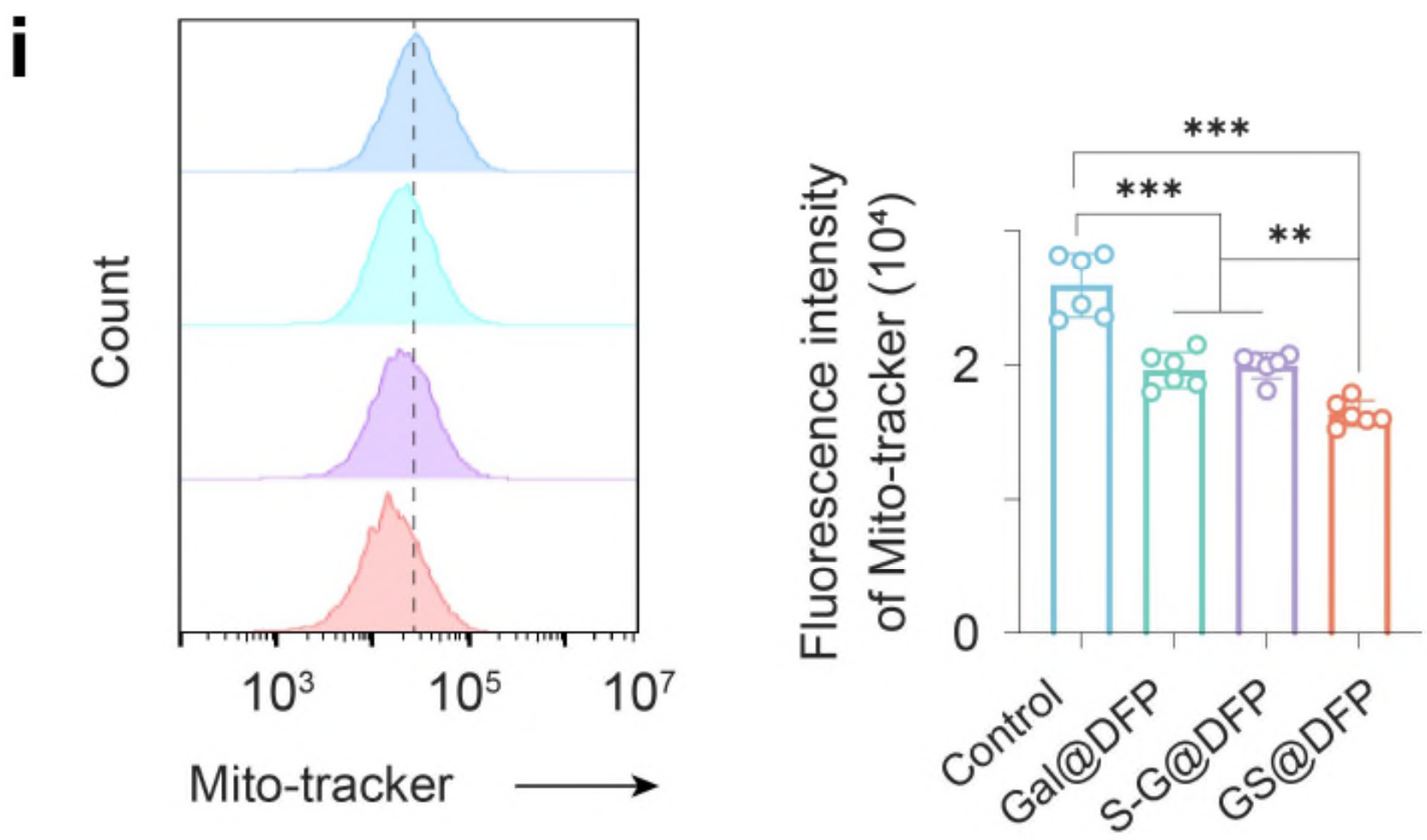

Figure 7i. **i** Representative FCM plots and quantitative results of mitochondrial content in tumors from different treatment groups (n = 6 independent samples);

(4) Regulation of cellular states and immune-related processes

In in vivo studies, Galunisertib demonstrates the ability to alleviate the immunosuppressive tumor microenvironment, which is of significant importance for enhancing overall antitumor efficacy.

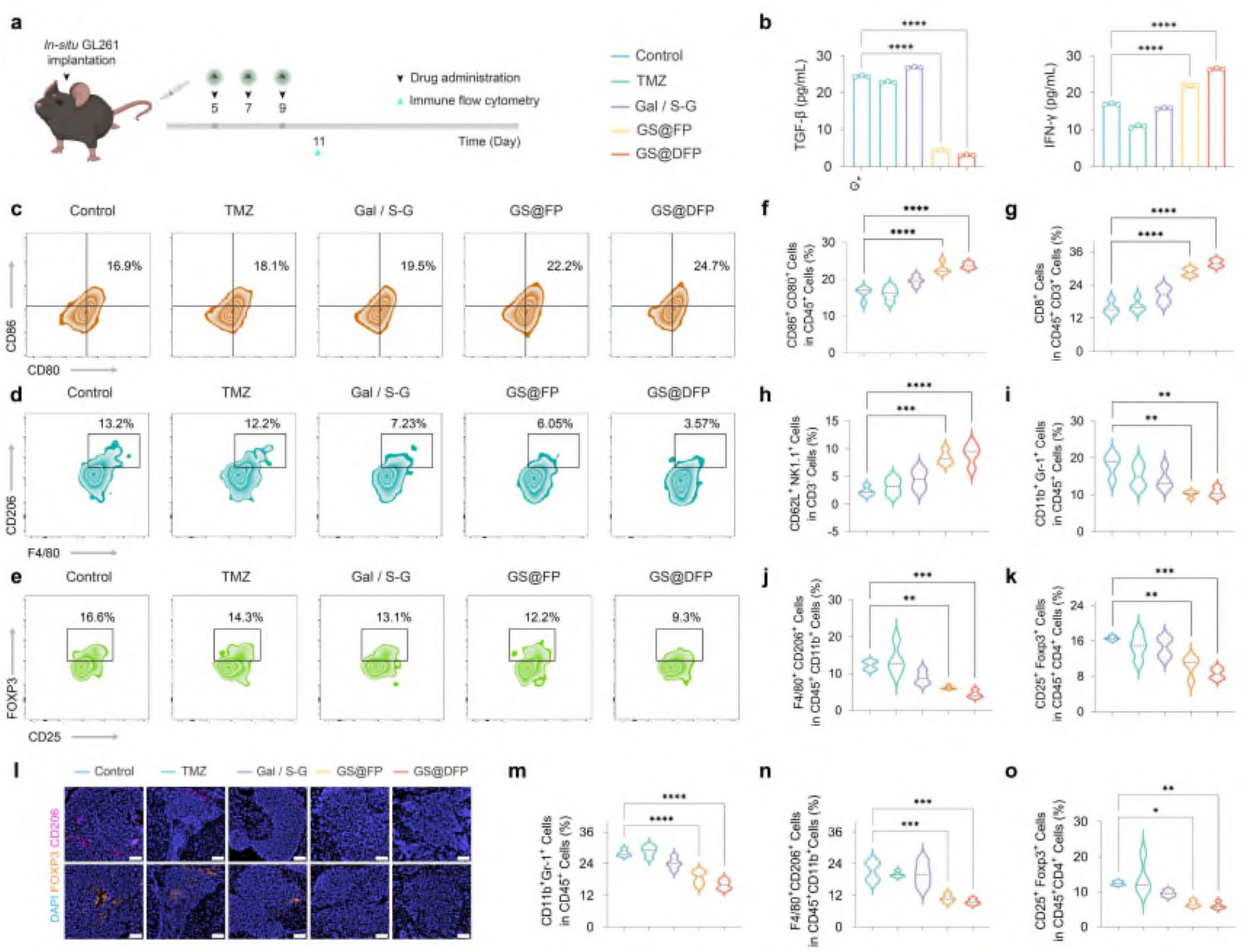

Fig. 9 | GS@DFP activates the tumor immune microenvironment. **a** Schematic illustration of the GL261-Luci tumor-bearing mouse model establishment, treatment schedule and subsequent immunological analyses; **b** ELISA measurements of TGF-β and IFN-γ secretion in GBM after treatment completion (n=3 mice in each group); **c-e** Representative flow cytometry plots showing DC maturation (**c**) and the intratumoral infiltration of M2 macrophages (**d**) and Treg cells (**e**) in GBM. (**f**) Flow cytometry analysis of the proportion of mature DCs in cervical lymph nodes (n=4 mice in each group); **g-k** Flow cytometry analysis of the infiltration of immune effector cells (T cells, NK cells) and immunosuppressive cells (MDSCs, M2 macrophages, and Treg

cells) in GBM (n=4 mice in each group). **l** Fluorescence staining of FOXP3 and CD206 expression in orthotopic GL261 tumor-bearing mice after treatment. Scale bar = 100 μm. **m-o** Flow cytometry analysis of the infiltration of MDSCs, M2 macrophages, and Treg cells in GBM (n=4 mice in each group). $^{*}p < 0.05$, $^{**}p < 0.01$, $^{***}p < 0.001$, $^{****}p < 0.0001$.

Based on the above results, we propose that the therapeutic strategy in this study does not rely on the direct cytotoxicity of a single agent, but rather establishes a synergistic intervention paradigm combining "metabolic inhibition (S-Gboxin) + microenvironment modulation (Galunisertib)". Specifically, S-Gboxin primarily targets oxidative phosphorylation (OXPHOS) in tumor cells, directly impairing their energy metabolism, whereas Galunisertib suppresses mitochondrial transfer and tumor microenvironmental support, thereby blocking tumor "metabolic compensation mechanisms" and immune evasion pathways. These two agents act on the "metabolic consumption side" and the "metabolic supply side," respectively, forming a complementary intervention that enhances overall therapeutic efficacy. Therefore, the role of Galunisertib is better characterized as "de-supporting" rather than "direct cytotoxicity." Its value lies not in increasing short-term cytotoxic readouts, but in disrupting the key ecological niches required for tumor maintenance and adaptation.Furthermore, these findings suggest that, in complex tumor systems, evaluation strategies based solely on cytotoxicity may be insufficient to fully capture the therapeutic potential of microenvironment-modulating agents.

In summary, we believe that the role of Galunisertib in this study should not be assessed based on its direct cytotoxic potency, but rather understood as weakening tumor metabolic compensation and adaptive capacity through modulation of the tumor microenvironment and intercellular interactions, thereby enhancing the overall efficacy of the therapeutic system.

4. Many experimental details are missing, and as a result, a lot of cross-group comparison, like those in fig. 3, fig. 5 and fig. 6, can not result in solid conclusions. The authors need to provide experimental details, including dosage, treatment duration, etc. in both figure legend and methods, and re-modify descriptions in the manuscript.

<u>**Response:**</u> We thank the reviewer for the valuable comments regarding experimental details and the comparability of results. In response to these suggestions, we have made the following revisions in the manuscript:

(2) Revision of figure legends and main text:

For cross-group comparisons presented in Figures 3, 5, and 6, we have revised both the figure legends and the main text to clearly specify treatment conditions, experimental parameters, and statistical methods for each group.

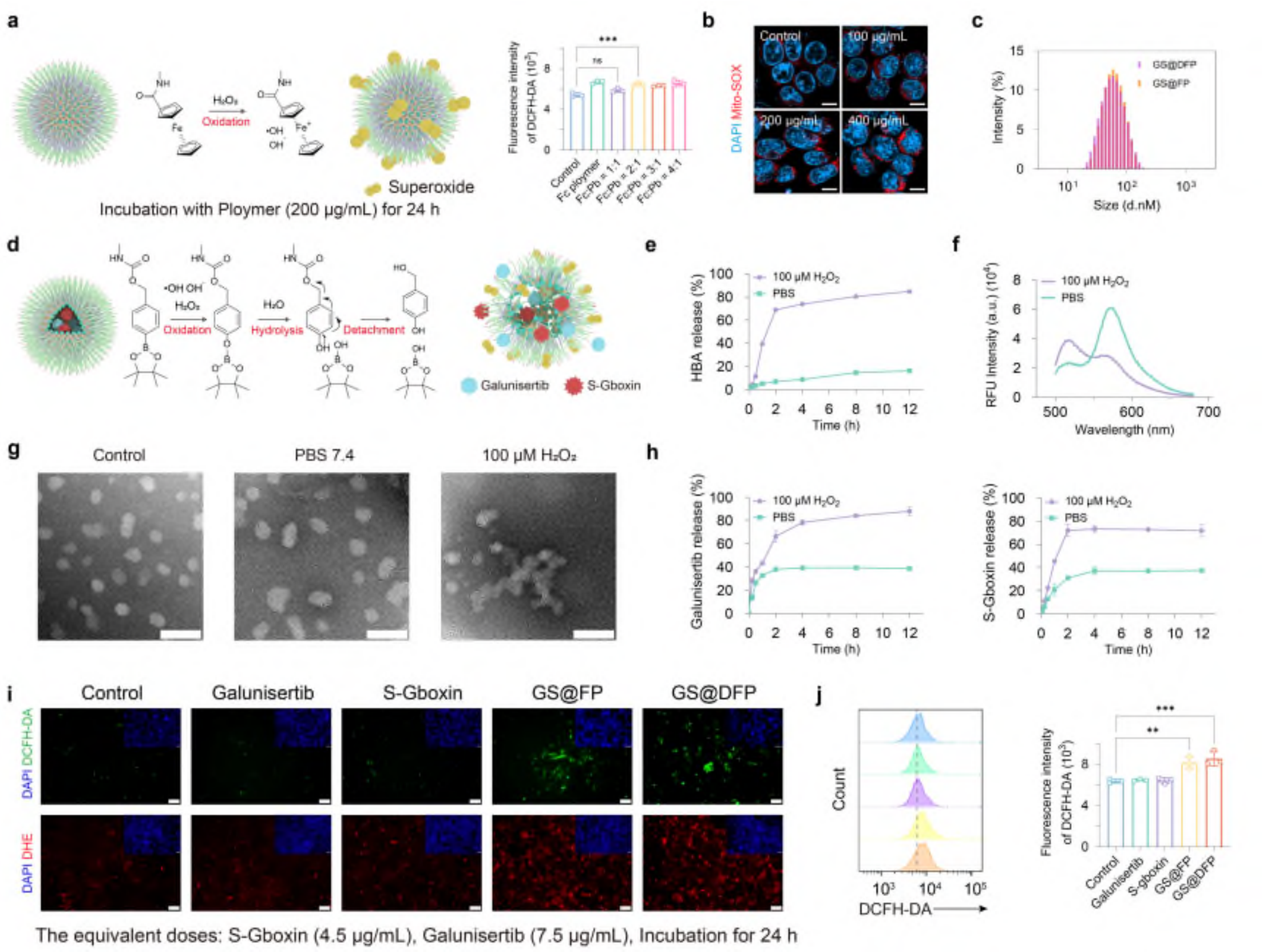

Fig. 3 | Construction and characterization of GS@DFP. **a** Left: Schematic illustration showing the mechanism of superoxide generation by ferrocene within blank nanoparticles. Right: Quantitative analysis of mean fluorescence intensity (MFI) indicating intracellular oxidative stress levels in GL261 cells after incubation with

different blank nanoparticles (n=3 independent samples). **b** Blank nanoparticles with a 2:1 ratio induce mitochondrial superoxide accumulation. Scar bar = 20 µm; **c** Hydrodynamic diameters of GS@FP and GS@DFP. **d** Schematic illustration of the ROS-responsive cleavage mechanism of GS@DFP. **e** Cumulative release profiles of HBA from GS@DFP under different conditions (n=3 independent samples). **f** H_2O_2 triggers the hydrophilic-hydrophobic transition that alters the emission intensity (535-580 nm) of the fluorescent donor Pb-FITC polymer and the fluorescent acceptor Fc-TAMRA polymer at an excitation wavelength of 460 nm. **g** TEM images of GS@DFP before drug release and after 24 h of release under different conditions. Scar bar = 100 nm. **h** Cumulative release profiles of Galunisertib and S-Gboxin from GS@DFP under different conditions (n=3 independent samples). **i** Representative CLSM images showing intracellular oxidative stress indicated by DCFH-DA and DHE probes under different treatment conditions. Scar bar = 100 µm. **j** Representative FCM and quantitative results of MFI for DCFH-DA-stained GL261 cells (n=3 independent samples). ns>0.05, $^{**}p < 0.01$, $^{***}p < 0.001$.

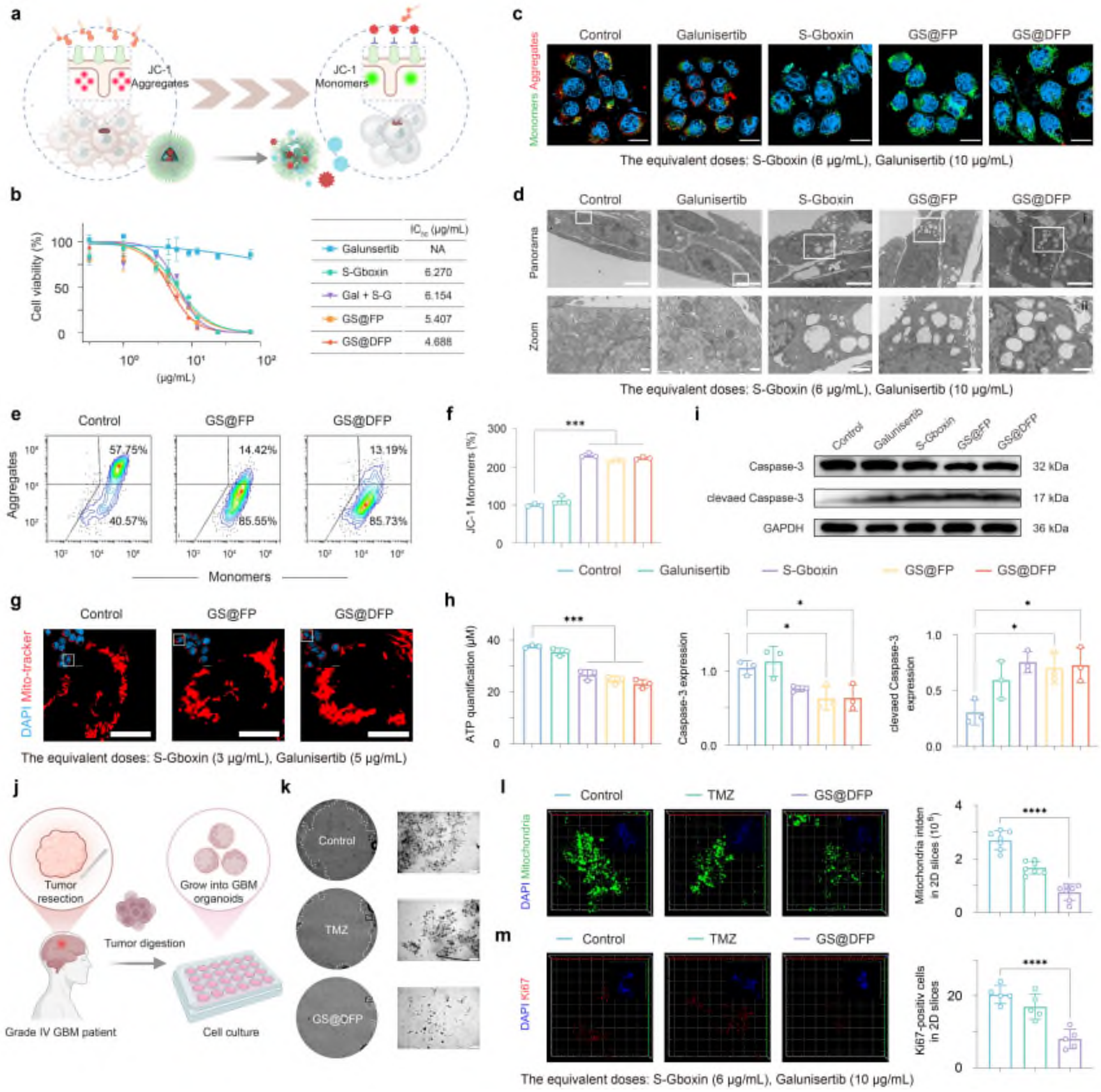

Fig. 5 | GS@DFP disrupts mitochondria and inhibits cell proliferation. a Schematic illustration of mitochondrial disruption and JC-1–indicated membrane potential changes. **b** Fitted cell viability curves and half-maximal inhibitory concentrations (IC_{50}) of GL261 cells after treatment with Galunisertib, S-Gboxin, Galunisertib + S-Gboxin, GS@FP, or GS@DFP for 24 h (n=4 independent samples). **c** Representative CLSM images of JC-1–stained GL261 cells after different treatments for 6 h. Scar bar = 20 μm. **d** TEM images showing mitochondrial morphological changes in cancer cells after different treatments for 12 h. Scar bar = 5 μm in (i). Scar bar = 1 μm in (ii). **e f** Representative flow cytometry plots of JC-1 staining (**e**) and quantitative analysis of JC-1 monomers (**f**) in GL261 cells (n=3 independent samples). **g** CLSM images of mitochondrial morphology in GL261 cells after treatments. Scar bar = 10 μm. **h** Intracellular ATP levels of GL261 cells measured after different

treatments for 12 h (n=3 independent samples). **i** Western blot analysis and quantitative results of apoptosis-related proteins Caspase-3 and cleaved Caspase-3 following mitochondrial disruption (n=3 independent samples). **j** Establishment of patient-derived tumor organoid models from primary WHO grade IV GBM tissues. **k** Panoramic and representative local images showing the effects of TMZ or GS@DFP treatment for 48 h on GBM organoid formation. Scar bar = 100 μm. **l** Representative 3D mitochondrial staining images and fluorescence quantification of GBM organoids after different treatments (n=7 independent slices). **m** Representative 3D Ki67 fluorescence staining images and quantification of $Ki67^+$ cells in GBM organoids after different treatments (n=5 independent slices). *$p < 0.05$, ***$p < 0.001$, ****$p < 0.0001$.

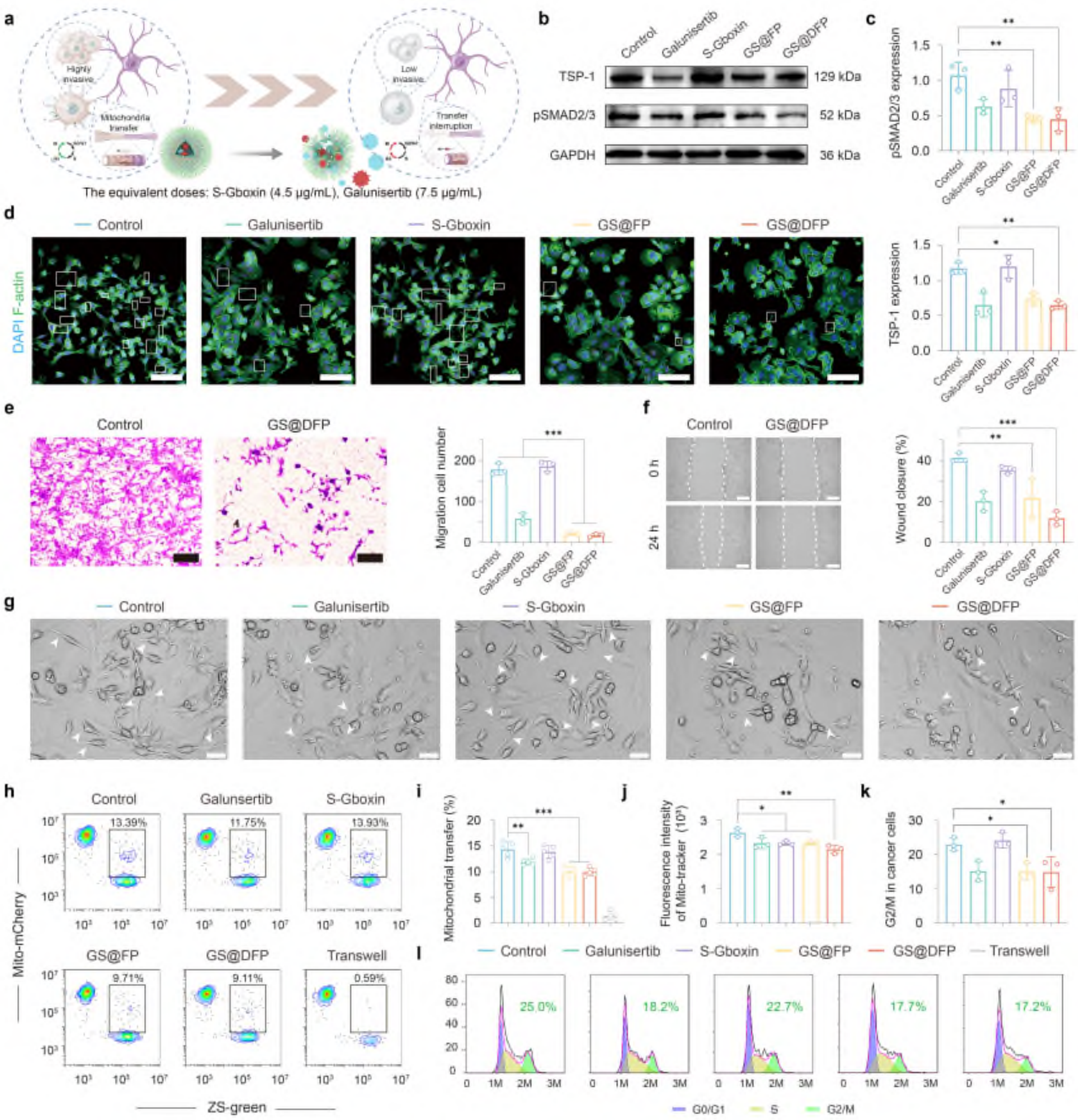

Fig. 6 | GS@DFP inhibits tumor MTs, thereby downregulating mitochondrial transfer and malignant proliferation. **a** Schematic illustration showing that GS@DFP inhibits tumor MTs, thereby weakening cancer cell invasion, reducing mitochondrial transfer, and decreasing tumorigenicity. **b c** Western blot analysis of GL261 cells treated with Galunisertib, S-Gboxin, GS@FP, or GS@DFP for 24 h, showing the expression of pSMAD2/3 and TSP-1 (**b**) and the quantitative results (**c**) (n=3 independent samples). **d** Tumor MT expression in GL261 cells after different treatments for 24 h. Scar bar = 100 μm. **e** Representative images and quantification of invasive GL261 cells stained with crystal violet 24 h after incubation with different treatments, determined by Transwell assay (n=3 independent samples). Scar bar = 100 μm. **f** Representative images and quantification of migratory GL261 cells after incubation with different treatments for 24 h, determined by wound-healing assay (n=3 independent samples). Scar bar = 20 μm. **g** Representative images showing tumor MT-mediated connections between cancer cells and astrocytes after 24 h of pretreatment. Scar bar = 20 μm. **h i** Representative flow cytometry plots (**h**) and quantitative analysis (**i**) showing the proportion of ZS-green GL261 cells receiving astrocyte-derived mitochondria after co-culture with Mito-mCherry–transfected astrocytes (n=5 independent samples). **j** Flow cytometric quantification of mitochondrial content in ZS-green GL261 cells after co-culture with astrocytes (n=3 independent samples). **k l** Flow cytometric plots (**l**) and quantitative results (**k**) of cell cycle phases in ZS-green GL261 cells after co-culture with astrocytes (n=3 independent samples). $*p < 0.05$, $**p < 0.01$, $***p < 0.001$.

(2) Supplementation of experimental details:

In the Methods section and corresponding figure legends, we have provided detailed descriptions of key parameters for each experimental group, including dosage, treatment duration, administration route, and cell or animal handling conditions, to ensure that the data can be accurately interpreted and readily reproduced.

(3) Ensuring the reliability of conclusions:

By providing complete experimental information and clearer descriptions, readers are

now able to accurately interpret comparisons among groups, thereby supporting the conclusions presented in the manuscript.

We believe that these revisions substantially improve the reproducibility of the experiments and the clarity of data interpretation, while also strengthening the validity and persuasiveness of the comparative analyses.

Minor Comments:

1. Authors shall provide figure files with high resolution. A lot of images in the manuscript, especially those of immunofluorescence assays, are so blurred to tell details. For example, fig.2f can not prove the fluorescence signal is mitochondrial, in terms of smeared localization.

Response:

Thank you for the reviewer's comment regarding image resolution and mitochondrial localization. We would like to clarify that the version provided for reviewer was a compressed PDF, in which some immunofluorescence images appeared blurred due to file size limitations during export.

In the original high-resolution images, the fluorescence signals exhibit clear subcellular localization patterns. To address this concern, we have now provided high-resolution versions of figures Fig. 2f. In these updated images, the mitochondrial signals display distinct punctate distributions, consistent with typical mitochondrial morphology, rather than diffuse or smeared patterns.

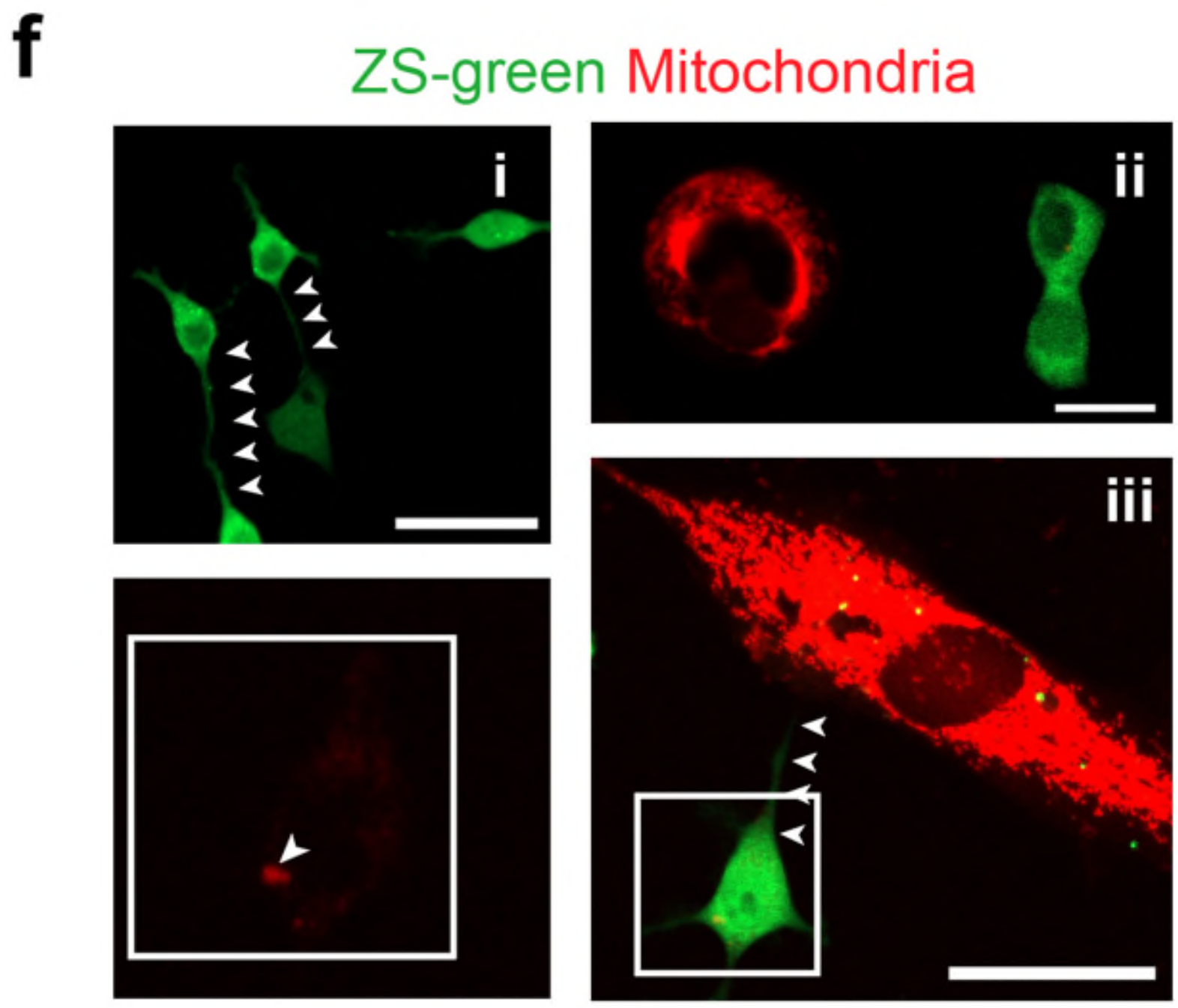

Figure. 2f. GL261 cells form intercellular connections (i); mitochondrial transfer does not occur without tumor MTs (ii), while hijacking mitochondria from astrocytes in the presence of MTs (iii). Scar bar = 20 µm.

In addition, the mitochondrial identity of the signal is supported by the use of specific mitochondrial probes and the observed intracellular localization pattern.

We believe that the improved image quality in the revised version clearly supports the mitochondrial localization and eliminates the ambiguity caused by compression artifacts.

2. Please point out the tubular structures referred to transfer mitochondria in fig. 2e.

Response: We thank the reviewer for pointing out this issue.

It should be noted that this experiment was conducted using a live-cell imaging system, in which tumor cells express Zs-green fluorescent protein, whereas astrocytes were not globally labeled; only their mitochondria were specifically stained. Under these imaging conditions, intercellular protrusions may lack sufficient Zs-green signal coverage, resulting in some tubular structures appearing less continuous or less well-defined compared with those observed under fixed-cell staining conditions. In addition, because this was a live-cell imaging experiment, actin cytoskeleton staining

was not feasible, which may further limit the precise delineation of thin structural boundaries.

Nevertheless, these structures can still be identified based on the following features: (i) they appear as elongated protrusions spanning the space between adjacent cells; and (ii) mitochondrial fluorescence signals are distributed directionally along these structures, rather than appearing as diffuse background signals, suggesting their potential involvement in intercellular mitochondrial transfer.

3. According to fig. 3i, S-Gboxin sole treatment also generates ROS although being much lower than GS@FP and GS@DFP, but it is not presented in statistic data, fig. 3i.

Response: We thank the reviewer for pointing out the changes in ROS levels upon treatment with S-Gboxin alone. Upon further analysis, we found that this discrepancy is primarily attributable to differences in the ROS detection methods employed. In the revised manuscript, for quantitative analysis, we used DCFH-DA (2′,7′-dichlorodihydrofluorescein diacetate) as the indicator (**Adv Mater. 2026 Jan;38(2):e14419.**). This probe mainly reflects overall cellular oxidative stress, encompassing the cumulative effects of multiple ROS species, and is therefore suitable for evaluating the pronounced ROS amplification induced by GS@FP or GS@DFP. Under this assay, the ROS level generated by S-Gboxin alone is relatively low and thus not prominently reflected in the quantitative statistical data.

However, when using DHE (dihydroethidium) staining, which specifically detects superoxide (O_2^{-}) generation (**Adv Mater. 2026 Jan;38(2):e09444.**), we observed that S-Gboxin monotherapy does indeed induce a slight increase in ROS. This is consistent with its mechanism of mildly elevating oxidative stress through inhibition of mitochondrial function in tumor cells. It is important to note that DHE is more sensitive to transient, mitochondria-derived ROS, whereas DCFH-DA reflects overall, cumulative ROS levels. This difference explains why the S-Gboxin-alone group did not show a significant increase in ROS in the original quantitative analysis.

To avoid potential misunderstanding, we acknowledge that S-Gboxin alone may

indeed induce a modest increase in ROS; however, this effect is substantially lower than the ROS amplification observed with GS@FP/GS@DFP. The enhanced ROS generation in GS@FP and GS@DFP primarily arises from ferrocene-mediated Fenton-like reactions, which depend on the inherently elevated basal ROS levels in tumor cells, thereby allowing a degree of selectivity over normal cells. This experimental design clearly distinguishes the mild oxidative stress induced by mitochondrial inhibition from the robust ROS amplification mediated by the nanoplatform, supporting our conclusion regarding ROS amplification and selective tumor cell killing.

In summary, this observation is fully consistent with expectations and does not reflect experimental bias or data omission.

4. The so-called tubular structures in fig. 6g are much more like junctions between cells and authors are encouraged to give direct evidences to prove they are MT connections.

<u>Response:</u> We thank the reviewer for this insightful comment. In response, we have carefully re-examined the structures shown in Fig. 6g. We would first like to clarify that these tubular structures have already been explicitly annotated in the main text to improve their visibility and interpretability.

We understand the reviewer's concern that these structures may not appear sufficiently prominent in the images. We also acknowledge that there are certain limitations in the current experimental conditions. Although we have not yet performed additional dynamic tracking or 3D reconstruction analyses to fully exclude other types of intercellular contacts, based on previously reported morphological characteristics of tumor microtubes—such as length, diameter, and elongated morphology—together with the distribution patterns observed in our study, we consider that these structures are most consistent with tumor microtubes.

5. The n number (i.e. the sample size used to derive statistics) should be provided and defined as a precise value (not a range), using the wording "n=X

samples/cells/independent experiments" etc. where applicable.

Response: We thank the reviewer for pointing out the lack of clarity regarding sample size reporting. In the revised manuscript, we have provided detailed clarifications for all statistical analyses and standardized the notation to explicitly indicate "n = X independent samples," rather than using ranges as in the previous version. This clarification ensures the reliability and reproducibility of the statistical analyses.

Reviewer #2:

This manuscript presents a multifunctional ROS-responsive nanoplatform (GS@DFP) designed to simultaneously disrupt intrinsic tumor mitochondrial function and astrocyte-to-glioblastoma mitochondrial transfer. By integrating DHA-mediated BBB penetration, ferrocene-driven ROS amplification, mitochondrial ATP synthase inhibition (S-Gboxin), and TGF-β/SMAD/TSP-1 pathway blockade (Galunisertib), the authors address a critical and emerging mechanism of GBM metabolic adaptation and therapy resistance.

The study is comprehensive, technically rigorous, and conceptually innovative. The combination of advanced nanomaterial engineering with mechanistic insights into tumor–astrocyte crosstalk and immune remodeling is a major strength. However, some mechanistic clarifications are required to fully support the conclusions and enhance translational relevance.

• The authors attribute enhanced BBB penetration and tumor targeting primarily to DHA-mediated Glut1 transport. While supportive in vitro and in vivo evidence is provided, direct mechanistic validation remains incomplete: it is unclear whether Glut1 is strictly required for nanoparticle transport across the BBB; the in vitro BBB model lacks competitive inhibition or transporter-blocking experiments.

Response: We thank the reviewer for the valuable comments regarding the mechanism of DHA-mediated Glut1 transport in blood–brain barrier (BBB) penetration. We fully agree that direct experimental evidence is required to support this mechanism. To address this, we conducted additional mechanistic validation experiments, including Glut1 inhibition and competition studies (using a Glut1 inhibitor as well as excess glucose/DHA for competition). Specifically, bEnd.3 cells were pretreated with the Glut1 inhibitor WZB117 (10 μM) or 50 mM glucose solution for 30 min, and the permeability coefficients were measured before and after treatment. An in vitro Transwell model was again employed to simulate the BBB, with an extended incubation time in this set of experiments.

The results show that under high-glucose competitive conditions, Glut1-mediated nanoparticle transport was hindered, and the efficiency of BBB penetration by

DHA-modified nanoparticles decreased to approximately 75%. Under Glut1 inhibition conditions, the ability of nanoparticles to cross the BBB was significantly reduced, dropping to about 50% of the original level compared with the control group. These findings indicate that when Glut1 function is disrupted, the penetration efficiency of DHA-modified nanoparticles is consistently diminished, thereby directly supporting the contributory role of Glut1 in DHA-mediated BBB transport.

At the same time, we recognize that Glut1 is unlikely to be the sole pathway, and that other mechanisms, such as nonspecific endocytosis, may also be involved. Accordingly, we have revised the manuscript to state that DHA modification promotes BBB penetration of nanoparticles primarily via Glut1-related mechanisms, but not exclusively.

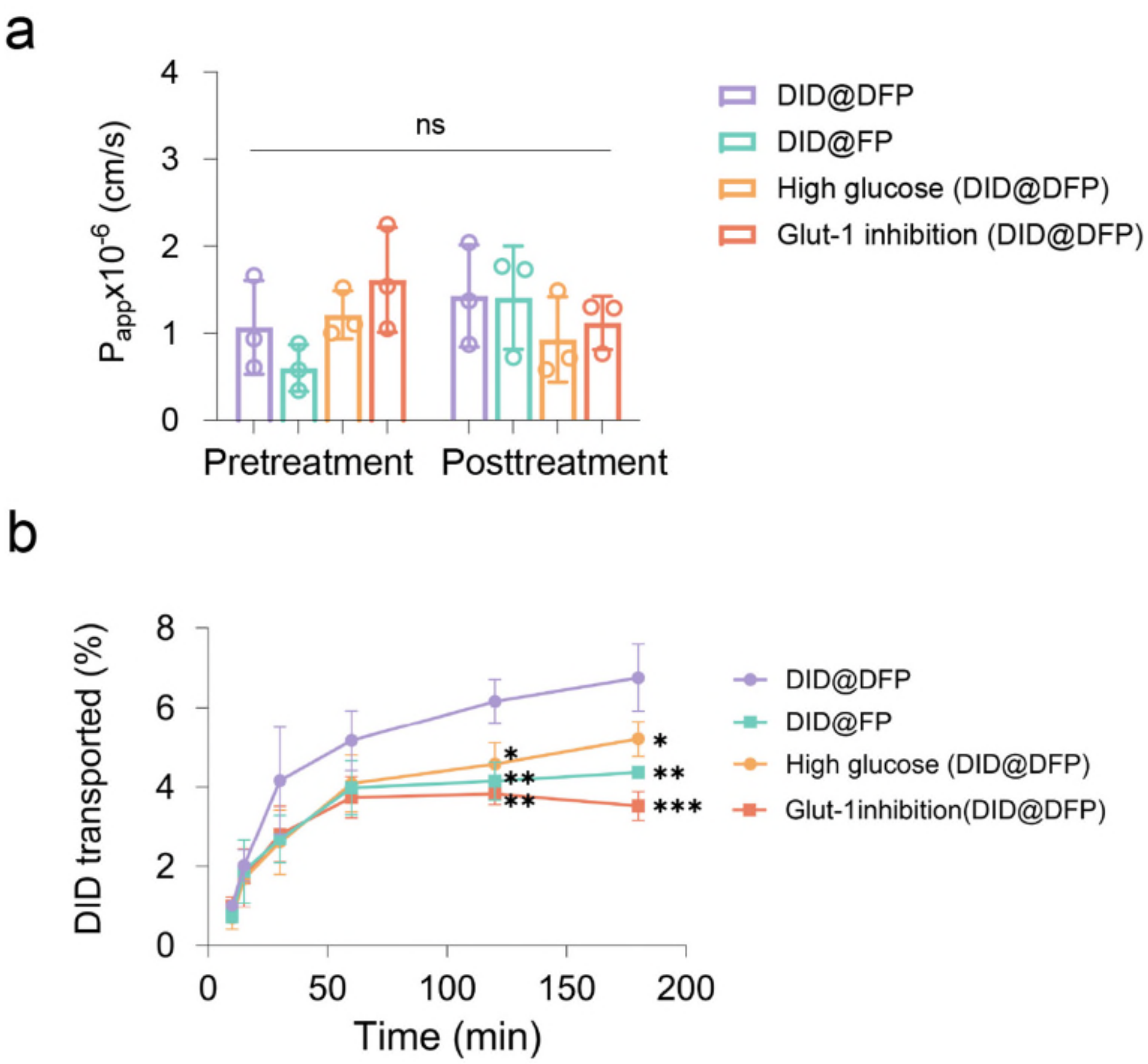

Figure R8. a) Changes in permeability coefficients of bEnd.3 cells before and after various treatments (n = 3 independent samples); b) DiD penetration ratios across different groups (n = 3 independent samples).

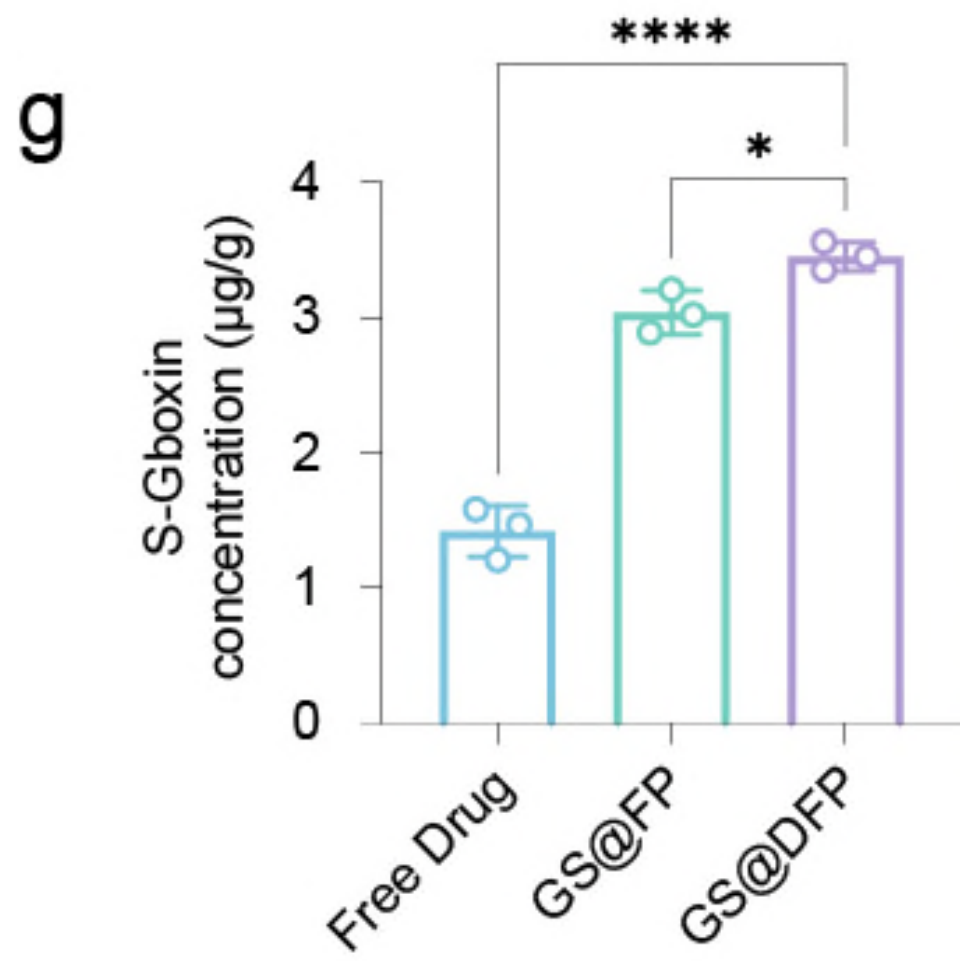

Figure R2. g) Distribution of S-Gboxin in tumor tissue under different treatment groups (n = 3 independent samples)

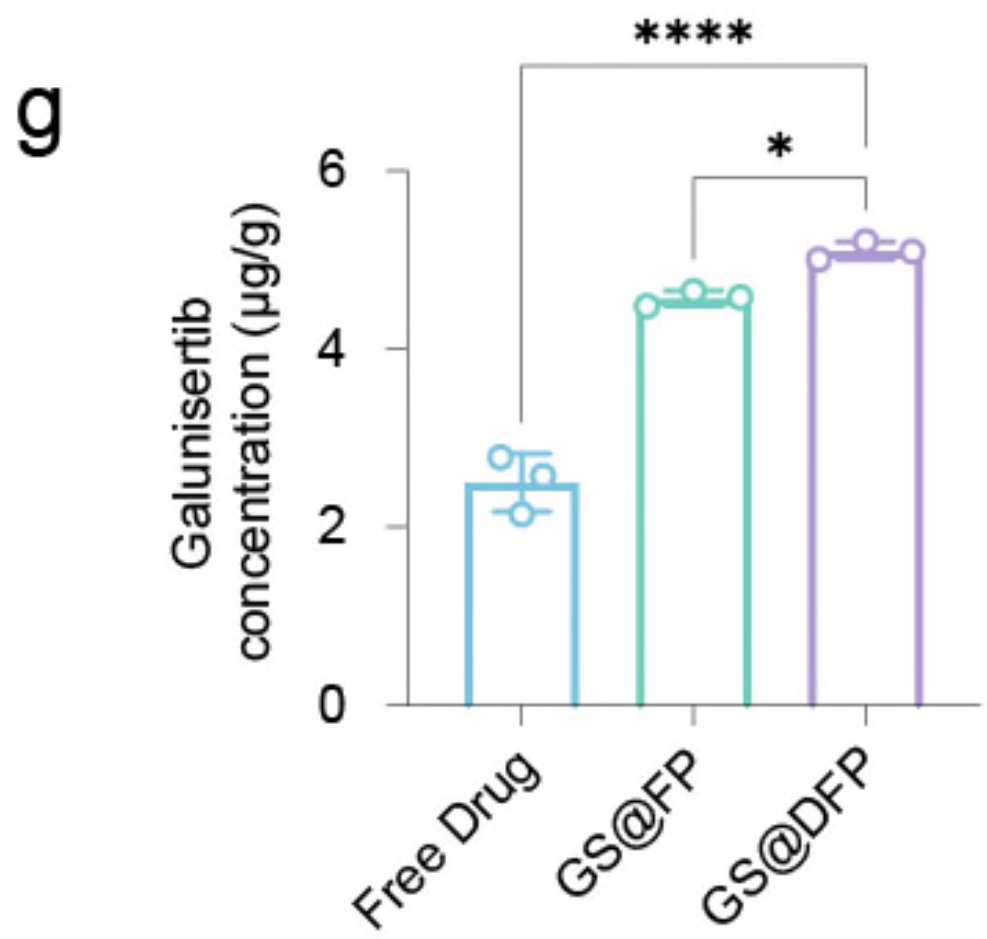

Figure R3. g) Distribution of Galunisertib in tumor tissue under different treatment groups (n = 3 independent samples)

Meanwhile, at the 6 h of the in vivo distribution experiment,, the tumor accumulation of the formulated dual-drug system was approximately 2.2-fold higher than that of the free drug group. Meanwhile, nonspecific distribution in non-target organs was reduced. We also observed that, compared with the non-modified group, the DHA-modified group exhibited relatively increased tumor accumulation of both drugs, directly indicating the important role of DHA modification in enhancing tumor-targeted accumulation.

In summary, the newly added competition and Glut1 inhibition experiments substantially strengthen the mechanistic basis of this study and directly address the reviewer's concern regarding whether Glut1 is strictly required.

• Ferrocene-mediated Fenton-like reactions play a central role in ROS amplification and drug release. However, potential off-target oxidative stress in normal brain tissue is insufficiently addressed: elevated ROS levels may damage neurons or normal astrocytes, especially given the brain's vulnerability to oxidative stress.

Response: We thank the reviewer for raising concerns regarding potential off-target oxidative stress. As noted, the ferrocene-mediated Fenton-like reaction is central to the ROS self-amplification and drug release behavior of this nanoplatform. However, brain tissues are highly sensitive to ROS, particularly neurons and endothelial cells, in which intracellular ROS homeostasis is critical for maintaining mitochondrial function, membrane potential, neuronal signaling, and cell viability. Excessive ROS can lead to oxidative damage, DNA breaks, and apoptosis; therefore, potential ROS-mediated off-target effects must be rigorously evaluated in brain-targeted therapies.

To address this, we conducted systematic in vitro safety assessments, focusing on the differential ROS responses between tumor cells and normal brain cells upon treatment with blank nanoparticles. Specifically, blank nanoparticles (200 μg/mL) were incubated for 24 h with murine glioma cells (GL261), human neuroblastoma cells (SH-SY5Y), and brain microvascular endothelial cells (bEnd.3). Intracellular ROS levels were then quantitatively measured using fluorescent probes. The results show a significant increase in ROS levels in tumor cells following treatment, consistent with their high metabolic activity, elevated basal ROS levels, and increased electron leakage from the mitochondrial respiratory chain. In contrast, ROS levels in normal neuronal and endothelial cells remained largely unchanged. This finding suggests that the intrinsically higher ROS environment in tumor cells provides sufficient substrate for ferrocene-catalyzed Fenton-like reactions, thereby preferentially triggering ROS amplification within tumor cells and enabling selective cytotoxicity.

Furthermore, this experimental design mechanistically supports the specificity of the ferrocene-catalyzed process: upon cellular internalization, the higher hydrogen peroxide levels in tumor cells provide the necessary conditions for the Fenton reaction, resulting in rapid local ROS accumulation. In contrast, the relatively low hydrogen peroxide levels in normal brain cells prevent substantial ROS amplification even after nanoparticle uptake, thereby minimizing off-target damage. These results highlight the differential ROS responses between tumor and normal brain cells and provide direct experimental evidence supporting the safety profile of the nanoplatform.

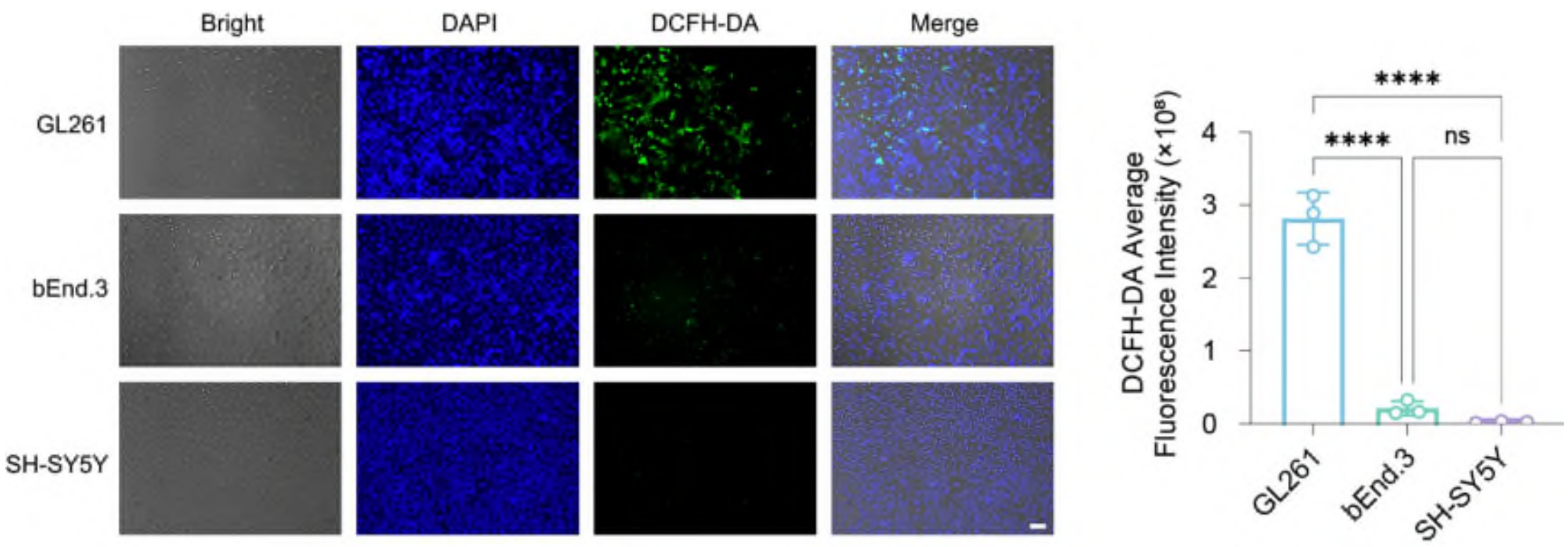

Figure R10. After treatment of different cell types with blank nanoparticles for 24 h, cells were stained with the ROS probe DCFH-DA and observed using an inverted fluorescence microscope, along with quantitative analysis (n = 3 independent fields of view), scale bar = 100 μm.

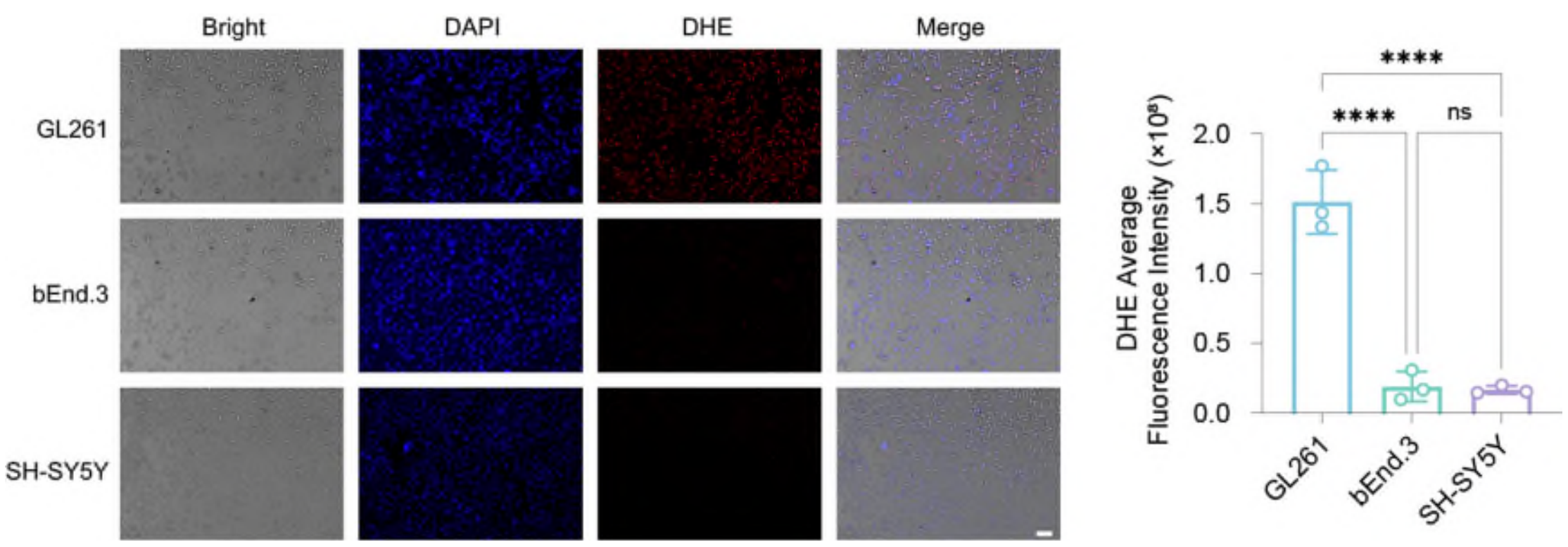

Figure R11. After treatment of different cell types with blank nanoparticles for 24 h, cells were stained with the ROS probe DHE and observed using an inverted fluorescence microscope, along with quantitative analysis (n = 3 independent fields of

view), scale bar = 100 μm.

In addition, the *in vitro* findings are consistent with our in vivo maximum tolerated dose (MTD) evaluation. Histological (H&E) analysis showed no evident tissue damage in brain tissues of mice treated with 2× or even 4× doses of nanoparticles, indicating favorable safety in non-tumor brain regions. This further addresses the reviewer's concern regarding potential off-target oxidative stress.

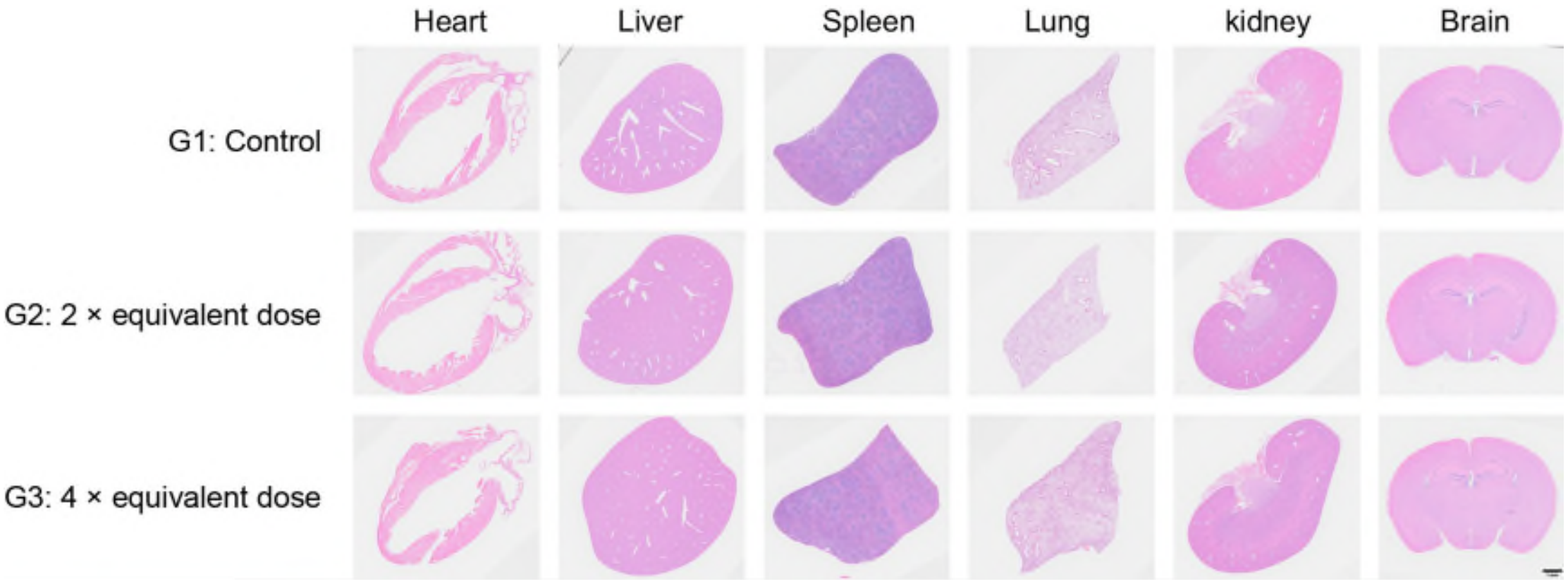

Figure R4. H&E-stained sections of major organs from mice in different treatment groups. Scale bar = 1 mm.

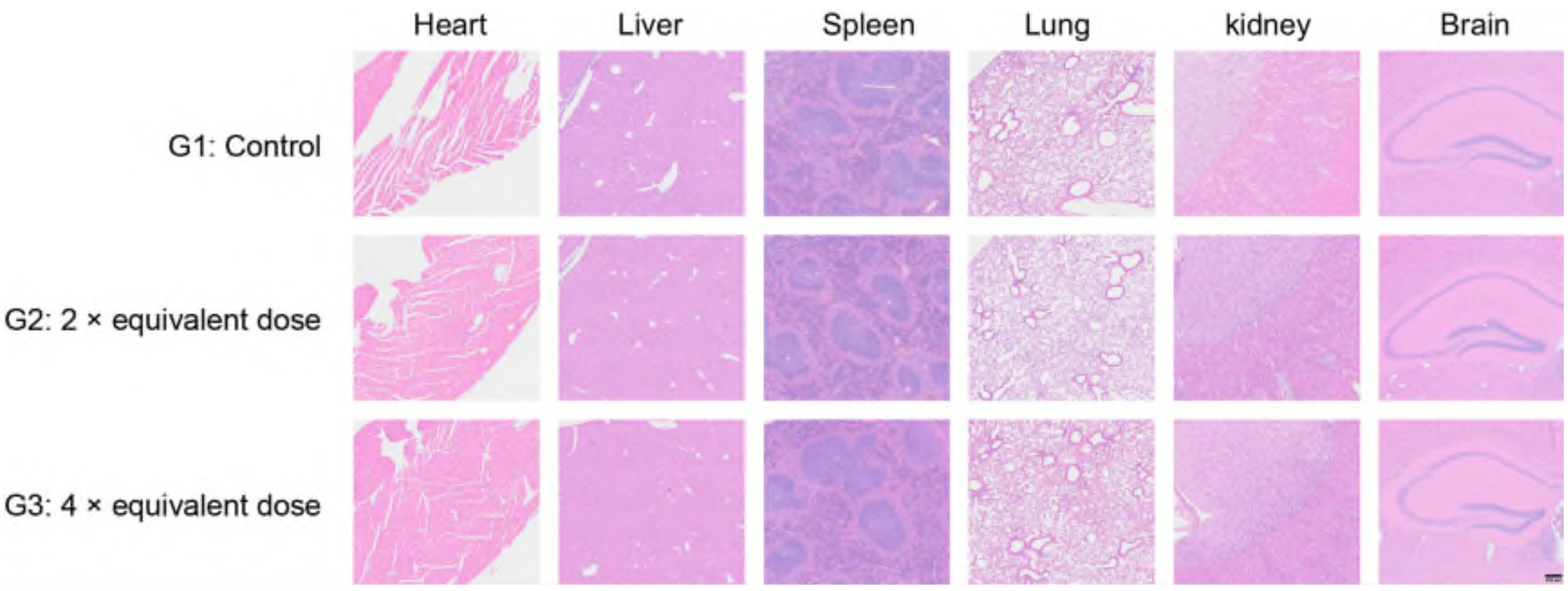

Figure R5. Magnified views of H&E-stained sections of major organs from mice in different treatment groups. Scale bar = 200 μm.

In summary, through systematic in vitro and in vivo validation, this study demonstrates differential ROS amplification effects between tumor and normal brain

cells, confirming tumor selectivity, ferrocene-catalytic specificity, and brain tissue tolerability, thereby providing strong experimental support for the safety of this nanoplatform.

• The manuscript emphasizes suppression of astrocyte-derived mitochondrial support but does not clearly distinguish between functional inhibition and direct astrocyte damage. It remains unclear whether GS@DFP selectively inhibits mitochondrial transfer mechanisms or induces astrocyte dysfunction/cytotoxicity.

<u>Response:</u> We thank the reviewer for raising this critical point regarding the distinction between “inhibition of mitochondrial transfer function” and “astrocyte damage.” We fully agree that, when investigating astrocyte-derived mitochondrial support, it is essential to clearly exclude potential interference from donor cell (astrocyte) dysfunction or cytotoxicity. In the present study, our experimental design inherently avoids direct effects of the nanoplatform on astrocytes: GS@DFP was used solely to pretreat tumor cells, which were subsequently co-cultured with untreated astrocytes. Therefore, astrocytes were not directly exposed to nanoparticles or drug-containing environments throughout the experiment, effectively excluding the possibility of direct cytotoxic damage from the experimental setup.

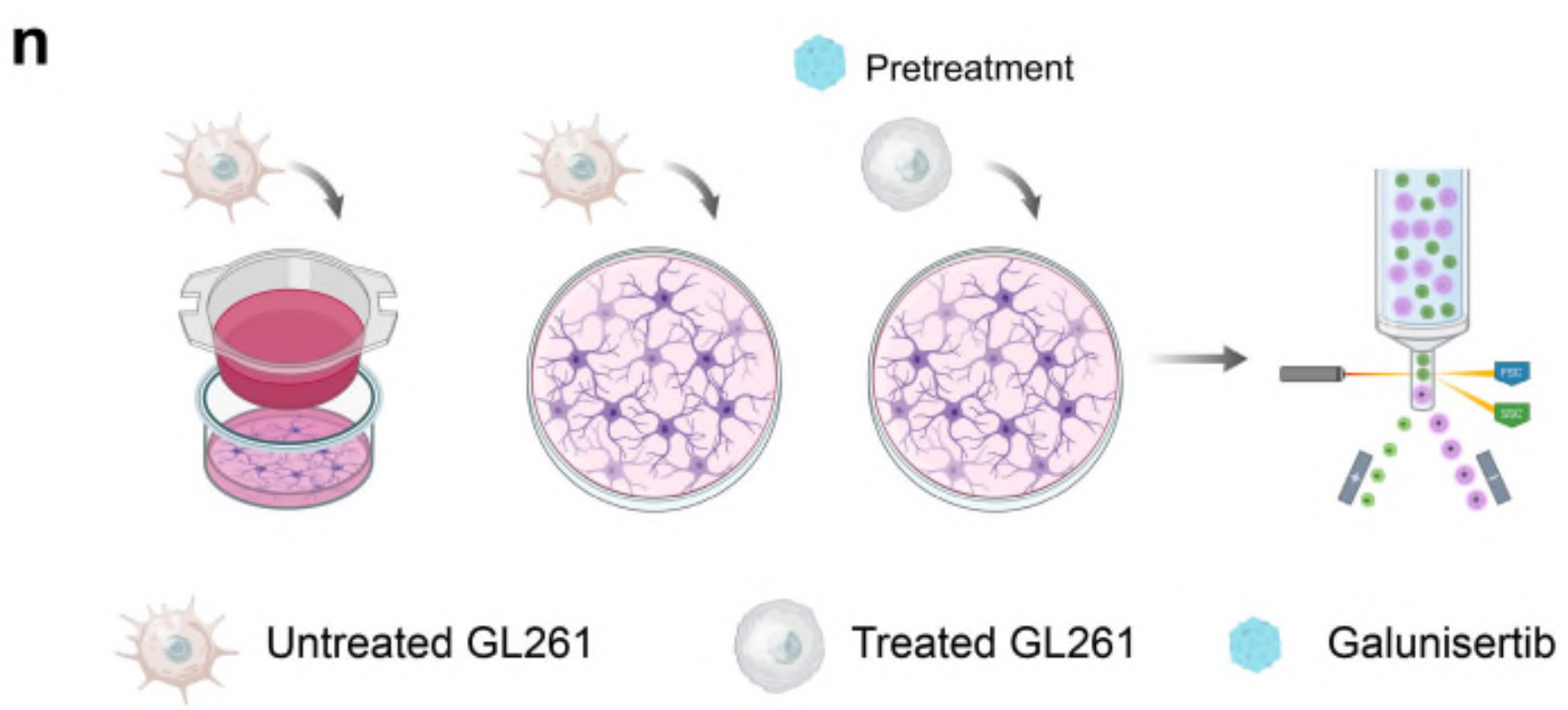

Fig. 1n. Schematic illustration of co-culture conditions.

Under these conditions, we observed a significant reduction in the acquisition of astrocyte-derived mitochondria by tumor cells, accompanied by alterations in tumor

cell-associated cycle. These findings suggest that GS@DFP primarily acts on tumor cells to suppress their ability to uptake or utilize exogenous mitochondria, rather than indirectly reducing mitochondrial supply via astrocyte impairment. Mechanistically, this effect is more consistent with a "recipient-side regulation" model, in which the capacity of tumor cells to actively receive mitochondrial transfer is diminished, rather than being a consequence of compromised donor cell (astrocytes) function. In addition, no apparent morphological abnormalities were observed in astrocytes within the co-culture system, further supporting that their function remained largely unaffected.

Based on the above experimental design and findings, we conclude that GS@DFP primarily exerts functional inhibition of mitochondrial transfer by suppressing the uptake and/or utilization of astrocyte-derived mitochondria by tumor cells, rather than inducing cytotoxicity or functional impairment in astrocytes. This clarification can clearly distinguish between "mechanistic inhibition" and "cellular damage," thereby addressing the reviewer's concern.

• The authors attribute immune activation primarily to TGF-β pathway inhibition. However, mitochondrial disruption and ROS generation may also contribute to immune remodeling.

Response: We thank the reviewer for raising this important point. We agree that, in addition to TGF-β pathway inhibition, mitochondrial dysfunction and ROS generation may also contribute to tumor immune remodeling.

In this study, we consider inhibition of the TGF-β pathway as the one of the mechanism underlying immune activation, based on the following considerations:

(1) TGF-β signaling plays a well-established central role in the immunosuppressive tumor microenvironment, and its downstream SMAD/TSP-1 axis is closely associated with immune cell suppression;

(2) We observed significant reversal of immunosuppressive markers following treatment, which is highly consistent with modulation of the TGF-β signaling

pathway.

At the same time, we acknowledge the reviewer's point that mitochondrial damage and increased ROS levels may act as contributing factors in immune regulation. Previous studies have shown that ROS accumulation and mitochondrial dysfunction can enhance antitumor immune responses by inducing immunogenic cell death or modulating inflammatory signaling pathways. In the present study, ferrocene-mediated ROS amplification and mitochondrial perturbation may, to some extent, increase the immunogenicity of tumor cells, thereby working in concert with TGF-β pathway inhibition to promote remodeling of the immune microenvironment. Accordingly, in the revised manuscript, we have refined our statements to avoid overemphasis on a single mechanism. The immune activation observed in this study is now described as being primarily driven by TGF-β pathway inhibition, with ROS generation and mitochondrial dysfunction acting as auxiliary, synergistic mechanisms.

In the revised manuscript (**lines 484–496**), we have added relevant discussions:

The ability of GS@DFP to simultaneously target mitochondria transferred from astrocytes and the hyperactive tumor mitochondria driven by GBM's high metabolic demands is central to its therapeutic efficacy. GBM, as one of the most aggressive brain tumors, exhibits strong network-forming behavior and can co-opt surrounding neuronal and astrocytic ecosystems, including the highly malignant process of mitochondrial transfer. Galunisertib attenuates astrocyte-derived support by inhibiting tumor microtubules and disrupts the TGF-β-driven immunosuppressive core of the TME. In addition to TGF-β pathway inhibition, mitochondrial disruption and ROS generation also contribute to immune remodeling, acting in parallel to reinforce the antitumor immune response. Meanwhile, although intense metabolic activity promotes tumor evolution, it also exposes metabolic vulnerabilities. As a selective ATP synthase inhibitor, S-Gboxin readily disrupts tumor mitochondria and demonstrates substantially greater cytotoxicity than TMZ. Although mitochondrial function is essential for immune cells, the tumor-targeted and ROS-responsive nature

of GS@DFP restricts this effect predominantly to tumor regions, thereby preserving overall immune competence. The ROS-responsive polymer nanoparticles modified with a glucose analogue enable efficient co-delivery of both therapeutics. After crossing the BBB, GS@DFP releases its payload in response to elevated ROS levels, achieving precise targeting of both tumor mitochondria and mitochondria acquired via intercellular transfer.

In summary, we consider the observed immune remodeling effect to result from the coordinated action of multiple mechanisms, with TGF-β pathway inhibition playing a dominant role and ROS- and mitochondria-related effects providing supportive enhancement. This revision offers a more comprehensive and balanced mechanistic interpretation, consistent with the current understanding of the complexity of tumor immune regulation.

• The survival extension observed in GS@DFP-treated mice is notable; however, the survival curves show partial, not complete, tumor control: in this regard, claims such as "paralyzing the GBM mitochondrial network" may overstate the therapeutic effect.

Response: We thank the reviewer for the careful and critical evaluation of our results. We agree that, although GS@DFP treatment shows a significant survival benefit, the survival curves indicate that complete tumor control has not been achieved. Therefore, expressions such as "paralyzing the GBM mitochondrial network" in the original manuscript may indeed represent a certain degree of overstatement.

In the revised manuscript, we have thoroughly reviewed and refined the relevant descriptions, replacing overly absolute language with more cautious and objective wording. At the same time, we further emphasize that the main contributions of this study are as follows:

(1) Proposing and validating a combinatorial intervention strategy targeting both mitochondrial function and astrocyte-derived mitochondrial transfer;

(2) Demonstrating that this strategy significantly prolongs survival and mitigates tumor progression in vivo;

(3) Elucidating its potential mechanisms in metabolic regulation and immune microenvironment remodeling.

In the revised manuscript (lines 64 – 66), we have changed the explanation:

S-Gboxin subsequently disrupts intrinsic mitochondria, while Galunisertib blocks extrinsic mitochondrial transfer—together paralyzing the GBM mitochondrial network.

S-Gboxin subsequently disrupts intrinsic mitochondria, while Galunisertib blocks extrinsic mitochondrial transfer, together resulting in a substantial disruption of the GBM mitochondrial network.

Accordingly, we have consistently revised the manuscript to avoid expressions that may be interpreted as "complete inhibition" or "fundamental blockade," ensuring that all conclusions are strictly aligned with the experimental data.

Reviewer #3:

In this study, the authors designed a nanoparticle-based dehydroascorbic acid (DHA)-functionalized, ROS-responsive, ferrocene-integrated polymer nanoplatform (GS@DFP) co-loaded with S-Gboxin and Galunisertib, aiming to simultaneously target GBM mitochondria and astrocyte–cancer cell mitochondrial transfer. This is a translational study and focuses on evaluating the therapeutic efficacy of the proposed nanodrug. Authors provides a new combination strategy, and this is the major novelty of this study. Based on authors' results, the therapeutic effect seems promising. Collectively, this study is promising and potentially important in developing effective treatment for cancers. However, a major concern for this study is the scientific rational of this work that the authors provided. The rationale is based on very few literatures, which make it looks questionable and is insufficient. Based on current study, it is difficult to comprehend that the promising therapeutic effect of targeting both mechanisms (GBM mitochondria and astrocyte–cancer cell mitochondrial transfer) can be attirbuted to the very reason claimed by authors. It is possible that the underlying mechanisms of two mechanisms are totally irrelevant or other mechanisms may also be involved. To support or strengthen the hypothesis and conclusion claimed by the authors, strong and convincing evidence/data are needed. Additionally, the scientific rigor needs to be improved. The current enthusiasm for this manuscript is moderate.

Major Comments:

1. The major concern from this reviewer is the rationale for this study. Here are the detailed comments.

(1) Reference 8 is an abstract from a meeting without a manuscript in fall length and a peer review, which is inappropriate as key evidence for its scientific premise.

<u>Response:</u> Regarding Reference 8: We thank the reviewer for pointing out the issue with the cited reference. We fully agree that the conference abstract cited in the original manuscript is not appropriate as key supporting evidence. We also fully acknowledge the reviewers' comments regarding this reference. To ensure the rigor of

the manuscript, we have removed the original literature and updated it to the latest peer-reviewed supporting literature (**Neuro Oncol. 2026 Feb 23:noag038. Nat Rev Cancer 22, 481－491 (2022).**) for better literature support.

It is also worth noting that research on mitochondrial transfer in glioblastoma is still in its early stages, and available peer-reviewed literature remains very limited. This gap in knowledge is precisely what underscores the scientific value of our study—we aim to explore this emerging mechanism rather than relying solely on extrapolation from limited existing reports.

And to address this, we have deliberately established a comprehensive and systematic experimental framework in this study to investigate the process from multiple levels. Specifically, our analyses include bioinformatics pathway enrichment, visualization of tumor microtube structures, molecular regulation of tumor microtube-associated proteins, functional phenotypic assays, and direct assessment of mitochondrial transfer from astrocytes to tumor cells, as well as evaluation of its downstream effects on the cell cycle. Through these multi-level and complementary approaches, we have constructed a coherent and mutually validated body of evidence, providing robust support for understanding the mechanism of mitochondrial transfer and highlighting the novelty and scientific significance of this work.

(2) In lines 50–53, the authors claim that S-Gboxin fails to block astrocyte-derived mitochondrial compensation, but they did not provide convincing evidence or appropriate reference. One would presume that the conclusion is based on references 10 or 11. Studies in these two reports are insufficient to support for this conclusion. It is imperative that the authors provide direct evidence or supporting references. Moreover, it is also questionable that an ATP synthase inhibitor would be unable to suppress astrocyte-derived mitochondria and mitochondrial compensation.

<u>Response:</u> We thank the reviewer for the valuable comment on this statement. We fully agree that the original claim that “S-Gboxin cannot block astrocyte-derived mitochondria or compensatory mechanisms” was an unverified speculation, lacking direct experimental evidence and sufficient literature support, which was an oversight

on our part. The background for this statement was as follows: mitochondrial transfer can indeed enhance tumorigenicity, and merely inhibiting the existing mitochondrial function of tumor cells might not be sufficient to prevent this process, which led us at the time to theoretically speculate on the limitations of S-Gboxin.

In the revised manuscript (**lines 50–53**), we have removed the unsupported statement and the IC_{50} comparison, and replaced it with a more accurate and evidence-based description:

~~S-Gboxin exhibits approximately 200-fold lower IC50 than TMZ in GL261 cells, but fails to block astrocyte-derived mitochondrial compensation resulting in tumor recurrence and increased proliferative capacity in orthotopic models.~~

S-Gboxin is a potent inhibitor of oxidative phosphorylation in tumor cells. However, tumor-microenvironment interactions may influence its therapeutic efficacy, suggesting the need for combinational strategies.

We understand that the original statement could have caused misunderstanding. The revised manuscript corrects this while ensuring that the core experimental design, results, and conclusions of the study remain unaffected. We sincerely apologize for this oversight and thank the reviewer for the guidance, which helps improve the scientific rigor of the manuscript.

(3) Only single publication is used to support the role of TGF-β in regulating tumor microtubes and mitochondrial transfer, which is insufficient to establish a strong rationale. Given the well-known roles of TGF-β in immunology and many other biological processes, regarding mitochondrial transfer as the main effect of TGF-β signaling and using it as a central rationale is not convincing. In addition, in line 55, reference 12 only reports the use of a TGF-β pathway inhibitor but does not examine mitochondrial transfer phenotypes. Overall, the involvement of the TGF-β pathway in mitochondrial transfer is insufficiently supported and the data of mitochondrial transfer inhibition is the primary effect of TGF-β pathway inhibitors needs additional supporting evidence. It remains possible that the major therapeutic effect observed in

this study is unrelated to mitochondrial transfer.

Response: We thank the reviewer for raising the important issue regarding the limited evidence for TGF-β's role in tumor microtube formation and mitochondrial transfer. We agree that current research directly linking TGF-β signaling to regulation of tumor microtubes (**Nat Rev Cancer 22, 481 - 491 (2022). Nature 617, 599 - 607 (2023).**) and mitochondrial transfer (**Neuro Oncol. 2026 Feb 23:noag038.**) is limited . However, from a research strategy perspective, we selected the TGF-β/SMAD/TSP-1 pathway as an intervention point primarily because of its aberrant activation in tumor tissue and its favorable pharmacological accessibility, rather than assuming it as the only regulatory mechanism. Direct interventions targeting tumor microtube structures mainly fall into two categories:

1. Directly targeting structural proteins such as GAP43.

However, GAP43 plays a critical role in neural development and axon guidance, and its widespread expression in the normal nervous system could lead to significant off-target risks, potentially disturbing neuronal structural stability and plasticity, with associated neurotoxicity or developmental side effects.

2. **Intervening via the cytoskeleton (e.g., actin) to disrupt tumor microtubes.**

While mechanistically feasible, such approaches often have high systemic toxicity due to the ubiquitous roles of actin in cell migration, division, and basic physiological activities. For example, actin-disrupting drugs can effectively inhibit cytoskeleton remodeling but have narrow therapeutic windows, limiting their in vivo applicability.

Although nanoparticle delivery could partially reduce toxicity or improve distribution, targeting aberrantly activated TGF-β pathways in tumor tissue offers better disease-context specificity, allowing regulation of tumor microtube processes while minimizing effects on normal neural structures. Additionally, TGF-β contributes to maintaining the immunosuppressive tumor microenvironment, so its inhibition may provide extra immune-modulatory benefits, which was another key reason for selecting this pathway.

we have systematically modified the relevant statements to clearly emphasize that TGF-β signaling is one of the contributing factors in this process, rather than the sole

or decisive factor, to avoid overinterpretation.

In the revised manuscript (lines 55–57)

~~Another strategy is TGF-β pathway inhibitors like Galunisertib, which downregulates TSP-1 by inhibiting SMAD2/3 phosphorylation, reducing MT formation and mitochondrial transfer~~

Another strategy involves TGF-β pathway inhibitors such as Galunisertib, which suppress SMAD2/3 phosphorylation and downregulate TSP-1, reducing MT formation and mitochondrial transfer. Compared with directly targeting axonal proteins like GAP43 or using cytokinins to disrupt the cytoskeleton, modulating the TGF-β-SMAD-TSP-1 pathway offers a safer and more feasible intervention point among these various options.

Within the context of the above strategy, to address the reviewer's concern regarding the "lack of direct evidence demonstrating TGF-β regulation of mitochondrial transfer," we have supplemented the revised manuscript with an exogenous mitochondrial rescue experiment to provide direct functional validation. Specifically, mitochondria were isolated from astrocytes, and we first confirmed that the mitochondria extracted from astrocytes were functionally active.

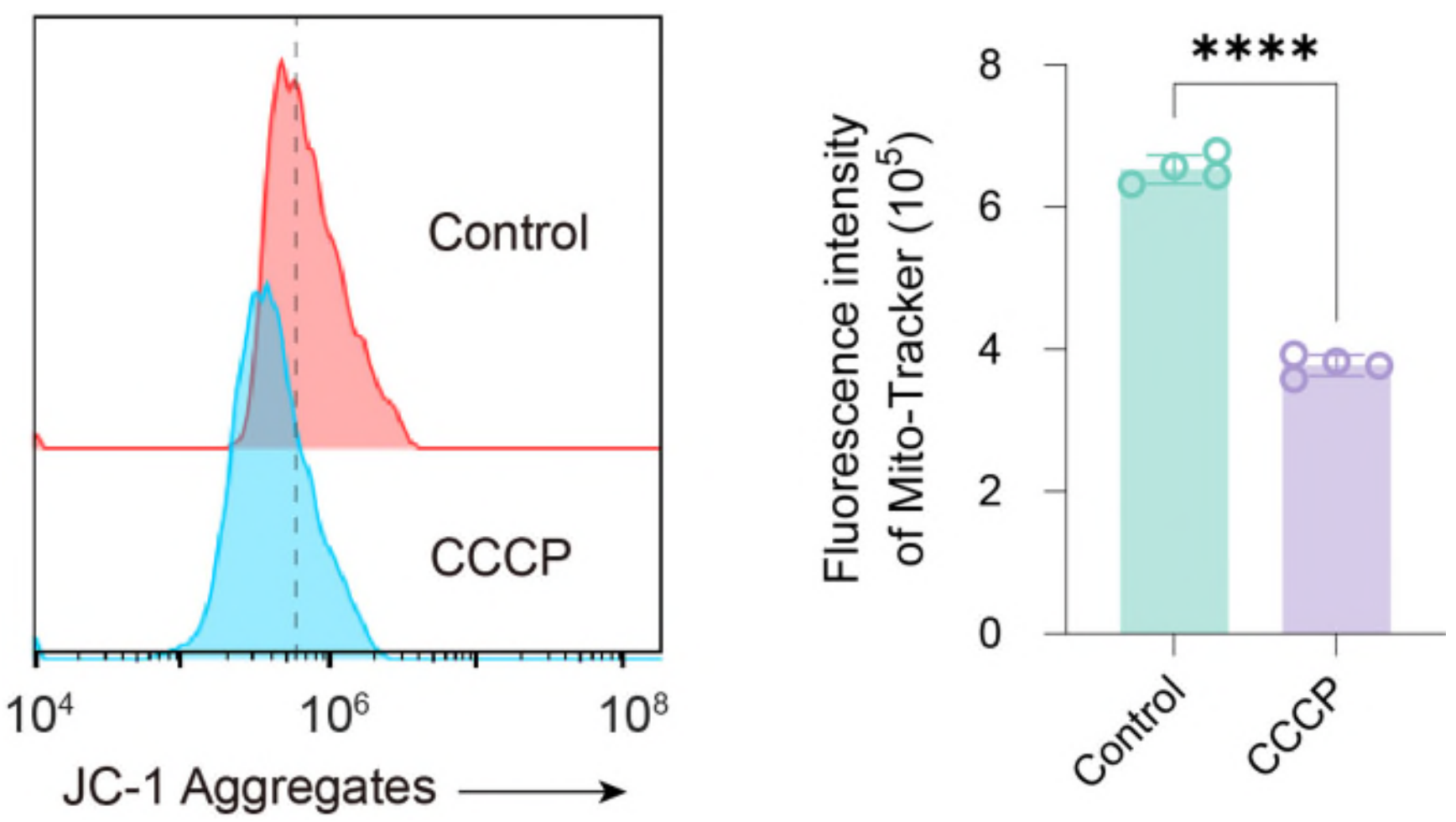

Figure R12. Flow cytometry schematic and quantitative results of mitochondria extracted from astrocytes after CCCP treatment (n = 3 independent samples). ****p < 0.0001.

These mitochondria were then co-incubated with tumor cells at a concentration of 50 μg/mL for 12 hours. Using confocal microscopy and flow cytometry, we verified that GL261 cells were capable of internalizing free astrocyte-derived mitochondria.

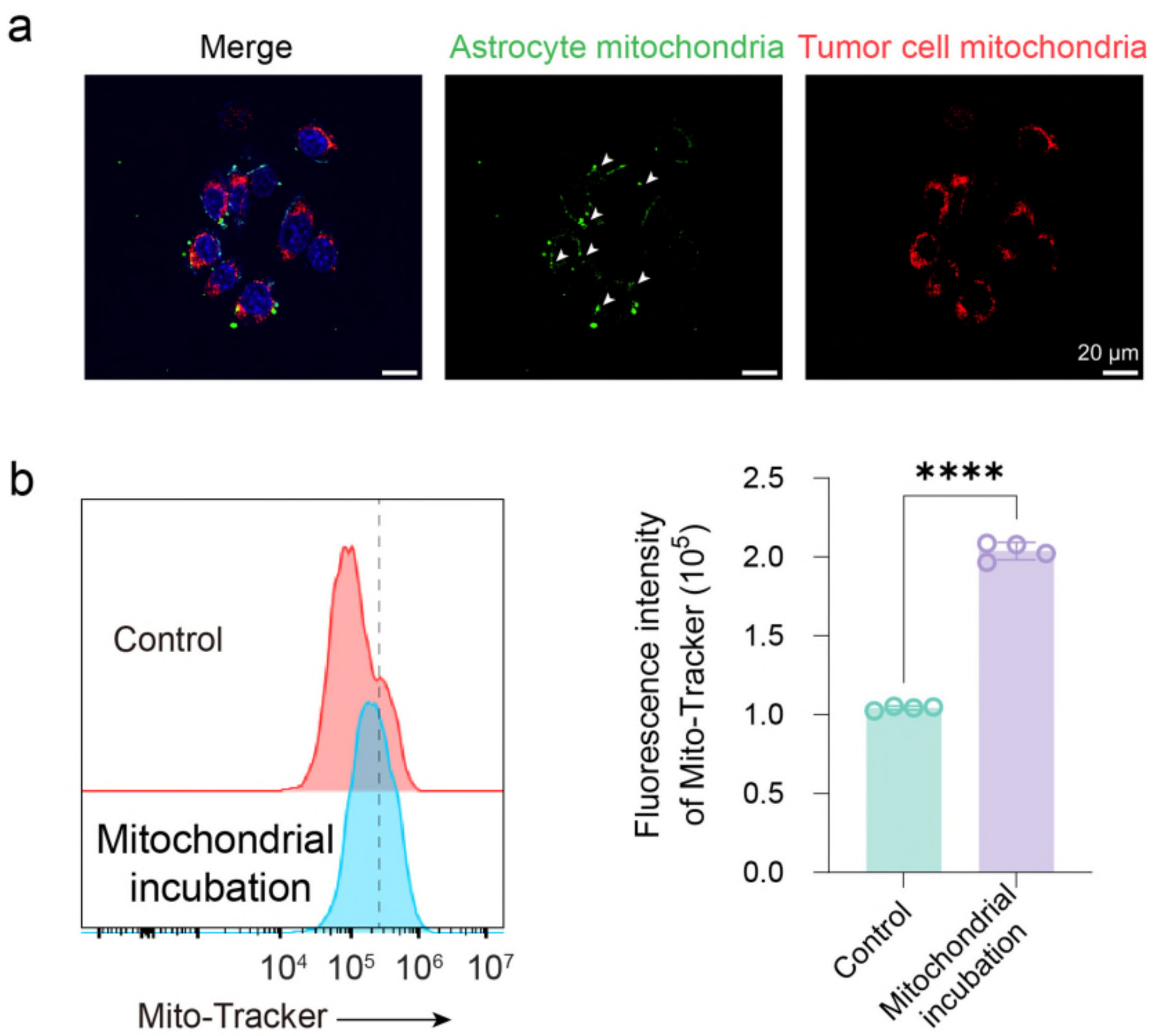

Figure R13. a) Representative confocal images of astrocyte-derived mitochondria (Mito-Tracker Green) after 12 h incubation with GL261 cells pre-stained with Mito-Tracker Red; b) After 12 h incubation of GL261 cells with astrocyte-derived mitochondria, intracellular mitochondria in GL261 were stained with Mito-Tracker Red, and mean fluorescence intensity was measured by flow cytometry, n = 3 independent samples. ****$p < 0.0001$.

Based on this, we established three experimental conditions: a Control group, a Galunisertib-treated group, and a rescue group in which Galunisertib-treated cells were subsequently co-incubated with astrocyte-derived mitochondria. After co-culture, cell cycle distribution was analyzed.

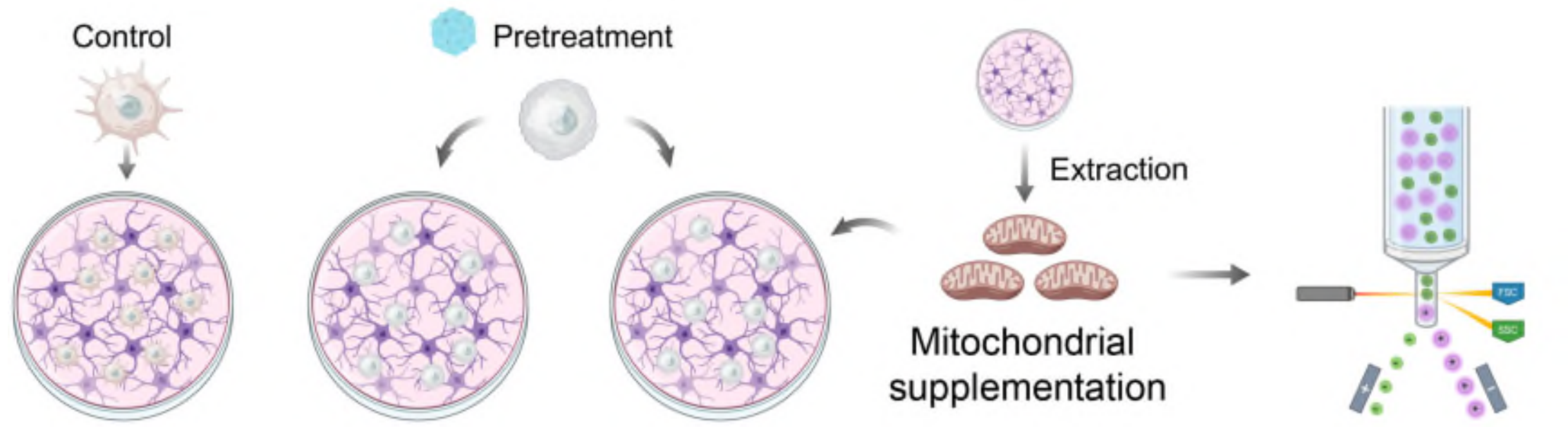

Figure R14. Schematic diagram of the mitochondrial transfer rescue experiment workflow

The results showed that under Galunisertib treatment, tumor cell proliferation was significantly inhibited, indicating that TGF-β pathway suppression affected tumor cell growth. In the rescue group, supplementation with exogenous astrocyte-derived mitochondria partially restored the cell cycle status of tumor cells.

These findings carry clear functional implications: restoration of exogenous mitochondrial supply can partially reverse the downstream phenotypes induced by TGF-β inhibition. Therefore, this phenomenon causally supports our conclusion that part of the biological effects resulting from TGF-β pathway inhibition depends on the transfer of mitochondria from astrocytes to tumor cells, providing direct experimental evidence for its role in regulating astrocyte–tumor cell mitochondrial transfer.

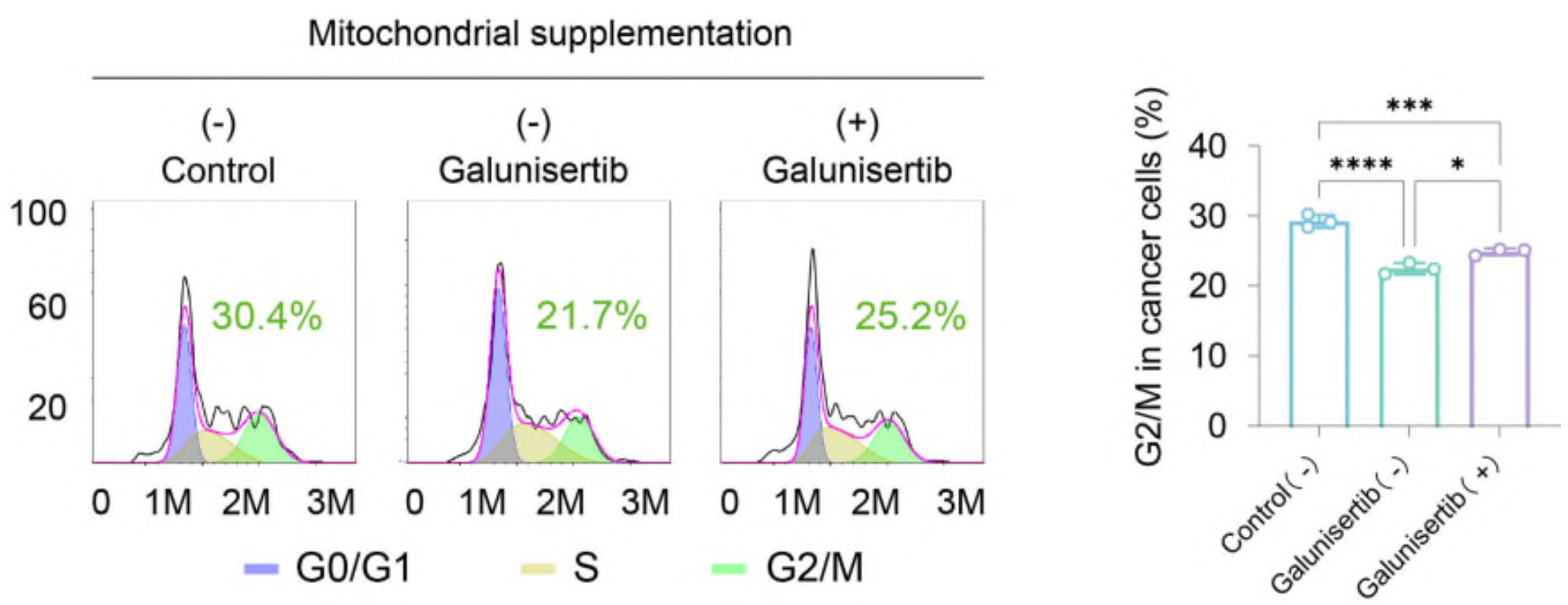

Figure R15. Representative cell cycle distribution of GL261 cells under three different treatments and the proportion of cells in G2/M phase (n = 3 independent samples). *$p < 0.05$, ***$p < 0.001$, ****$p < 0.0001$.

We also acknowledge that mitochondrial transfer is not the sole downstream output of TGF-β signaling, and its contribution to overall therapeutic effects likely acts in concert with other pathways, such as immune regulation or cell migration mechanisms.

In summary, although this field is still developing, the additional functional rescue experiments provide more direct experimental evidence for TGF-β's involvement in astrocyte-to-tumor mitochondrial transfer, while our revised statements carefully reflect the current mechanistic understanding.

(4) The authors combine multiple elements into a single drug design targeting different pathways. The rationale for this combination is weak. It is questionable about whether inhibition of mitochondrial transfer is indeed a key downstream effect of TGF-β inhibition, and whether the biological effects of inhibiting these two pathways (GBM mitochondria and astrocyte-cancer cell mitochondrial transfer) are mechanistically related. The authors should provide clear and supporting evidence demonstrating a true synergistic effect of simultaneously targeting GBM mitochondria and astrocyte-mediated mitochondrial transfer. In addition, they should show that the effects observed with single agents and their combination are on-target rather than the result of off-target effects.

Response: We thank the reviewer for this important and insightful comment regarding the rationale and potential synergistic effect of the combinational strategy and weather the combination are on-target.

(1) For the combinational strategy

First, we would like to clarify that this design is not intended as a simple combination of two independent pathways. Rather, it is based on a functionally coupled astrocyte–tumor cell donor–recipient system, in which astrocytes serve as mitochondrial donors and tumor cells act as recipients. Within this framework, the two agents target distinct but interdependent aspects of the same biological process.

Specifically, S-Gboxin inhibits oxidative phosphorylation in tumor cells, thereby

suppressing mitochondrial utilization on the recipient side. In parallel, Galunisertib modulates the TGF-β/SMAD/TSP-1 axis within tumor cells, reducing tumor microtube formation and consequently limiting the transfer of astrocyte-derived mitochondria. Importantly, both interventions act at the level of tumor cells, rather than independently targeting unrelated cell populations.

To demonstrate that the combinational strategy produces a coordinated and enhanced functional effect beyond single interventions, we performed the following analyses:

1) In the CCK-8 experiment, compared to free dual drugs, the formulated dual-drug system exhibited the lowest IC_{50} among all groups, indicating an enhanced therapeutic effect compared to single-agent treatments.

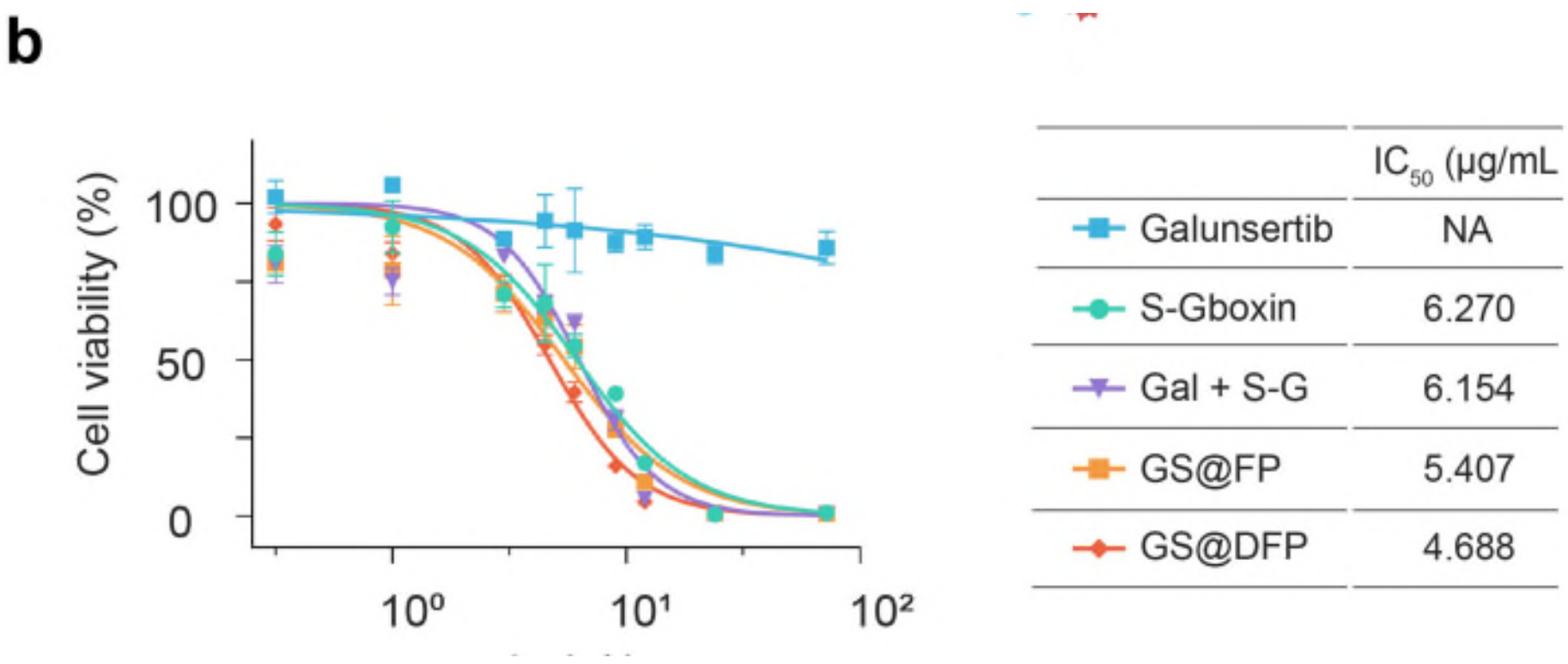

Fig. 5b. Fitted cell viability curves and half-maximal inhibitory concentrations (IC_{50}) of GL261 cells after treatment with Galunisertib, S-Gboxin, Galunisertib + S-Gboxin, GS@FP, or GS@DFP for 24 h (n=4 independent samples).

2) In co-culture systems, single treatments (either S-Gboxin or Galunisertib) led to only partial reductions in intracellular mitochondrial levels, whereas the combined treatment resulted in a significantly greater decrease.

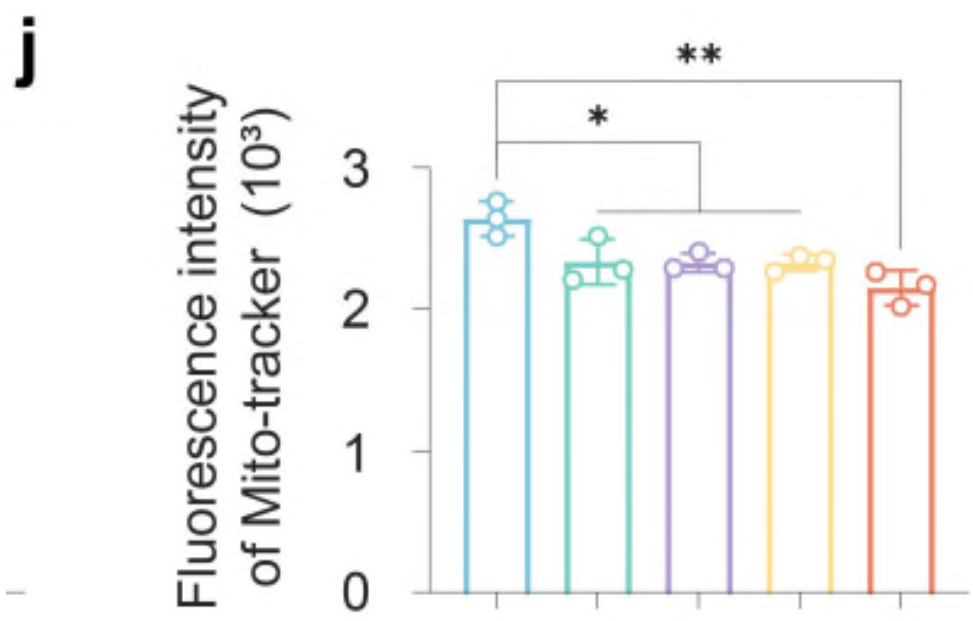

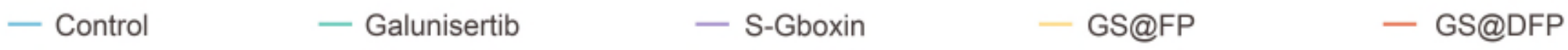

Fig. 6j. Flow cytometric quantification of mitochondrial content in ZS-green GL261 cells after co-culture with astrocytes (n=3 independent samples).

Similarly, in in vivo experiments, the dual-drug combination group exhibited the strongest tumor microtubule inhibitory effect, which also demonstrated the synergistic effect of the two drugs. We also assessed mitochondrial content in tumors of orthotopic ZS-green GL261 tumor-bearing mice after various treatments. Using Mito-Tracker Red to stain homogenized brain tissue, the cancer cells were distinguished by their green fluorescence through flow cytometry, and all three treatment groups showed reduced mitochondrial content. Gal@DFP reduced externally derived mitochondria through Galunisertib-mediated suppression of mitochondrial transfer, whereas S-G@DFP directly eliminated mitochondria originating from the cancer cells themselves via S-Gboxin. Consistent with our previous in vitro cell experimental results, tumors treated with GS@DFP, combining the dual-drug effect, exhibited the lowest mitochondrial content.

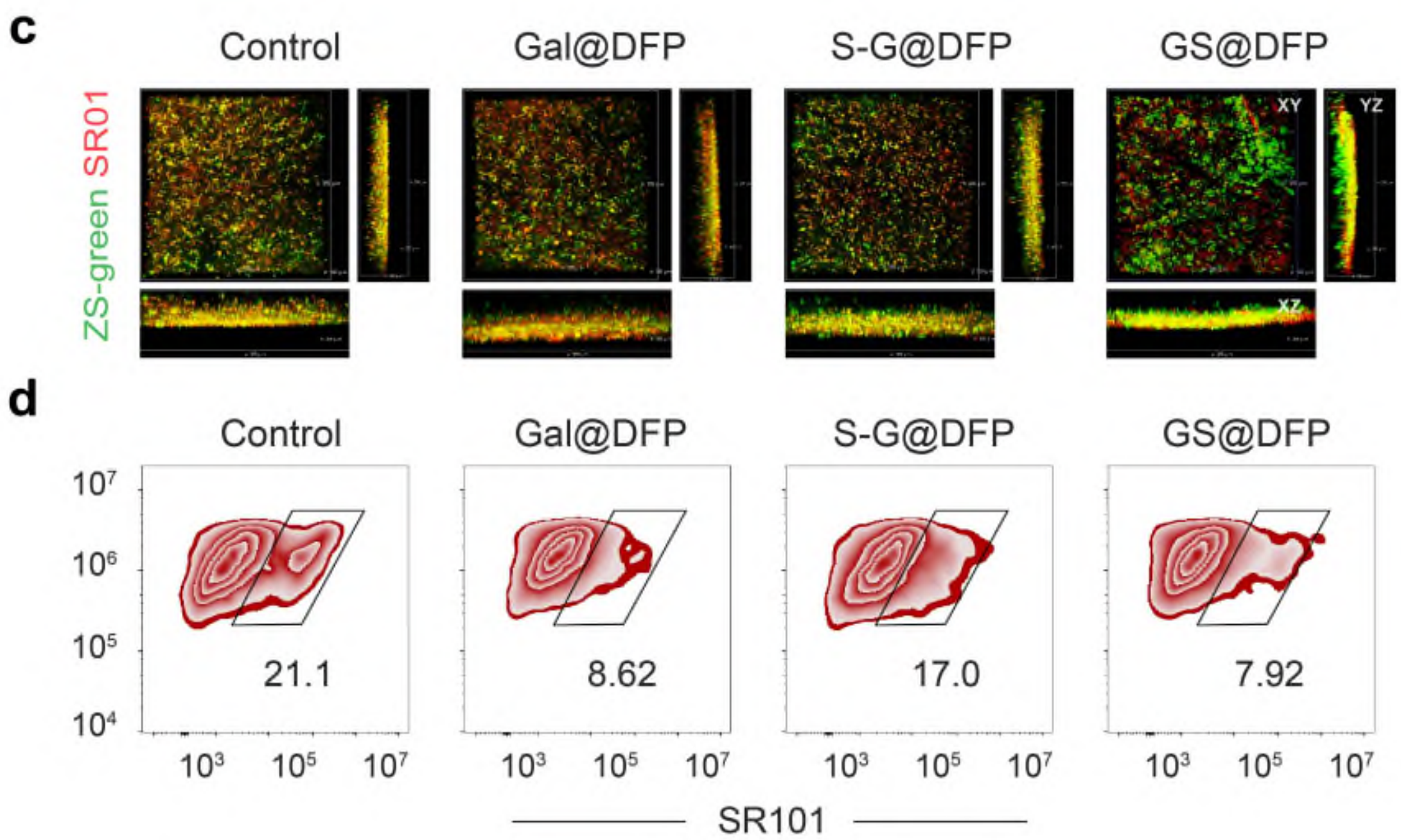

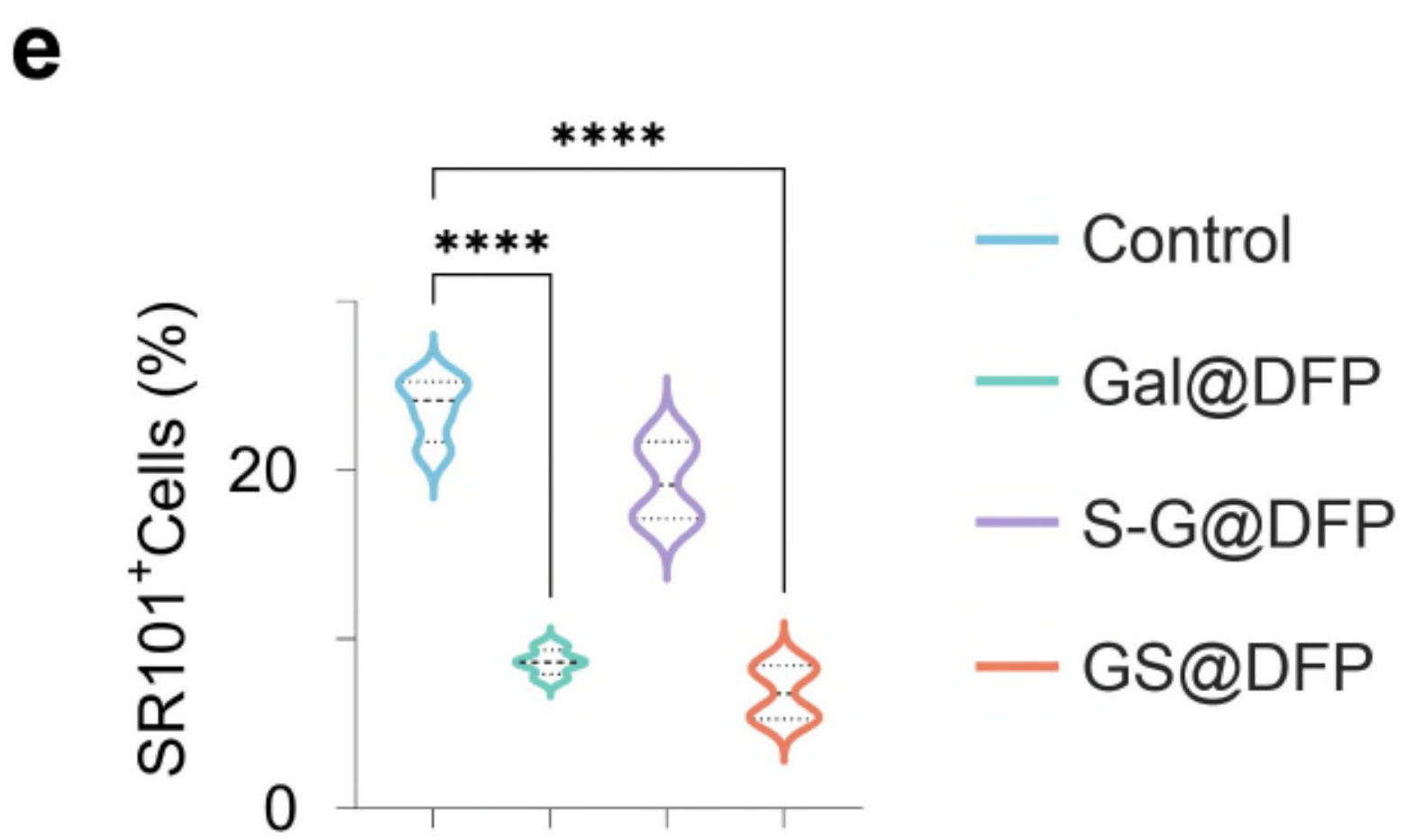

Figure 7c d e. c) *In vivo* observation of uptake of SR101 by cancer cells from astrocytes under two-photon microscopy; **d e)** Representative flow cytometry plots (**d**) and quantitative analysis (**e**) of $SR101^+$ cancer cells (n=4 independent samples);

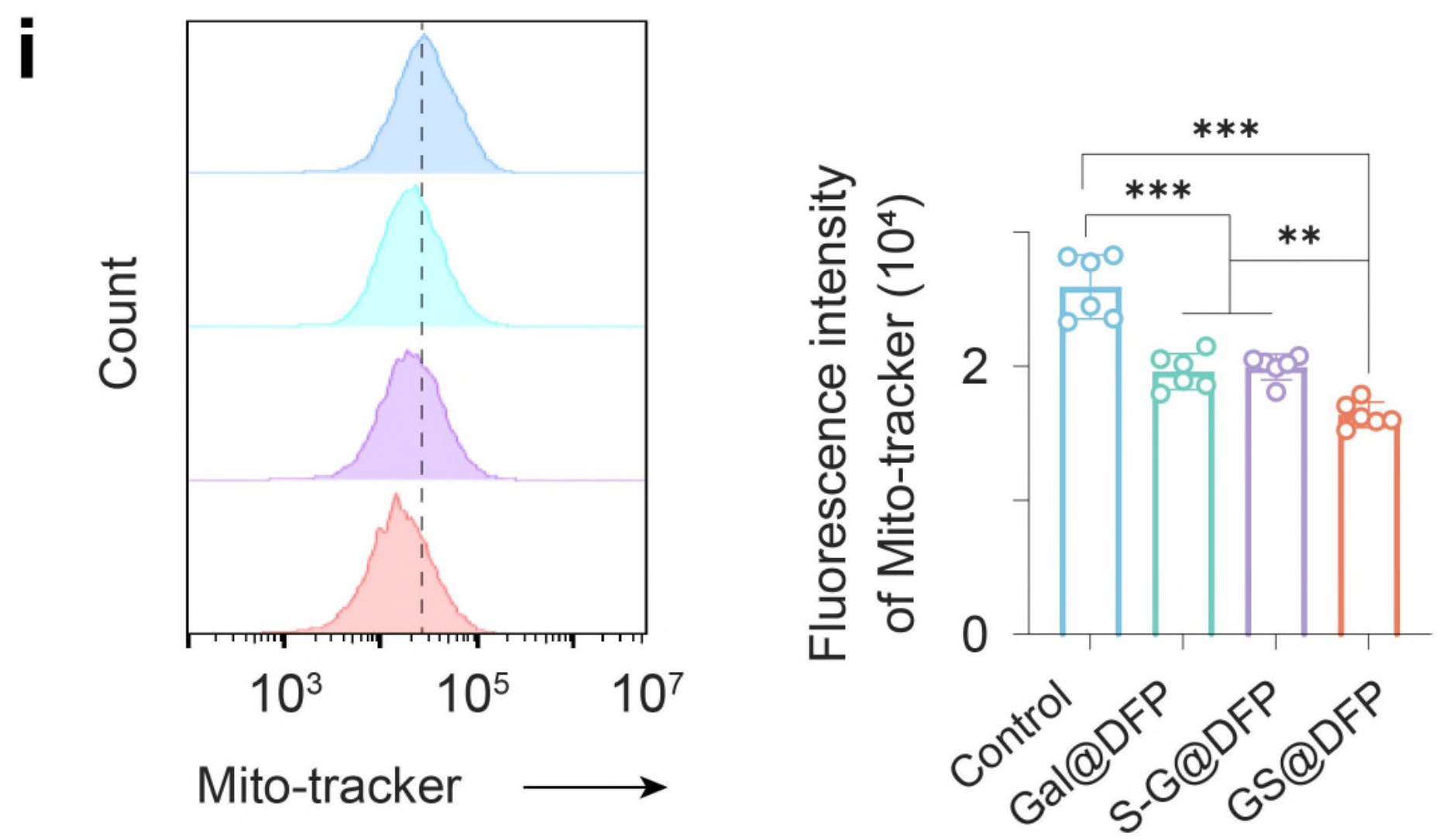

Figure 7i. Representative FCM plots and quantitative results of mitochondrial content in tumors from different treatment groups (n = 6 independent samples);

3) We have demonstrated in vitro that downregulating mitochondrial transfer suppresses malignant proliferation driven by metabolic reprogramming in cancer cells. We further investigated whether inhibition of mitochondrial transfer could also reduce astrocyte-mediated malignant tumor support. In tumor-bearing mice treated with Gal@DFP, Kiel 67 proliferation signals in tumor tissues were markedly

downregulated. S-G@DFP, through mitochondrial disruption, also suppressed tumor proliferation, while dual-drug GS@DFP treatment exhibited the lowest Ki67 signals . We further examined tumor apoptosis under different treatments, and all three treatment groups displayed enhanced pro-apoptotic activity. The downstream events of proliferation inhibition and apoptosis induction collectively demonstrate the superior antitumor efficacy of targeting tumor mitochondria and mitochondrial transfer strategies.

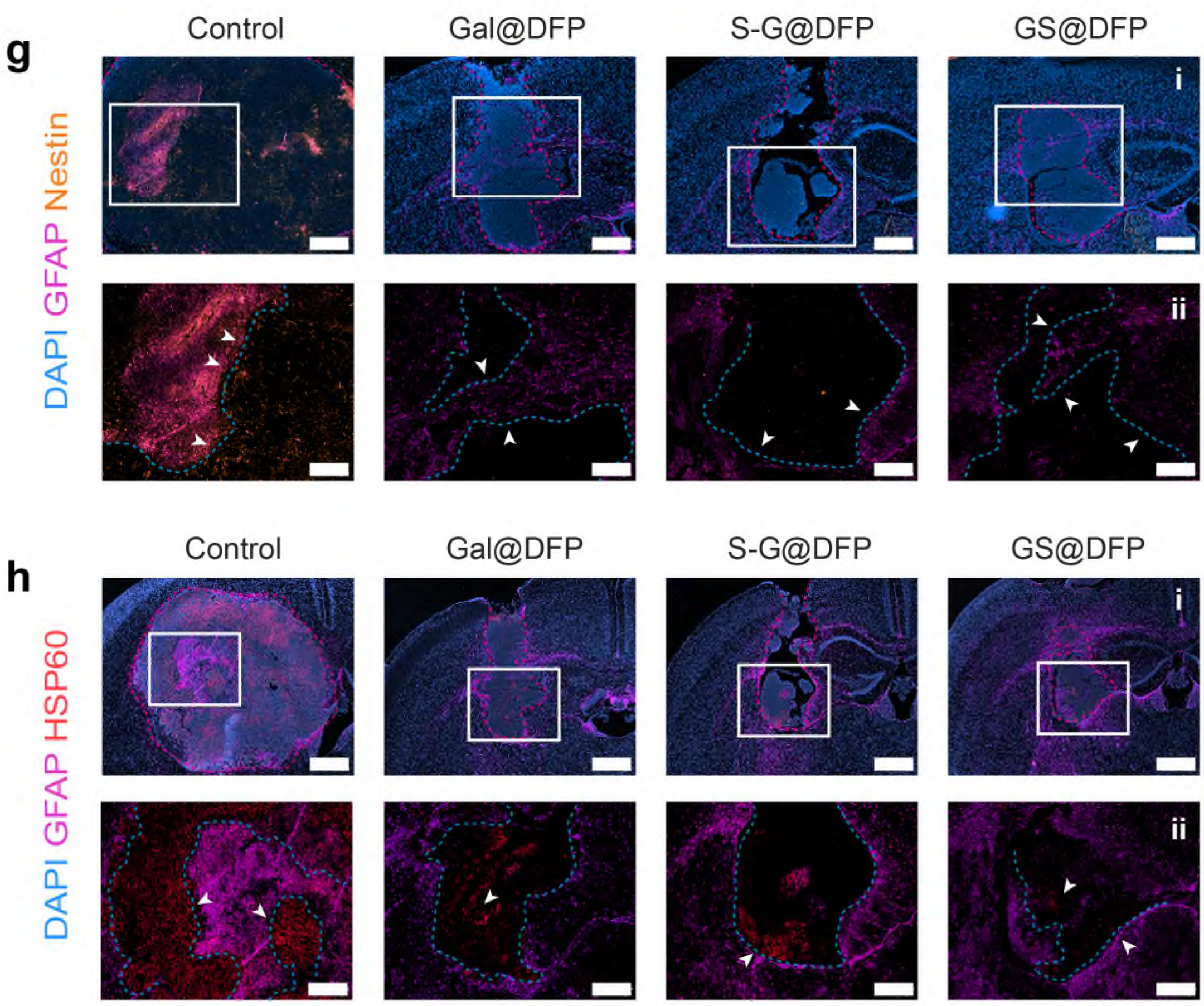

Fig.7g h. g Fluorescence staining analysis of the astrocyte and MT-associated protein Nestin; h Fluorescence staining analysis of the astrocyte and mitochondria-associated protein Heat Shock Protein 60 (HSP60); Tumor regions are indicated by red dashed lines in panel i (scale bar = 200 μm) and by blue dashed lines in panel ii (scale bar = 100 μm).

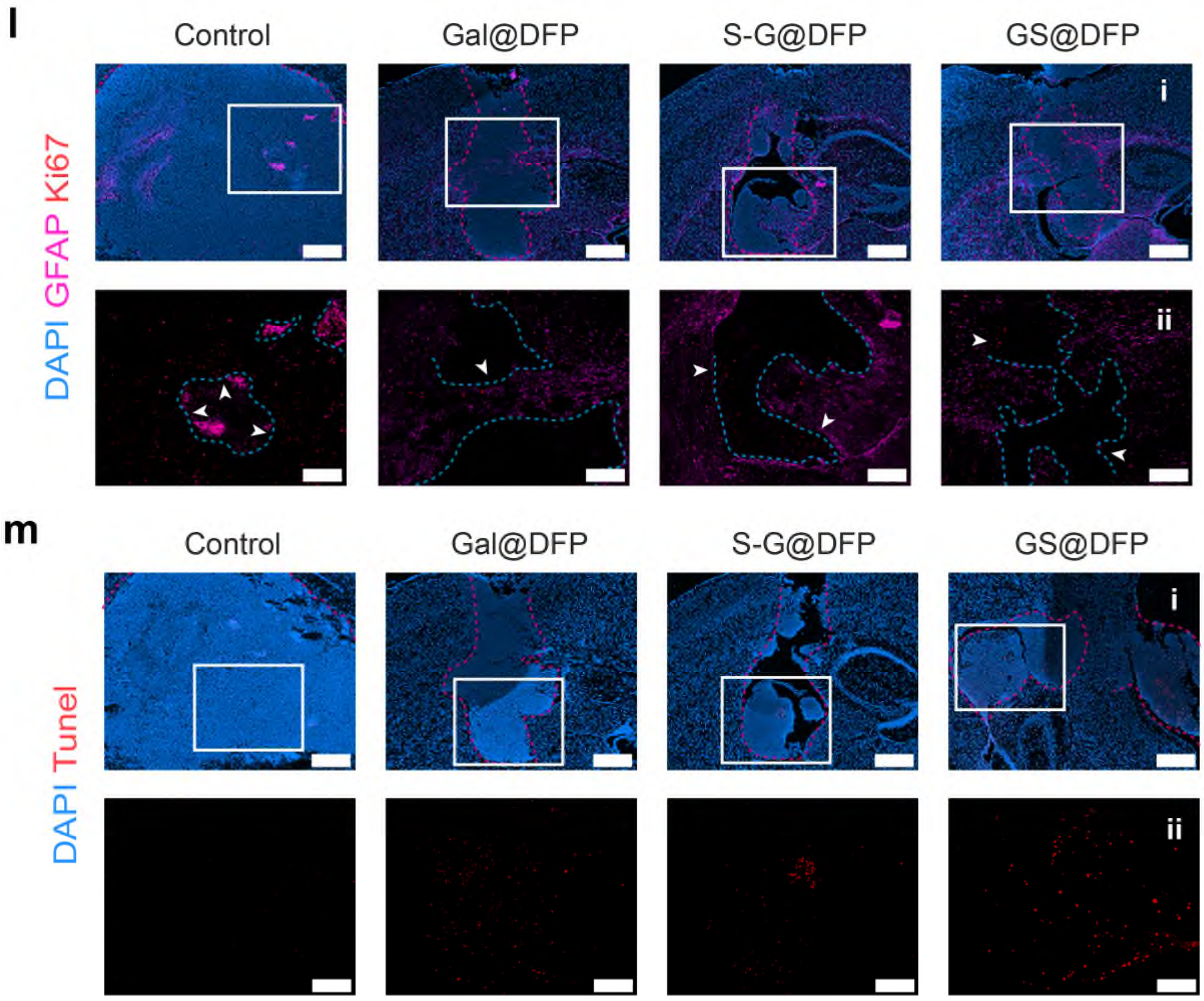

Fig. 7l m. l Fluorescence staining analysis of astrocyte and Ki67 protein expression; m Fluorescence staining analysis of apoptosis-related signal expression. Tumor regions are indicated by red dashed lines in panel i (scale bar = 200 μm) and by blue dashed lines in panel ii (scale bar = 100 μm).

4) Notably, in the mitochondrial rescue experiment, supplementation with astrocyte-derived mitochondria partially restored the cell cycle suppression induced by Galunisertib, demonstrating that mitochondrial supply functionally contributes to the observed phenotype.

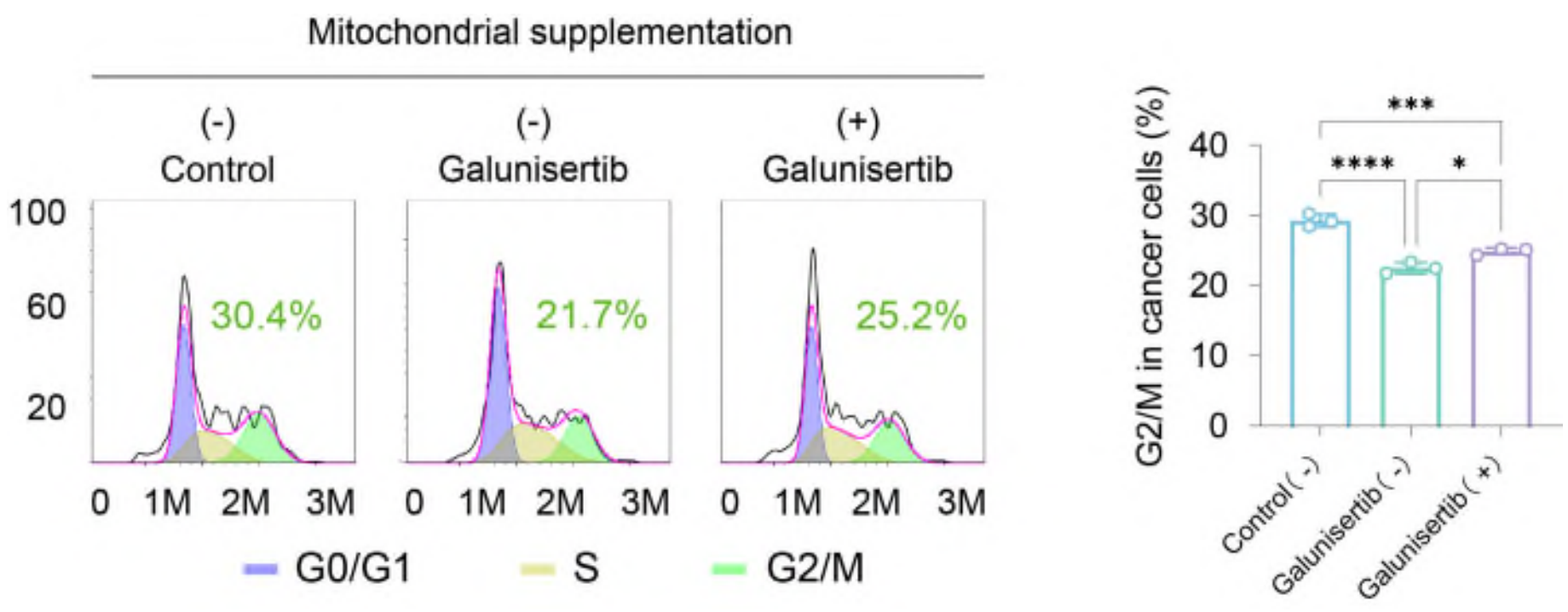

Figure R15. Representative cell cycle distribution of GL261 cells under three different treatments and the proportion of cells in G2/M phase (n = 3 independent samples). *p < 0.05, ***p < 0.001, ****p < 0.0001.

5) To further dissect the contribution of the donor side, we treated astrocytes with CCCP to impair mitochondrial function prior to co-culture. This intervention reduced mitochondrial transfer from astrocytes to tumor cells and led to partial attenuation of tumor cell-associated phenotypes. However, this effect was incomplete, indicating that reducing mitochondrial quality alone is insufficient to fully block the transfer process or its functional consequences.

Importantly, this finding highlights the presence of compensatory mechanisms within the astrocyte–tumor system. While CCCP disrupts mitochondrial function at the donor level, it does not prevent transfer through structural connections nor inhibit mitochondrial utilization in recipient tumor cells.

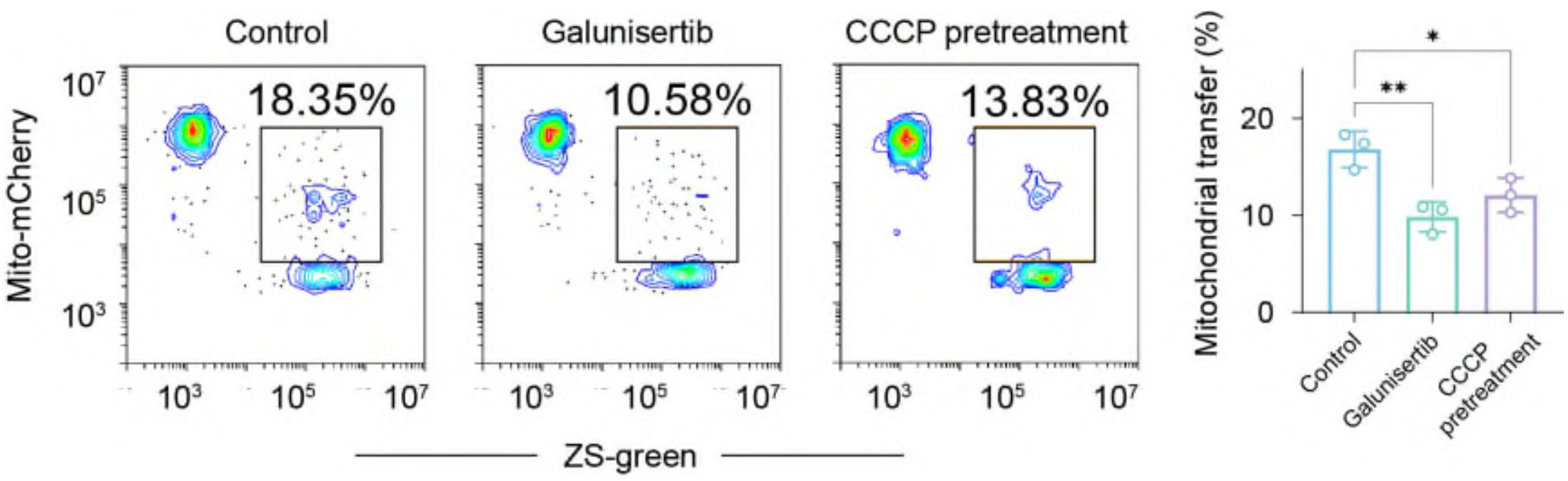

Figure R16. Proportion and quantitative analysis of mitochondria transferred from astrocytes to GL261 cells under different treatment conditions (n = 3 independent samples). *p < 0.05, **p < 0.01.

In contrast, interventions targeting tumor cells affect mitochondrial utilization or transfer pathways from the recipient side. Therefore, the combined strategy, which simultaneously disrupts mitochondrial function, transfer, and utilization, produces a more pronounced effect than any single intervention.

Together, these findings indicate that single interventions targeting either mitochondrial function or mitochondrial transfer alone are insufficient due to compensatory mechanisms within the tumor microenvironment. In contrast, these results collectively demonstrate that the enhanced effect arises from coordinated disruption of mitochondrial supply, transfer, and utilization, rather than simple additive cytotoxicity.

We would like to emphasize that we do not claim strict pharmacological synergy in a mathematical sense. Rather, our data support a coordinated and functionally synergistic effect arising from targeting a biologically coupled system, rather than from additive cytotoxicity or off-target effects.

(2) Regarding potential off-target effects

We appreciate the reviewer's concern regarding the possibility that the observed effects may arise from nonspecific or off-target toxicity. We have carefully evaluated this possibility and provide the following evidence supporting a mechanism-driven effect.

1) the molecular and phenotypic changes observed in our study are consistent with the known mechanisms of the respective interventions. Specifically, modulation of the TGF-β/SMAD/TSP-1 axis and inhibition of mitochondrial function were observed following treatment, supporting pathway-specific activity rather than indiscriminate cellular damage.

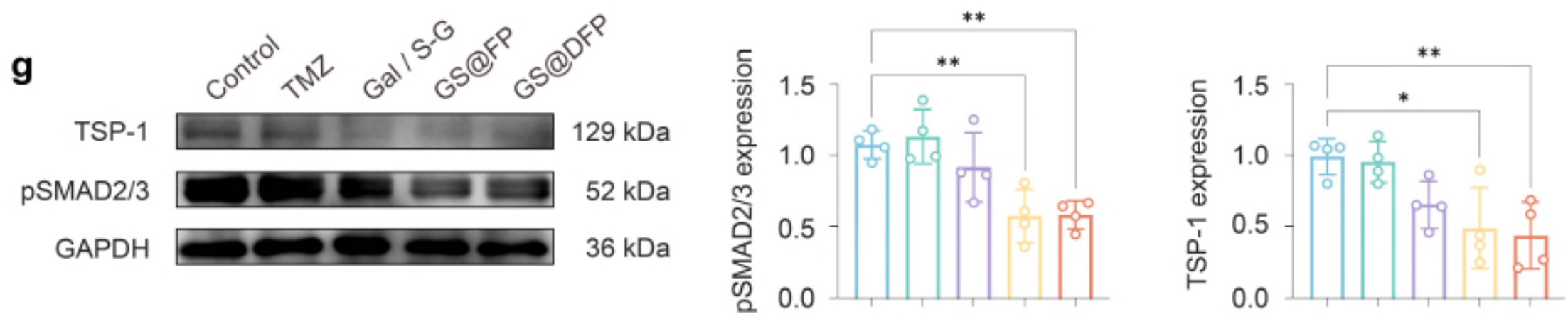

Fig. 8g. Western blot analysis and quantitative results of pSMAD2/3 and TSP-1 expression in tumor tissues from each treatment group (n = 3). *$p < 0.05$, **$p < 0.01$, ***$p < 0.001$.

2) Histological and immunofluorescence analyses revealed that the observed changes were predominantly localized within tumor regions (**Fig. 7g h l m**), while adjacent normal brain tissues did not exhibit obvious structural damage or abnormal alterations. This spatially restricted effect argues against nonspecific systemic or neural toxicity.

In addition, the *in vitro* findings are consistent with our in vivo maximum tolerated dose (MTD) evaluation. Histological (H&E) analysis showed no evident tissue damage in brain tissues of mice treated with 2× or even 4× doses of nanoparticles, indicating favorable safety in non-tumor brain regions. This further addresses the reviewer's concern regarding potential off-target oxidative stress.

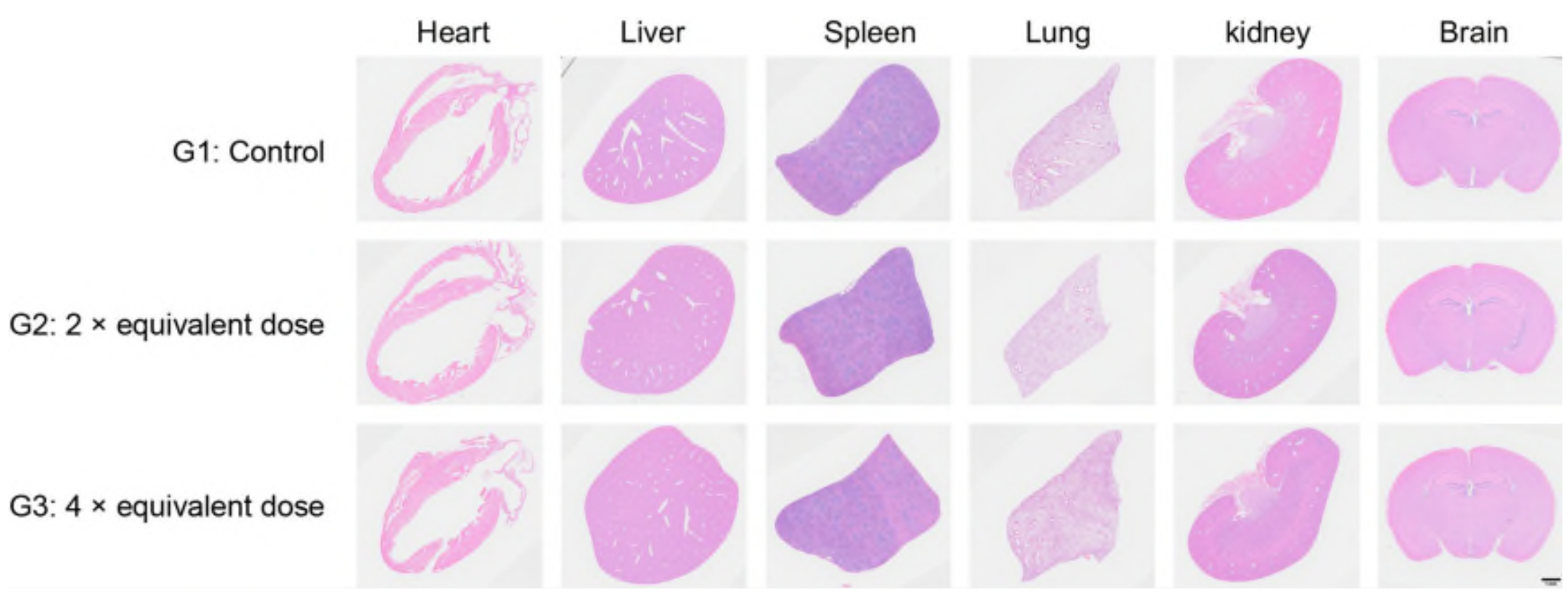

Figure R4. H&E-stained sections of major organs from mice in different treatment groups. Scale bar = 1 mm.

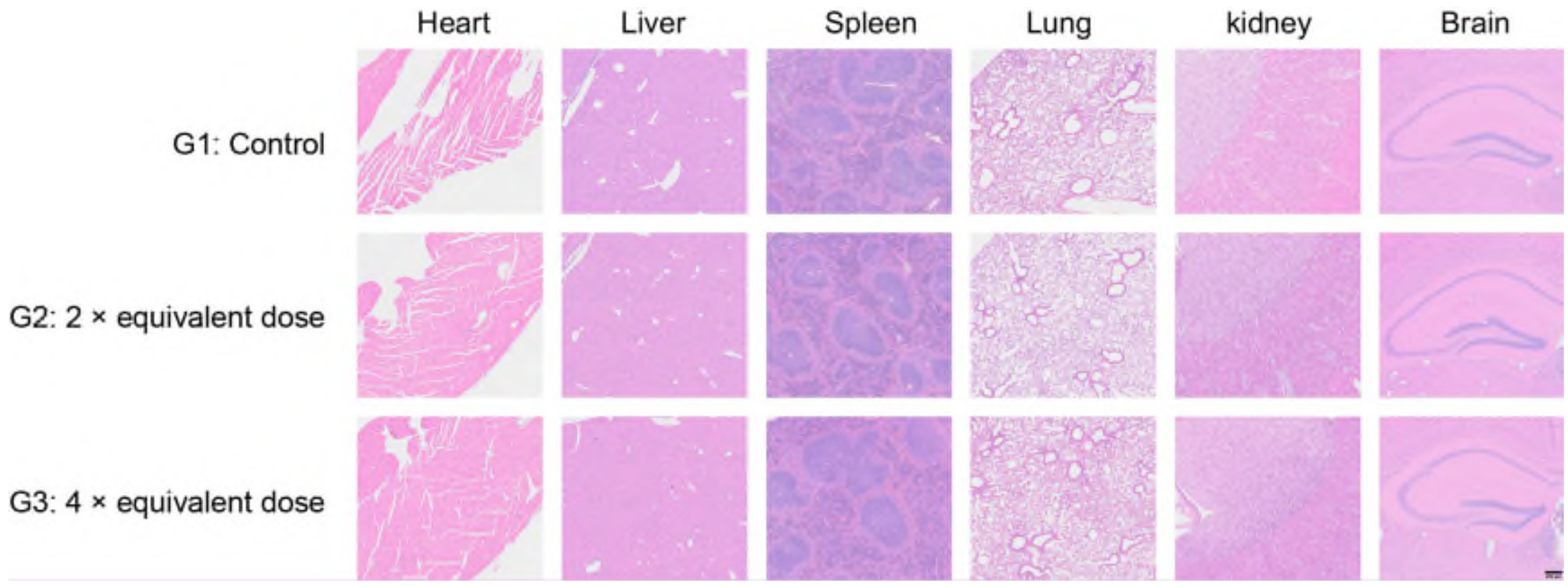

Figure R5. Magnified views of H&E-stained sections of major organs from mice in different treatment groups. Scale bar = 200 μm.

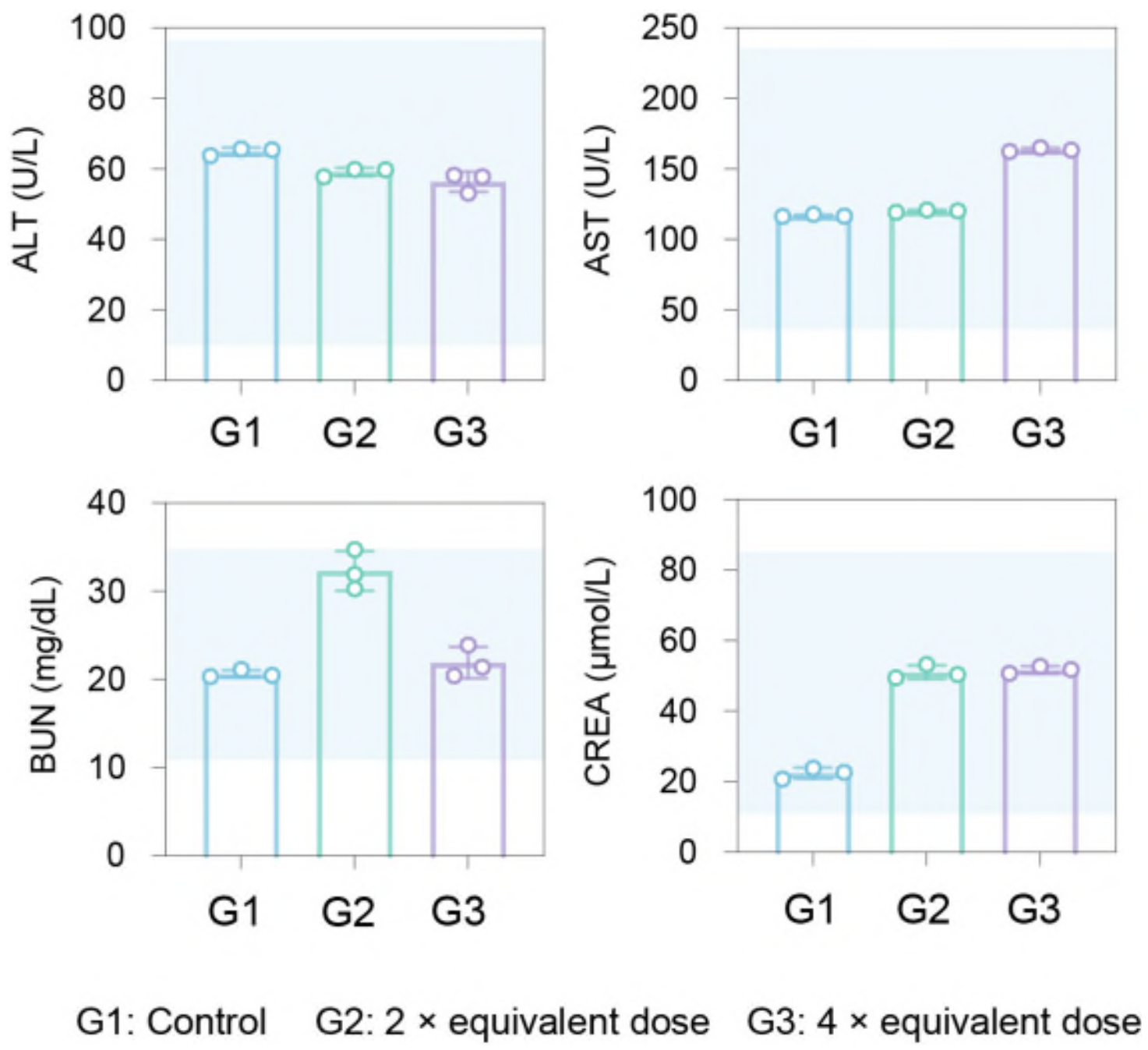

Figure R6. Semi-quantitative analysis of ALT, AST, BUN, and CREA levels in the serum of mice from different treatment groups. Blue shading indicates the normal range for each parameter. ALT, alanine aminotransferase; AST, aspartate aminotransferase; CREA, creatinine; BUN, blood urea nitrogen (n=3 independent samples).

3) Our safety and ROS analyses showed that blank nanoparticles did not induce significant oxidative stress in normal neural or endothelial cells, while tumor cells exhibited higher sensitivity. This differential response argues against generalized

toxicity and supports a tumor context–dependent effect.

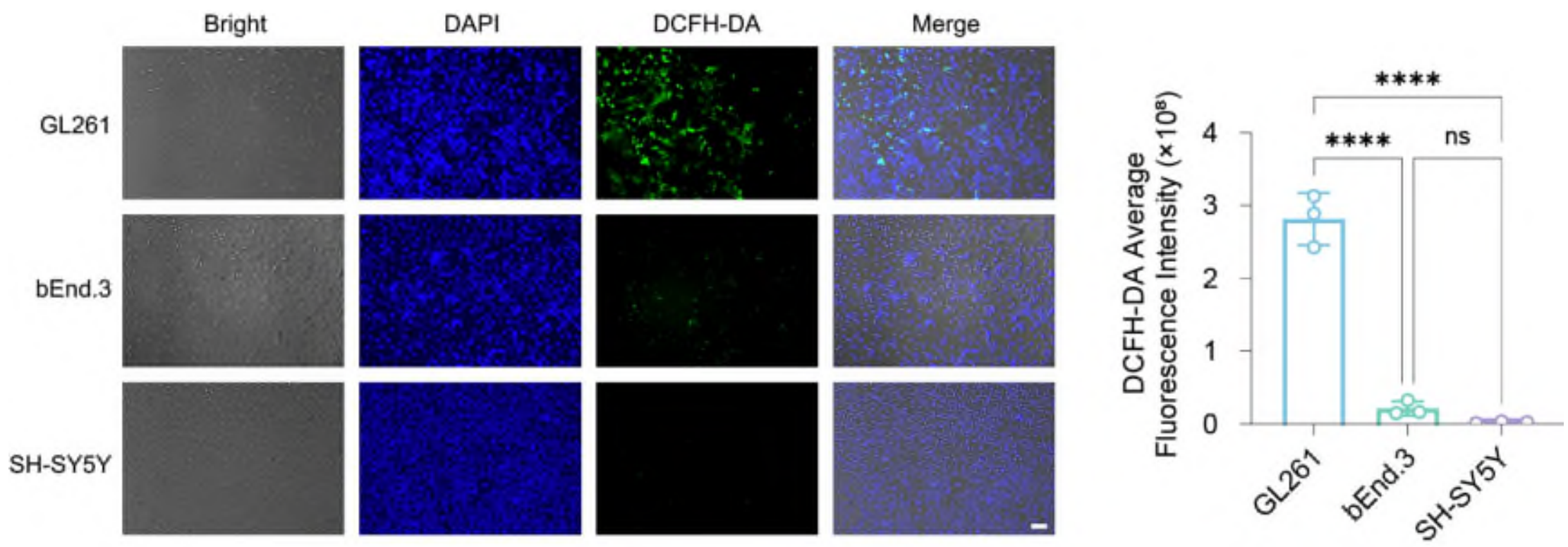

Figure R10. After treatment of different cell types with blank nanoparticles for 24 h, cells were stained with the ROS probe DCFH-DA and observed using an inverted fluorescence microscope, along with quantitative analysis (n = 3 independent fields of view), scale bar = 100 μm.

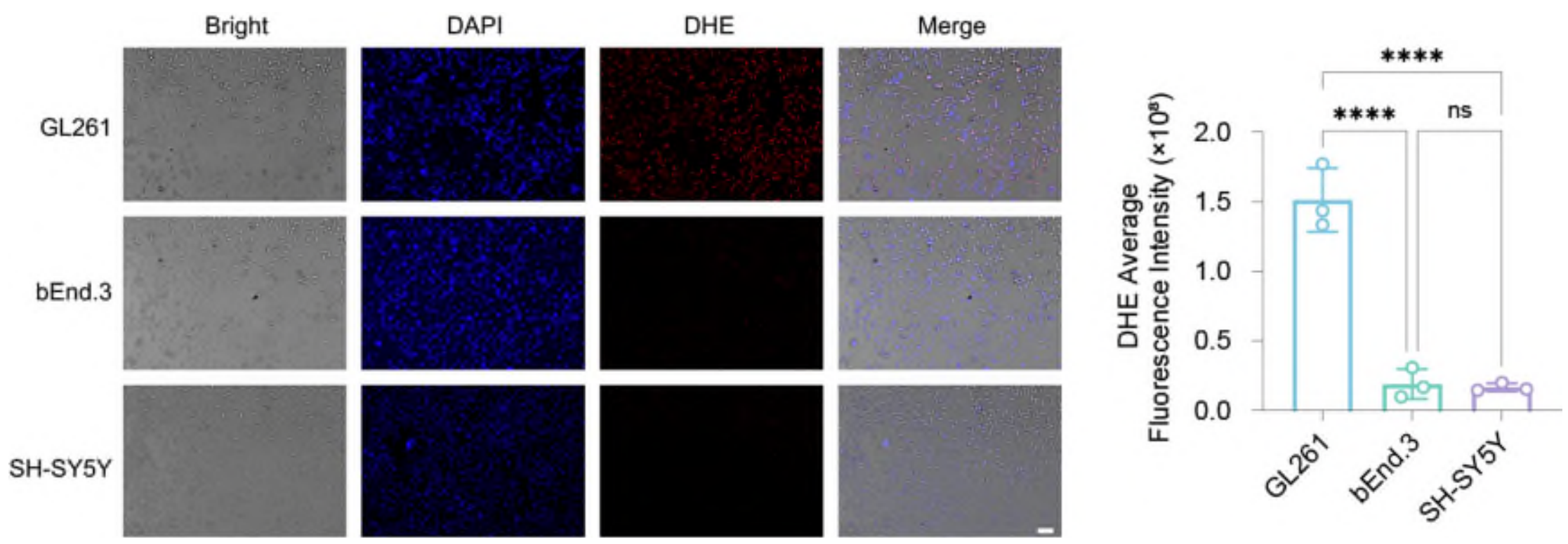

Figure R11. After treatment of different cell types with blank nanoparticles for 24 h, cells were stained with the ROS probe DHE and observed using an inverted fluorescence microscope, along with quantitative analysis (n = 3 independent fields of view), scale bar = 100 μm.

Importantly, under combination treatment, the enhanced therapeutic outcome is characterized by coordinated disruption of mitochondrial supply, transfer, and utilization, rather than a simple increase in cytotoxicity. No evidence of abnormal activation of unrelated pathways was observed. These findings support that the observed effects are predominantly on-target and mechanism-driven, with no

indication that nonspecific or off-target toxicity is the primary contributor.

In summary

Taken together, our combinational strategy is rationally designed based on a coordinated biological framework rather than arbitrary integration, and elicits tumor-specific, mechanism-driven effects without evidence of off-target toxicity.

2. The conclusions are based on only a single model, which is not convincing. GBM is well known for its high degree of heterogeneity, and at least one additional model is required, at least in key data.

Response: We thank the reviewer for highlighting the importance of model diversity. We fully agree that glioblastoma (GBM) is highly heterogeneous, and that a single model is insufficient to comprehensively represent its biological characteristics.

(1) We would like to clarify that the conclusions of this study are not based on a single model. In Figure 2, we have systematically demonstrated the generality of mitochondrial transfer from multiple perspectives. In the G422 and GL261 murine glioma models, mitochondrial transfer from astrocytes to tumor cells was observed through both co-culture systems and in vivo experiments. In addition, similar features of mitochondrial transfer were also detected in human GBM tissue samples, suggesting that this phenomenon is not restricted to a specific mouse model but is also present in clinically relevant systems.

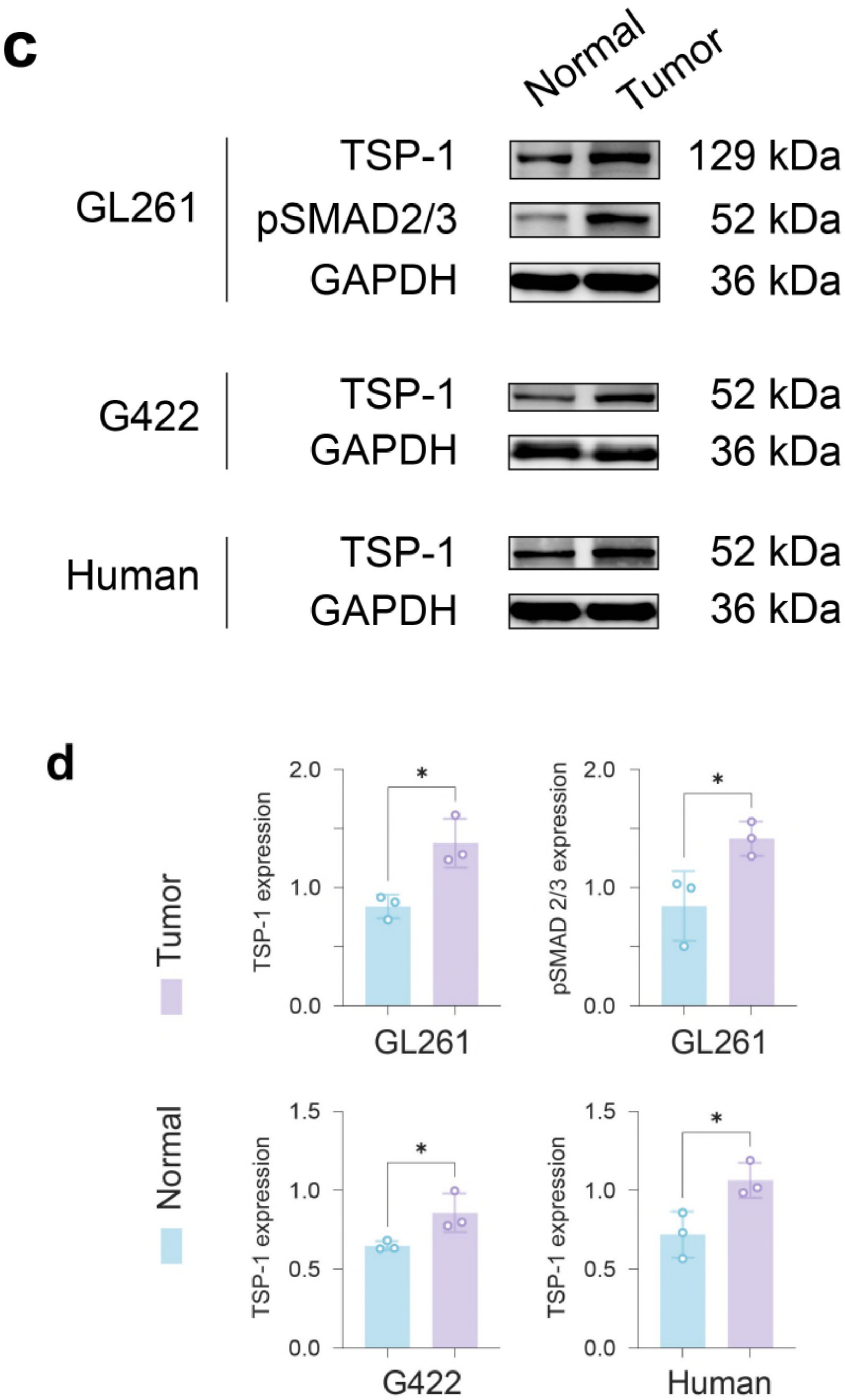

Fig. 2c d. Western blot analysis of pSMAD2/3 and TSP-1 levels in GL261/G422 tumor-bearing mouse models, clinical GBM patients (c) and the quantitative results (d).

g

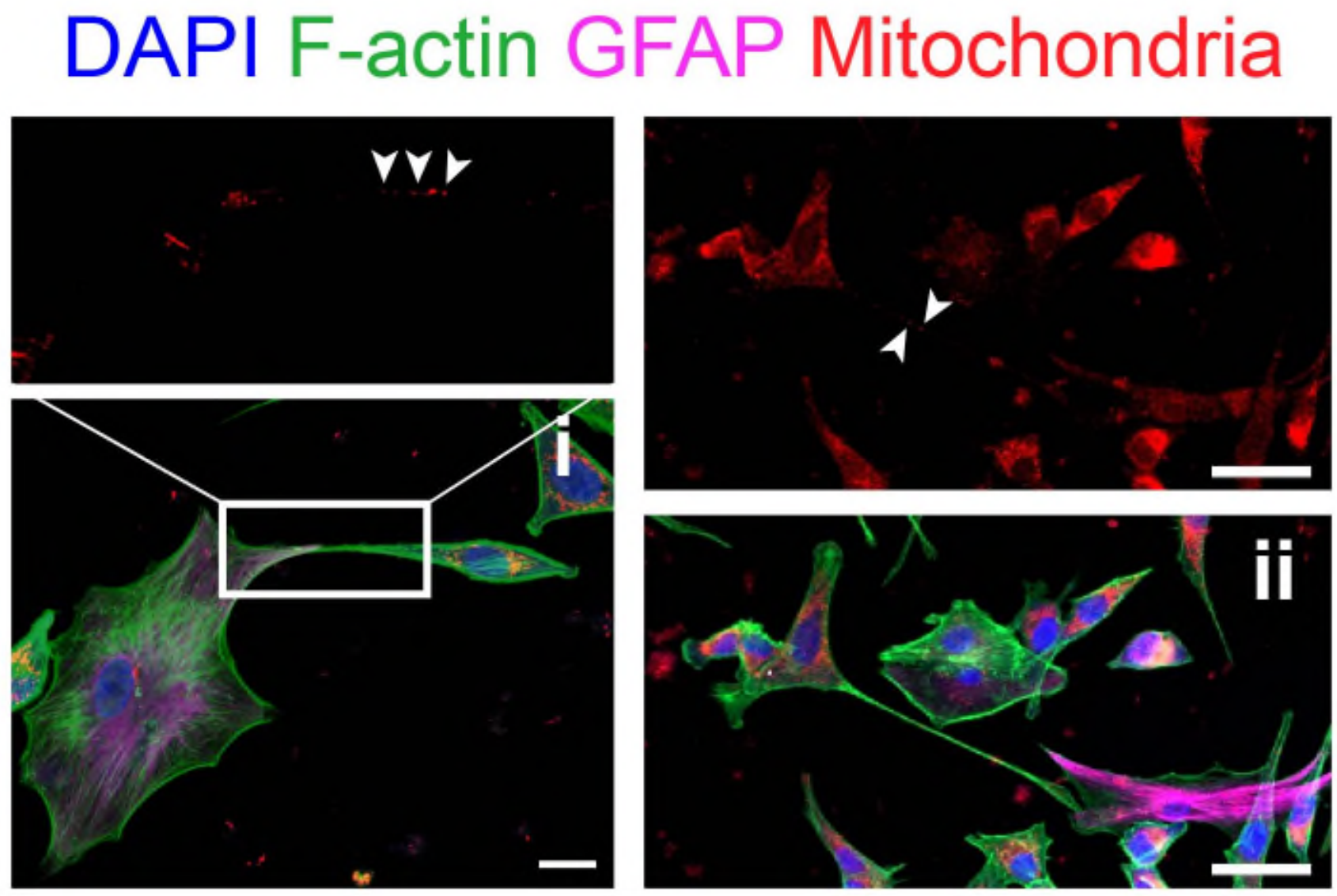

Fig.2g. Punctate mitochondrial signals were observed within tumor MTs. Scale bar = 20 μm.

i

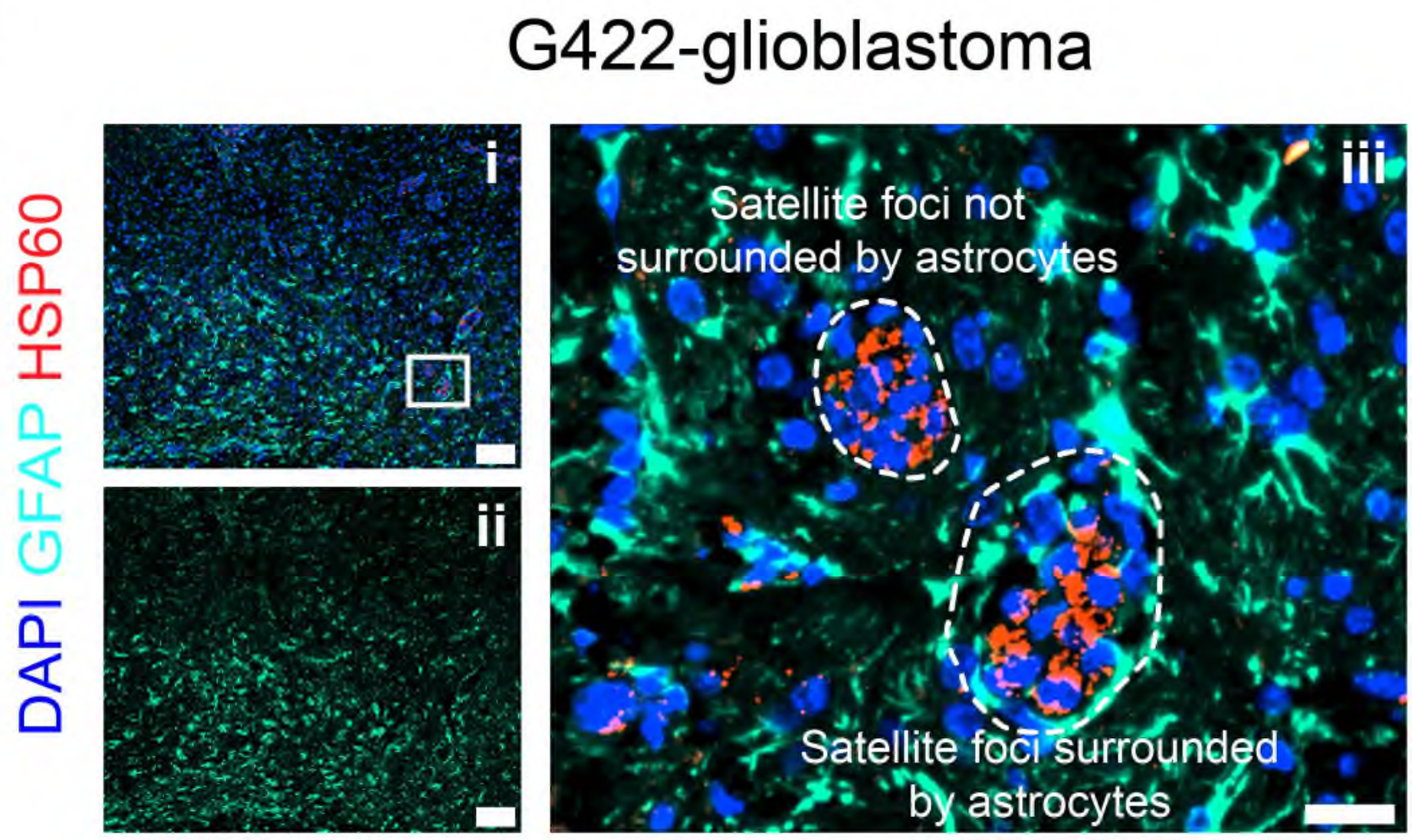

Fig.2i. In G422 tumor-bearing mouse model sections (i), reactive astrocytes accumulate (ii), tumor satellite foci surrounded by reactive astrocytes contain more mitochondria (iii). Scale bar = 100 μm (i), (ii), 20 μm (iii).

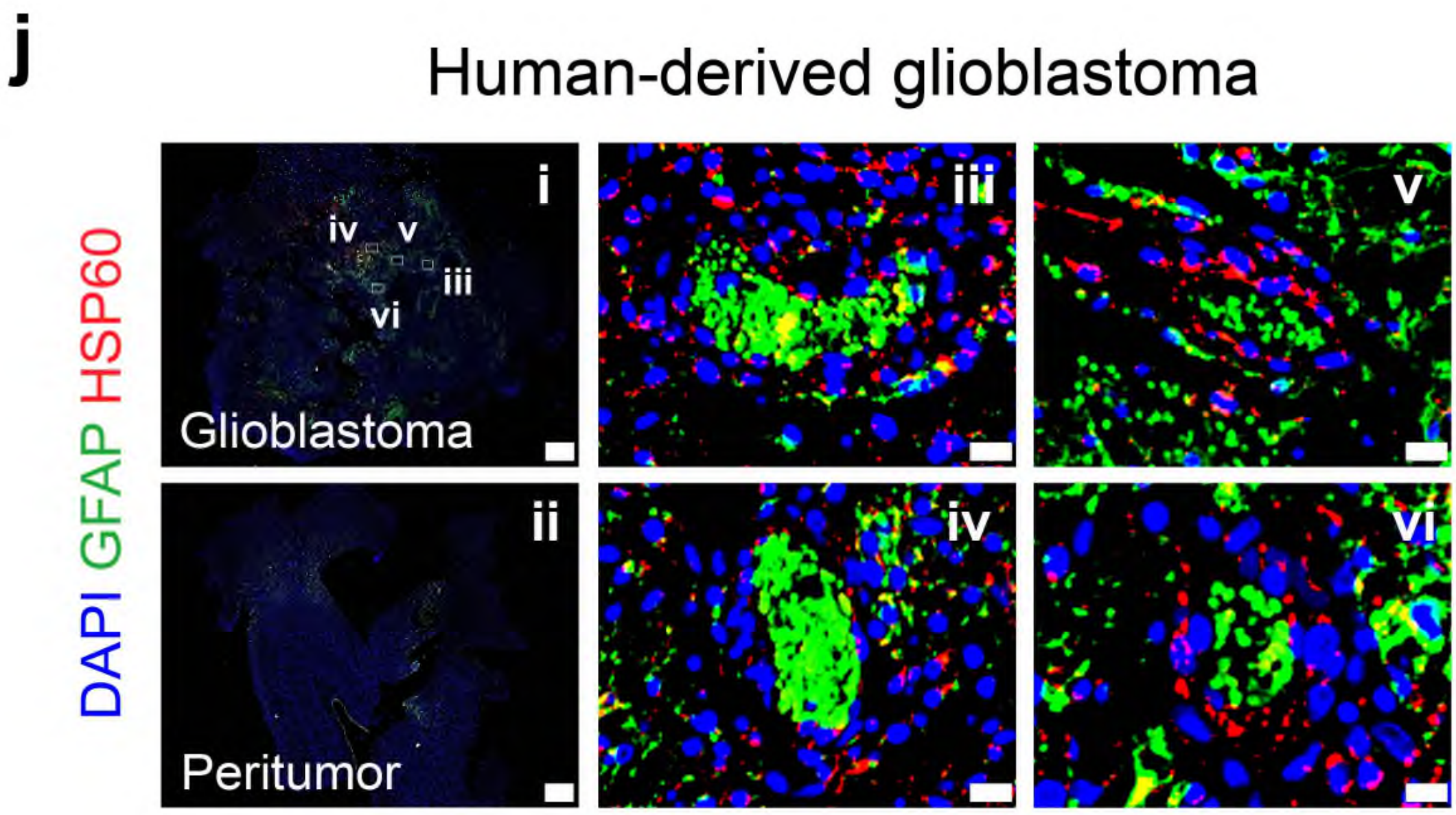

Fig.2j. In clinical samples of GBM and adjacent normal tissue sections (i), (ii), and astrocytes are engulfed (iii), (iv), more mitochondria signals are present in the surrounding tumor tissues (v), (vi). Scale bar = 1mm (i), (ii), 20 μm (iii)-(vi).

Therefore, this study integrates both animal models and human tissue samples, covering different levels of investigation and partially addressing the biological heterogeneity of GBM.

(2) We agree with the reviewer that further expansion of models would strengthen the generalizability of the conclusions. Accordingly, we have also performed mitochondrial transfer inhibition experiments in the G422 model. Consistent with the approach used in GL261 cells, G422 cells were pretreated with Galunisertib, while astrocytes were pre-labeled with the mitochondrial probe Mito-Tracker Red. After extensive washing, G422 cells were co-incubated with astrocytes for 24 hours, and mitochondrial transfer was quantitatively analyzed by flow cytometry. The results showed that Galunisertib similarly reduced mitochondrial transfer from astrocytes to G422 cells. Together with our previous observation that Galunisertib reduces tumor microtubes in G422 cells, these findings support the generality of Galunisertib-mediated inhibition of mitochondrial transfer via suppression of tumor

microtubes.

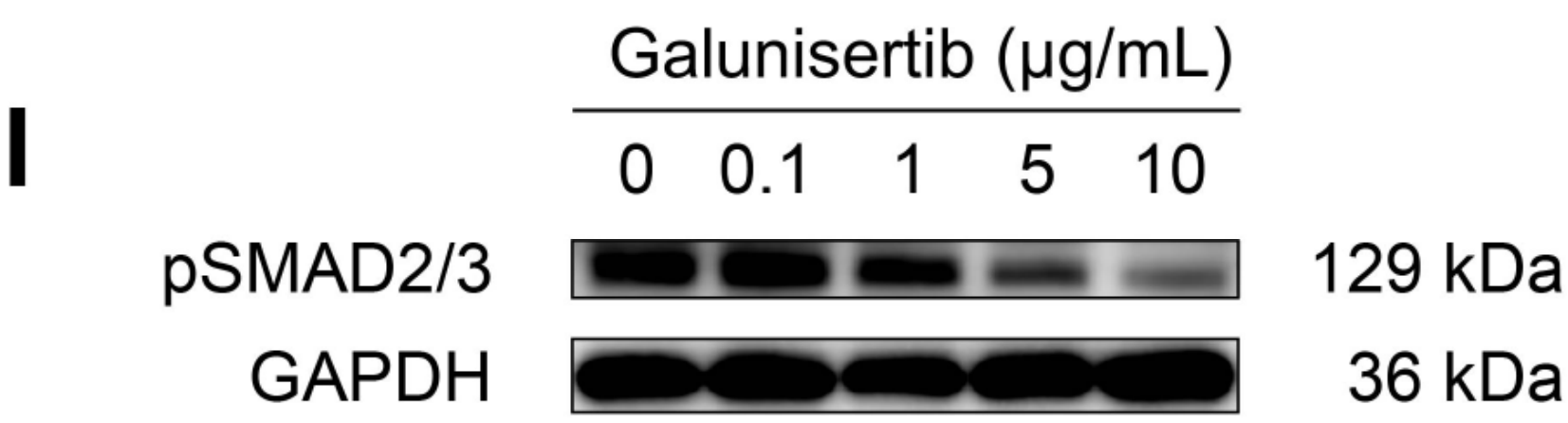

Fig. 2l. Western blot showing TSP-1 and pSMAD2/3 expression in G422 cells treated with Galunisertib.

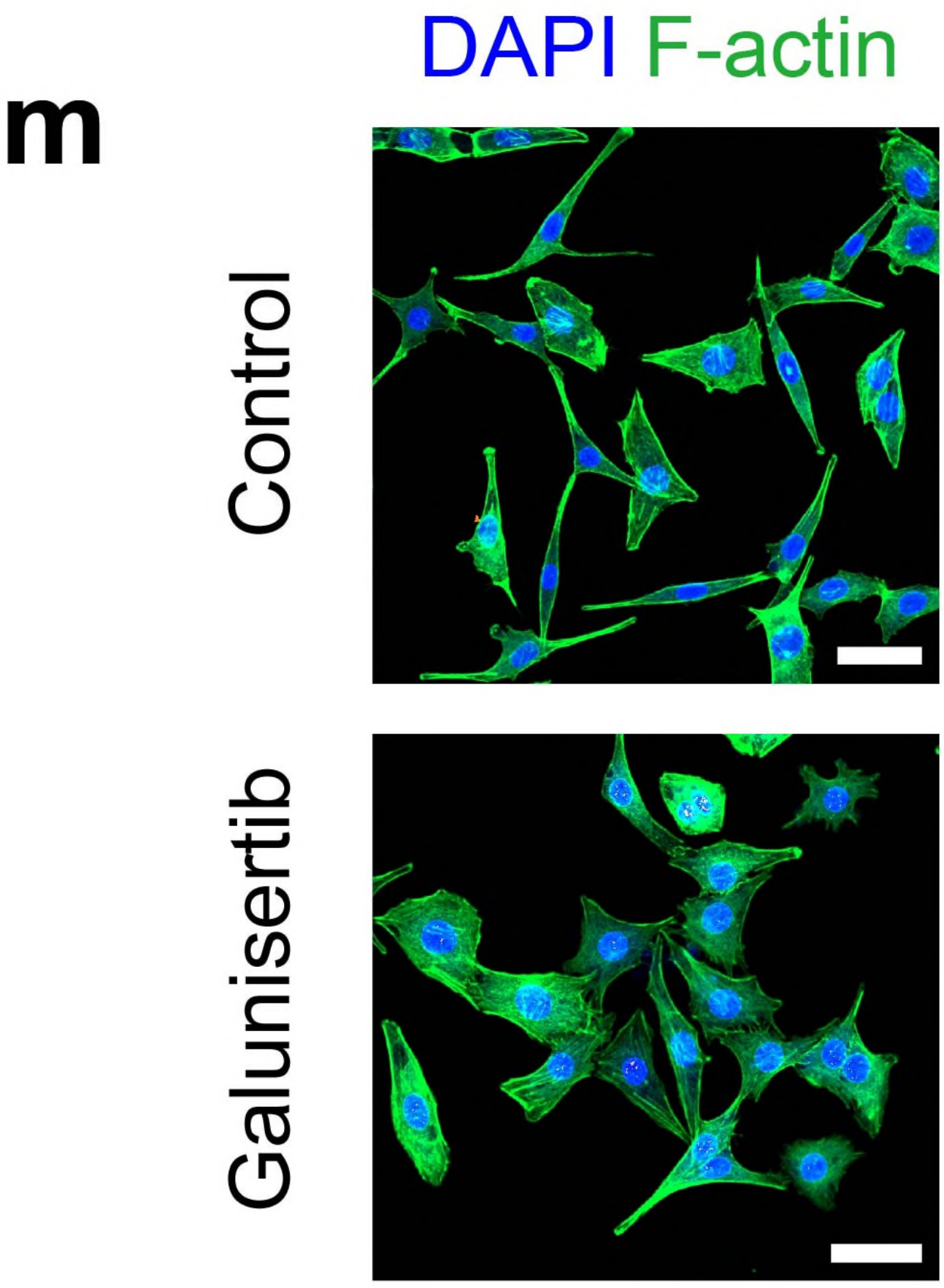

Fig. 2m. hological observation of tumor MTs in G422 cells treated with Galunisertib. Scar bar = 20 μm.

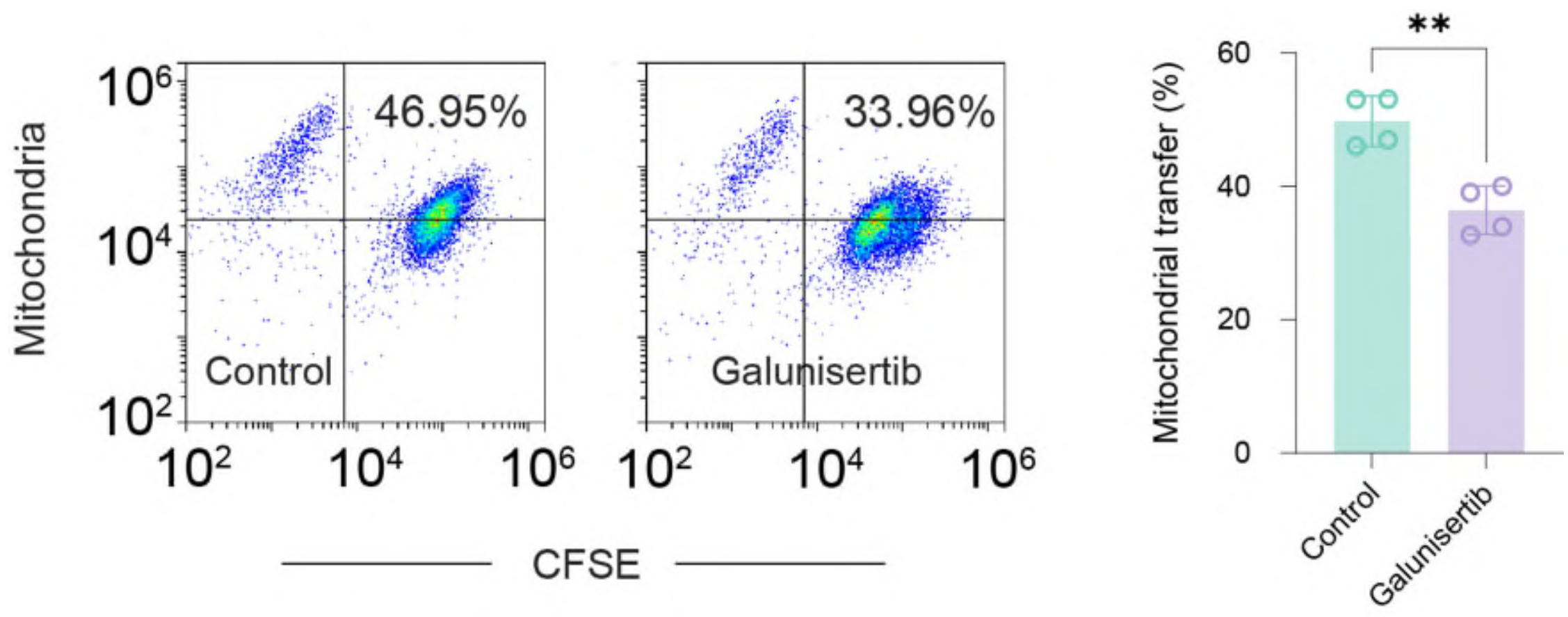

Figure R17. Proportion and quantitative analysis of mitochondria transferred from astrocytes to G422 cells under different treatment conditions (n = 3 independent samples). **p < 0.01.

In summary, our current data support the existence and functional relevance of mitochondrial transfer in GBM at both the murine model and human tissue levels, and further demonstrate that Galunisertib inhibits mitochondrial transfer between astrocytes and G422 tumor cells. We also suggest that future studies should further validate this mechanism using patient-derived models to enhance its translational relevance.

3. Typically, figures are presented in numerical order. Starting the Results section with Figure 2 is not very common.

Response: We thank the reviewer for this helpful suggestion regarding figure order. We agree that figures are typically presented in numerical order. However, in our study, Figure 1 is designed as a schematic/overview figure to summarize the conceptual framework and does not correspond directly to a specific set of experimental results. Therefore, we chose to begin the Results section with Figure 2, which presents the first set of primary experimental data, in order to maintain a clear logical flow from experimental observations to mechanistic insights.

We believe that this structure improves readability and better guides the reader through the study design and key findings.

4. Immunoblot images throughout the manuscript should include sufficient surrounding background and should not be cropped too narrowly.

<u>Response:</u> We thank the reviewer for the valuable comment regarding the presentation of immunoblot images. We fully agree that providing sufficient background is essential for data transparency and reproducibility.

We would like to clarify that the cropping in the main figures was performed solely for clarity and to focus on the target proteins, and not to selectively present or manipulate data. All uncropped Western blot images, with clearly labeled molecular weight markers, have been provided in the Supplementary Information. Considering these complete data and the optimized presentation in the main figures, we believe that the transparency and rigor of the Western blot results are fully ensured.

5. In line 51, comparing the IC_{50} values of S-Gboxin and TMZ in a mouse cell line is confusing and does not provide meaningful information.

<u>Response:</u> Thank the reviewer for pointing out this issue. We agree that directly comparing the IC_{50} values of S-Gboxin and TMZ in mouse cell lines may be misleading, and given the distinct mechanisms of action of the two drugs, such a comparison lacks sufficient reference value.

In the revised manuscript (**lines 50–53**), we have removed the unsupported statement and the IC_{50} comparison, and replaced it with a more accurate and evidence-based description:

~~S-Gboxin exhibits approximately 200-fold lower IC50 than TMZ in GL261 cells, but fails to block astrocyte-derived mitochondrial compensation resulting in tumor recurrence and increased proliferative capacity in orthotopic models.~~

S-Gboxin is a potent inhibitor of oxidative phosphorylation in tumor cells. However, tumor-microenvironment interactions may influence its therapeutic efficacy, suggesting the need for combinational strategies.

In the revised manuscript, the inappropriate direct comparison has been removed, and the section has been revised to improve the rigor and interpretability of the data presentation.

6. In Figures 5i and 5j, the labels are incorrect. Several panels in this part are mislabeled and confusing. In Figures 5k–5m and the corresponding Results section, the inclusion of TMZ appears irrelevant to this study. The rationale for introducing TMZ at this point is confusing. In addition, in line 265, “GS@Defficiently” appears to be a typographical error. This section contains too many mistakes and is extremely confusing. Additionally, in Figure 2m, for the statistical analysis of Ki-67, it is unclear whether the y-axis represents percentages or absolute numbers.

Response: We thank the reviewer for the detailed comments on Figure 5 and the related Results section. In response to the concerns, we have made the following adjustments and clarifications in the revised manuscript:

1. Regarding Figure 5i, 5j and labeling issues

We carefully checked the panels and confirmed that there were labeling errors and spelling mistakes in the original manuscript (e.g., “GS@Defficiently”).

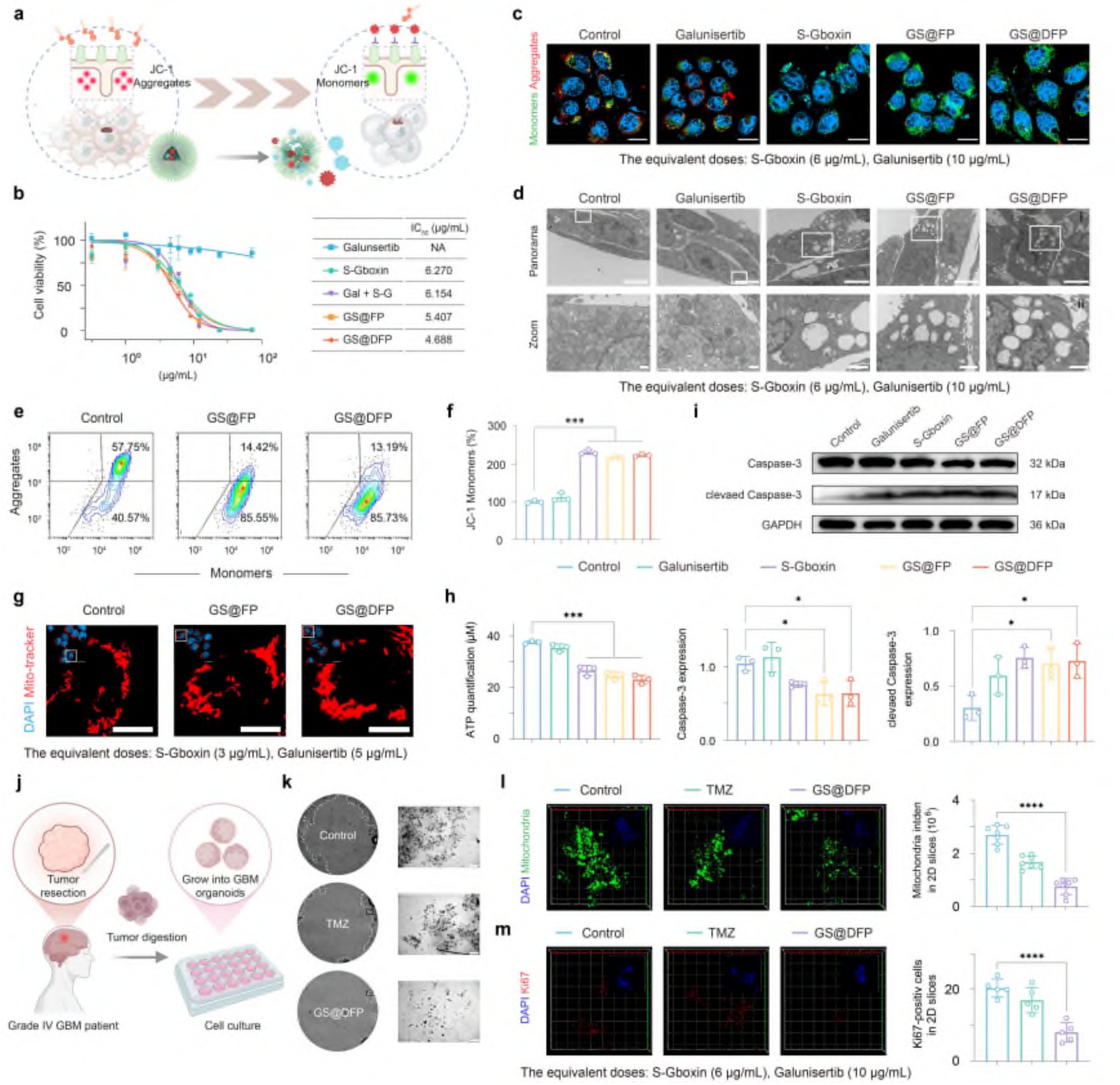

Fig. 5 | GS@DFP disrupts mitochondria and inhibits cell proliferation. **a** Schematic illustration of mitochondrial disruption and JC-1–indicated membrane potential changes. **b** Fitted cell viability curves and half-maximal inhibitory concentrations (IC_{50}) of GL261 cells after treatment with Galunisertib, S-Gboxin, Galunisertib + S-Gboxin, GS@FP, or GS@DFP for 24 h (n=4 independent samples). **c** Representative CLSM images of JC-1–stained GL261 cells after different treatments for 6 h. Scar bar = 20 μm. **d** TEM images showing mitochondrial morphological changes in cancer cells after different treatments for 12 h. Scar bar = 5 μm in (i). Scar bar = 1 μm in (ii). **e f** Representative flow cytometry plots of JC-1 staining (**e**) and quantitative analysis of JC-1 monomers (**f**) in GL261 cells (n=3 independent samples). **g** CLSM images of mitochondrial morphology in GL261 cells after treatments. Scar bar = 10 μm. **h** Intracellular ATP levels of GL261 cells measured after different

treatments for 12 h (n=3 independent samples). **i** Western blot analysis and quantitative results of apoptosis-related proteins Caspase-3 and cleaved Caspase-3 following mitochondrial disruption (n=3 independent samples). **j** Establishment of patient-derived tumor organoid models from primary WHO grade IV GBM tissues. **k** Panoramic and representative local images showing the effects of TMZ or GS@DFP treatment for 48 h on GBM organoid formation. Scar bar = 100 μm. **l** Representative 3D mitochondrial staining images and fluorescence quantification of GBM organoids after different treatments (n=7 independent slices). **m** Representative 3D Ki67 fluorescence staining images and quantification of $Ki67^{+}$ cells in GBM organoids after different treatments (n=5 independent slices). $*p < 0.05$, $***p < 0.001$, $****p < 0.0001$.

The panels in Figures 5i and 5j are not labeled incorrectly in terms of data errors. In terms of logical flow, we systematically elaborated on the inhibition of tumor cell ATP synthesis by GS@DFP, the destruction of mitochondria, the initiation of necroptosis, and ultimately the activation of the apoptosis program. At the same time, considering the aesthetic layout, we deliberately set 5i for WB to investigate the apoptosis of tumor cells, and subsequently set the investigation of the effect on human-derived organoids in 5j

To optimize the logical flow and aid reader comprehension, we have revised the label descriptions in Figure 5 to clearly reflect the sequence and context of the experiments, ensuring consistency between the figures and the text.

2. Regarding the role of TMZ in Figures 5k–5m

TMZ was included as a clinical benchmark to provide a reference for comparing the efficacy of our proposed GS@DFP strategy with an existing first-line therapy. TMZ is not a core part of the mechanistic study nor directly involved in the drug design of this work; it serves as a clinically relevant comparator to help readers intuitively assess the relative advantage of GS@DFP.

3. Regarding Ki-67 quantification and Figure 5m

The y-axis units in Figure 2m have been explicitly labeled as absolute values,ensuring unambiguous interpretation of the data.

In summary, the revised version addresses the reviewer's concerns regarding confusing figure labels, the seemingly abrupt inclusion of TMZ, and unclear statistical annotations. It also clarifies the rationale for including TMZ as a clinical reference, allowing readers to better appreciate the potential translational value of our GS@DFP strategy.

7. Throughout the manuscript, the colors used in the figures are very similar, making it difficult to distinguish different groups. The authors should also clearly indicate what each bar represents, rather than relying on a single legend or representative description across whole figure. This issue is particularly severe in Figures 6 and 7.

Response: We thank the reviewer for pointing out the issues regarding color differentiation and figure legend labeling. In response, we have made the following adjustments and clarifications in the revised manuscript:

1. Color Optimization

Our color scheme was designed with reference to the visualization style commonly used in Nature journals, aiming to balance professionalism and aesthetic appeal while ensuring clear visual contrast among groups. This color palette, commonly seen in high-impact journals, was carefully adjusted to be both visually pleasing and scientifically readable. Beyond color, we also employed shapes, symbols, and contrast to distinguish different experimental groups, preventing potential confusion if relying solely on color. We paid special attention to the combination of different data types (bar graphs, line graphs, scatter plots) to ensure overall clarity and readability. When selecting colors, we considered visual accessibility, as well as print and electronic presentation, ensuring that group differences remain distinguishable even in grayscale. Although some colors are close, the use of legends and symbols allows each experimental group to be clearly identified.

2. Explicit Labeling of Each Bar/Group

All bar graphs, line graphs, and scatter plots now directly label specific groups within the figure, rather than relying solely on a general legend. Repeated experimental groups and control groups are marked consistently to ensure that the meaning of every element in the figure is unambiguous.

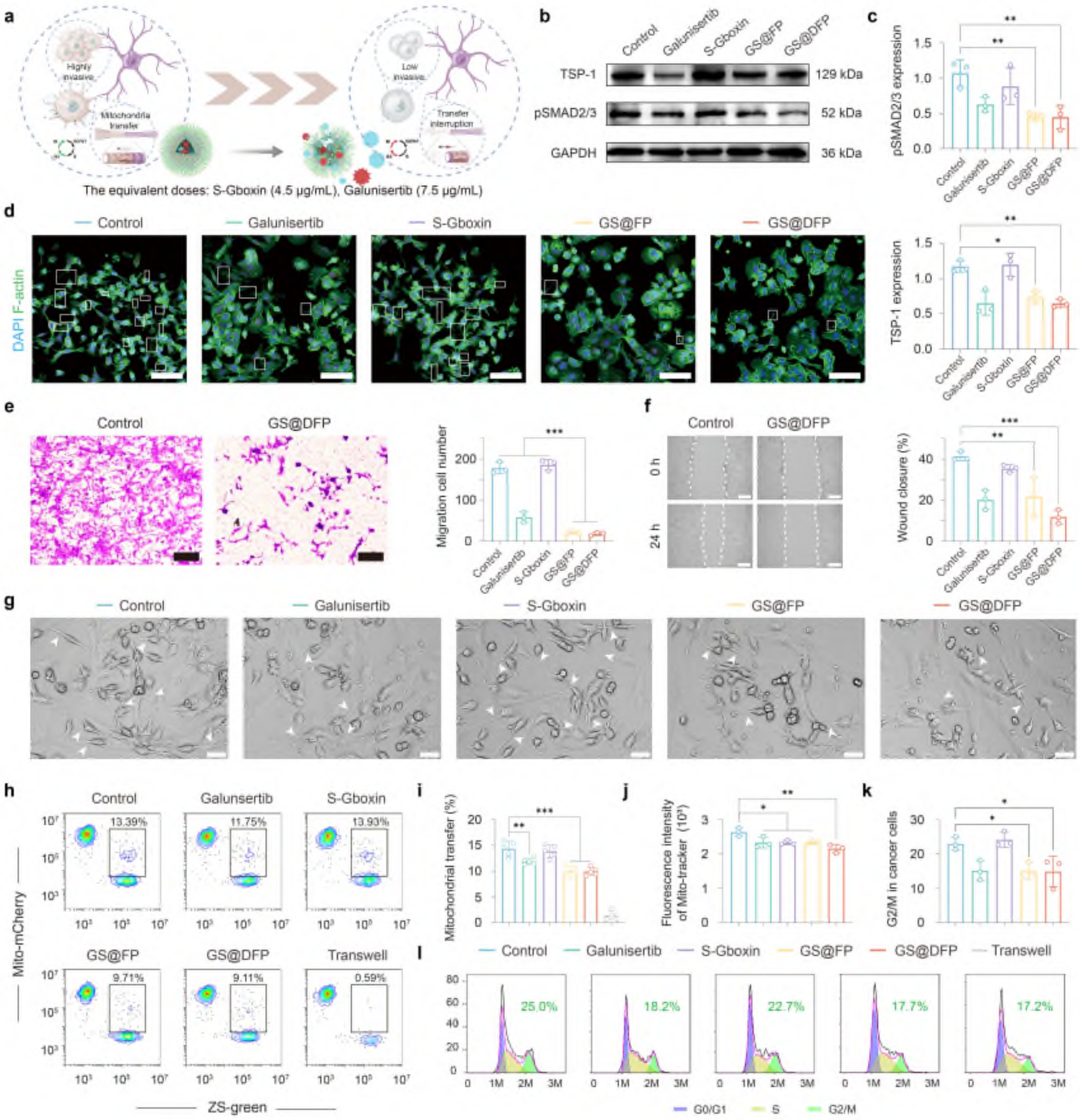

Fig. 6 | GS@DFP inhibits tumor MTs, thereby downregulating mitochondrial transfer and malignant proliferation. **a** Schematic illustration showing that GS@DFP inhibits tumor MTs, thereby weakening cancer cell invasion, reducing mitochondrial transfer, and decreasing tumorigenicity. **b c** Western blot analysis of GL261 cells treated with Galunisertib, S-Gboxin, GS@FP, or GS@DFP for 24 h, showing the expression of pSMAD2/3 and TSP-1 (**b**) and the quantitative results (**c**) (n=3 independent samples). **d** Tumor MT expression in GL261 cells after different

treatments for 24 h. Scar bar = 100 μm. **e** Representative images and quantification of invasive GL261 cells stained with crystal violet 24 h after incubation with different treatments, determined by Transwell assay (n=3 independent samples). Scar bar = 100 μm. **f** Representative images and quantification of migratory GL261 cells after incubation with different treatments for 24 h, determined by wound-healing assay (n=3 independent samples). Scar bar = 20 μm. **g** Representative images showing tumor MT-mediated connections between cancer cells and astrocytes after 24 h of pretreatment. Scar bar = 20 μm. **h i** Representative flow cytometry plots (**h**) and quantitative analysis (**i**) showing the proportion of ZS-green GL261 cells receiving astrocyte-derived mitochondria after co-culture with Mito-mCherry–transfected astrocytes (n=5 independent samples). **j** Flow cytometric quantification of mitochondrial content in ZS-green GL261 cells after co-culture with astrocytes (n=3 independent samples). **k l** Flow cytometric plots (**l**) and quantitative results (**k**) of cell cycle phases in ZS-green GL261 cells after co-culture with astrocytes (n=3 independent samples). *$p < 0.05$, **$p < 0.01$, ***$p < 0.001$.

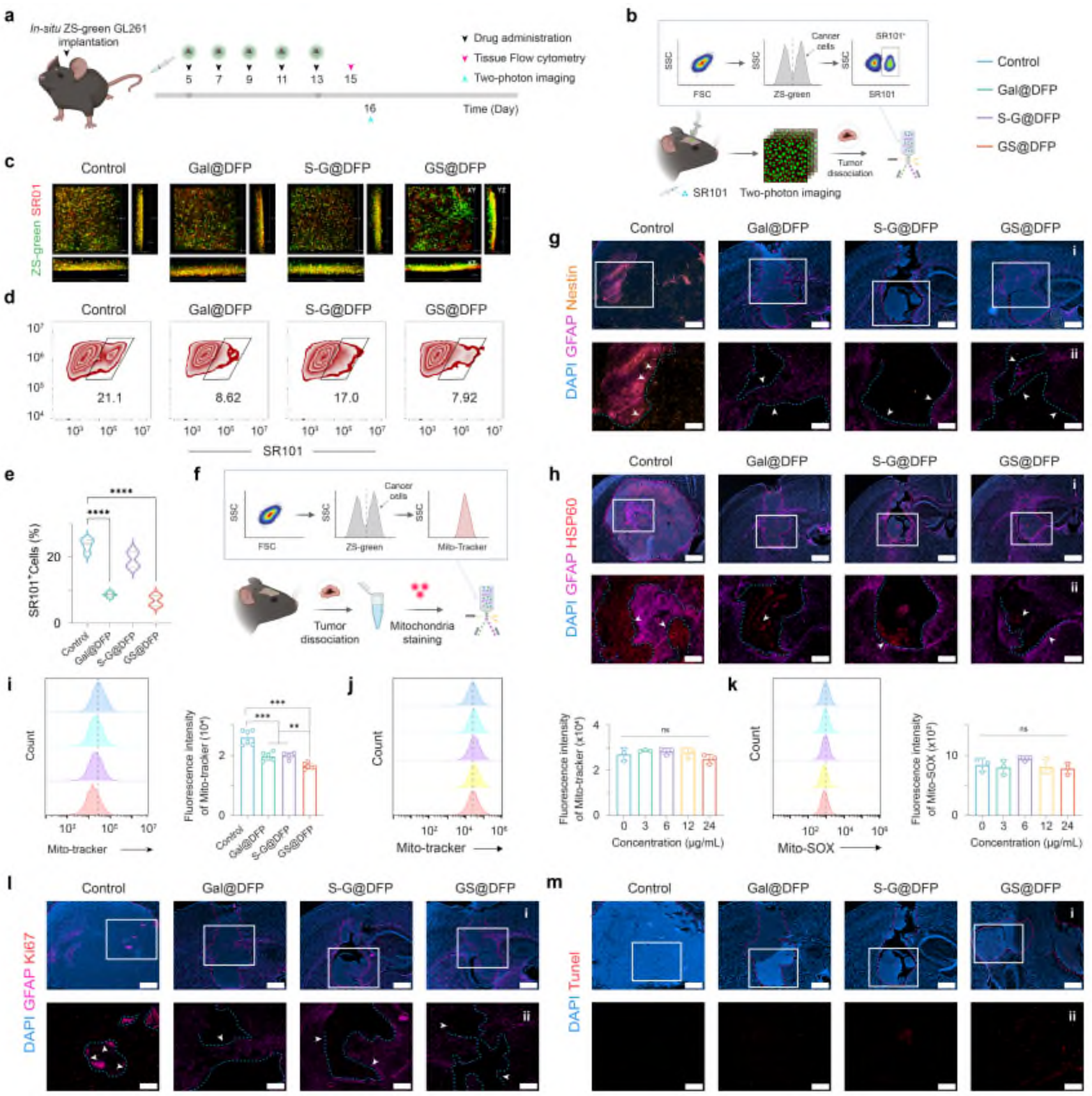

Fig. 7 | GS@DFP disrupts mitochondria in tumor tissues and downregulates mitochondrial transfer. **a** Schematic timeline of ZS-Green GL261 orthotopic tumor-bearing mouse model establishment and treatments; **b** Schematic diagram showing tumor observation by two-photon confocal microscopy after intraperitoneal injection of SR101 and the flow cytometry gating strategy; **c** *In vivo* observation of uptake of SR101 by cancer cells from astrocytes under two-photon microscopy; **d e** Representative flow cytometry plots (**d**) and quantitative analysis (**e**) of $SR101^+$ cancer cells (n=4 mice in each group); **f** Schematic diagram of mitochondrial content measurement in orthotopic tumor-bearing mice and flow cytometry gating strategy; **g** Fluorescence staining analysis of the astrocyte and MT-associated protein Nestin; **h** Fluorescence staining analysis of the astrocyte and mitochondria-associated protein Heat Shock Protein 60 (HSP60); **i** Representative FCM plots and quantitative results

of mitochondrial content in tumors from different treatment groups (n = 6 mice in each group); **j k** Representative FCM plots and quantitative results showing mitochondrial quantity (**j**) and quality (**k**) in GL261 cells treated with different concentrations of Galunisertib (n=3 independent samples); **l** Fluorescence staining analysis of astrocyte and Ki67 protein expression; **m** Fluorescence staining analysis of apoptosis-related signal expression. Tumor regions are indicated by red dashed lines in panel i (scale bar = 200 μm) and by blue dashed lines in panel ii (scale bar = 100 μm). $**p < 0.01$, $***p < 0.001$, $****p < 0.0001$.

3. Overall Improvements

We also conducted a comprehensive review and revision of all figure captions throughout the manuscript to ensure that every figure and every data group is clearly explained, avoiding potential reader confusion. The revised figures, especially Figures 6 and 7, show significant improvements in visual distinction, legend clarity, and overall readability.

Through these modifications, we believe that the revised manuscript now presents clear color differentiation, group labeling, and figure legends, allowing readers to accurately interpret the experimental results for each group.

8. In Figure 7, the effects of the authors' drug and TMZ are not directly comparable. Based on the data presented, the authors' conclusion that their drug is better than TMZ is not well supported. The dosing across different groups is not comparable. In addition, sensitivity of cancer cells to TMZ varies dramatically among different cell types Therefore the drug presented in this study is an inappropriate candidate.

Response: We thank the reviewer for the comments regarding the drug comparisons in Figure 7. Our clarifications are as follows:

1. Role of TMZ as a reference control

TMZ was included in the experiments primarily as a clinical benchmark, to provide a

point of reference for assessing relative efficacy trends and mechanistic validation of our drug in this model, rather than to enable absolute dose equivalence or a direct superiority comparison. It is well known that TMZ sensitivity can vary significantly among different cell types, including GL261, G422, and patient-derived glioma cells. However, as a first-line GBM therapy, TMZ at appropriate doses serves as a useful comparator, highlighting the mechanistic advantages of GS@DFP—specifically, its ability to interfere with astrocyte–tumor cell mitochondrial interactions and microtubule pathways—without claiming generalized superiority over TMZ.

2. Experimental dosing design

All experimental doses were chosen based on pharmacological properties, literature references (**Nat Commun 14, 4557 (2023). Nat Nanotechnol 19, 1869-1879 (2024).**), and preliminary experiments, ensuring observable efficacy in vitro and in vivo while avoiding excessive toxicity. Compared with TMZ, GS@DFP not only suppresses tumor cell mitochondrial function but also effectively disrupts mitochondrial transfer and microtubule support from astrocytes to tumor cells, thereby achieving comprehensive modulation of the tumor microenvironment. This dual-targeted strategy represents the core innovation of this study and underlies the observed efficacy.

3. Scientific rationale

Based on the experimental data, TMZ remains a valid reference control, while the advantages observed for GS@DFP primarily reflect its mechanism-focused intervention. Overall, the inclusion of this experimental candidate is scientifically justified and necessary for the study design.

9. Mitochondrial function is known critical for the immune microenvironment, including T cells and other immune populations. Thus, mitochondrial inhibition may impair antitumor immune responses or other tumor-suppressive factors. The Discussion section should consider effects of both sides.

Response: We thank the reviewer for highlighting the importance of mitochondrial function in the immune microenvironment, as well as the potential impact of

mitochondrial inhibition on antitumor immunity. We have supplemented the Discussion section in the revised manuscript, with the following clarifications:

1. Bidirectional role of mitochondrial function in immune regulation

We appreciate the reviewer's insightful comment regarding the potential bidirectional effects of mitochondrial inhibition strategies. We agree that, while targeting tumor mitochondrial metabolism can effectively reduce tumor growth and astrocyte-mediated tumor support, it may also have potential impacts on the metabolic activity of certain immune cells.

2. Balancing the bidirectional effect

We fully acknowledge that mitochondria are critical for energy supply, activation, and functional maintenance in T cells, NK cells, and other immune cells. Accordingly, inhibition of mitochondria within tumor cells may not only directly suppress tumor proliferation but also indirectly influence antitumor responses by modulating the immune microenvironment. Therefore, in designing therapeutic strategies, it is essential to balance tumor mitochondrial inhibition with the preservation of normal immune function.

3. Considerations in experimental design

In this study, our drug primarily targets tumor cells and the mitochondrial transfer between astrocytes and tumor cells, modulating the supportive tumor microenvironment rather than broadly disrupting mitochondria in all immune cells. At the same time, we must emphasize that the Gboxin series of drugs has been tested for its selectivity since its development. Compared to normal cells such as embryonic fibroblasts and astrocytes, the Gboxin series of drugs exhibits lower half maximal inhibitory concentration and harm (**Nature 567, 341–346 (2019).**). And in our in vivo experiments, the activity of T cells, NK cells, and macrophages was not significantly impaired; in some cases, their function appeared improved under partially immunosuppressive conditions. This suggests that the drug can suppress tumor-supportive pathways without substantially compromising antitumor immunity.

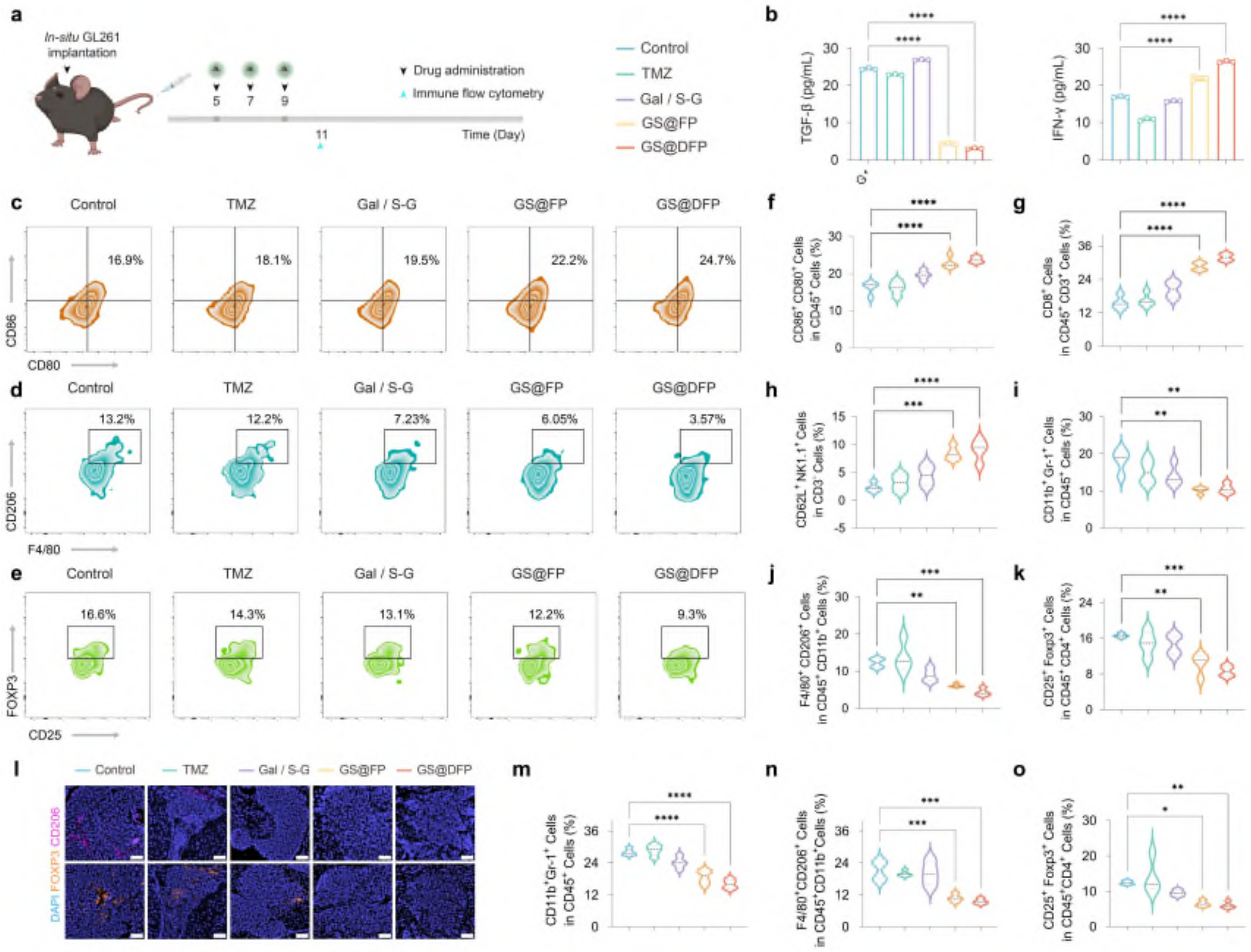

Fig. 7 | GS@DFP effectively inhibits tumor growth *in vivo*. **a** Schematic illustration of the establishment and treatment schedule of the GL261-Luci tumor-bearing mouse model; **b** Body weight changes of tumor-bearing mice treated with PBS, TMZ, equivalent doses of Galunisertib and S-Gboxin *via* intravenous injection, GS@FP, or GS@DFP over 20 days (n=6 mice in each group); **c** Survival curves of tumor-bearing mice with different treatment (n=10 mice in each group); **d** *In vivo* bioluminescence imaging of GL261 tumor-bearing mice, with black boxes representing deceased mice; **e** Quantitative analysis of normalized individual brain bioluminescence intensity of tumor-bearing mice after different treatments (n=6 mice in each group). **f** Quantitative results of brain bioluminescence imaging of tumor-bearing mice. **g** Western blot analysis and quantitative results of pSMAD2/3 and TSP-1 expression in tumor tissues from each treatment group (n=3 mice in each group). ns>0.05, *$p < 0.05$, **$p < 0.01$, ***$p < 0.001$, ****$p < 0.0001$.

In the revised manuscript (**lines 484–496**), we have added relevant discussions:

The ability of GS@DFP to simultaneously target mitochondria transferred from astrocytes and the hyperactive tumor mitochondria driven by GBM's high metabolic demands is central to its therapeutic efficacy. GBM, as one of the most aggressive brain tumors, exhibits strong network-forming behavior and can co-opt surrounding neuronal and astrocytic ecosystems, including the highly malignant process of mitochondrial transfer. Galunisertib attenuates astrocyte-derived support by inhibiting tumor microtubules and disrupts the TGF-β-driven immunosuppressive core of the TME. In addition to TGF-β pathway inhibition, mitochondrial disruption and ROS generation also contribute to immune remodeling, acting in parallel to reinforce the antitumor immune response. Meanwhile, although intense metabolic activity promotes tumor evolution, it also exposes metabolic vulnerabilities. As a selective ATP synthase inhibitor, S-Gboxin readily disrupts tumor mitochondria and demonstrates substantially greater cytotoxicity than TMZ. Although mitochondrial function is essential for immune cells, the tumor-targeted and ROS-responsive nature of GS@DFP restricts this effect predominantly to tumor regions, thereby preserving overall immune competence. The ROS-responsive polymer nanoparticles modified with a glucose analogue enable efficient co-delivery of both therapeutics. After crossing the BBB, GS@DFP releases its payload in response to elevated ROS levels, achieving precise targeting of both tumor mitochondria and mitochondria acquired via intercellular transfer.

In the current manuscript, this point is considered as part of the overall safety and biological context of the strategy. Our design employs targeted delivery and controlled dosing to preferentially affect tumor cells, thereby aiming to maximize antitumor efficacy while minimizing potential adverse effects on antitumor immunity. We further acknowledge that a more precise balance between tumor metabolic inhibition and immune preservation remains an important consideration for future investigations, particularly in optimizing mitochondrial-targeting strategies within the

tumor microenvironment.

Minor comments:

1. Figure 5b: missing a “)”.

2. Line 331: misuse of spacing.

Response: We thank the reviewer for pointing out these minor errors. In the revised manuscript, we have: corrected Figure 5b by adding the missing closing parenthesis “)”; and fixed the spacing issue in line 331 to ensure proper formatting. These corrections have been implemented in the revised version.
